# Species Diversity of *Penicillium* in Southwest China with Discovery of Forty-Three New Species

**DOI:** 10.3390/jof9121150

**Published:** 2023-11-28

**Authors:** Xin-Cun Wang, Zhi-Kang Zhang, Wen-Ying Zhuang

**Affiliations:** 1 State Key Laboratory of Mycology, Institute of Microbiology, Chinese Academy of Sciences, Beijing 100101, China; zhuangwy@im.ac.cn; 2 School of Agriculture, Ludong University, Yantai 264025, China

**Keywords:** *Aspergillaceae*, biodiversity, *Eurotiales*, phylogeny, soil fungi, taxonomy

## Abstract

*Penicillium* species are ubiquitous in all kinds of environments, and they are of industrial, agricultural and clinical importance. In this study, soil fungal diversity in Southwestern China was investigated, and that of *Penicillium* turned out to be unexpectedly high. The survey included a total of 179 cultures of the genus isolated from 33 soil samples. Three-locus phylogenetic analyses and morphological comparisons were carried out. The examinations revealed that they belonged to two subgenera (*Aspergilloides* and *Penicillium*), 11 sections (*Aspergilloides*, *Canescentia*, *Citrina*, *Exilicaulis*, *Fasciculata*, *Gracilenta*, *Lanata-Divaricata*, *Penicillium*, *Ramosum*, *Robsamsonia*, and *Sclerotiorum*), 25 series, and 74 species. Forty-three species were discovered as new to science, and a new series, *Simianshanica*, was established in sect. *Aspergilloides*. Additionally, 11 species were recorded for the first time in China. Species isolation frequency and distribution of the group were also discussed.

## 1. Introduction

Species of *Penicillium* are ubiquitous in environments worldwide and are of industrial, agricultural, and clinical importance. They can be isolated from various substrates: soil, air, fresh water, marine sediments, plants, animals, food, indoor environments, infected human-beings, etc. They have been reported across the world: deserts [1,2], the Arctic [3,4], the Antarctic [5,6,7], high-altitude glaciers [8], and even in the mesosphere [9,10]. They also have an immense impact on human societies. *Penicillium janthinellum* Biourge produces xylanases which are widely adopted in the food and pharmaceutical industries [11]. *Penicillium decumbens* Thom has been utilized for cellulolytic enzyme production at an industrial scale, and a novel cellobiohydrolase purified from it was discovered to be efficient for bioethanol production [12]. For food fermentation, *P. nalgiovense* Laxa and *P. salamii* G. Perrone et al. are used in the production of sausages [13], and *P. camemberti* Thom and *P. roqueforti* Thom are used in the production of white and blue cheeses, respectively [14]. *Penicillium simplicissimum* (Oudem.) Thom was reported to significantly increase the release of phosphorus, potassium, calcium, and magnesium from rocks and promote leguminous plant growth for bioremediation [15]. Penicillin, originally discovered in *P. rubens* Biourge, the first antibiotic, has saved millions of lives throughout history [16], and is mainly produced by *P. chrysogenum* Thom [17]. On the other hand, *Penicillium* species cause food spoilage and produce many kinds of mycotoxins, e.g., nephrotoxic citrinin and hepatotoxic patulin produced by *P. expansum* Link [18]. *Penicillium digitatum* (Pers.) Sacc. is the major causal agent of postharvest decay in citrus fruits [19]; recently, it was even reported in a COVID-19 patient, causing pulmonary co-infection as an extremely rare human pathogen [20].

This genus was established by Link in 1809 with *P. expansum* as its type species. According to the monographic study, *Penicillium* was classified into two subgenera, 32 sections, 89 series, and 483 species [21]. More recently, additional 75 new species of this genus were described from different countries (by the end of September 2023), e.g., *P. donggangicum* L. Wang, *P. linzhiense* H.K. Wang & R. Jeewon and *P. soli* Doilom et al. from China [22,23,24], *P. fusiforme* B.D. Sun et al. from Netherlands [25], *P. melanosporum* Rodr.-Andr. et al. from Spain [26], *P. claroviride* Visagie & Yilmaz from South Africa [27], *P. silybi* Labuda et al. from USA [28], *P. vascosobrinhoanum* R.N. Barbosa & J.D.P. Bezerra from Brazil [29], and *P. allaniae* Y.P. Tan et al. from Australia [30]. There are more than 550 species currently recognized in *Penicillium*.

In China, 77 *Penicillium* species were recorded in Flora Fungorum Sinicorum vol. 35 *Pennicilium* et Teleomorphi Cognati [31]. Among them, eight were typified by the Chinese materials, i.e., *P. guizhouanum* H.Z. Kong from Guizhou Province, *P. heteromorphum* H.Z. Kong & Z.T. Qi, *P. nodulum* H.Z. Kong & Z.T. Qi and *P. shennongjianum* H.Z. Kong & Z.T. Qi from Hubei, *P. incoloratum* L.Q. Huang & Z.T. Qi from Beijing, *P. jiangxiense* H.Z. Kong & Z.Q. Liang from Jiangxi, and *P. formosanum* H.M. Hsieh et al. and *P. ulaiense* H.M. Hsieh et al. from Taiwan Province. Afterwards, 40 new species of the genus were further added: 14 in Hainan (*P. sanshaense* X.C. Wang & W.Y. Zhuang, *P. austrosinense* L. Cai et al., *P. flaviroseum* L. Cai & X.Z. Jiang, *P. globosum* L. Cai et al., *P. griseoflavum* L. Cai & X.Z. Jiang, *P. hainanense* L. Cai & X.Z. Jiang, *P. jianfenglingense* L. Cai & X.Z. Jiang, *P. laevigatum* L. Cai et al., *P. rubriannulatum* L. Cai & X.Z. Jiang, *P. soliforme* L. Cai & X.Z. Jiang, *P. spinuliferum* L. Cai & X.Z. Jiang, *P. viridissimum* L. Cai & X.Z. Jiang, *P. danzhouense* C. Liu et al. and *P. tenue* C. Liu et al.), *P. austrosinicum* X.C. Wang & W.Y. Zhuang, *P. exsudans* X.C. Wang & W.Y. Zhuang and *P. zhanjiangense* C. Liu et al. from Guangdong, *P. guangxiense* L. Cai & X.Z. Jiang and *P. hepuense* L. Wang from Guangxi, *P. choerospondiatis* X.C. Wang & W.Y. Zhuang and *P. verrucisporum* X.C. Wang & W.Y. Zhuang from Hunan, *P. brevistipitatum* L. Wang & W.Y. Zhuang and *P. saturniforme* (L. Wang & W.Y. Zhuang) Houbraken & Samson from Jilin, *P. persicinum* L. Wang et al. and *P. samsonianum* L. Wang et al. from Qinghai, *P. fusisporum* L. Wang and *P. zhuangii* L. Wang from Shaanxi, *P. kongii* L. Wang and *P. linzhiense* from Tibet, *P. yunnanense* L. Cai & X.Z. Jiang and *P. soli* from Yunnan, *P. chroogomphum* F. Xu et al. from Beijing, *P. macrosclerotiorum* L. Wang et al. from Chongqing, *P. fujianense* Z.Y. Zhang et al. from Fujian, *P. glycyrrhizacola* A.J. Chen et al. from Gansu, *P. terrarumae* Houbraken et al. from Guizhou, *P. compactum* L. Wang & Houbraken from Heilongjiang, *P. donggangicum* from Liaoning, *P. jiaozhouwanicum* L. Wang from Shandong, and *P. xinjiangense* A.J. Chen et al. from Xinjiang [22,23,24,32,33,34,35,36,37,38,39,40,41,42,43,44,45]. In total, there are more than 100 *Penicillium* species distributed in China and 48 of them were originally described from this country.

In this study, soil fungal diversity in some areas of Chongqing Municipality and Sichuan Province in Southwestern China was surveyed and unexpectedly high species diversity of *Penicillium* was discovered.

## 2. Materials and Methods

### 2.1. Fungal Materials

Cultures were isolated from soil samples collected from different sites of Chongqing City and a small part of Sichuan Province, Southwestern China, in 2020. Soil fungi were isolated by using the standard dilution plating technique. Four dilution gradients (10^−1^, 10^−2^, 10^−3^ and 10^−4^) were adopted and PDA with chloramphenicol was chosen as the selective medium. Dried cultures were deposited in the Herbarium Mycologicum Academiae Sinicae (HMAS), and living ex-type strains were preserved in the China General Microbiological Culture Collection Center (CGMCC).

### 2.2. Morphological Observations

Morphological characterization was conducted following standardized methods [46]. Four standard growth media were used: Czapek yeast autolysate agar (CYA, yeast extract Oxoid), malt extract agar (MEA, Amresco, Solon, OH, USA), yeast extract agar (YES), and potato dextrose agar (PDA). The methods for inoculation, incubation, microscopic examinations, and digital recordings followed our previous studies [42,47,48,49,50,51].

### 2.3. DNA Extraction, PCR Amplification, and Sequencing

DNA was extracted from the cultures grown on PDA for 7 days using the Plant Genomic DNA Kit (DP305, TIANGEN Biotech, Beijing, China). Polymerase chain reaction (PCR) amplifications of the internal transcribed spacer (ITS), beta-tubulin (BenA), calmodulin (CaM), and RNA polymerase II second largest subunit (RPB2) gene regions were conducted with the routine methods [46]. The products were purified and subject to sequencing on an ABI 3730 DNA Sequencer (Applied Biosystems, Foster, CA, USA). Although the ITS region, the proposed universal DNA barcode for fungi, is helpful to classify a *Penicillium* species at series level, it is not sufficient to distinguish them at species level. ITS sequences are still provided here as they might be beneficial to other researchers.

### 2.4. Phylogenetic Analyses

Forward and reverse sequences newly generated in this study were assembled using Seqman v. 7.1.0 (DNASTAR Inc., Madison, WI, USA). The assembled sequences were deposited at GenBank. The sequences used for phylogenetic analyses are listed in Table 1, Table 2, Table 3, Table 4, Table 5, Table 6 and Table 7. Sequences of the combined loci (BenA, CaM and RPB2) of each of the datasets were aligned using MAFFT v. 7.221 [52], then manually edited and combined in BioEdit v. 7.1.10 [53] and MEGA v. 6.0.6 [54], and analyzed to infer the phylogenies of different groups of *Penicillium*. Maximum likelihood (ML) analyses were conducted using RAxML-HPC2 [55] on XSEDE 8.2.12 on CIPRES Science Gateway v. 3.3 [56] with the default GTRCAT model. Bayesian inference (BI) analyses were performed with MrBayes v. 3.2.5 [57]. Appropriate nucleotide substitution models and parameters were determined using Modeltest v. 3.7 [58]. The consensus trees were viewed in FigTree v. 1.3.1 (http://tree.bio.ed.ac.uk/software/figtree/ (accessed on 3 June 2015)).

## 3. Results

A total of 33 soil samples were collected in 10 days of 2020 from Southwest China, including 28 from Chongqing Municipality, 4 from Dazhou City of Sichuan Province, and 1 from Ankang City of Shaanxi Province. In isolation of the samples, 179 *Penicillium* cultures were obtained and subsequently placed in five sections of subgen. *Penicillium* and six sections of subgen. *Aspergilloides*.

Seven 3-locus (BenA + CaM + RPB2) datasets were correspondingly compiled, i.e., subgen. *Penicillium* (Table 1), sect. *Aspergilloides* (Table 2), sect. *Citrina* (Table 3), sect. *Exilicaulis* (Table 4), sect. *Gracilenta* (Table 5), sect. *Lanata-Divaricata* (Table 6) and sect. *Sclerotiorum* (Table 7) of subgen. *Aspergilloides*. Detailed characteristics of the datasets were given in Table 8. Phylogenies inferred from single gene datasets for each subgenus or section were also given in Supplementary Figures S1–S21.

**Figure 1:**
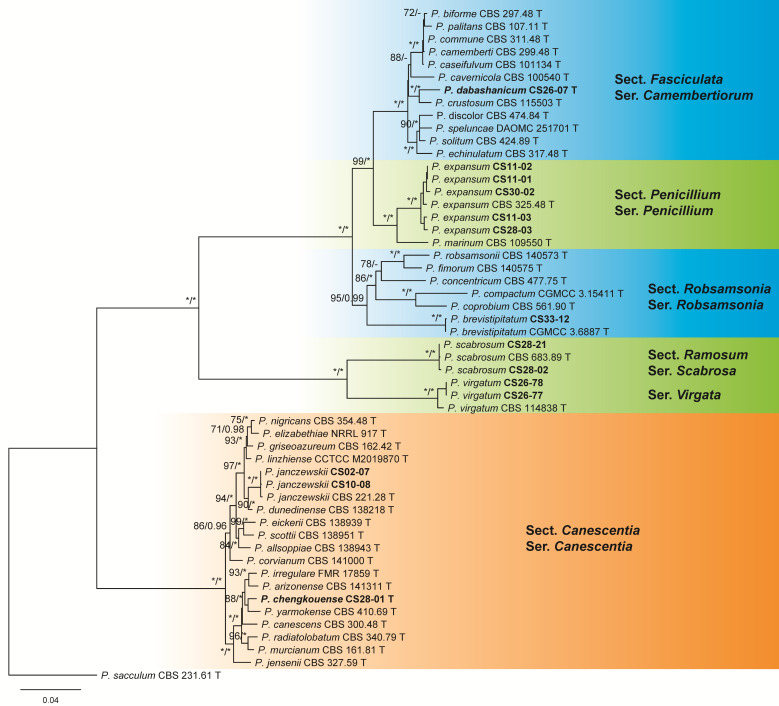
Maximum likelihood phylogeny of *Penicillium* subgen. *Penicillium* inferred from the combined BenA, CaM, and RPB2 dataset. Bootstrap values ≥ 70% (**left**) or posterior probability values ≥ 0.95 (**right**) are indicated at nodes. Asterisk denotes 100% bootstrap or 1.00 posterior probability.

Abbreviations of models: GTR + I + G (general time reversible model with invariant sites and Gamma distribution); SYM + I + G (symmetrical model with invariant sites and Gamma distribution); TIMef + G (equal-frequency transition model with Gamma distribution); TrN + I + G (Tamura–Nei model with invariant sites and Gamma distribution); TrN+G (Tamura–Nei model with Gamma distribution); TVM + I + G (transversion model with invariant sites and Gamma distribution).The dataset of subgen. *Penicillium* contained 53 samples including 38 ex-type cultures of the known species belonging to different series of sects. *Canescentia*, *Fasciculata*, *Penicillium*, *Ramosum*, and *Robsamsonia*, 14 newly isolated cultures, and 1 outgroup of sect. *Eladia*. Twelve isolates were identified as five known species, but two cultures (CS 28-01 and CS 26-07) represented two new species in ser. *Canescentia* of sect. *Canescentia* and ser. *Camembertiorum* of sect. *Fasciculata*, respectively (Figure 1 and Figures S1–S3, Table 1).

**Figure 2:**
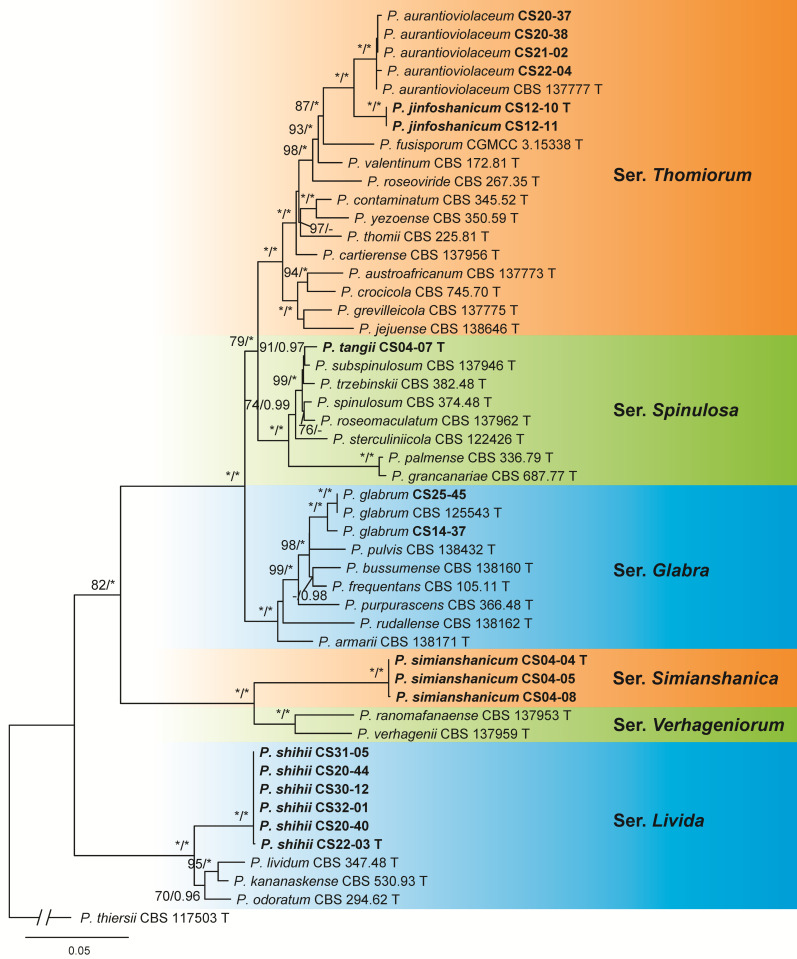
Maximum likelihood phylogeny of *Penicillium* subgen. *Aspergilloides* sect. *Aspergilloides* inferred from the combined BenA, CaM, and RPB2 dataset. Bootstrap values ≥ 70% (**left**) or posterior probability values ≥ 0.95 (**right**) are indicated at nodes. Asterisk denotes 100% bootstrap or 1.00 posterior probability.

The dataset of sect. *Aspergilloides* contained 50 samples including 31 ex-type cultures of the known species belonging to sers. *Glabra*, *Livida*, *Spinulosa*, *Thomiorum* and *Verhageniorum*, 18 isolates from this study, and 1 outgroup of ser. *Thiersiorum*. Six isolates were determined as known species, i.e., *P. glabrum* of ser. *Glabra* and *P. aurantioviolaceum* of ser. *Thomiorum*. The remaining 12 represent 4 new species of ser. *Livida*, *Spinulosa*, *Thomiorum* and a newly proposed series (Figure 2 and Figures S4–S6, Table 2).

**Figure 3:**
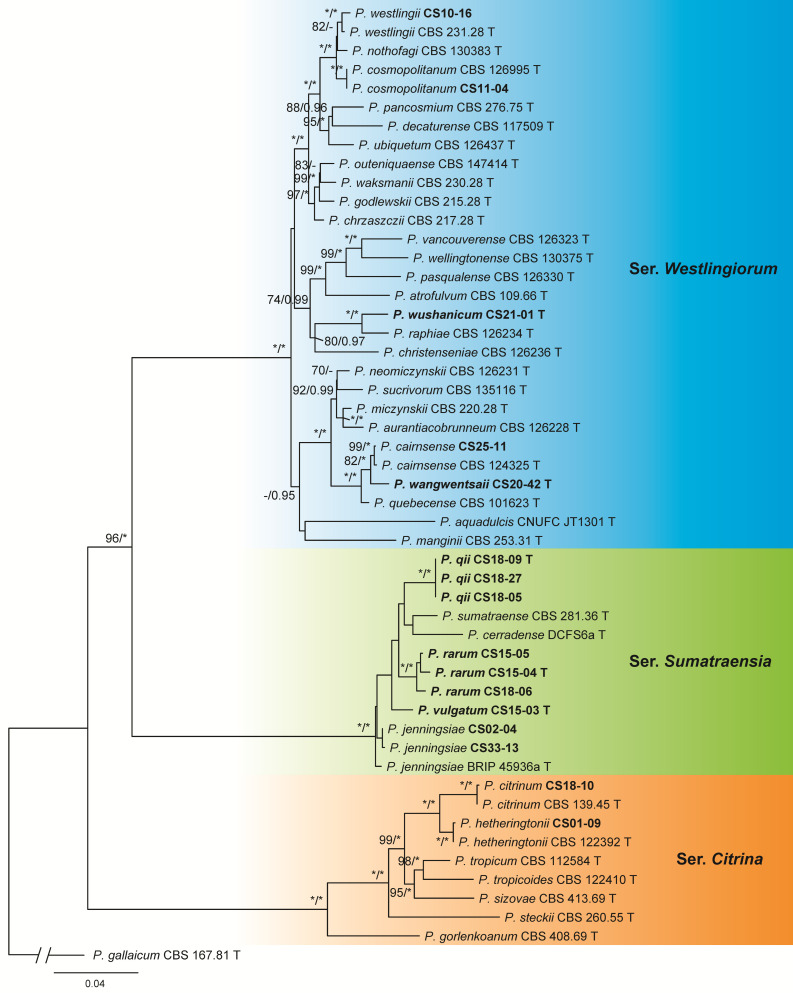
Maximum likelihood phylogeny of *Penicillium* subgen. *Aspergilloides* sect. *Citrina* inferred from the combined BenA, CaM, and RPB2 dataset. Bootstrap values ≥ 70% (**left**) or posterior probability values ≥ 0.95 (**right**) are indicated at nodes. Asterisk denotes 100% bootstrap or 1.00 posterior probability.

The dataset of sect. *Citrina* contained 51 samples including 34 ex-type cultures of the known species belonging to sers. *Citrina*, *Sumatraensia* and *Westlingiorum*, 16 isolates from this study, and 1 outgroup of ser. *Gallaica*. Seven isolates were identified as six known species, while the other nine ones represented five new species: three in ser. *Sumatraensia* and two in ser. *Westlingiorum* (Figure 3 and Figures S7–S9, Table 3).

**Figure 4:**
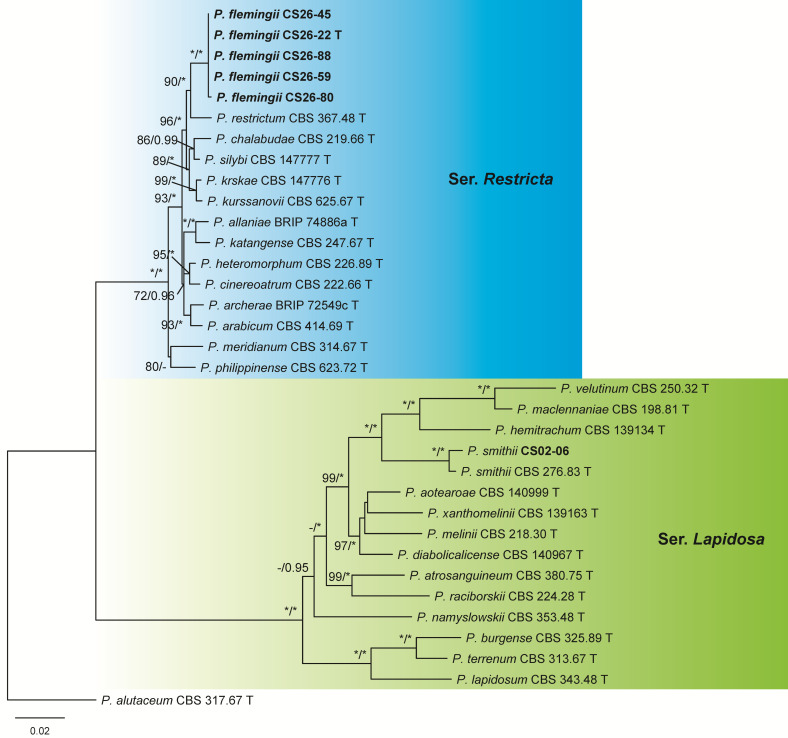
Maximum likelihood phylogeny of *Penicillium* subgen. *Aspergilloides* sect. *Exilicaulis* inferred from the combined BenA, CaM, and RPB2 dataset. Bootstrap values ≥ 70% (**left**) or posterior probability values ≥ 0.95 (**right**) are indicated at nodes. Asterisk denotes 100% bootstrap or 1.00 posterior probability.

The dataset of sect. *Exilicaulis* contained 34 samples including 27 ex-type cultures of the known species belonging to sers. *Lapidosa* and *Restricta*, six isolates from this study, and one outgroup of ser. *Alutacea*. CS02-06 was *P. smithii* of ser. *Lapidosa*, while the other five cultures formed a distinct lineage representing a new species in ser. *Restricta* (Figure 4 and Figures S10–S12, Table 4).

**Figure 5:**
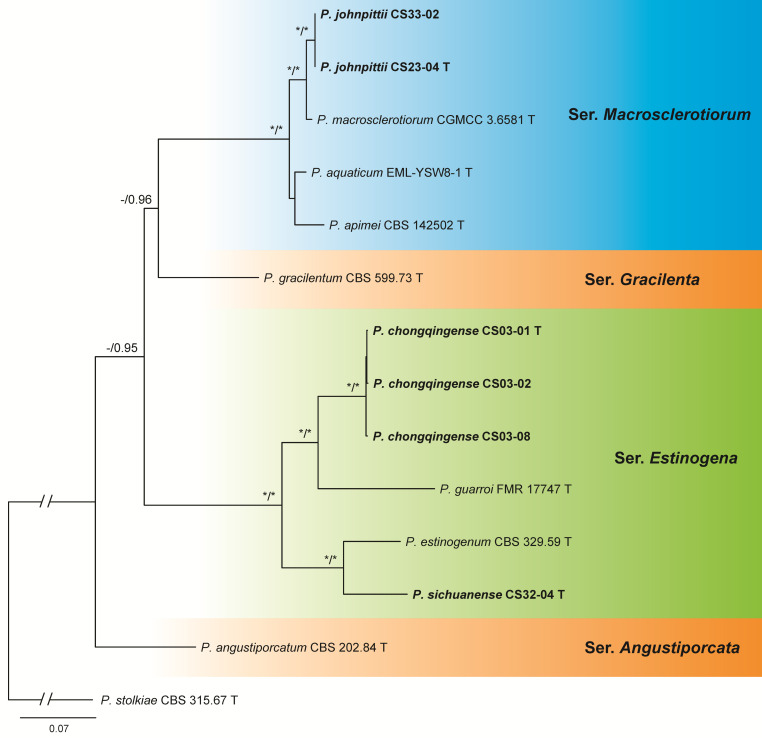
Maximum likelihood phylogeny of *Penicillium* subgen. *Aspergilloides* sect. *Gracilenta* inferred from the combined BenA, CaM, and RPB2 dataset. Bootstrap values ≥ 70% (**left**) or posterior probability values ≥ 0.95 (**right**) are indicated at nodes. Asterisk denotes 100% bootstrap or 1.00 posterior probability.

The dataset of sect. *Gracilenta* contained 14 samples including 7 ex-type cultures of the known species belonging to sers. *Angustiporcata*, *Estinogena*, *Gracilenta*, and *Macrosclerotiorum*, 6 isolates from this study, and 1 outgroup of sect. *Stolkia*. These six isolates were determined as three new species: two in ser. *Estinogena* and one in ser. *Macrosclerotiorum* (Figure 5 and Figures S13–S15, Table 5).

**Figure 6:**
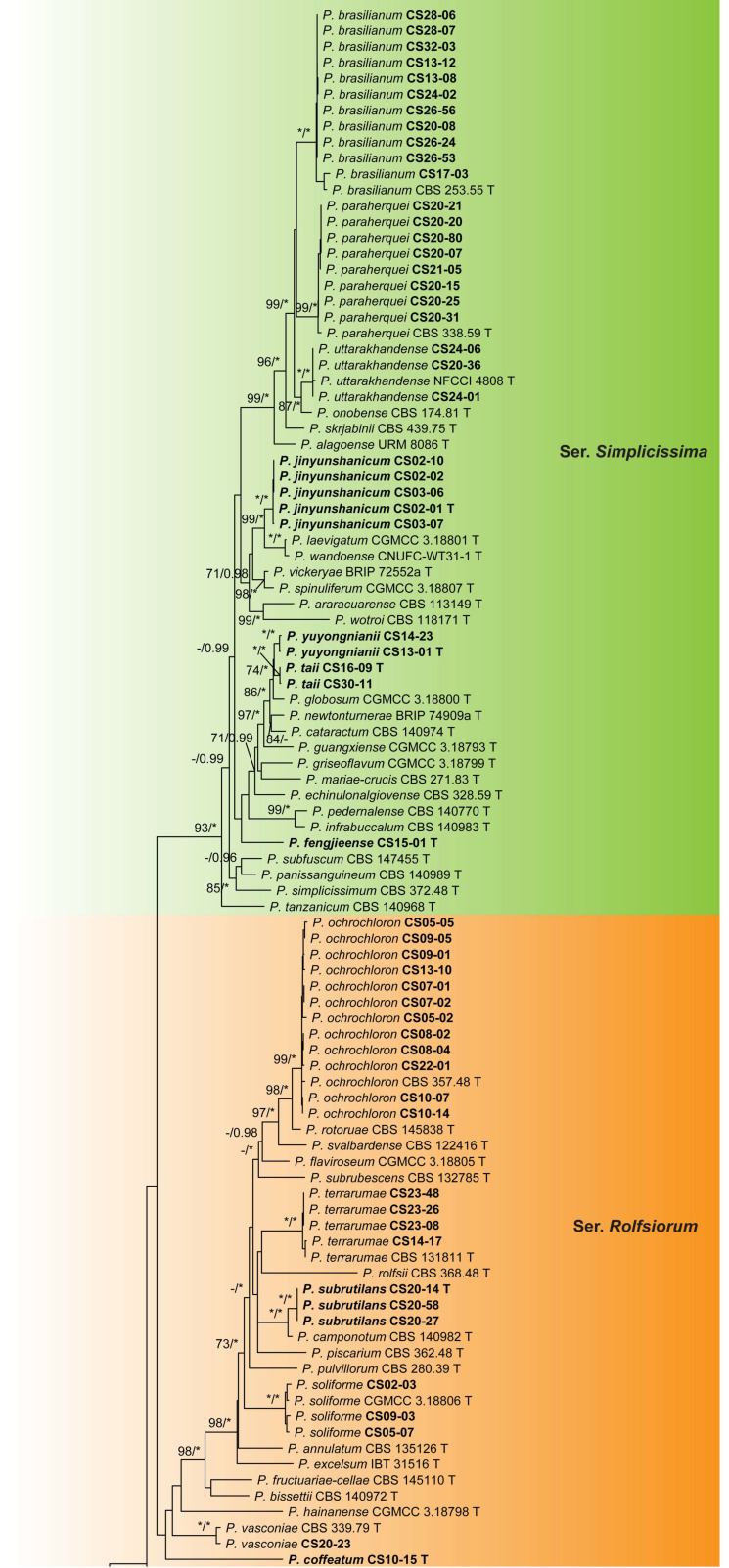

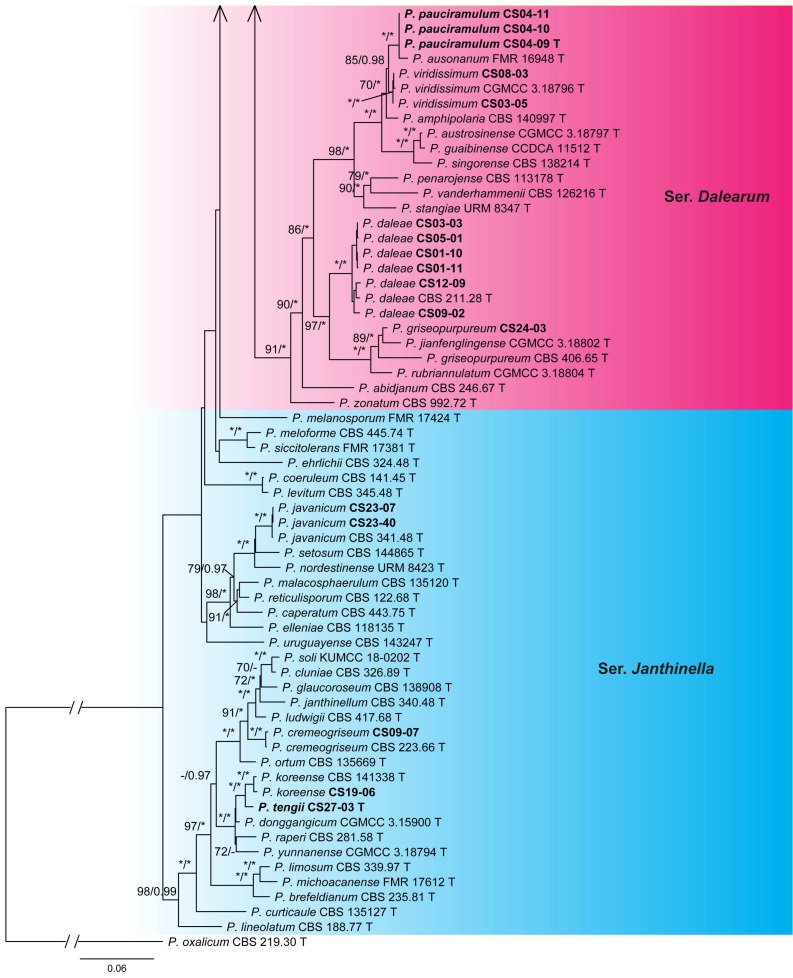
Maximum likelihood phylogeny of *Penicillium* subgen. *Aspergilloides* sect. *Lanata-Divaricata* inferred from the combined BenA, CaM, and RPB2 dataset. Bootstrap values ≥ 70% (**left**) or posterior probability values ≥ 0.95 (**right**) are indicated at nodes. Asterisk denotes 100% bootstrap or 1.00 posterior probability.

The dataset of sect. *Lanata-Divaricata* contained 161 samples including 87 ex-type cultures of the known species belonging to sers. *Dalearum*, *Janthinella*, *Rolfsiorum*, and *Simplicissima*, 73 isolates from this study and 1 outgroup of ser. *Oxalica*. Eighteen isolates represented eight new species: four in ser. *Simplicissima*, two in ser. *Rolfsiorum*, and one in sers. *Dalearum* and *Janthinella*, respectively. The remaining 55 were identified as 13 known species (Figure 6 and Figures S16–S18, Table 6).

**Figure 7:**
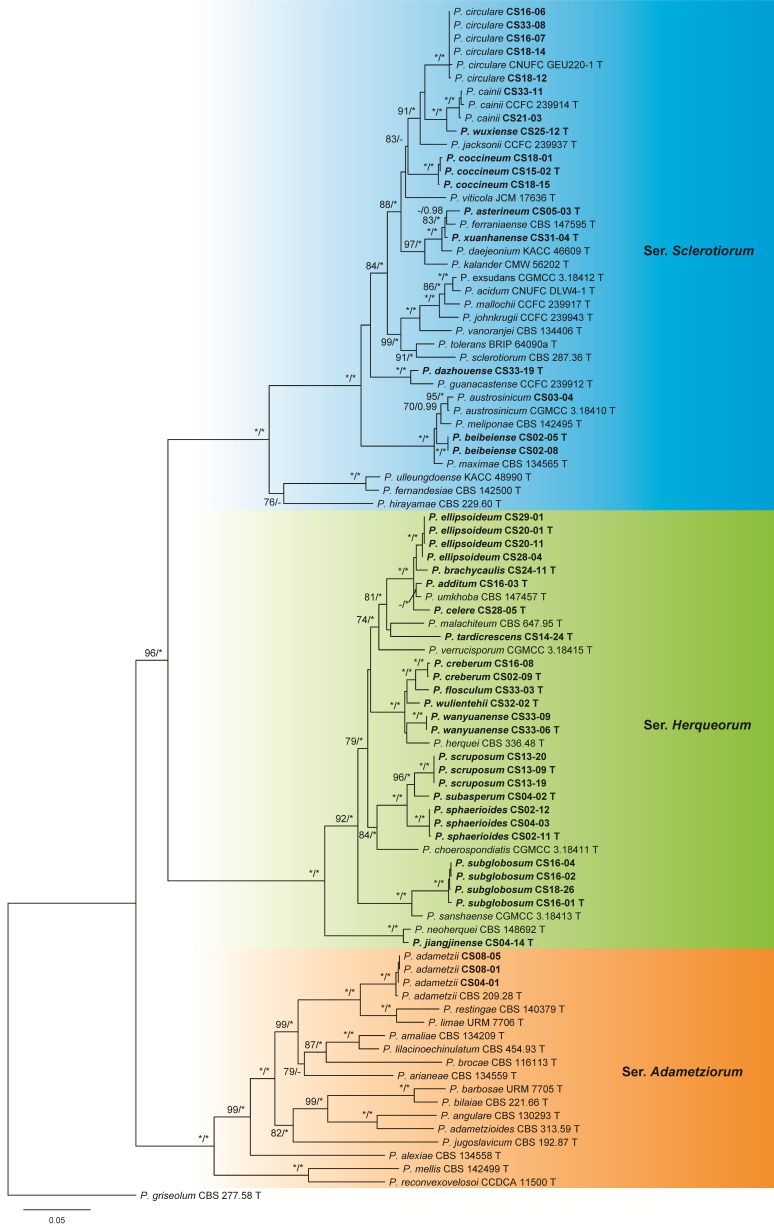
Maximum likelihood phylogeny of *Penicillium* subgen. *Aspergilloides* sect. *Sclerotiorum* inferred from the combined BenA, CaM, and RPB2 dataset. Bootstrap values ≥ 70% (**left**) or posterior probability values ≥ 0.95 (**right**) are indicated at nodes. Asterisk denotes 100% bootstrap or 1.00 posterior probability.

The dataset of sect. *Sclerotiorum* contained 90 samples including 43 ex-type cultures of the known species belonging to sers. *Adametziorum*, *Herqueorum,* and *Sclerotiorum*, 46 isolates from this study, and 1 outgroup of sect. *Griseola*. Eleven isolates could be identified as four known species of sers. *Adametziorum* and *Sclerotiorum*. The remaining 35 represented 20 undescribed new species: 14 in ser. *Herqueorum* and 6 in ser. *Sclerotiorum* (Figure 7 and Figures S19–S21, Table 7).

## 4. Taxonomy

### 4.1. New Series

***Penicillium* series *Simianshanica*** X.C. Wang & W.Y. Zhuang, ser. nov.

**Fungal Names:** FN571493.

**Etymology:** Named after the type species of the series, *Penicillium simianshanicum*.

**Type species:** *Penicillium simianshanicum* X.C. Wang & W.Y. Zhuang.

In *Penicillium* subgenus *Aspergilloides* section *Aspergilloides*.

**Diagnosis:** Series *Simianshanica* is phylogenetically close to ser. *Verhageniorum* (Figure 2). The species of this series is of monoverticillate conidiophores, different from the ones in ser. *Verhageniorum* having biverticillate or divaricate conidiophores [59]. Additionally, it is also characterized by white mycelia, wide margins of the colonies, and bluish green conidia *en masse* on the four media, rough-walled stipes and subglobose and rough-walled conidia.

### 4.2. New Species

**Figure 8:**
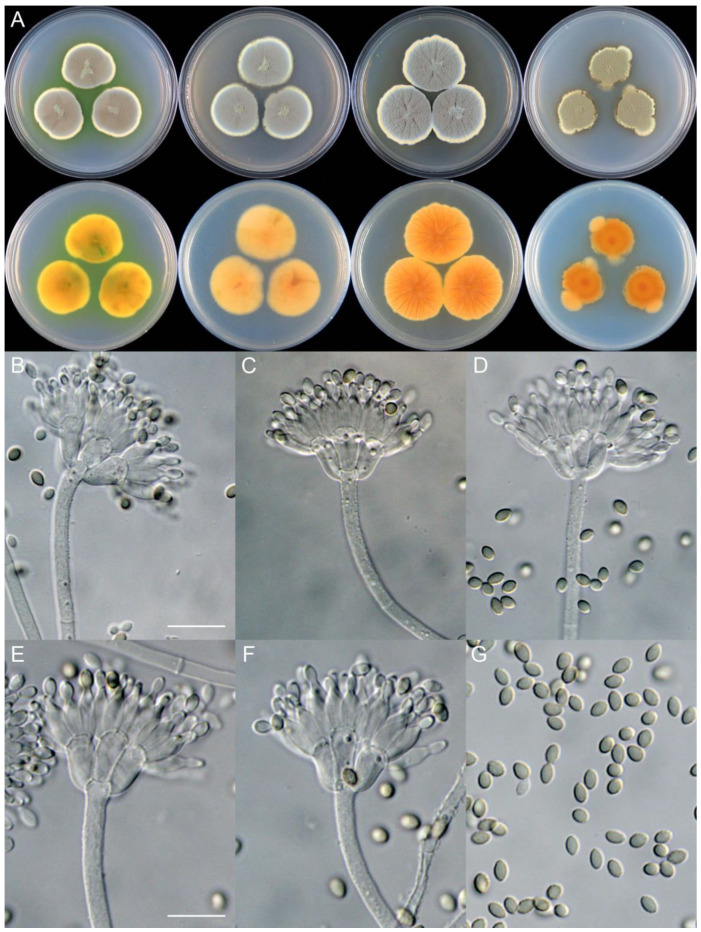
*Penicillium additum* (CS16-03). (**A**) Colonies: top row left to right, obverse CYA, MEA, YES, and PDA; bottom row left to right, reverse CYA, MEA, YES, and PDA; (**B**–**F**) Conidiophores; (**G**) Conidia. Bars: (**B**) = 12.5 µm, also for (**C**,**D**); (**E**) = 10 µm, also for (**F**,**G**).

***Penicillium additum*** X.C. Wang & W.Y. Zhuang, sp. nov. Figure 8.

**Fungal Names:** F N571533.

**Etymology:** The specific epithet refers to the protrusions produced at colony margins on PDA.

In *Penicillium* subgenus *Aspergilloides* section *Sclerotiorum* series *Herqueorum*.

**Typification:** China. Chongqing City, Fengjie County, Caotang Town, 31°5′29″ N 109°38′57″ E, in soil, 29 October 2020, Xin-Cun Wang, Huan-Di Zheng and Chang Liu, culture, Zhi-Kang Zhang, CS16-03 (holotype HMAS 247884, ex-type strain CGMCC 3.25145).

**DNA barcodes:** ITS OQ870831, BenA OR051180, CaM OR051355, RPB2 OR062046.

**Colony diam.**, 7 days, 25 °C (unless stated otherwise): CYA 28–30 mm; CYA 37 °C no growth; CYA 5 °C no growth; MEA 30–34 mm; YES 35–37 mm; PDA 23–25 mm.

**Colony characteristics:** On CYA 25 °C, 7 days: Colonies nearly circular, slightly sulcate or plain, protuberant at centers; margins narrow, entire; mycelia yellow; texture velutinous; sporulation dense; conidia *en masse* dull green; soluble pigments greenish yellow; exudates absent; reverse yellow brown to light brown.

On MEA 25 °C, 7 days: Colonies nearly circular, protuberant at centers; margins narrow, entire or irregular; mycelia yellow; texture velutinous; sporulation dense; conidia *en masse* dull green; soluble pigments absent; exudates absent; reverse yellow brown.

On YES 25 °C, 7 days: Colonies nearly circular or irregular, radially sulcate, protuberant at centers; margins narrow, entire or irregular; mycelia white and yellow; texture velutinous; sporulation dense; conidia *en masse* greyish green; soluble pigments absent; exudates absent; reverse yellow brown to orange brown.

On PDA 25 °C, 7 days: Colonies irregular, slightly protuberant at centers; margins narrow, irregular; mycelia yellow; texture velutinous; sporulation dense; conidia *en masse* greyish green; soluble pigments absent; exudates absent; reverse yellow brown to orange brown.

**Micromorphology:** Conidiophores biverticillate; stipes rough-walled, 200–575 × 3.0–3.5 μm; metulae 5, 9.0–10.5 × 4.0–6.0 μm; phialides ampulliform, tapering into very thin neck, 5–7 per metula, 8.5–9.5 × 3.0–4.0 μm; conidia oval to broad fusiform, finely rough-walled, 3.5–4.5 × 2.0–3.0 μm.

**Notes:** This species is a sister of *P. umkhoba* (PP = 1.00, Figure 7). It differs from the latter in six bp for BenA, two bp for CaM and seven bp for RPB2. Morphologically, it differs in faster growth rate on YES at 25 °C (35–37 vs. 24–26 mm), rough-walled stipes and shorter phialides (8.5–9.5 vs. 8–13 μm) [27]. The protrusions at colony margins on PDA differs from the traditional concept of *P. herquei*.

**Figure 9:**
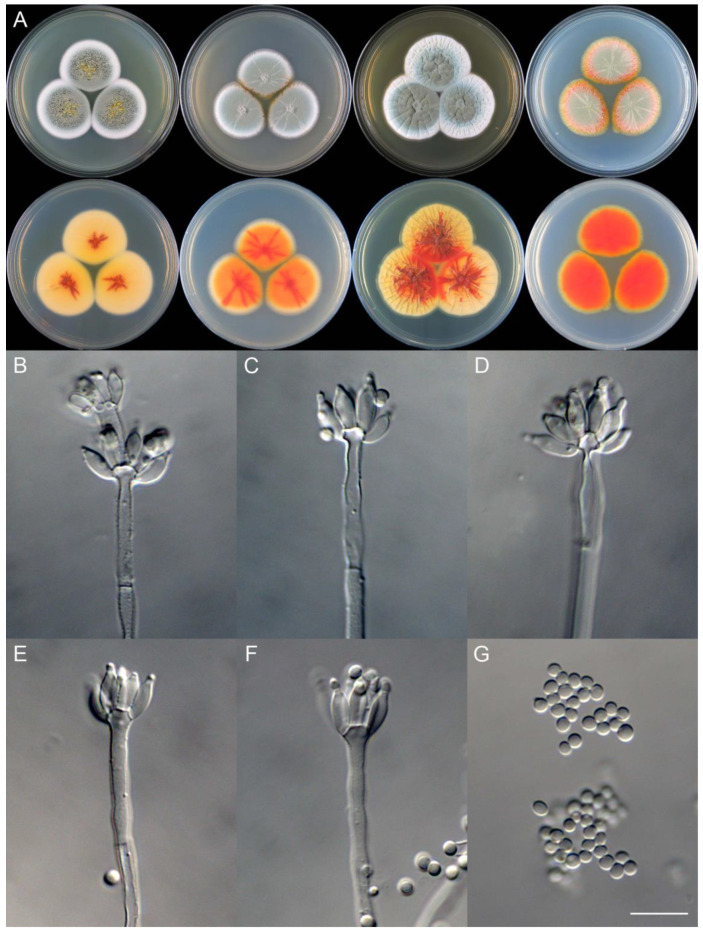
*Penicillium asterineum* (CS05-03). (**A**) Colonies: top row left to right, obverse CYA, MEA, YES, and PDA; bottom row left to right, reverse CYA, MEA, YES, and PDA; (**B**–**F**) Conidiophores; (**G**) Conidia. Bar: (**G**) = 10 µm, also for (**B**–**F**).

***Penicillium asterineum*** X.C. Wang & W.Y. Zhuang, sp. nov. Figure 9.

**Fungal Names:** FN571534.

**Etymology:** The specific epithet refers to the star-like radiate branches in colonies, especially on reverse view.

In *Penicillium* subgenus *Aspergilloides* section *Sclerotiorum* series *Sclerotiorum*.

**Typification:** China. Chongqing City, Jiangjin District, Simian Mountain Nature Reserve, 28°35′57″ N 106°26′51″ E, in soil, 24 October 2020, Chang Liu, Zhao-Qing Zeng, Xin-Cun Wang and Huan-Di Zheng, culture, Zhi-Kang Zhang, CS05-03 (holotype HMAS 247885, ex-type strain CGMCC 3.25146).

**DNA barcodes:** ITS OQ870857, BenA OR051206, CaM OR051381, RPB2 OR062071.

**Colony diam.**, 7 days, 25 °C (unless stated otherwise): CYA 35–40 mm; CYA 37 °C no growth; CYA 5 °C no growth; MEA 34–37 mm; YES 40–42 mm; PDA 35–37 mm.

**Colony characteristics:** On CYA 25 °C, 7 days: Colonies nearly circular, slightly sulcate; margins wide, entire; mycelia white; texture velutinous; sporulation dense; conidia *en masse* dull green; soluble pigments absent; exudates bright yellow, clear, massive; reverse buff to yellow, with red brown radiate branches at centers.

On MEA 25 °C, 7 days: Colonies nearly circular, protuberant at centers, with light-color radiating branches; margins moderately wide, entire or irregular; mycelia white; texture velutinous; sporulation dense; conidia *en masse* greyish green; soluble pigments absent; exudates absent; reverse orange, with red radiate branches.

On YES 25 °C, 7 days: Colonies nearly circular, radially and concentrically sulcate, concave at centers; margins wide, undulated; mycelia white; texture velutinous; sporulation dense; conidia *en masse* bluish green; soluble pigments absent; exudates hyaline, tiny; reverse yellow brown, with red brown radiate branches or patches.

On PDA 25 °C, 7 days: Colonies nearly circular, slightly protuberant at centers, light pinkish orange at margins, with light-colored radiate branches; margins moderately wide, entire; mycelia yellow; texture velutinous; sporulation moderately dense; conidia *en masse* greyish green; soluble pigments absent; exudates absent; reverse red, yellow at margins.

**Micromorphology:** Conidiophores monoverticillate; stipes smooth–rough-walled, 100–300 × 2.5–4.0 μm; phialides ampulliform, tapering into very thin neck, 5–9 per metula, 7.0–10 × 3.0–4.0 μm; conidia subglobose–ellipsoidal, smooth-walled, 2.5–3.0 μm.

**Notes:** This species is a sister of *P. ferraniaense* (PP = 0.98, Figure 7). It differs from the latter in 20 bp for BenA, four bp for CaM and 12 bp for RPB2. Morphologically, it differs in faster growth rate on CYA (35–40 vs. 25–28 mm), MEA (34–37 vs. 25–28 mm) and YES (40–42 vs. 21–23 mm) at 25 °C, and much longer stipes (100–300 vs. 50–80 μm) [60]. The red brown radiate branches on CYA reverse differs this species from the traditional concept of *P. sclerotiorum*.

**Figure 10:**
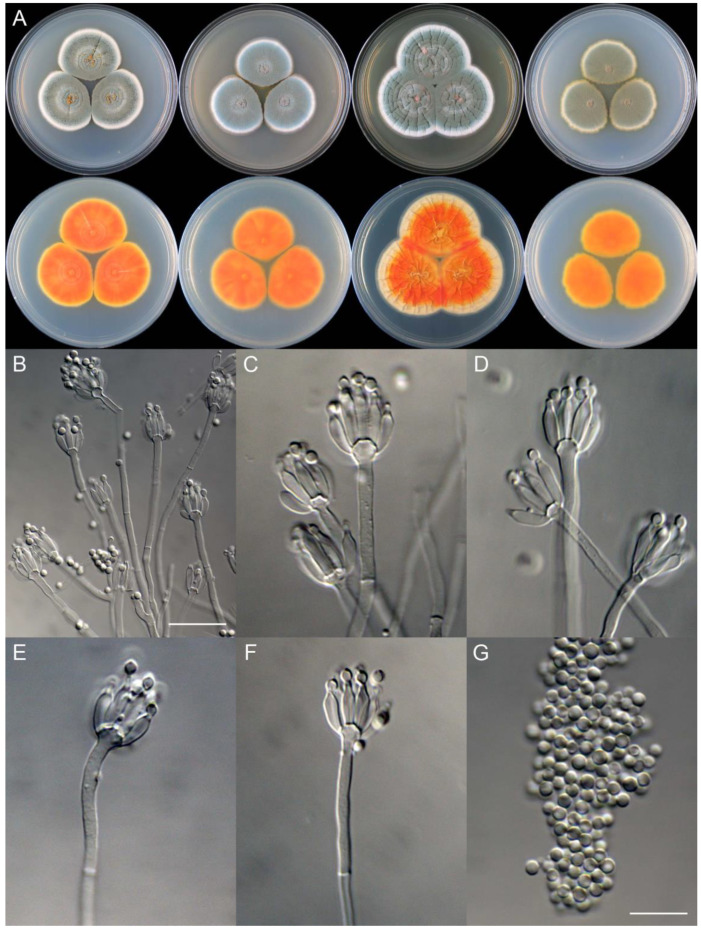
*Penicillium beibeiense* (CS02-05). (**A**) Colonies: top row left to right, obverse CYA, MEA, YES, and PDA; bottom row left to right, reverse CYA, MEA, YES, and PDA; (**B**–**F**) Conidiophores; (**G**) Conidia. Bars: (**B**) = 20 µm; (**G**) = 10 µm, also for (**C**–**F**).

***Penicillium beibeiense*** X.C. Wang & W.Y. Zhuang, sp. nov. Figure 10.

**Fungal Names:** FN571535.

**Etymology:** The specific epithet refers to the type locality.

In *Penicillium* subgenus *Aspergilloides* section *Sclerotiorum* series *Sclerotiorum*.

**Typification:** China. Chongqing City, Beibei District, Jinyun Mountain National Nature Reserve, 29°50′18″ N 106°23′45″ E, in soil, 23 October 2020, Chang Liu, Zhao-Qing Zeng, Xin-Cun Wang and Huan-Di Zheng, culture, Zhi-Kang Zhang, CS02-05 (holotype HMAS 247886, ex-type strain CGMCC 3.25147).

**DNA barcodes:** ITS OQ870859, BenA OR051208, CaM OR051383, RPB2 OR062073.

**Colony diam.**, 7 days, 25 °C (unless stated otherwise): CYA 35–37 mm; CYA 37 °C no growth; CYA 5 °C no growth; MEA 34–36 mm; YES 45–46 mm; PDA 31–33 mm.

**Colony characteristics:** On CYA 25 °C, 7 days: Colonies nearly circular, concentrically and radially sulcate; margins narrow, entire; mycelia white; texture velutinous; sporulation dense; conidia *en masse* vivid green; soluble pigments orange; exudates bright yellow at the centers, but hyaline at margins; reverse orange.

On MEA 25 °C, 7 days: Colonies nearly circular, protuberant at centers; margins narrow, entire; mycelia white; texture velutinous; sporulation dense; conidia *en masse* bluish green; soluble pigments orange; exudates absent; reverse orange.

On YES 25 °C, 7 days: Colonies nearly circular, radially and concentrically sulcate, concave or protuberant at centers, red hyphae present at centers; margins narrow, undulated; mycelia white; texture velutinous; sporulation dense; conidia *en masse* bluish green; soluble pigments absent; exudates absent; reverse cream to somewhat buff at margins.

On PDA 25 °C, 7 days: Colonies nearly circular or irregular, protuberant at centers; margins narrow, irregular; mycelia yellow; texture velutinous; sporulation dense; conidia *en masse* dull green; soluble pigments yellow; exudates absent; reverse orange.

**Micromorphology:** Conidiophores monoverticillate; stipes smooth-walled, 60–165 × 2.0–2.5 μm; phialides ampulliform to acerose, tapering into very thin neck, 7–9 per stipe, 7.5–10 × 2.0–3.0 μm; conidia subglobose, smooth-walled, 2.5–3.0 μm.

**Additional strain examined:** China. Chongqing City, Beibei District, Jinyun Mountain National Nature Reserve, 29°50′18″ N 106°23′45″ E, in soil, 23 October 2020, Chang Liu, Zhao-Qing Zeng, Xin-Cun Wang and Huan-Di Zheng, culture, Zhi-Kang Zhang, CS02-08.

**Notes:** This species is closely related to *P. maximae* (Figure 7). It differs from the latter in 12 bp for BenA, six bp for CaM and 15 bp for RPB2. Morphologically, it differs in vivid green or bluish green conidia *en masse* on CYA and YES at 25 °C, lacking pinkish orange mycelia at margins on MEA, colony reverse orange instead of red brown on CYA, MEA and YES, and subglobose but not ellipsoidal conidia [61].

**Figure 11:**
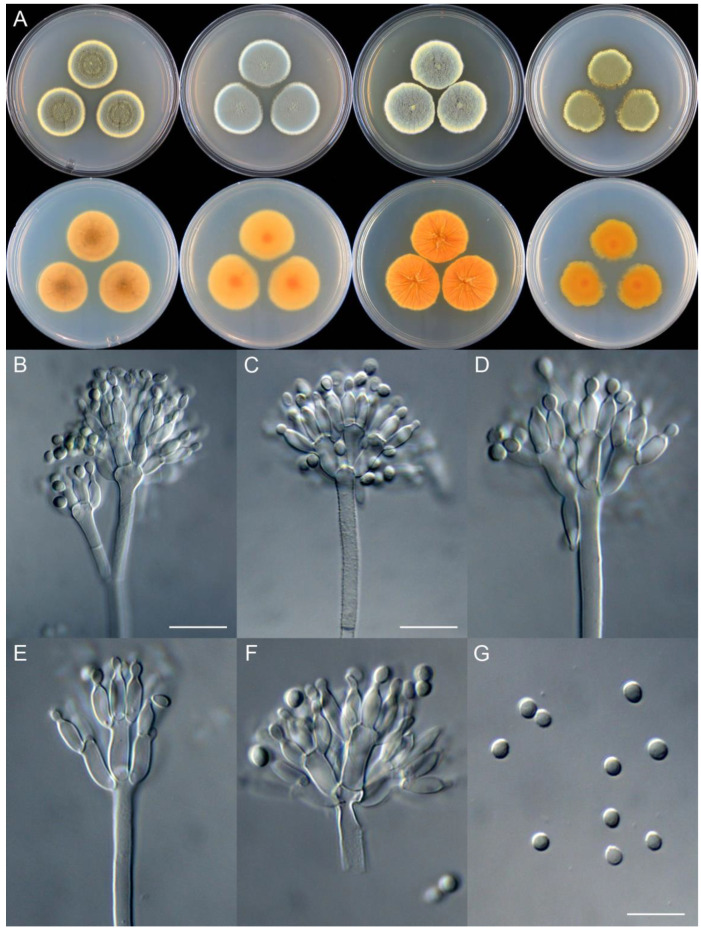
*Penicillium brachycaulis* (CS24-11). (**A**) Colonies: top row left to right, obverse CYA, MEA, YES, and PDA; bottom row left to right, reverse CYA, MEA, YES, and PDA; (**B**–**F**) Conidiophores; (**G**) Conidia. Bars: (**B**) = 15 µm; (**C**) = 12.5 µm; (**G**) = 10 µm, also for (**D**–**F**).

***Penicillium brachycaulis*** X.C. Wang & W.Y. Zhuang, sp. nov. Figure 11.

**Fungal Names:** FN571536.

**Etymology:** The specific epithet refers to the short stipes.

In *Penicillium* subgenus *Aspergilloides* section *Sclerotiorum* series *Herqueorum*.

**Typification:** China. Chongqing City, Wuxi County, Hongchiba National Forest Park, 31°33′3″ N 109°1′36″ E, in soil under *Larix* sp., 30 October 2020, Xin-Cun Wang, Huan-Di Zheng and Chang Liu, culture, Zhi-Kang Zhang, CS24-11 (holotype HMAS 247887, ex-type strain CGMCC 3.25148).

**DNA barcodes:** ITS OQ870832, BenA OR051181, CaM OR051356, RPB2 OR062047.

**Colony diam.**, 7 days, 25 °C (unless stated otherwise): CYA 27–28 mm; CYA 37 °C no growth; CYA 5 °C no growth; MEA 29–30 mm; YES 30–31 mm; PDA 24–26 mm.

**Colony characteristics:** On CYA 25 °C, 7 days: Colonies nearly circular, protuberant at centers, radially sulcate; margins narrow to moderately wide, entire; mycelia yellow; texture velutinous; sporulation dense; conidia *en masse* dull green; soluble pigments yellow; exudates absent; reverse yellow brown to light brown, somewhat brownish at centers.

On MEA 25 °C, 7 days: Colonies nearly circular, funiculose at centers; margins narrow, entire; mycelia yellow; texture velutinous; sporulation dense; conidia *en masse* dull green; soluble pigments absent; exudates absent; reverse light orange, orange at centers.

On YES 25 °C, 7 days: Colonies nearly circular, radially sulcate, concave at centers; margins moderately wide, undulated; mycelia yellow; texture velutinous; sporulation dense; conidia *en masse* yellowish green; soluble pigments absent; exudates absent; reverse yellow brown.

On PDA 25 °C, 7 days: Colonies nearly circular or irregular, slightly funiculose at centers; margins narrow, entire or irregular; mycelia yellow; texture velutinous; sporulation dense; conidia *en masse* dull green; soluble pigments light brown; exudates absent; reverse dirty orange, orange at centers.

**Micromorphology:** Conidiophores biverticillate or terverticillate; stipes smooth to rough-walled, 115–240 × 2.5–4.0 μm; rami 2, 32–33 × 3.5–8.0 μm; metulae 5–6, 9–16 × 3.0–5.0 μm; phialides ampulliform, tapering into very thin neck, 5–8 per metula, 6.5–12 × 2.5–3.5 μm; conidia subglobose to ellipsoidal, smooth to finely rough-walled, 3.0–4.5 × 2.5–4.0 μm.

**Notes:** This species is closely related to *P. ellipsoideum* (Figure 7). It differs from the latter in nine bp for BenA, 11 bp for CaM and nine bp for RPB2. Morphologically, it differs in slower growth rates on CYA (27–28 vs. 34–35 mm) and YES (30–31 vs. 35–37 mm) at 25 °C, faster growth rate on PDA (24–26 vs. 20–21 mm), lacking dark green patches on reverse of CYA, longer metulae (9–16 vs. 8–13.5 μm), and subglobose conidia. The shorter stipes differs from the traditional concept of *P. herquei*.

**Figure 12:**
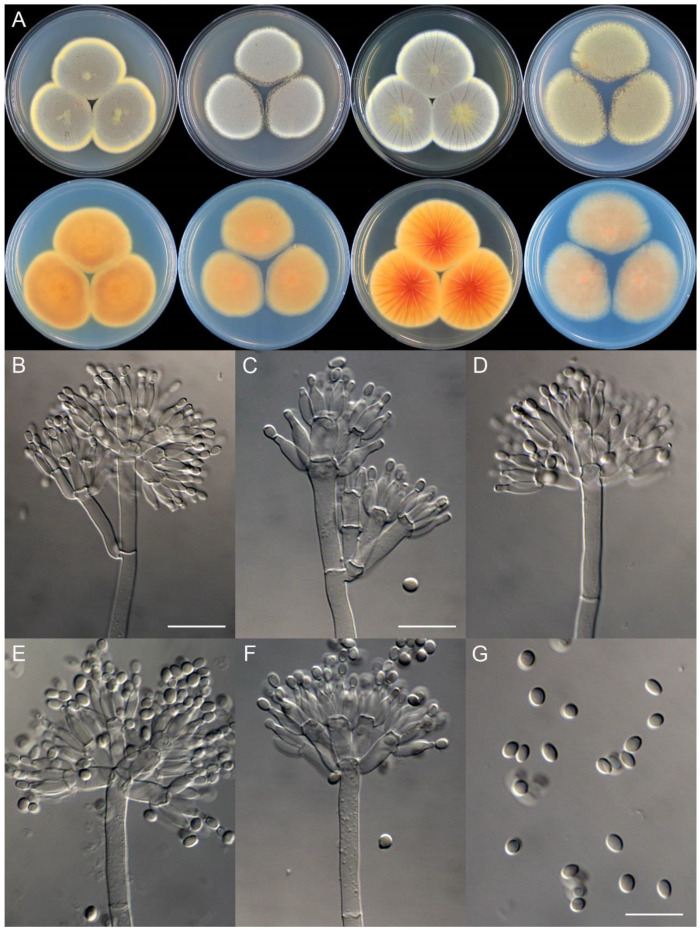
*Penicillium celere* (CS28-05). (**A**) Colonies: top row left to right, obverse CYA, MEA, YES, and PDA; bottom row left to right, reverse CYA, MEA, YES, and PDA; (**B**–**F**) Conidiophores; (**G**) Conidia. Bars: (**B**) = 15 µm; (**C**) = 12.5 µm, also for (**D**–**F**); (**G**) = 10 µm.

***Penicillium celere*** X.C. Wang & W.Y. Zhuang, sp. nov. Figure 12.

**Fungal Names:** FN571560.

**Etymology:** The specific epithet refers to the fast growth rate on PDA.

In *Penicillium* subgenus *Aspergilloides* section *Sclerotiorum* series *Herqueorum*.

**Typification:** China. Chongqing City, Chengkou County, Daba Mountain National Nature Reserve, Beiping Town, 31°58′17″ N 108°47′5″ E, in soil, 31 October 2020, Xin-Cun Wang, Huan-Di Zheng and Chang Liu, culture, Zhi-Kang Zhang, CS28-05 (holotype HMAS 247911, ex-type strain CGMCC 3.25172).

**DNA barcodes:** ITS OQ870848, BenA OR051197, CaM OR051372, RPB2 OR062062.

**Colony diam.**, 7 days, 25 °C (unless stated otherwise): CYA 42–44 mm; CYA 37 °C no growth; CYA 5 °C no growth; MEA 38–39 mm; YES 44–45 mm; PDA 44–46 mm.

**Colony characteristics:** On CYA 25 °C, 7 days: Colonies nearly circular, protuberant at centers; margins moderately wide, entire; mycelia white and yellow; texture velutinous; sporulation dense; conidia *en masse* yellowish green; soluble pigments absent; exudates hyaline, tiny; reverse yellow brown.

On MEA 25 °C, 7 days: Colonies nearly circular, protuberant at centers; margins narrow to moderately wide, entire; mycelia yellow; texture velutinous; sporulation dense; conidia *en masse* dull green; soluble pigments absent; exudates absent; reverse yellow brown.

On YES 25 °C, 7 days: Colonies nearly circular, radially sulcate, protuberant at centers; margins narrow, undulated; mycelia white and yellow; texture velutinous; sporulation dense; conidia *en masse* yellowish green; soluble pigments absent; exudates absent; reverse yellow brown to red brown.

On PDA 25 °C, 7 days: Colonies nearly circular, protuberant at centers; margins narrow, entire; mycelia yellow; texture velutinous; sporulation dense; conidia *en masse* dull green; soluble pigments absent; exudates absent; reverse pale pinkish.

**Micromorphology:** Conidiophores biverticillate or terverticillate; stipes smooth- to finely rough-walled, 275–725 × 4.0–5.0 μm; rami 2–3, 15–28 × 3.5–7.0 μm; metulae 4–7, 10–14 × 3.0–6.0 μm; phialides ampulliform to acerose, tapering into very thin neck, 5–9 per metula, 9–11 × 2.5–3.5 μm; conidia ellipsoidal to broad fusiform, smooth-walled, 3.0–4.5 × 2.0–3.5 μm.

**Notes:** This species appears to be a distinct lineage in ser. *Herqueorum* (Figure 7). Morphologically, it differs from *P. umkhoba* in faster growth rates on CYA (42–44 vs. 28–31 mm), MEA (38–39 vs. 28–32 mm), and YES (44–45 vs. 24–26 mm) at 25 °C, red brown on YES reverse, terverticillate conidiophores and smooth-walled conidia [27]. The faster growth rate on CYA at 25 °C differs from the traditional concept of *P. herquei*.

**Figure 13:**
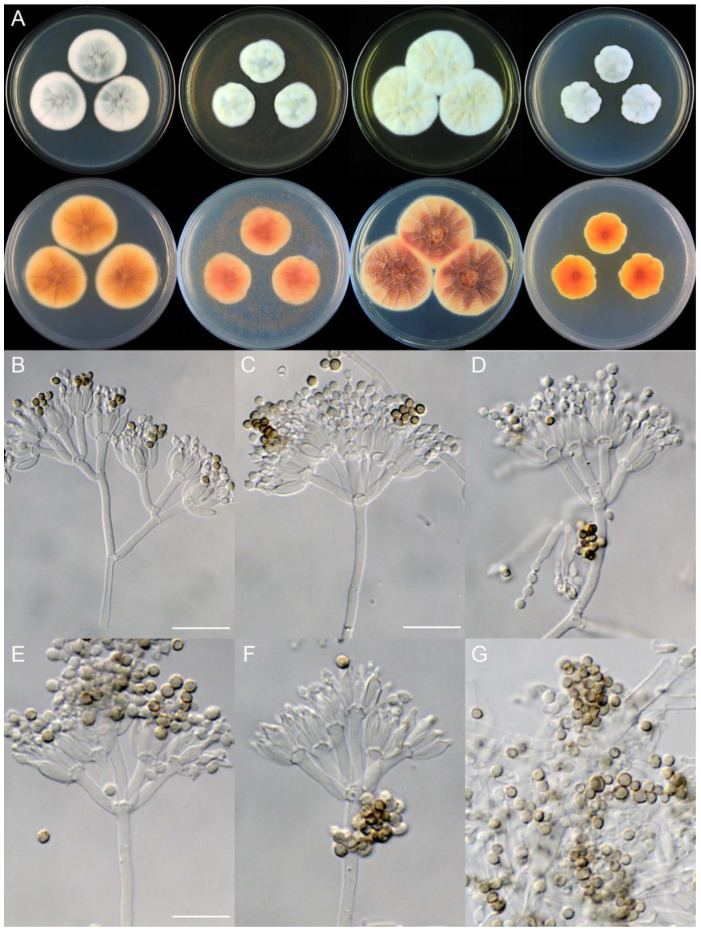
*Penicillium chengkouense* (CS28-01). (**A**) Colonies: top row left to right, obverse CYA, MEA, YES, and PDA; bottom row left to right, reverse CYA, MEA, YES, and PDA; (**B**–**F**) Conidiophores; (**G**) Conidia. Bars: (**B**) = 15 µm; (**C**) = 12.5 µm, also for (**D**); (**E**) = 10 µm, also for (**F**,**G**).

***Penicillium chengkouense*** X.C. Wang & W.Y. Zhuang, sp. nov. Figure 13.

**Fungal Names:** FN571537.

**Etymology:** The specific epithet refers to the type locality.

In *Penicillium* subgenus *Penicillium* section *Canescentia* series *Canescentia*.

**Typification:** China. Chongqing City, Chengkou County, Daba Mountain National Nature Reserve, Beiping Town, 31°58′17″ N 108°47′5″ E, in soil, 31 October 2020, Xin-Cun Wang, Huan-Di Zheng and Chang Liu, culture, Zhi-Kang Zhang, CS28-01 (holotype HMAS 247888, ex-type strain CGMCC 3.25149).

**DNA barcodes:** ITS OQ870783, BenA OR051044, CaM OR051223, RPB2 OR051397.

**Colony diam.**, 7 days, 25 °C (unless stated otherwise): CYA 34–36 mm; CYA 37 °C no growth; CYA 5 °C no growth; MEA 26–27 mm; YES 40–42 mm; PDA 22–25 mm.

**Colony characteristics:** On CYA 25 °C, 7 days: Colonies nearly circular, radially sulcate; margins very wide, entire; mycelia white; texture velutinous; sporulation moderately dense; conidia *en masse* bluish grey; soluble pigments absent; exudates absent; reverse buff to yellow brown.

On MEA 25 °C, 7 days: Colonies irregular, protuberant at centers; margins very wide, irregular; mycelia white and yellow; texture velutinous; sporulation sparse; conidia *en masse* bluish grey; soluble pigments absent; exudates absent; reverse buff to reddish brown.

On YES 25 °C, 7 days: Colonies nearly circular, radially and concentrically sulcate; margins narrow, entire; mycelia white and light yellow; texture velutinous; sporulation very sparse; conidia *en masse* light grey; soluble pigments absent; exudates absent; reverse yellow brown to light brown, with radiate branches and brown patches.

On PDA 25 °C, 7 days: Colonies irregular, protuberant, slight sulcate; margins wide, irregular; mycelia white; texture velutinous; sporulation moderately dense; conidia *en masse* light grey; soluble pigments absent; exudates absent; reverse buff to red brown.

**Micromorphology:** Conidiophores biverticillate, terverticillate to quaterverticillate; stipes smooth-walled, 85–275 × 2.0–2.5 μm; branches 2, 9.0–28 × 2.0–2.5 μm; rami 2, 9.0–32 × 2.0–2.5 μm; metulae 4–6, 8.5–14 × 1.5–3.0 μm; phialides 5–7, acerose to ampulliform, tapering into thin neck, 6.0–8.0 × 2.0–3.0 μm; conidia globose to subglobose, rough-walled, brown, 2.5–3.0 × 2.0–2.5 μm.

**Notes:** This species is phylogenetically close to *P. yarmokense* with strong support (BP = 88, PP = 1.00, Figure 1). It differs from the latter in five bp for BenA, 11 bp for CaM and 11 bp for RPB2. Morphologically, it differs in smooth and shorter stipes (85–275 vs. 400–600 μm) and shorter metulae (8.5–14 vs. 10–20 μm) [62].

**Figure 14:**
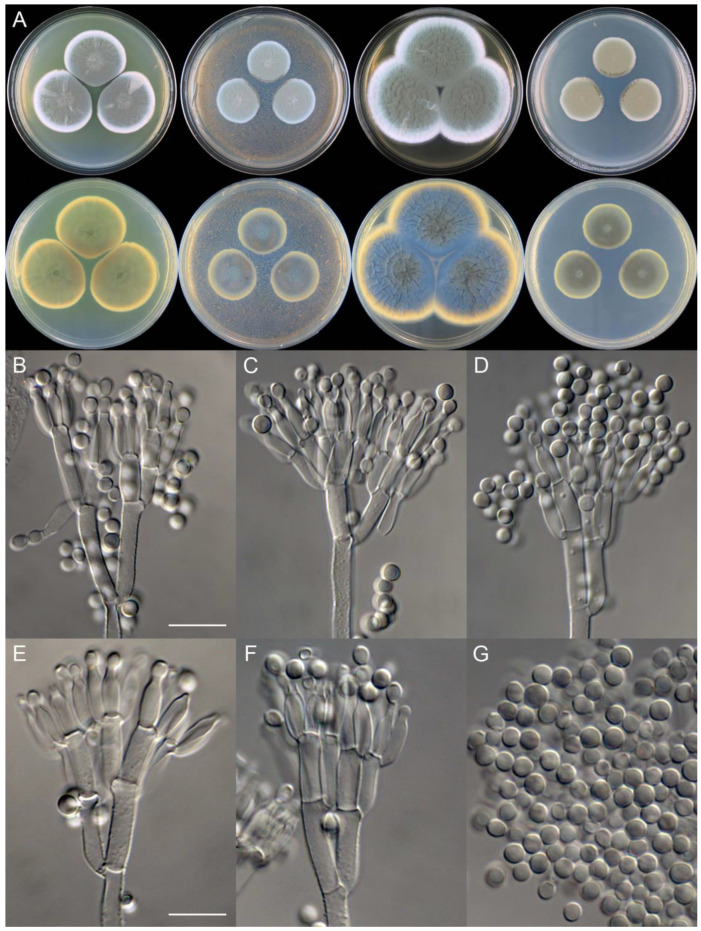
*Penicillium chongqingense* (CS03-01). (**A**) Colonies: top row left to right, obverse CYA, MEA, YES, and PDA; bottom row left to right, reverse CYA, MEA, YES, and PDA; (**B**–**F**) Conidiophores; (**G**) Conidia. Bars: (**B**) = 12.5 µm, also for (**C**,**D**); (**E**) = 10 µm, also for (**F**,**G**).

***Penicillium chongqingense*** X.C. Wang & W.Y. Zhuang, sp. nov. Figure 14.

**Fungal Names:** FN571538.

**Etymology:** The specific epithet refers to the type locality.

In *Penicillium* subgenus *Aspergilloides* section *Gracilenta* series *Estinogena*.

**Typification:** China. Chongqing City, Beibei District, Jinyun Mountain National Nature Reserve, 29°50′18″ N 106°23′45″ E, in soil, 23 October 2020, Chang Liu, Zhao-Qing Zeng, Xin-Cun Wang and Huan-Di Zheng, culture, Zhi-Kang Zhang, CS03-01 (holotype HMAS 247889, ex-type strain CGMCC 3.25150).

**DNA barcodes:** ITS OQ870822, BenA OR051098, CaM OR051275, RPB2 OR051444.

**Colony diam.**, 7 days, 25 °C (unless stated otherwise): CYA 36–38 mm; CYA 37 °C no growth; CYA 5 °C no growth; MEA 25–27 mm; YES 55–56 mm; PDA 25–27 mm.

**Colony characteristics:** On CYA 25 °C, 7 days: Colonies irregular, radially sulcate, slightly protuberant at centers, some with sectors; margins narrow to moderately wide, entire or irregular; mycelia white; texture velutinous; sporulation dense; conidia *en masse* dull green; soluble pigments yellow; exudates yellow, clear; reverse olive, yellow at margins.

On MEA 25 °C, 7 days: Colonies nearly circular, plain, slightly protuberant at centers; margins narrow, entire; mycelia white; texture velutinous to floccose; sporulation dense; conidia *en masse* dull green; soluble pigments absent; exudates absent; reverse bluish brown to greyish, yellowish at margins.

On YES 25 °C, 7 days: Colonies nearly circular, deep, strongly sulcate; margins wide, entire; mycelia white; texture velutinous; sporulation dense; conidia *en masse* dull green; soluble pigments absent; exudates absent; reverse bluish grey, yellow at margins, with radiate branches.

On PDA 25 °C, 7 days: Colonies nearly circular, plain, slightly protuberant at centers; margins narrow, entire or irregular; mycelia white; texture velutinous; sporulation dense; conidia *en masse* dull green; soluble pigments absent; exudates absent; reverse grey, pale grey at centers.

**Micromorphology:** Conidiophores terverticillate, occasionally quaterverticillate; stipes rough-walled, 60–125 × 3.5–4.5 μm; rami 2, 12.5–22.5 × 4.0–4.5 μm; metulae 3–5, 9–19 × 3.5–5.0 μm; phialides ampulliform to acerose, tapering into very thin neck, 4–6 per metula, 8–13 × 2.5–3.5 μm; conidia subglobose, smooth-walled, 3.0–4.5 μm.

**Additional strains examined:** China. Chongqing City, Beibei District, Jinyun Mountain National Nature Reserve, 29°50′18″ N 106°23′45″ E, in soil, 23 October 2020, Chang Liu, Zhao-Qing Zeng, Xin-Cun Wang and Huan-Di Zheng, culture, Zhi-Kang Zhang, CS03-02; *ibid.*, CS03-08.

**Notes:** This species is a sister to *P. guarroi* with strong support (BP = 100, PP = 1.00, Figure 5). It differs from the latter in 53 bp for BenA, 69 bp for CaM and 70 bp for RPB2. Morphologically, it differs in slower growth rate on MEA at 25 °C (25–27 vs. 41–43 mm), faster growth rate on YES (55–56 vs. 49–51 mm), terverticillate instead of biverticillate conidiophores, shorter stipes (60–125 vs. 88–215 μm), much longer metulae and phialides (9–19 vs. 5–10 μm and 8–13 vs. 6–9 μm, respectively) and larger conidia (3.0–4.5 vs. 2.0–2.5 μm) [63].

**Figure 15:**
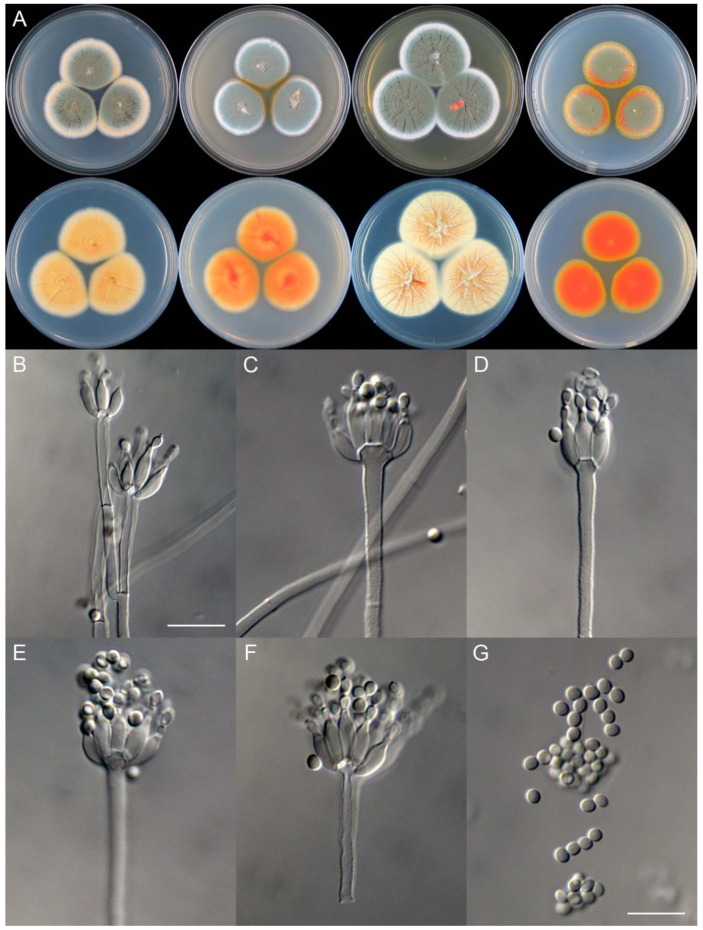
*Penicillium coccineum* (CS15-02). (**A**) Colonies: top row left to right, obverse CYA, MEA, YES, and PDA; bottom row left to right, reverse CYA, MEA, YES, and PDA; (**B**–**F**) Conidiophores; (**G**) Conidia. Bars: (**B**) = 12.5 µm; (**G**) = 10 µm, also for (**C**–**F**).

***Penicillium coccineum*** X.C. Wang & W.Y. Zhuang, sp. nov. Figure 15.

**Fungal Names:** FN571539.

**Etymology:** The specific epithet refers to the red color on PDA reverse.

In *Penicillium* subgenus *Aspergilloides* section *Sclerotiorum* series *Sclerotiorum*.

**Typification:** China. Chongqing City, Fengjie County, Caotang Town, 31°5′29″ N 109°38′57″ E, in soil, 29 October 2020, Xin-Cun Wang, Huan-Di Zheng and Chang Liu, culture, Zhi-Kang Zhang, CS15-02 (holotype HMAS 247890, ex-type strain CGMCC 3.25151).

**DNA barcodes:** ITS OQ870868, BenA OR051217, CaM OR051392, RPB2 OR062082.

**Colony diam.**, 7 days, 25 °C (unless stated otherwise): CYA 35–37 mm; CYA 37 °C no growth; CYA 5 °C no growth; MEA 32–34 mm; YES 41–42 mm; PDA 30–31 mm.

**Colony characteristics:** On CYA 25 °C, 7 days: Colonies nearly circular, radially sulcate; margins moderately wide, entire or undulated; mycelia white and yellow; texture velutinous; sporulation dense; conidia *en masse* dull green; soluble pigments absent; exudates orange or hyaline, clear; reverse light orange.

On MEA 25 °C, 7 days: Colonies nearly circular, protuberant at centers; margins narrow, entire; mycelia white; texture velutinous; sporulation dense; conidia *en masse* greyish green; soluble pigments absent; exudates absent; reverse orange, with a few red patches at centers.

On YES 25 °C, 7 days: Colonies nearly circular, radially and concentrically sulcate, concave at centers; margins moderately wide, undulated; mycelia white; texture velutinous; sporulation dense; conidia *en masse* dull green; soluble pigments absent; exudates absent; reverse cream to buff, with radiate branches.

On PDA 25 °C, 7 days: Colonies nearly circular, slightly protuberant at centers; margins moderately wide, entire; mycelia orange; texture velutinous; sporulation moderately dense; conidia *en masse* greyish green; soluble pigments absent; exudates absent; reverse vivid orange red.

**Micromorphology:** Conidiophores monoverticillate; stipes smooth- to finely rough-walled, 50–275 × 2.5–3.5 μm; phialides ampulliform to acerose, tapering into very thin neck, 5–10 per stipe, 7.5–11.5 × 2.5–3.5 μm; conidia subglobose to ellipsoidal, smooth-walled, 2.5–3.0 × 2.0–2.5 μm.

**Additional strains examined:** China. Chongqing City, Wushan County, Shuanglong Town, Huazhu Village, 31°9′48″ N 109°47′7″ E, in soil, 29 October 2020, Xin-Cun Wang, Huan-Di Zheng and Chang Liu, culture, Zhi-Kang Zhang, CS18-01; *ibid.*, CS18-15.

**Notes:** This species is close to *P. jacksonii* in the phylogenetic tree (Figure 7). It differs from the latter in 29 bp for BenA and 24 bp for CaM. Morphologically, it differs in faster growth rate on YES at 25 °C (41–42 vs. 30–32 mm) and longer stipes (50–275 vs. 80–135 μm) [64].

**Figure 16:**
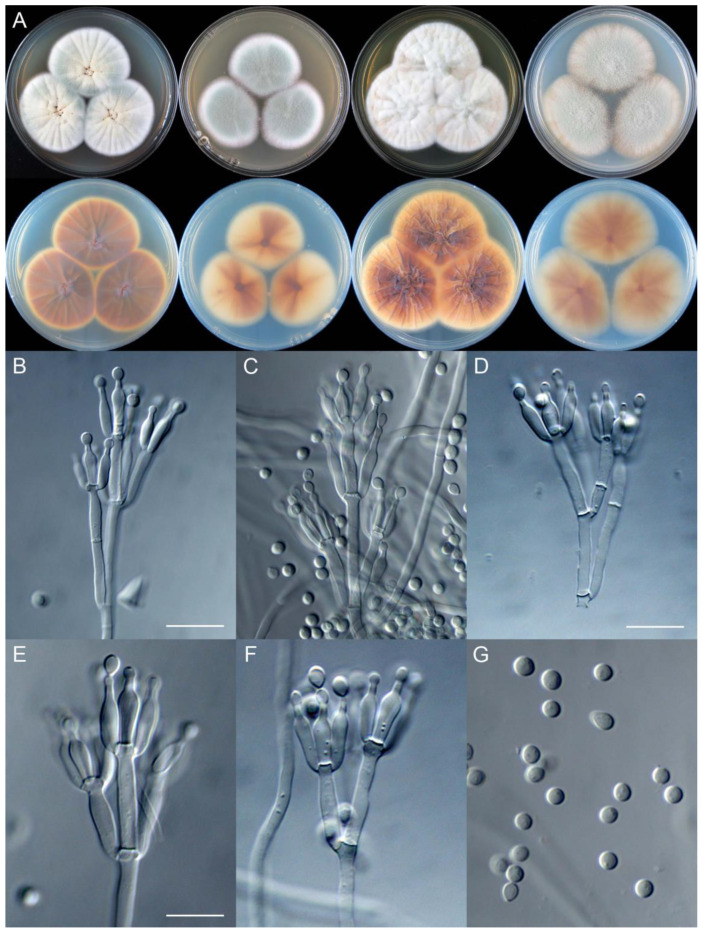
*Penicillium coffeatum* (CS10-15). (**A**) Colonies: top row left to right, obverse CYA, MEA, YES, and PDA; bottom row left to right, reverse CYA, MEA, YES, and PDA; (**B**–**F**) Conidiophores; (**G**) Conidia. Bars: (**B**) = 15 µm, also for (**C**); (**D**) = 12.5 µm; (**E**) = 10 µm, also for (**F**,**G**).

***Penicillium coffeatum*** X.C. Wang & W.Y. Zhuang, sp. nov. Figure 16.

**Fungal Names:** FN571540.

**Etymology:** The specific epithet refers to the coffeecolor on CYA reverse.

In *Penicillium* subgenus *Aspergilloides* section *Lanata-Divaricata* series *Rolfsiorum*.

**Typification:** China. Chongqing City, Nanchuan District, Jinfo Mountain National Nature Reserve, North mountain slope, 29°5′35″ N 107°14′47″ E, in soil, 25 October 2020, Chang Liu, Zhao-Qing Zeng, Xin-Cun Wang and Huan-Di Zheng, culture, Zhi-Kang Zhang, CS10-15 (holotype HMAS 247891, ex-type strain CGMCC 3.25152).

**DNA barcodes:** ITS OQ870815, BenA OR051121, CaM OR051298, RPB2 OR051466.

**Colony diam.**, 7 days, 25 °C (unless stated otherwise): CYA 44–47 mm; CYA 37 °C no growth; CYA 5 °C no growth; MEA 41–42 mm; YES 52–53 mm; PDA 49–51 mm.

**Colony characteristics:** On CYA 25 °C, 7 days: Colonies nearly circular, concave at centers, radially sulcate; margins narrow, entire; mycelia white; texture velutinous; sporulation sparse to moderately dense; conidia *en masse* greyish green; soluble pigments absent; exudates hyaline to brown or absent; reverse coffee color.

On MEA 25 °C, 7 days: Colonies nearly circular, plain, slightly protuberant at centers or not, with light-colored radiate branches; margins moderately wide, entire; mycelia white; texture velutinous; sporulation dense; conidia *en masse* greyish green; soluble pigments absent; exudates absent; reverse buff to brownish, with light brown sectors or radiate branches.

On YES 25 °C, 7 days: Colonies nearly circular, strongly sulcate, protuberant at centers; margins wide, fimbriate; mycelia white; texture velutinous; sporulation sparse; conidia *en masse* greenish grey; soluble pigments absent; exudates absent; reverse buff to pale brown, with brown radiations.

On PDA 25 °C, 7 days: Colonies nearly circular, protuberant at centers; margins moderately wide, entire; mycelia white; texture velutinous; sporulation dense; conidia *en masse* greyish green; soluble pigments absent; exudates absent; reverse pale brownish, with light brown radiate branches.

**Micromorphology:** Conidiophores terverticillate or biverticillate, occasionally quaterverticillate; stipes smooth-walled, 50–225 × 2.0–3.0 μm; rami 2–3, 14–32.5 × 2.5–3.0 μm; metulae 1–3, 15–22 × 2.0–4.0 μm; phialides ampulliform to acerose, tapering into very thin neck, 4–5 per metula, 10–16 × 3.0–4.0 μm; conidia subglobose to ellipsoidal, smooth-walled, 3.5–5.0 × 3.0–4.0 μm.

**Notes:** This species forms a distinct lineage in ser. *Rolfsiorum* (Figure 6). It seems to have close relationship with *P. hainanense* and *P. vasconiae*, but differs from them in its terverticillate conidiophores [43,65].

**Figure 17:**
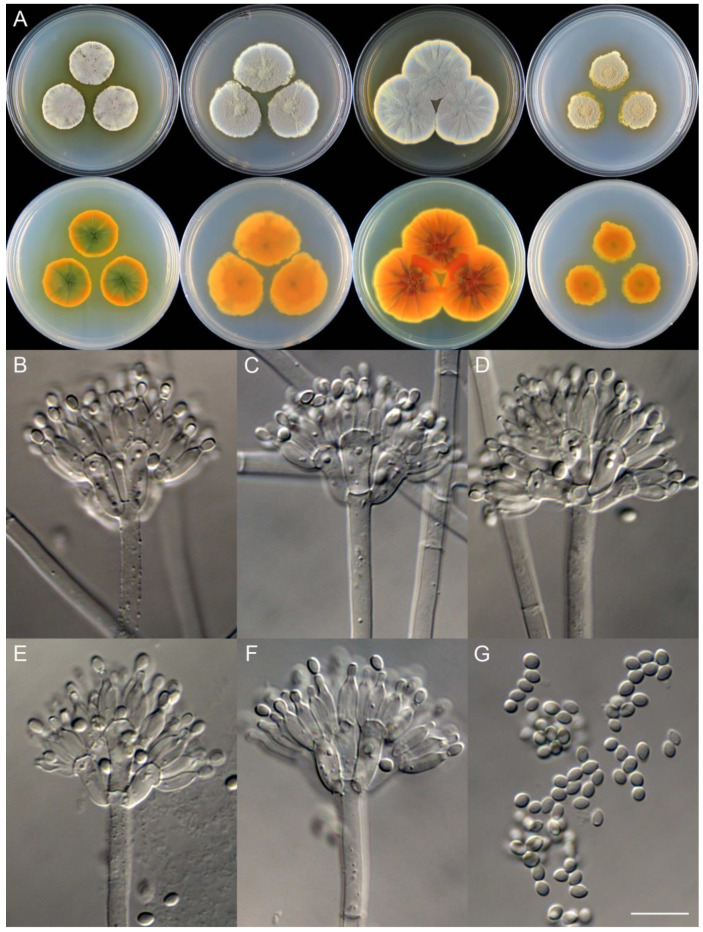
*Penicillium creberum* (CS02-09). (**A**) Colonies: top row left to right, obverse CYA, MEA, YES, and PDA; bottom row left to right, reverse CYA, MEA, YES, and PDA; (**B**–**F**) Conidiophores; (**G**) Conidia. Bar: (**G**) = 10 µm, also for (**B**–**F**).

***Penicillium creberum*** X.C. Wang & W.Y. Zhuang, sp. nov. Figure 17.

**Fungal Names:** FN571541.

**Etymology:** The specific epithet refers to the dense phialides of the fungus.

In *Penicillium* subgenus *Aspergilloides* section *Sclerotiorum* series *Herqueorum*.

**Typification:** China. Chongqing City, Beibei District, Jinyun Mountain National Nature Reserve, 29°50′18″ N 106°23′45″ E, in soil, 23 October 2020, Chang Liu, Zhao-Qing Zeng, Xin-Cun Wang and Huan-Di Zheng, culture, Zhi-Kang Zhang, CS02-09 (holotype HMAS 247892, ex-type strain CGMCC 3.25153).

**DNA barcodes:** ITS OQ870833, BenA OR051182, CaM OR051357, RPB2 OR062048.

**Colony diam.**, 7 days, 25 °C (unless stated otherwise): CYA 26–27 mm; CYA 37 °C no growth; CYA 5 °C no growth; MEA 34–36 mm; YES 41–42 mm; PDA 23–24 mm.

**Colony characteristics:** On CYA 25 °C, 7 days: Colonies nearly circular, protuberant; margins narrow, entire; mycelia yellow; texture velutinous; sporulation dense; conidia *en masse* greyish green; soluble pigments yellow; exudates yellow, clear; reverse orange at margins, with olive to dark green radiate branches.

On MEA 25 °C, 7 days: Colonies irregular, protuberant at centers; margins narrow, irregular; mycelia yellow; texture velutinous; sporulation dense; conidia *en masse* yellowish green; soluble pigments yellow; exudates absent; reverse yellow brown to orange brown.

On YES 25 °C, 7 days: Colonies nearly circular or irregular, radially sulcate; margins narrow, undulated; mycelia yellow; texture velutinous; sporulation dense; conidia *en masse* yellowish green; soluble pigments yellow; exudates absent; reverse yellow to orange with brown radiate branches.

On PDA 25 °C, 7 days: Colonies irregular, protuberant at centers; margins narrow, irregular; mycelia yellow; texture velutinous; sporulation dense; conidia *en masse* yellowish green; soluble pigments yellow; exudates absent; reverse yellow to orange.

**Micromorphology:** Conidiophores biverticillate; stipes smooth to rough-walled, 350–515 × 3.5–4.0 μm; metulae 5, 10–13.5 × 4.0–5.5 μm; phialides ampulliform, tapering into very thin neck, 6–8 per metula, 7.5–9 × 2.5–3.5 μm; conidia ellipsoidal to broad fusiform, smooth-walled, 3.0–3.5 × 2.5–3.0 μm.

**Additional strain examined:** China. Chongqing City, Fengjie County, Caotang Town, 31°5′29″ N 109°38′57″ E, in soil, 29 October 2020, Xin-Cun Wang, Huan-Di Zheng and Chang Liu, culture, Zhi-Kang Zhang, CS16-08.

**Notes:** This species is a sister of *P. flosculum* with strong support (BP = 100, PP = 1.00, Figure 7). It differs from the latter in nine bp for BenA, 13 bp for CaM and 19 bp for RPB2. Morphologically, it differs in slower growth rate on PDA at 25 °C (23–24 vs. 29–31 mm) and somewhat shorter phialides (7.5–9.0 vs. 8.5–12 μm) and conidia (3.0–3.5 vs. 3.5–4.5 μm). But it shows no morphological differences with the traditional concept of *P. herquei*.

**Figure 18:**
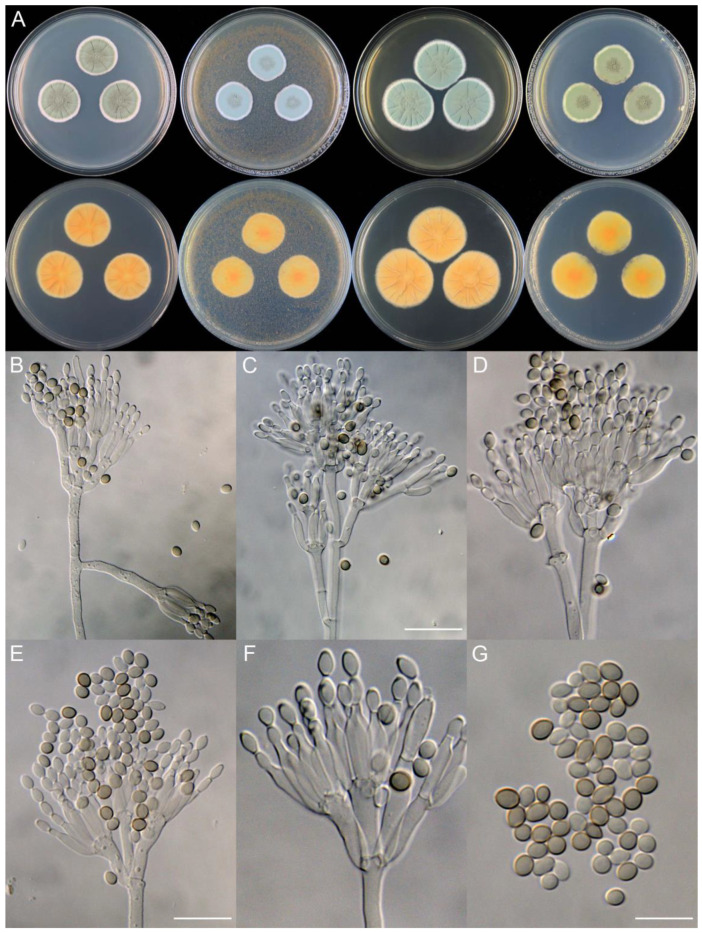
*Penicillium dabashanicum* (CS26-07). (**A**) Colonies: top row left to right, obverse CYA, MEA, YES, and PDA; bottom row left to right, reverse CYA, MEA, YES, and PDA; (**B**–**F**) Conidiophores; (**G**) Conidia. Bars: (**C**) = 20 µm, also for (**B**); (**E**) = 15 µm, also for (**D**); (**G**) = 10 µm, also for (**F**).

***Penicillium dabashanicum*** X.C. Wang & W.Y. Zhuang, sp. nov. Figure 18.

**Fungal Names:** FN571542.

**Etymology:** The specific epithet refers to the type locality.

In *Penicillium* subgenus *Penicillium* section *Fasciculata* series *Camembertiorum*.

**Typification:** China. Chongqing City, Chengkou County, Daba Mountain National Nature Reserve, Gaoguan Town, at the riverside of Ren River, 31°49′40″ N 109°0′24″ E, in soil under a palm tree, 30 October 2020, Xin-Cun Wang, Huan-Di Zheng and Chang Liu, culture, Zhi-Kang Zhang, CS26-07 (holotype HMAS 247893, ex-type strain CGMCC 3.25154).

**DNA barcodes:** ITS OQ870786, BenA OR051047, CaM OR051226, RPB2 OR051400.

**Colony diam.**, 7 days, 25 °C (unless stated otherwise): CYA 24–26 mm; CYA 37 °C no growth; CYA 5 °C no growth; MEA 22–23 mm; YES 31–32 mm; PDA 23–25 mm.

**Colony characteristics:** On CYA 25 °C, 7 days: Colonies nearly circular, radially sulcate, slightly protuberant at centers; margins moderately wide, entire; mycelia white; texture velutinous; sporulation dense; conidia *en masse* viridian green; soluble pigments absent; exudates tiny, clear; reverse buff to yellow.

On MEA 25 °C, 7 days: Colonies nearly circular or irregular, plain, slightly protuberant at centers; margins moderately wide, entire, protuberant; mycelia white; texture velutinous; sporulation dense; conidia *en masse* viridian green; soluble pigments absent; exudates tiny, clear; reverse yellow, pale orange at centers.

On YES 25 °C, 7 days: Colonies nearly circular, radially sulcate, slightly protuberant and with funiculose hyphae at centers; margins moderately wide, entire; mycelia white; texture velutinous; sporulation dense; conidia *en masse* viridian green; soluble pigments absent; exudates absent; reverse buff to yellow.

On PDA 25 °C, 7 days: Colonies nearly circular or irregular, plain, protuberant at centers; margins moderately wide, entire or irregular, protuberant; mycelia white; texture velutinous; sporulation dense; conidia *en masse* viridian green; soluble pigments absent; exudates clear, hyaline, present at the centers; reverse yellowish, orange at centers.

**Micromorphology:** Conidiophores terverticillate to quaterverticillate; stipes smooth-walled to rough-walled, 100–285 × 3.0–3.5 μm; branches 2, 21–28 × 3.0–4.0 μm; rami 2, 14–30 × 3.0–5.0 μm; metulae 3–5, 14–24 × 3.0–4.0 μm; phialides 4–6, ampulliform to acerose, tapering into thin neck, 12–15 × 3.0–4.0 μm; conidia ellipsoidal, smooth-walled, 3.5–5.5 (–7.5) × 3.0–4.5 μm.

**Notes:** This new species is a sister of *P. crustosum* in the phylogenetic tree with strong supports (BP = 100, PP = 1.00, Figure 1). It differs the latter in 14 bp for BenA, six bp for CaM and 19 bp for RPB2. Morphologically, it differs in slower growth rates on CYA 25 °C (24–26 vs. 35–40 mm) and MEA (22–23 vs. 25–40 mm), shorter stipes (100–285 vs. 200–400), longer metulae (14–24 vs. 10–15 μm) and phialides (12–15 vs. 9–11 μm), and larger conidia (3.5–5.5 × 3.0–4.5 vs. 3.5–4.0 × 2.8–3.2 μm) [62].

**Figure 19:**
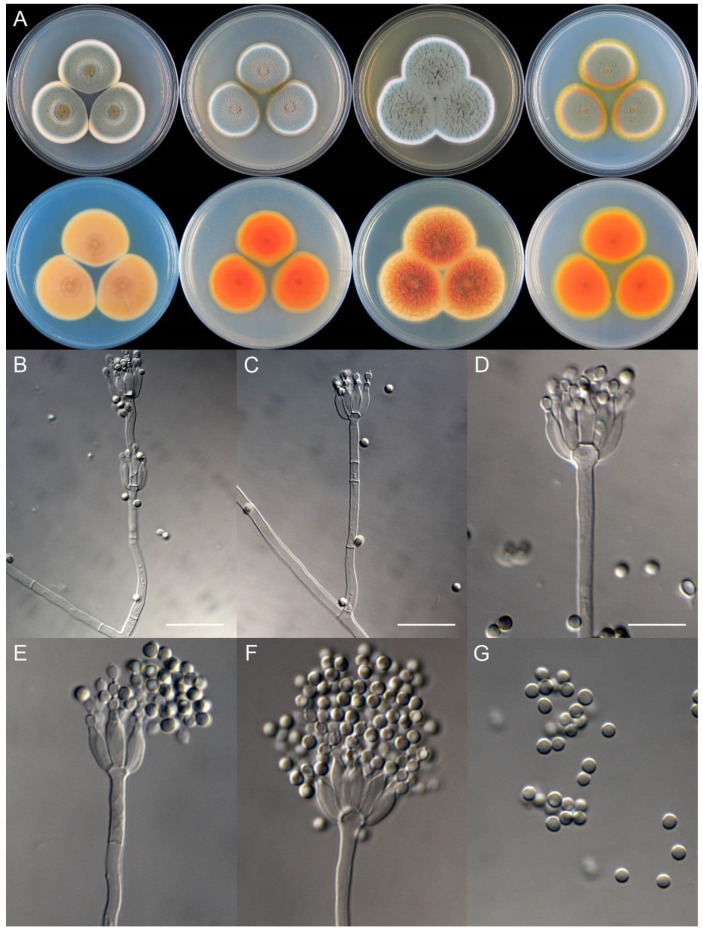
*Penicillium dazhouense* (CS33-19). (**A**) Colonies: top row left to right, obverse CYA, MEA, YES, and PDA; bottom row left to right, reverse CYA, MEA, YES, and PDA; (**B**–**F**) Conidiophores; (**G**) Conidia. Bars: (**B**) = 20 µm; (**C**) = 17.5 µm; (**D**) = 10 µm, also for (**E**–**G**).

***Penicillium dazhouense*** X.C. Wang & W.Y. Zhuang, sp. nov. Figure 19.

**Fungal Names:** FN571543.

**Etymology:** The specific epithet refers to the type locality.

In *Penicillium* subgenus *Aspergilloides* section *Sclerotiorum* series *Sclerotiorum*.

**Typification:** China. Sichuan Province, Dazhou City, Wanyuan City, Longtanhe, 31°50′19″ N 108°19′15″ E, in soil, 1 November 2020, Xin-Cun Wang, Huan-Di Zheng and Chang Liu, culture, Zhi-Kang Zhang, CS33-19 (holotype HMAS 247894, ex-type strain CGMCC 3.25155).

**DNA barcodes:** ITS OQ870871, BenA OR051220, CaM OR051394, RPB2 n.a.

**Colony diam.**, 7 days, 25 °C (unless stated otherwise): CYA 33–36 mm; CYA 37 °C no growth; CYA 5 °C no growth; MEA 31–33 mm; YES 40–42 mm; PDA 34–36 mm.

**Colony characteristics:** On CYA 25 °C, 7 days: Colonies nearly circular, concentrically sulcate at centers; margins moderately wide, entire; mycelia white; texture velutinous; sporulation dense; conidia *en masse* dull green; soluble pigments absent; exudates yellow, clear; reverse yellowish buff.

On MEA 25 °C, 7 days: Colonies nearly circular, protuberant at centers; margins narrow to moderately wide, entire; mycelia white; texture velutinous; sporulation dense; conidia *en masse* dull green; soluble pigments absent; exudates absent; reverse reddish orange.

On YES 25 °C, 7 days: Colonies nearly circular, strongly sulcate, concave at centers; margins narrow, undulated and fimbriate; mycelia white; texture velutinous; sporulation dense; conidia *en masse* dull green; soluble pigments absent; exudates yellow, clear; reverse yellowish buff to reddish brown.

On PDA 25 °C, 7 days: Colonies nearly circular, protuberant at centers; margins moderately wide, entire; mycelia orange; texture velutinous; sporulation dense; conidia *en masse* dull green; soluble pigments absent; exudates absent; reverse orange.

**Micromorphology:** Conidiophores monoverticillate; stipes smooth-walled, 50–140 × 2.5–3.0 μm; phialides ampulliform to acerose, tapering into very thin neck, 6–10 per metula, 7.5–10 × 3.0–3.5 μm; conidia subglobose, smooth-walled, 2.5–3.0 μm.

**Notes:** This species is a sister of *P. guanacastense* wit strong support (BP = 100, PP = 1.00, Figure 7). It differs from the latter in eight bp for BenA and 14 bp for CaM. Morphologically, it differs in faster growth rate on CYA at 25 °C (33–36 vs. 25–33 mm), orange color on MEA reverse and smooth-walled conidia [66].

**Figure 20:**
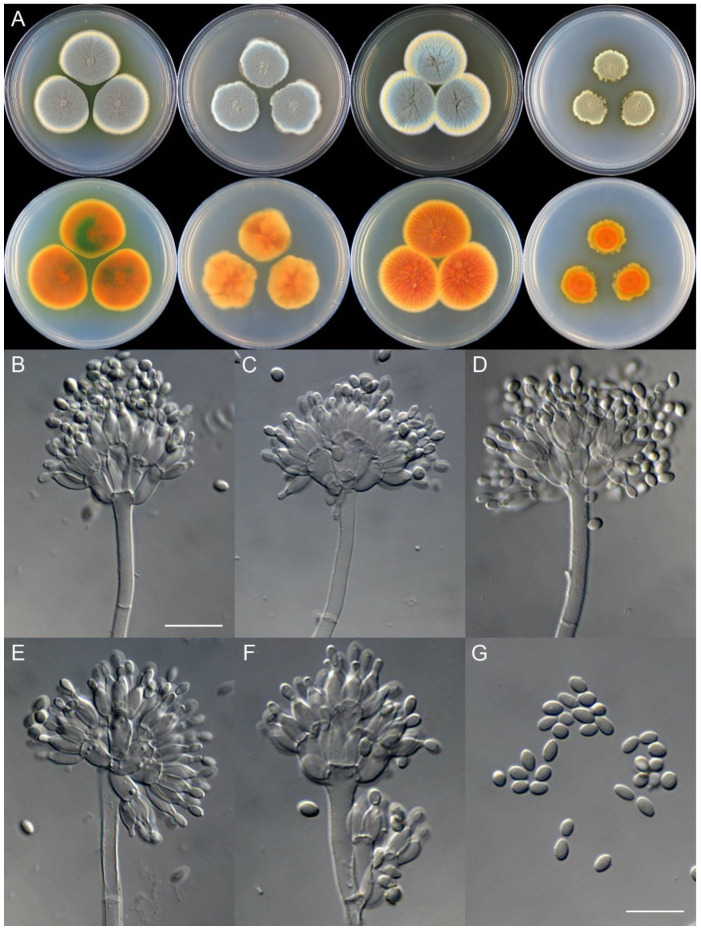
*Penicillium ellipsoideum* (CS20-01). (**A**) Colonies: top row left to right, obverse CYA, MEA, YES, and PDA; bottom row left to right, reverse CYA, MEA, YES, and PDA; (**B**–**F**) Conidiophores; (**G**) Conidia. Bars: (**B**) = 12.5 µm, also for (**C**–**E**); (**G**) = 10 µm, also for (**F**).

***Penicillium ellipsoideum*** X.C. Wang & W.Y. Zhuang, sp. nov. Figure 20.

**Fungal Names:** FN571544.

**Etymology:** The specific epithet refers to the ellipsoidal conidia.

In *Penicillium* subgenus *Aspergilloides* section *Sclerotiorum* series *Herqueorum*.

**Typification:** China. Chongqing City, Wushan County, Wulipo National Nature Reserve, 31°22′59″ N 109°56′11″ E, in soil, 29 October 2020, Xin-Cun Wang, Huan-Di Zheng and Chang Liu, culture, Zhi-Kang Zhang, CS20-01 (holotype HMAS 247895, ex-type strain CGMCC 3.25156).

**DNA barcodes:** ITS OQ870835, BenA OR051184, CaM OR051359, RPB2 OR062050.

**Colony diam.**, 7 days, 25 °C (unless stated otherwise): CYA 34–35 mm; CYA 37 °C no growth; CYA 5 °C no growth; MEA 28–32 mm; YES 35–37 mm; PDA 20–21 mm.

**Colony characteristics:** On CYA 25 °C, 7 days: Colonies nearly circular, protuberant at centers, slightly sulcate; margins narrow to moderately wide, entire; mycelia yellow; texture velutinous; sporulation dense; conidia *en masse* dull green; soluble pigments yellow; exudates absent; reverse orange, with dark green patches, yellow at margins.

On MEA 25 °C, 7 days: Colonies nearly circular or irregular, protuberant at centers; margins narrow to moderately wide, entire or irregular; mycelia yellow; texture velutinous; sporulation dense; conidia *en masse* yellowish green; soluble pigments absent; exudates absent; reverse yellow brown, with light brownish patches.

On YES 25 °C, 7 days: Colonies nearly circular, radially sulcate, concave at centers; margins moderately wide, undulated; mycelia yellow; texture velutinous; sporulation dense; conidia *en masse* dull green; soluble pigments absent; exudates absent; reverse orange brown, yellow at margins.

On PDA 25 °C, 7 days: Colonies nearly circular or irregular, slightly protuberant at centers; margins narrow, entire or irregular; mycelia yellow; texture velutinous; sporulation dense; conidia *en masse* dull green; soluble pigments light brown; exudates absent; reverse orange with margin paler.

**Micromorphology:** Conidiophores biverticillate, occasionally terverticillate; stipes smooth- to finely rough-walled, 135–315 × 3.0–4.0 μm; metulae 5–8, 8–13.5 × 3.5–6.5 μm; phialides ampulliform, tapering into very thin neck, 5–9 per metula, 6.5–10 × 3.0–3.5 μm; conidia ellipsoidal to broad fusiform, smooth-walled, 3.0–4.5 × 2.0–3.0 μm.

**Additional strains examined:** China. Chongqing City, Wushan County, Wulipo National Nature Reserve, 31°22′59″ N 109°56′11″ E, in soil, 29 October 2020, Xin-Cun Wang, Huan-Di Zheng and Chang Liu, culture, Zhi-Kang Zhang, CS20-11; *ibid.*, Chengkou County, Daba Mountain National Nature Reserve, Beiping Town, 31°58′17″ N 108°47′5″ E, in soil, 31 October 2020, Xin-Cun Wang, Huan-Di Zheng and Chang Liu, culture, Zhi-Kang Zhang, CS28-04. Shaanxi Province, Ankang City, Langao County, 32°2′45″ N 108°50′51″ E, in soil, 31 October 2020, Xin-Cun Wang, Huan-Di Zheng and Chang Liu, culture, Zhi-Kang Zhang, CS29-01.

**Notes:** This species is closely related to *P. brachycaulis* (Figure 7). It differs from the latter in nine bp for BenA, 11 bp for CaM and nine bp for RPB2. Morphologically, it differs in faster growth rates on CYA (34–35 vs. 27–28 mm) and YES (35–37 vs. 30–31 mm) at 25 °C, slower growth rate on PDA (20–21 vs. 24–26 mm), green patches on reverse of CYA, shorter metulae (8–13.5 vs. 9–16 μm), and much narrow, ellipsoidal conidia. But it shows no morphological differences with the traditional concept of *P. herquei*.

**Figure 21:**
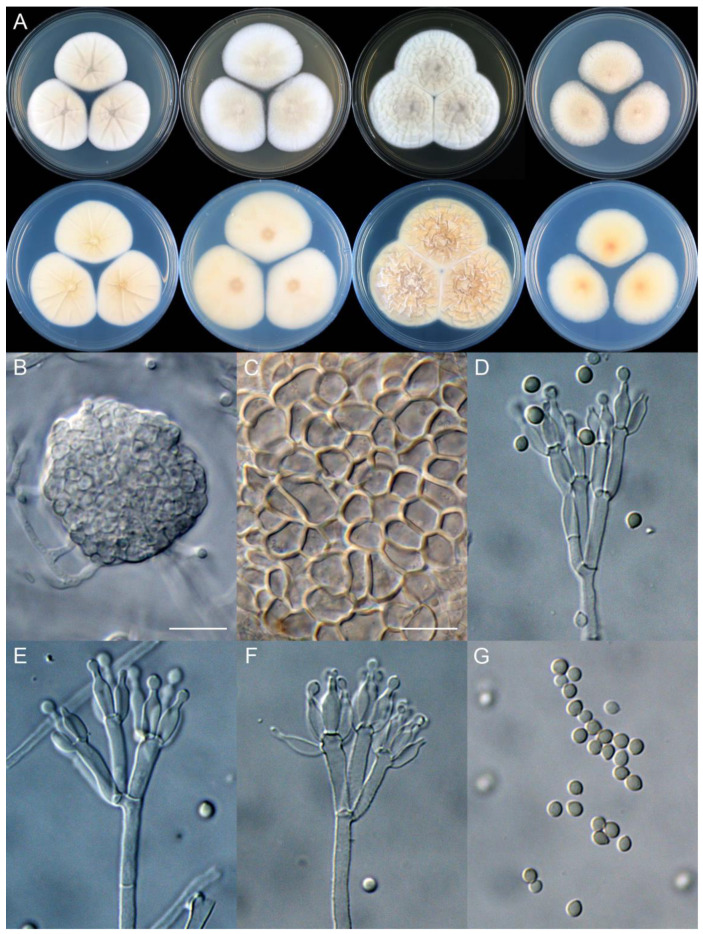
*Penicillium fengjieense* (CS15-01). (**A**) Colonies: top row left to right, obverse CYA, MEA, YES, and PDA; bottom row left to right, reverse CYA, MEA, YES, and PDA; (**B**) Young sclerotium; (**C**) Surface of mature sclerotium; (**D**–**F**) Conidiophores; (**G**) Conidia. Bars: (**B**) = 10 µm, also for (**D**–**G**); (**C**) = 15 µm.

***Penicillium fengjieense*** X.C. Wang & W.Y. Zhuang, sp. nov. Figure 21.

**Fungal Names:** FN571545.

**Etymology:** The specific epithet refers to the type locality.

In *Penicillium* subgenus *Aspergilloides* section *Lanata-Divaricata* series *Simplicissima*.

**Typification:** China. Chongqing City, Fengjie County, Caotang Town, 31°5′29″ N 109°38′57″ E, in soil, 29 October 2020, Xin-Cun Wang, Huan-Di Zheng and Chang Liu, culture, Zhi-Kang Zhang, CS15-01 (holotype HMAS 247896, ex-type strain CGMCC 3.25157).

**DNA barcodes:** ITS OQ870765, BenA OR051156, CaM OR051333, RPB2 OR051489.

**Colony diam.**, 7 days, 25 °C (unless stated otherwise): CYA 40–41 mm; CYA 37 °C no growth; CYA 5 °C no growth; MEA 44–46 mm; YES 46–48 mm; PDA 37–39 mm.

**Colony characteristics:** On CYA 25 °C, 7 days: Colonies nearly circular, slightly concave at centers, radially sulcate, orange brown at central areas; margins moderately wide, entire; mycelia white; texture velutinous; sclerotia abundant, white to light yellow; sporulation sparse; conidia *en masse* light grey; soluble pigments absent; exudates hyaline, clear; reverse buff.

On MEA 25 °C, 7 days: Colonies nearly circular, protuberant at centers; margins wide, entire; mycelia white; texture velutinous; sclerotia abundant, white to light yellow; sporulation sparse; conidia *en masse* light grey; soluble pigments absent; exudates absent; reverse white, light brown at centers.

On YES 25 °C, 7 days: Colonies nearly circular, strongly sulcate, concave at centers; margins wide, fimbriate; mycelia white; texture velutinous; sclerotia abundant, white to light yellow; sporulation sparse; conidia *en masse* light grey; soluble pigments absent; exudates absent; reverse yellow brown.

On PDA 25 °C, 7 days: Colonies nearly circular, plain, slightly protuberant at centers; margins wide, entire; mycelia white; texture velutinous; sclerotia abundant, white to light yellow; sporulation sparse; conidia *en masse* light grey; soluble pigments absent; exudates absent; reverse white to yellow, light brown at centers.

**Micromorphology:** Conidiophores biverticillate to terverticillate; stipes rough- or smooth-walled, 200–325 × 2.2–3.0 μm; rami 2–3, 11–14 × 2.5–3.5 μm; metulae 2–4, 9.0–17 × 2.0–4.0 μm; phialides ampulliform, tapering into very thin neck, 3–7 per metula, 7.0–9.0 × 2.0–3.0 μm; conidia subglobose to ellipsoidal, smooth-walled, 3.0–4.0 × 2.5–3.5 μm; sclerotia ellipsoidal or irregular, 30–120 × 28–115 μm.

**Notes:** This species is a distinct lineage in ser. *Simplicissima* (Figure 6). It produces sclerotia on different media, similar to that of *P. tanzanicum* on MEA. But it is distinguished from the latter in buff not orange CYA reverse, partly terverticillate conidiophores, shorter stipes (200–325 vs. 200–875 μm) and smooth-walled conidia [67].

**Figure 22:**
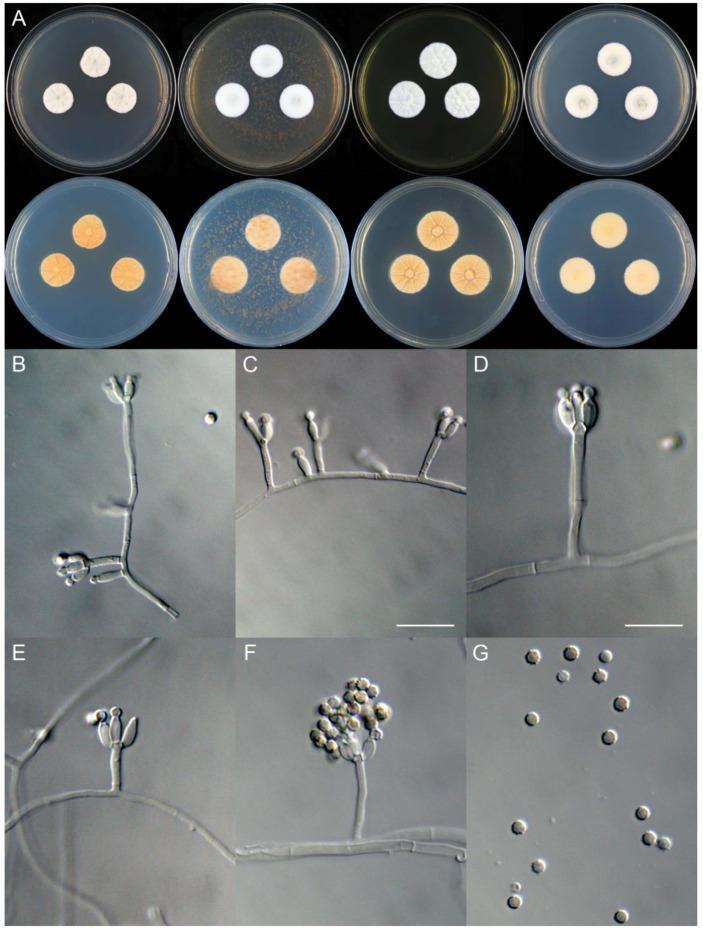
*Penicillium flemingii* (CS26-22). (**A**) Colonies: top row left to right, obverse CYA, MEA, YES, and PDA; bottom row left to right, reverse CYA, MEA, YES, and PDA; (**B**–**F**) Conidiophores; (**G**) Conidia. Bars: (**C**) = 12.5 µm, also for (**B**); (**D**) = 10 µm, also for (**E**–**G**).

***Penicillium flemingii*** X.C. Wang & W.Y. Zhuang, sp. nov. Figure 22.

**Fungal Names:** FN571546.

**Etymology:** The specific epithet is in memory of the late Scottish bacteriologist Alexander Fleming (1881.08–1955.03).

In *Penicillium* subgenus *Aspergilloides* section *Exilicaulis* series *Restricta*.

**Typification:** China. Chongqing City, Chengkou County, Daba Mountain National Nature Reserve, Gaoguan Town, at the riverside of Ren River, 31°49′40″ N 109°0′24″ E, in soil under a palm tree, 30 October 2020, Xin-Cun Wang, Huan-Di Zheng and Chang Liu, culture, Zhi-Kang Zhang, CS26-22 (holotype HMAS 247897, ex-type strain CGMCC 3.25158).

**DNA barcodes:** ITS OQ867293, BenA OR051093, CaM OR051270, RPB2 OR051441.

**Colony diam.**, 7 days, 25 °C (unless stated otherwise): CYA 17–18 mm; CYA 37 °C 13–15 mm; CYA 5 °C no growth; MEA 19–21 mm; YES 21–22 mm; PDA 19–20 mm.

**Colony characteristics:** On CYA 25 °C, 7 days: Colonies nearly circular or irregular, radially sulcate, concave at centers; margins narrow, entire; mycelia white; texture velutinous; sporulation absent; conidia *en masse* unknown; soluble pigments pink; exudates greenish yellow, clear; reverse pale orange buff.

On CYA 37 °C, 7 days: Colonies irregular, like the flower of *Chrysanthemum*, concave at centers, deep to the bottom and making the media ripped; margins narrow, irregular, protuberant; mycelia white; texture velutinous; sporulation absent; conidia *en masse* unknown; soluble pigments light yellow brown; exudates hyaline, clear; reverse pale pinkish.

On MEA 25 °C, 7 days: Colonies nearly circular, slightly protuberant at centers; margins narrow, entire or irregular; mycelia white; texture velutinous; sporulation absent; conidia *en masse* unknown; soluble pigments absent; exudates green to yellow; reverse white.

On YES 25 °C, 7 days: Colonies nearly circular, radially sulcate, concave at centers; margins narrow, undulated; mycelia white; texture velutinous; sporulation absent; conidia *en masse* unknown; soluble pigments absent; exudates yellow; reverse buff.

On PDA 25 °C, 7 days: Colonies nearly circular, protuberant at centers; margins narrow, entire or irregular; mycelia white; texture velutinous; sporulation sparse; conidia *en masse* light grey; soluble pigments absent; exudates hyaline, green to yellow; reverse pale buff to cream.

**Micromorphology:** Conidiophores monoverticillate; stipes smooth-walled, 7–24 × 1.5–2.0 μm; phialides ampulliform, tapering into very thin neck, 3–5 per stipe, 4.0–6.0 × 2.0–2.5 μm; conidia subglobose, rough-walled, 2.5–3.0 μm.

**Additional strains examined:** China. Chongqing City, Chengkou County, Daba Mountain National Nature Reserve, Gaoguan Town, at the riverside of Ren River, 31°49′40″ N 109°0′24″ E, in soil under a palm tree, 30 October 2020, Xin-Cun Wang, Huan-Di Zheng and Chang Liu, culture, Zhi-Kang Zhang, CS26-45; *ibid.*, CS26-59; *ibid.*, CS26-80; *ibid.*, CS26-88.

**Notes:** This species is a sister to *P. restrictum* with strong support (BP = 90, PP = 1.00, Figure 4). It differs the latter in 12 bp for BenA, 11 bp for CaM and 5 bp for RPB2. Morphologically, it differs in buff to pink colonial reverses on CYA and green to yellow exudates on MEA [62].

**Figure 23:**
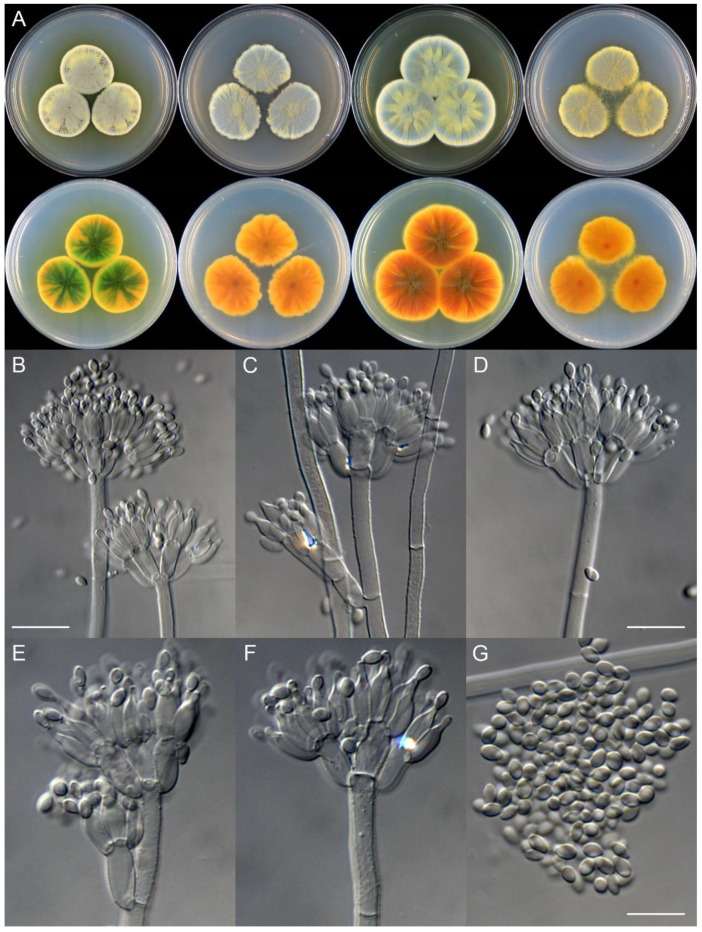
*Penicillium flosculum* (CS33-03). (**A**) Colonies: top row left to right, obverse CYA, MEA, YES, and PDA; bottom row left to right, reverse CYA, MEA, YES, and PDA; (**B**–**F**) Conidiophores; (**G**) Conidia. Bars: (**B**) = 15 µm; (**D**) = 12.5 µm, also for (**C**); (**G**) = 10 µm, also for (**E**,**F**).

***Penicillium flosculum*** X.C. Wang & W.Y. Zhuang, sp. nov. Figure 23.

**Fungal Names:** FN571547.

**Etymology:** The specific epithet refers to the flower-like colonies on YES.

In *Penicillium* subgenus *Aspergilloides* section *Sclerotiorum* series *Herqueorum*.

**Typification:** China. Sichuan Province, Dazhou City, Wanyuan City, Longtanhe, 31°50′19″ N 108°19′15″ E, in soil, 1 November 2020, Xin-Cun Wang, Huan-Di Zheng and Chang Liu, culture, Zhi-Kang Zhang, CS33-03 (holotype HMAS 247898, ex-type strain CGMCC 3.25159).

**DNA barcodes:** ITS OQ870839, BenA OR051188, CaM OR051363, RPB2 OR062053.

**Colony diam.**, 7 days, 25 °C (unless stated otherwise): CYA 29–31 mm; CYA 37 °C no growth; CYA 5 °C no growth; MEA 32–34 mm; YES 38–40 mm; PDA 29–31 mm.

**Colony characteristics:** On CYA 25 °C, 7 days: Colonies nearly circular, protuberant, slightly sulcate; margins narrow, entire; mycelia yellow; texture velutinous; sporulation dense; conidia *en masse* yellowish green; soluble pigments green; exudates yellow, clear; reverse light orange, with dark green sectors or radiate branches.

On MEA 25 °C, 7 days: Colonies nearly circular or irregular, protuberant at centers; margins narrow, entire or irregular; mycelia yellow; texture velutinous; sporulation dense; conidia *en masse* yellowish green; soluble pigments absent; exudates absent; reverse brownish orange, with light brown radiate branches.

On YES 25 °C, 7 days: Colonies nearly circular, radially sulcate; margins narrow, undulated; mycelia white and yellow; texture velutinous; sporulation dense; conidia *en masse* yellowish green; soluble pigments absent; exudates absent; reverse orange brown, with radiate branches.

On PDA 25 °C, 7 days: Colonies nearly circular or irregular, yellow hyphae present at centers and joint areas; margins narrow, entire or fimbriate; mycelia yellow; texture velutinous; sporulation dense; conidia *en masse* yellowish green; soluble pigments absent; exudates absent; reverse light orange, with light brown radiate branches.

**Micromorphology:** Conidiophores biverticillate or terverticillate; stipes finely rough-walled, 310–460 × 3.5–4.5 μm; rami 2, 22.5–30 × 4.0 μm; metulae 5–6, 9–13 × 4.0–6.0 μm; phialides ampulliform, tapering into very thin neck, 6–10 per metula, 8.5–12 × 3.0–4.5 μm; conidia ellipsoidal to broad fusiform, smooth-walled, 3.5–4.5 × 2.0–3.0 μm.

**Notes:** This species is a sister of *P. creberum* with strong support (BP = 100, PP = 1.00, Figure 7). It differs from the latter in nine bp for BenA, 13 bp for CaM and 19 bp for RPB2. Morphologically, it differs in faster growth rate on PDA at 25 °C (29–31 vs. 23–24 mm) and longer phialides (8.5–12 vs. 7.5–9.0 μm) and conidia (3.5–4.5 vs. 3.0–3.5μm). But it shows no morphological differences with the traditional concept of *P. herquei*.

**Figure 24:**
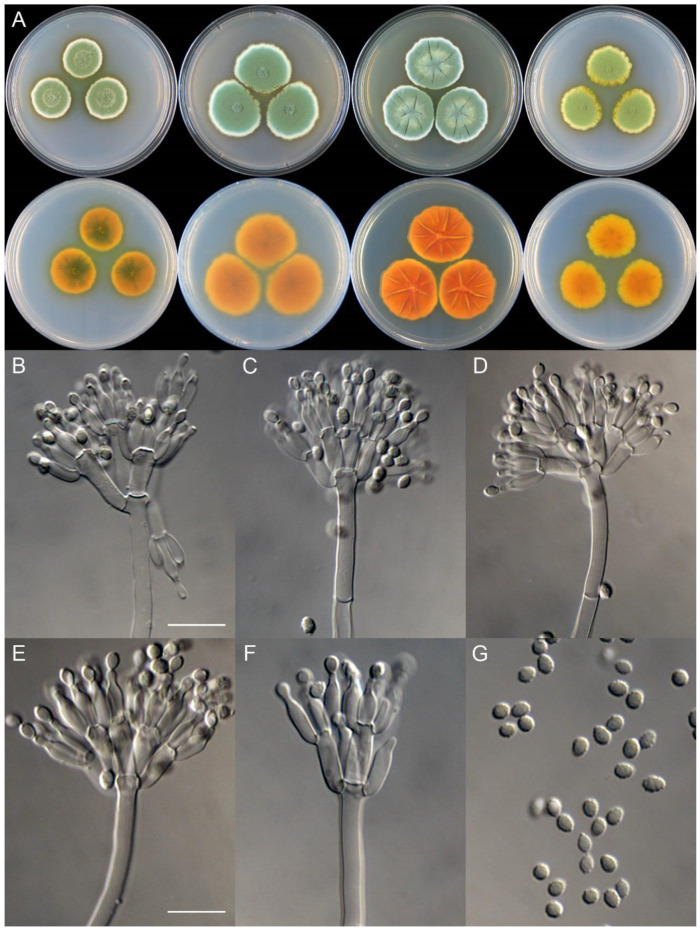
*Penicillium jiangjinense* (CS04-14). (**A**) Colonies: top row left to right, obverse CYA, MEA, YES, and PDA; bottom row left to right, reverse CYA, MEA, YES, and PDA; (**B**–**F**) Conidiophores; (**G**) Conidia. Bars: (**B**) = 12.5 µm, also for (**C**,**D**); (**E**) = 10 µm, also for (**F**,**G**).

***Penicillium jiangjinense*** X.C. Wang & W.Y. Zhuang, sp. nov. Figure 24.

**Fungal Names:** FN571548.

**Etymology:** The specific epithet refers to the type locality.

In *Penicillium* subgenus *Aspergilloides* section *Sclerotiorum* series *Herqueorum*.

**Typification:** China. Chongqing City, Jiangjin District, Simian Mountain Nature Reserve, 28°35′57″ N 106°26′51″ E, in soil of ant hole, 24 October 2020, Chang Liu, Zhao-Qing Zeng, Xin-Cun Wang and Huan-Di Zheng, culture, Zhi-Kang Zhang, CS04-14 (holotype HMAS 247899, ex-type strain CGMCC 3.25160).

**DNA barcodes:** ITS OQ870840, BenA OR051189, CaM OR051364, RPB2 OR062054.

**Colony diam.**, 7 days, 25 °C (unless stated otherwise): CYA 24–25 mm; CYA 37 °C no growth; CYA 5 °C no growth; MEA 34–35 mm; YES 33–35 mm; PDA 25–26 mm.

**Colony characteristics:** On CYA 25 °C, 7 days: Colonies nearly circular, protuberant at centers; margins narrow, entire; mycelia yellow; texture velutinous; sporulation dense; conidia *en masse* vivid green; soluble pigments yellow; exudates absent; reverse light orange, with greenish radiate branches.

On MEA 25 °C, 7 days: Colonies nearly circular, protuberant at centers; margins narrow, entire or irregular; mycelia yellow; texture velutinous; sporulation dense; conidia *en masse* yellowish green; soluble pigments absent; exudates absent; reverse yellow brown, with brownish radiate branches.

On YES 25 °C, 7 days: Colonies nearly circular, radially sulcate, concave at centers; margins narrow, undulated; mycelia yellow; texture velutinous; sporulation dense; conidia *en masse* yellowish green; soluble pigments yellow; exudates absent; reverse brownish orange with clear radiations.

On PDA 25 °C, 7 days: Colonies nearly circular, slightly protuberant at centers; margins narrow, entire; mycelia yellow; texture velutinous; sporulation dense; conidia *en masse* dull green; soluble pigments absent; exudates absent; reverse light brownish orange, with brownish patches at central areas.

**Micromorphology:** Conidiophores biverticillate; stipes smooth-walled, 210–300 × 3.0–4.5 μm; metulae 4–6, 10–16 × 3–5 μm; phialides ampulliform, tapering into very thin neck, 6–9 per metula, 8.5–12.5 × 2.5–4.0 μm; conidia ellipsoidal to broad fusiform, rough-walled, 3.5–4.5 × 2.5–3.0 μm.

**Notes:** This species is a sister of *P. neoherquei* with strong support (BP = 100, PP = 1.00, Figure 7). It differs from the latter in nine bp for BenA, 10 bp for CaM and one bp for RPB2. Morphologically, it differs in faster growth rates on CYA (24–25 vs. 18–22 mm), MEA (34–35 vs. 25–30 mm) and YES (33–35 vs. 27–30 mm), vivid green instead of dark dull green conidia *en masse* on CYA, with green branches on reverse of CYA, orange brown reverse of YES and longer phialides (8.5–12.5 vs. 8–10 μm) [68].

**Figure 25:**
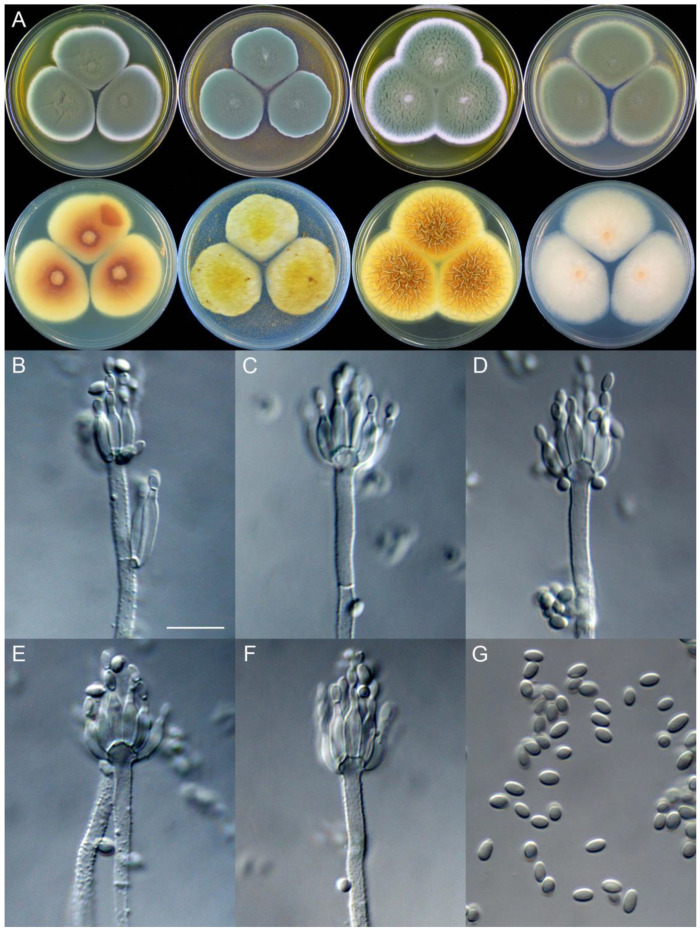
*Penicillium jinfoshanicum* (CS12-10). (**A**) Colonies: top row left to right, obverse CYA, MEA, YES, and PDA; bottom row left to right, reverse CYA, MEA, YES, and PDA; (**B**–**F**) Conidiophores; (**G**) Conidia. Bar: (**B**) = 10 µm, also for (**C**–**G**).

***Penicillium jinfoshanicum*** X.C. Wang & W.Y. Zhuang, sp. nov. Figure 25.

**Fungal Names:** FN571549.

**Etymology:** The specific epithet refers to the type locality.

In *Penicillium* subgenus *Aspergilloides* section *Aspergilloides* series *Thomiorum*.

**Typification:** China. Chongqing City, Nanchuan District, Jinfo Mountain National Nature Reserve, 29°1′30″ N 107°11′35″ E, in soil, 26 October 2020, Chang Liu, Zhao-Qing Zeng, Xin-Cun Wang and Huan-Di Zheng, culture, Zhi-Kang Zhang, CS12-10 (holotype HMAS 247900, ex-type strain CGMCC 3.25161).

**DNA barcodes:** ITS OQ870813, BenA OR051074, CaM OR051253, RPB2 OR051425.

**Colony diam.**, 7 days, 25 °C (unless stated otherwise): CYA 48–49 mm; CYA 37 °C no growth; CYA 5 °C no growth; MEA 39–41 mm; YES 54–55 mm; PDA 51–54 mm.

**Colony characteristics:** On CYA 25 °C, 7 days: Colonies nearly circular, sulcate and slightly protuberant at centers; margins narrow to moderately wide, entire; mycelia white; texture velutinous; sporulation dense; conidia *en masse* dull green; soluble pigments absent; exudates absent; reverse cream yellow at margins, light brown at centers.

On MEA 25 °C, 7 days: Colonies irregular, plain; margins narrow, irregular; mycelia white; texture velutinous to floccose; sporulation dense; conidia *en masse* dull green; soluble pigments absent; exudates absent; reverse cream.

On YES 25 °C, 7 days: Colonies nearly circular, strongly sulcate; margins moderately wide, fimbriate; mycelia white; texture velutinous, but floccose at centers; sporulation dense; conidia *en masse* dull green; soluble pigments absent; exudates absent; reverse buff to yellow at margins, brown with interwoven cracks at centers.

On PDA 25 °C, 7 days: Colonies nearly circular, plain, slightly protuberant at centers; margins moderately wide, entire; mycelia white; texture velutinous; sporulation dense; conidia *en masse* dull green; soluble pigments absent; exudates absent; reverse whitish with a pinkish tint, light pinkish orange at centers.

**Micromorphology:** Conidiophores monoverticillate, occasionally divaricate; stipes rough-walled, 60–160 × 2.5–4.0 μm; branch 18 × 3.0–3.5 μm; phialides acerose to ampulliform, tapering into very thin neck, 6–9, 8.5–13.5 × 2.5–3.5 μm; conidia narrow ellipsoidal, smooth-walled, 3.5–4.8 × 2.2–3.0 μm.

**Additional strain examined:** China. Chongqing City, Nanchuan District, Jinfo Mountain National Nature Reserve, 29°1′30″ N 107°11′35″ E, in soil, 26 October 2020, Chang Liu, Zhao-Qing Zeng, Xin-Cun Wang and Huan-Di Zheng, culture, Zhi-Kang Zhang, CS12-11.

**Notes:** This species is a sister of *P. aurantioviolaceum* with strong support (BP = 100, PP = 100, Figure 2). It differs from the latter in five bp for BenA, 17 bp for CaM and 21 bp for RPB2. Morphologically, these two species do not produce sclerotia; while *P. jinfoshanicum* differs from *P. aurantioviolaceum* in shorter stipes (60–160 vs. 200–400) and smooth conidia [62].

**Figure 26:**
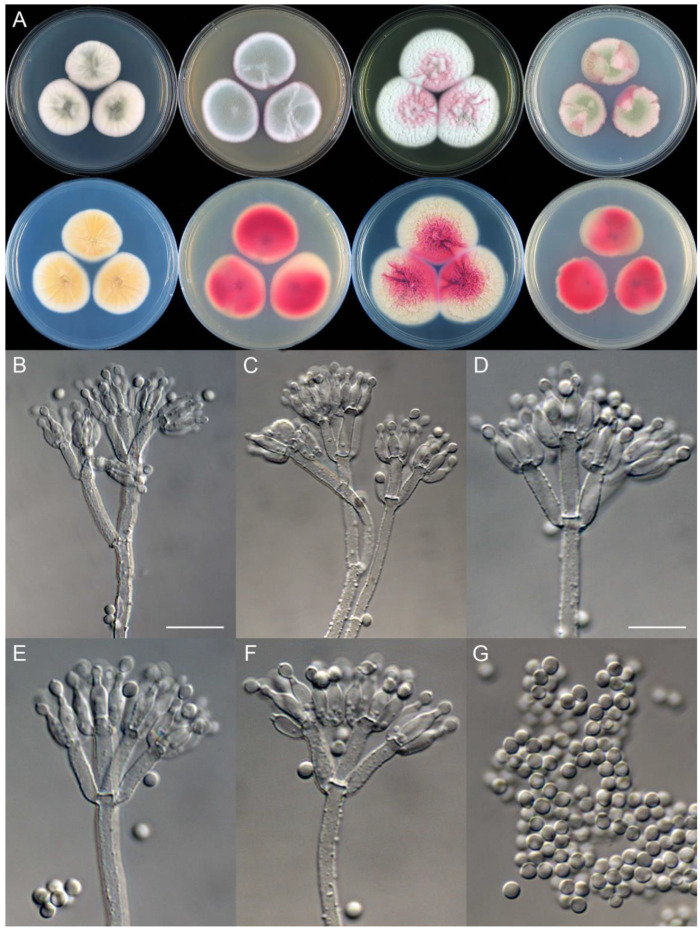
*Penicillium jinyunshanense* (CS02-01). (**A**) Colonies: top row left to right, obverse CYA, MEA, YES, and PDA; bottom row left to right, reverse CYA, MEA, YES, and PDA; (**B**–**F**) Conidiophores; (**G**) Conidia. Bars: (**B**) = 12.5 µm, also for (**C**); (**D**) = 10 µm, also for (**E**–**G**).

***Penicillium jinyunshanicum*** X.C. Wang & W.Y. Zhuang, sp. nov. Figure 26.

**Fungal Names:** FN571550.

**Etymology:** The specific epithet refers to the type locality.

In *Penicillium* subgenus *Aspergilloides* section *Lanata-Divaricata* series *Simplicissima*.

**Typification:** China. Chongqing City, Beibei District, Jinyun Mountain National Nature Reserve, 29°50′18″ N 106°23′45″ E, in soil, 23 October 2020, Chang Liu, Zhao-Qing Zeng, Xin-Cun Wang and Huan-Di Zheng, culture, Zhi-Kang Zhang, CS02-01 (holotype HMAS 247901, ex-type strain CGMCC 3.25162).

**DNA barcodes:** ITS OQ870766, BenA OR051157, CaM OR051334, RPB2 OR051490.

**Colony diam.**, 7 days, 25 °C (unless stated otherwise): CYA 32–34 mm; CYA 37 °C no growth; CYA 5 °C no growth; MEA 36–38 mm; YES 44–45 mm; PDA 29–33 mm.

**Colony characteristics:** On CYA 25 °C, 7 days: Colonies nearly circular, radially sulcate, usually with sectors; margins wide, entire; mycelia white; texture velutinous; sporulation sparse; conidia *en masse* greyish green; soluble pigments absent; exudates absent; reverse yellow, whitish at margins.

On MEA 25 °C, 7 days: Colonies nearly circular, slightly protuberant at centers, with light-colored sectors; margins moderately wide, entire; mycelia white; texture velutinous; sporulation dense; conidia *en masse* greyish green; soluble pigments absent; exudates absent; reverse rose color.

On YES 25 °C, 7 days: Colonies nearly circular, strongly sulcate, pink and protuberant at centers, with sectors; margins wide, fimbriate; mycelia white; texture velutinous; sporulation absent; soluble pigments absent; exudates absent; reverse buff, rose color at centers.

On PDA 25 °C, 7 days: Colonies nearly circular or irregular, plain, with sectors without sporulation; margins moderately wide, entire or irregular; mycelia white; texture velutinous; sporulation moderately dense; conidia *en masse* greyish green; soluble pigments absent; exudates absent; reverse rose color.

**Micromorphology:** Conidiophores biverticillate or terverticillate; stipes rough-walled, 160–375 × 2.0–3.0 μm; rami 2, 20–28 × 2.5–3.5 μm; metulae 3–5, 11–17.5 (–20) × 2.5–3.5 μm; phialides ampulliform, tapering into very thin neck, 5–8 per metula, 7.5–10 × 2.5–3.5 μm; conidia subglobose, smooth-walled, 2.5–3.5 μm.

**Additional strains examined:** China. Chongqing City, Beibei District, Jinyun Mountain National Nature Reserve, 29°50′18″ N 106°23′45″ E, in soil, 23 October 2020, Chang Liu, Zhao-Qing Zeng, Xin-Cun Wang and Huan-Di Zheng, culture, Zhi-Kang Zhang, CS02-02; *ibid.*, CS02-10; *ibid.*, CS03-06; *ibid.*, CS03-07.

**Notes:** This species is a sister to *P. laevigatum* and *P. wandoense* with strong support (BP = 99, PP = 100, Figure 6). *Penicillium laevigatum* was isolated from acidic soil of Hainan Province, China [43], and *P. wandoense* was from freshwater of South Korea [69]. Molecular divergences between them are limited (one bp for BenA, none for CaM and five bp for RPB2), and their morphological distinctions are obscure. Thus, they should represent the same species, and *P. laevigatum* has the priority. *Penicillium jinyunshanicum* differs from *P. laevigatum* in 16 bp for BenA, 16 bp for CaM and 18 bp for RPB2. Morphologically, its rose color on MEA, YES and PDA in reverse view at 25 °C distinguishes it from its sister taxon.

**Figure 27:**
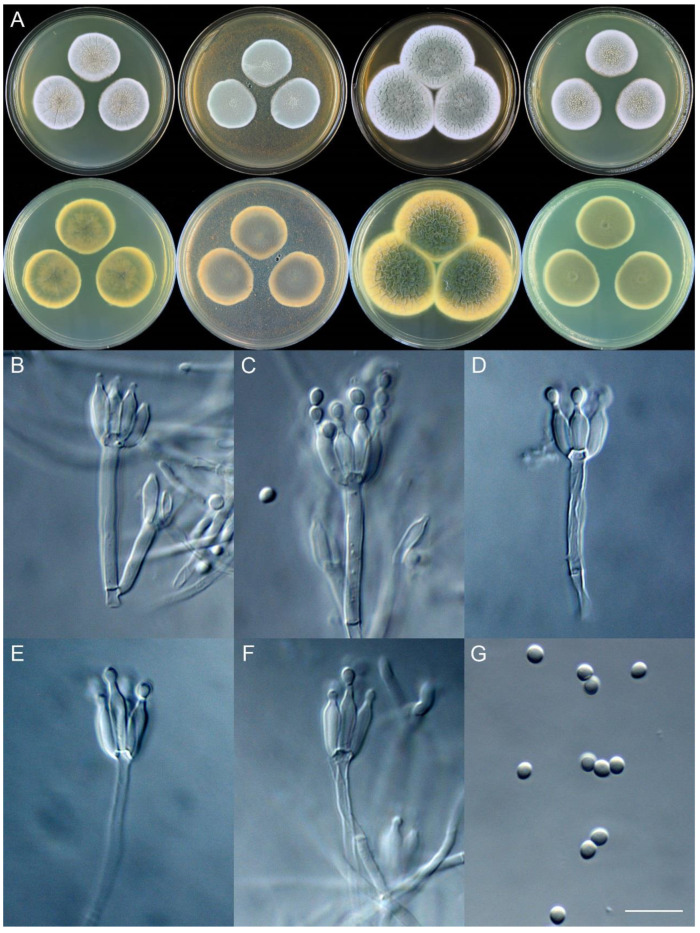
*Penicillium johnpittii* (CS23-04). (**A**) Colonies: top row left to right, obverse CYA, MEA, YES, and PDA; bottom row left to right, reverse CYA, MEA, YES, and PDA; (**B**–**F**) Conidiophores; (**G**) Conidia. Bar: (**G**) = 10 µm, also for (**B**–**F**).

***Penicillium johnpittii*** X.C. Wang & W.Y. Zhuang, sp. nov. Figure 27.

**Fungal Names:** FN571551.

**Etymology:** The specific epithet is in memory of the late distinguished mycologist John Ingram Pitt (1937.03–2022.03).

In *Penicillium* subgenus *Aspergilloides* section *Gracilenta* series *Macrosclerotiorum*.

**Typification:** China. Chongqing City, Wuxi County, Gulu Town, Changlong Village, 31°19′24″ N 109°26′39″ E, in soil, 30 October 2020, Xin-Cun Wang, Huan-Di Zheng and Chang Liu, culture, Zhi-Kang Zhang, CS23-04 (holotype HMAS 247902, ex-type strain CGMCC 3.25163).

**DNA barcodes:** ITS OQ870826, BenA OR051102, CaM OR051279, RPB2 OR051448.

**Colony diam.**, 7 days, 25 °C (unless stated otherwise): CYA 30–32 mm; CYA 37 °C no growth; CYA 5 °C no growth; MEA 29–38 mm; YES 47–49 mm; PDA 30–32 mm.

**Colony characteristics:** On CYA 25 °C, 7 days: Colonies nearly circular, radially sulcate, concave at centers; margins moderately wide, entire; mycelia white; texture velutinous; sporulation dense; conidia *en masse* greenish grey; soluble pigments yellow; exudates yellow, clear; sclerotia white to light yellow; reverse olivaceous, yellowish at margins.

On MEA 25 °C, 7 days: Colonies irregular, plain, slightly protuberant at centers; margins narrow to moderately wide, entire; mycelia white; texture velutinous; sporulation dense; conidia *en masse* greenish grey; soluble pigments absent; exudates absent; sclerotia white to light yellow; reverse grey, yellowish at margins.

On YES 25 °C, 7 days: Colonies nearly circular, deep, strongly sulcate; margins moderately wide, fimbriate; mycelia white; texture velutinous; sporulation dense; conidia *en masse* greenish grey; soluble pigments absent; exudates absent; sclerotia white to light yellow; reverse green to olivaceous, buff at margins.

On PDA 25 °C, 7 days: Colonies nearly circular, plain, slightly protuberant at centers; margins narrow to moderately wide, entire; mycelia white; texture velutinous; sporulation dense; conidia *en masse* greenish grey; soluble pigments absent; exudates hyaline, clear; sclerotia white to light yellow; reverse olivaceous and paler at margins.

**Micromorphology:** Conidiophores monoverticillate or divaricate; stipes smooth-walled, 50–100 × 2.0–3.0 μm; metulae 13.5–27 × 2.5–3.0 μm; phialides ampulliform to acerose, tapering into very thin neck, 5–6 per metula/stipe, 8–12 × 3.0–4.0 μm; conidia subglobose to broad ellipsoid, smooth-walled, 2.5–3.5 μm.

**Additional strain examined:** China. Sichuan Province, Dazhou City, Wanyuan City, Longtanhe, 31°50′19″ N 108°19′15″ E, in soil, 1 November 2020, Xin-Cun Wang, Huan-Di Zheng and Chang Liu, culture, Zhi-Kang Zhang, CS33-02.

**Notes:** This species is a sister to *P. macrosclerotiorum* with strong support (BP = 100, PP = 1.00, Figure 5), but differs from the latter in 13 bp for BenA, 21 bp for CaM and eight bp for RPB2. Morphologically, it grows much faster on MEA at 25 °C (29–38 vs. 15–17 mm), but slower on YES (47–49 vs. 54–56 mm). Greenish grey conidia *en masse* are produced by this species on CYA and YES, while *P. macrosclerotiorum* has pea green conidia *en masse* on the same media [34].

**Figure 28:**
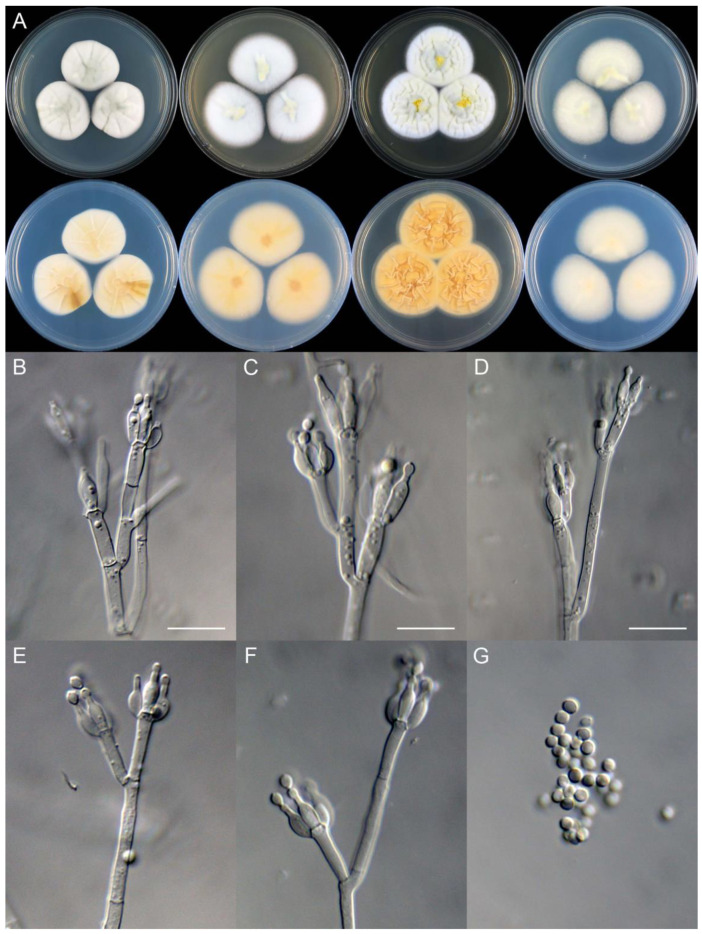
*Penicillium pauciramulum* (CS04-09). (**A**) Colonies: top row left to right, obverse CYA, MEA, YES, and PDA; bottom row left to right, reverse CYA, MEA, YES, and PDA; (**B**–**F**) Conidiophores; (**G**) Conidia. Bars: (**D**) = 15 µm; (**B**) = 12.5 µm; (**C**) = 10 µm, also for (**E**–**G**).

***Penicillium pauciramulum*** X.C. Wang & W.Y. Zhuang, sp. nov. Figure 28.

**Fungal Names:** FN571552.

**Etymology:** The specific epithet refers to the fewer number of rami.

In *Penicillium* subgenus *Aspergilloides* section *Lanata-Divaricata* series *Dalearum*.

**Typification:** China. Chongqing City, Jiangjin District, Simian Mountain Nature Reserve, 28°35′57″ N 106°26′51″ E, in soil of ant hole, 24 October 2020, Chang Liu, Zhao-Qing Zeng, Xin-Cun Wang and Huan-Di Zheng, culture, Zhi-Kang Zhang, CS04-09 (holotype HMAS 247903, ex-type strain CGMCC 3.25164).

**DNA barcodes:** ITS OQ870726, BenA OR051111, CaM OR051288, RPB2 OR051457.

**Colony diam.**, 7 days, 25 °C (unless stated otherwise): CYA 32–34 mm; CYA 37 °C 12–14 mm; CYA 5 °C no growth; MEA 38–40 mm; YES 40–42 mm; PDA 38–39 mm.

**Colony characteristics:** On CYA 25 °C, 7 days: Colonies nearly circular or irregular, slightly protuberant at centers, radially sulcate; margins wide, entire; mycelia white; texture velutinous; sporulation sparse; conidia *en masse* greyish green; soluble pigments absent; exudates absent; reverse buff, occasionally with yellow brown patches.

On CYA 37 °C, 7 days: Colonies nearly circular, concave at centers like volcanic vents, radially sulcate; margins narrow, entire; mycelia white; texture tight; sporulation absent; soluble pigments absent; exudates absent; reverse buff.

On MEA 25 °C, 7 days: Colonies nearly circular, plain, irregularly protuberant at centers; margins wide, entire; mycelia white; texture velutinous; sporulation sparse; conidia *en masse* light grey; soluble pigments absent; exudates yellow, clear; reverse buff to yellowish.

On YES 25 °C, 7 days: Colonies nearly circular, strongly sulcate, protuberant at centers; margins wide, entire; mycelia white; texture velutinous; sporulation very sparse; conidia *en masse* light grey; soluble pigments absent; exudates yellow, clear; reverse yellow brown with brownish cracks at centers.

On PDA 25 °C, 7 days: Colonies nearly circular, plain, irregularly protuberant at centers; margins wide, entire; mycelia white; texture velutinous; sporulation sparse; conidia *en masse* light grey; soluble pigments absent; exudates absent; reverse whitish.

**Micromorphology:** Conidiophores biverticillate, terverticillate to quaterverticillate; stipes smooth-walled, 75–250 × 2.0–3.0 μm; branches 2, 14–18 × 2.5–3.0 μm; rami 2, 10–47 × 2.0–3.5 μm; metulae 2–3, 10–33 × 2.0–3.5 μm; phialides ampulliform, tapering into very thin neck, 3–5 per metula, 5.0–10 × 2.5–4.5 μm; conidia subglobose to ellipsoidal, smooth-walled, 3.0–4.5 × 2.5–4.0 μm.

**Additional strains examined:** China. Chongqing City, Jiangjin District, Simian Mountain Nature Reserve, 28°35′57″ N 106°26′51″ E, in soil of ant hole, 24 October 2020, Chang Liu, Zhao-Qing Zeng, Xin-Cun Wang and Huan-Di Zheng, culture, Zhi-Kang Zhang, CS04-10; *ibid.*, CS04-11.

**Notes:** This species is a sister of *P. ausonanum* with strong support (BP = 100, PP = 1.00, Figure 6). It differs from the latter in one bp for BenA, four bp for CaM and six bp for RPB2. Morphologically, the new species is obviously different from the latter in slower growth rate on CYA (32–34 vs. 58–59 mm), MEA (38–40 vs. 61–62 mm) and YES (40–42 vs. 67–71 mm) at 25 °C and on CYA (12–14 vs. 38–39 mm) at 37 °C, terverticillate or quaterverticillate conidiophores, longer stipes (75–250 vs. 20–120 μm) and larger conidia (3.0–4.5 vs. 2.0–3.0 μm) [63].

**Figure 29:**
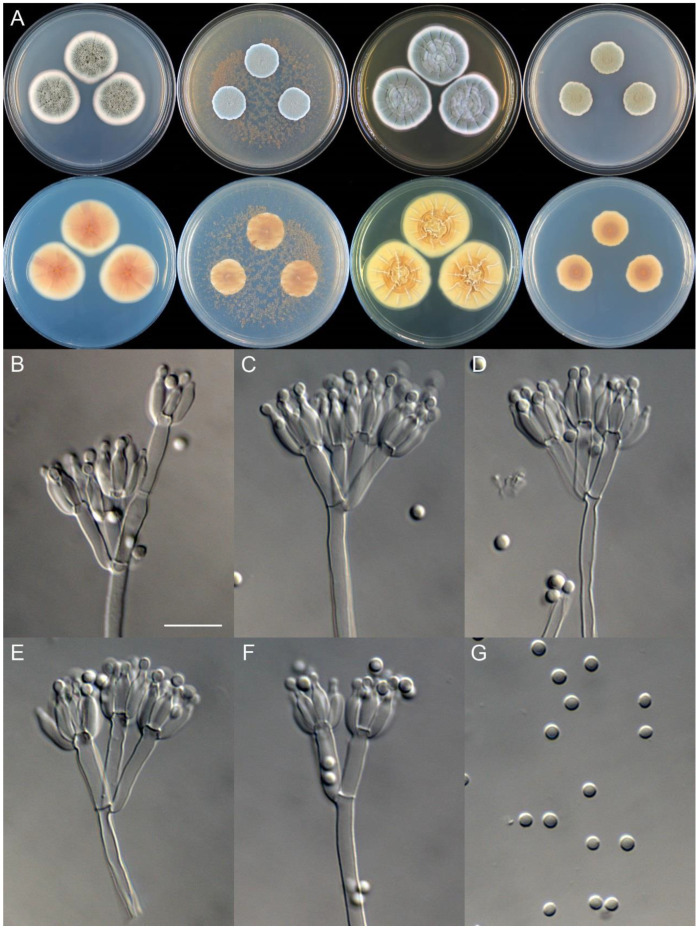
*Penicillium qii* (CS18-09). (**A**) Colonies: top row left to right, obverse CYA, MEA, YES, and PDA; bottom row left to right, reverse CYA, MEA, YES, and PDA; (**B**–**F**) Conidiophores; (**G**) Conidia. Bar: (**B**) = 10 µm, also for (**C**–**G**).

***Penicillium qii*** X.C. Wang & W.Y. Zhuang, sp. nov. Figure 29.

**Fungal Names:** FN571553.

**Etymology:** The specific epithet is in memory of the late Chinese mycologist Zu-Tong Qi (1926.12–2010.01), who described nine new species of *Aspergillus* and five ones of *Penicillium* from this country.

In *Penicillium* subgenus *Aspergilloides* section *Citrina* series *Sumatraensia*.

**Typification:** China. Chongqing City, Wushan County, Shuanglong Town, Huazhu Village, 31°9′48″ N 109°47′7″ E, in soil, 29 October 2020, Xin-Cun Wang, Huan-Di Zheng and Chang Liu, culture, Zhi-Kang Zhang, CS18-09 (holotype HMAS 247904, ex-type strain CGMCC 3.25165).

**DNA barcodes:** ITS OQ870878, BenA OR051080, CaM OR051257, RPB2 OR051430.

**Colony diam.**, 7 days, 25 °C (unless stated otherwise): CYA 31–32 mm; CYA 37 °C no growth; CYA 5 °C no growth; MEA 19–20 mm; YES 37–38 mm; PDA 19–21 mm.

**Colony characteristics:** On CYA 25 °C, 7 days: Colonies nearly circular, radially sulcate; margins wide, entire; mycelia white; texture velutinous; sporulation dense; conidia *en masse* viridian green; soluble pigments absent; exudates hyaline, clear, massive; reverse buff to orange brown.

On MEA 25 °C, 7 days: Colonies nearly circular, radially sulcate, slightly concave at centers; margins narrow, undulated; mycelia white; texture velutinous; sporulation dense; conidia *en masse* greyish green; soluble pigments absent; exudates absent; reverse light brown.

On YES 25 °C, 7 days: Colonies nearly circular, radially and concentrically sulcate, concave at centers; margins moderately wide, undulated; mycelia white; texture velutinous; sporulation dense; conidia *en masse* dull green; soluble pigments absent; exudates absent; reverse buff to yellow brown.

On PDA 25 °C, 7 days: Colonies irregular, protuberant at centers; margins narrow, irregular; mycelia white; texture velutinous; sporulation dense; conidia *en masse* dull green; soluble pigments absent; exudates absent; reverse light brown to light purplish brown.

**Micromorphology:** Conidiophores biverticillate, occasionally terverticillate; stipes smooth-walled, 135–275 × 2.0–3.0 μm; metulae 2–4, 12–17 × 3.0–3.5 μm; phialides ampulliform, tapering into very thin neck, 5–7 per metula, 7.5–9 × 2.5–3.5 μm; conidia subglobose to broad ellipsoidal, smooth-walled, 2.5–3.0 μm.

**Additional strains examined:** China. Chongqing City, Wushan County, Shuanglong Town, Huazhu Village, 31°9′48″ N 109°47′7″ E, in soil, 29 October 2020, Xin-Cun Wang, Huan-Di Zheng and Chang Liu, culture, Zhi-Kang Zhang, CS18-05; *ibid.*, CS18-27.

**Notes:** This species has close relationships to *P. cerradense* and *P. sumatraense* (Figure 3). It differs from *P. sumatraense* in 15 bp for BenA, 15 bp for CaM and 28 bp for RPB2, and differs from *P. cerradense* in 26 bp for BenA, 22 bp for CaM and 25 bp for RPB2. Morphologically, *P. qii* has slower growth rate on MYA at 25 °C (19–20 vs. 30–45 mm) than *P. sumatraense* [62]; and has faster growth rate on MYA at 25 °C (19–20 vs. 15 mm), slower growth rate on PDA at 25 °C (19–21 vs. 30 mm) than *P. cerradense* [70]. Additionally, *P. qii* does not produce sclerotia which are commonly found in *P. cerradense*.

**Figure 30:**
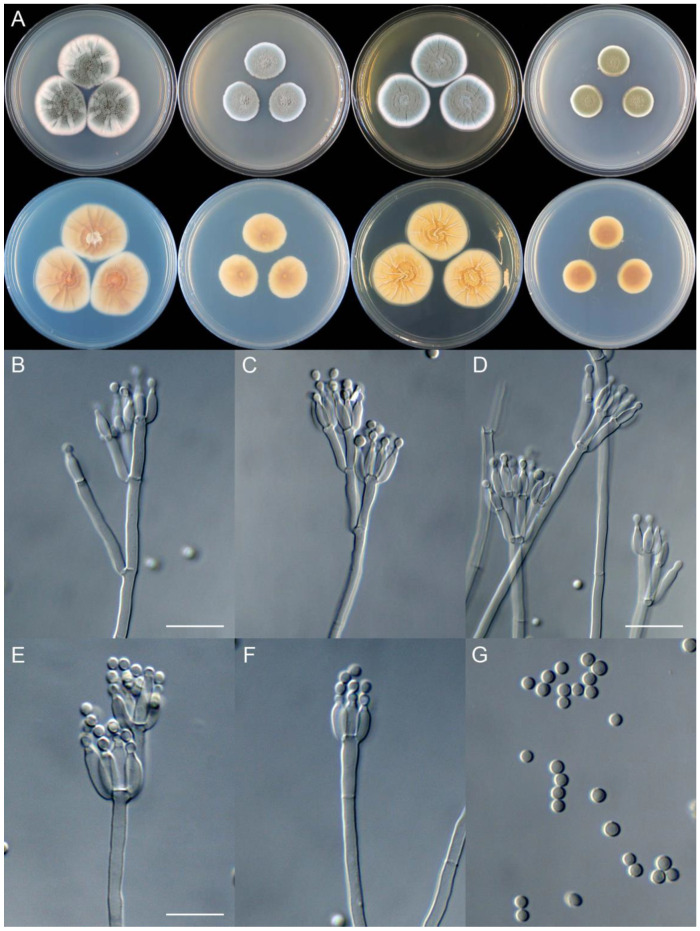
*Penicillium rarum* (CS15-04). (**A**) Colonies: top row left to right, obverse CYA, MEA, YES, and PDA; bottom row left to right, reverse CYA, MEA, YES, and PDA; (**B**–**F**) Conidiophores; (**G**) Conidia. Bars: (**B**) = 12.5 µm, also for (**C**); (**D**) = 15 µm; (**E**) = 10 µm, also for (**F**,**G**).

***Penicillium rarum*** X.C. Wang & W.Y. Zhuang, sp. nov. Figure 30.

**Fungal Names:** FN571554.

**Etymology:** The specific epithet refers to the fewer number of metulae.

In *Penicillium* subgenus *Aspergilloides* section *Citrina* series *Sumatraensia*.

**Typification:** China. Chongqing City, Fengjie County, Caotang Town, 31°5′29″ N 109°38′57″ E, in soil, 29 October 2020, Xin-Cun Wang, Huan-Di Zheng and Chang Liu, culture, Zhi-Kang Zhang, CS15-04 (holotype HMAS 247905, ex-type strain CGMCC 3.25166).

**DNA barcodes:** ITS OQ870881, BenA OR051083, CaM OR051260, RPB2 OR051432.

**Colony diam.**, 7 days, 25 °C (unless stated otherwise): CYA 28–36 mm; CYA 37 °C no growth; CYA 5 °C no growth; MEA 17–23 mm; YES 32–42 mm; PDA 18–21 mm.

**Colony characteristics:** On CYA 25 °C, 7 days: Colonies nearly circular, radially sulcate; margins moderately wide, entire; mycelia white; texture velutinous; sporulation dense; conidia *en masse* viridian green; soluble pigments absent; exudates hyaline, clear; reverse buff to brownish.

On MEA 25 °C, 7 days: Colonies nearly circular, slightly protuberant; margins narrow, entire or irregular; mycelia white; texture velutinous; sporulation dense; conidia *en masse* dull green; soluble pigments absent; exudates absent; reverse buff to pale brown.

On YES 25 °C, 7 days: Colonies nearly circular, radially and concentrically sulcate; margins moderately wide, undulated; mycelia white; texture velutinous; sporulation dense; conidia *en masse* dull green; soluble pigments absent; exudates absent; reverse buff with brownish cracks.

On PDA 25 °C, 7 days: Colonies irregular, protuberant at centers; margins narrow, entire or irregular; mycelia white; texture velutinous; sporulation dense; conidia *en masse* dull green; soluble pigments absent; exudates absent; reverse white to light brown.

**Micromorphology:** Conidiophores terverticillate, biverticillate or monoverticillate; stipes smooth-walled, 285–515 × 2.0–3.0 μm; rami 2, 14–20 × 2.5–3.0 μm; metulae 2–5, 10–15 × 2.0–3.0 (–4.5) μm; phialides ampulliform, tapering into very thin neck, 5–7 per metula, 7–8.5 × 2.0–3.0 μm; conidia subglobose, smooth-walled, 2.5–3.0 μm.

**Additional strains examined:** China. Chongqing City, Fengjie County, Caotang Town, 31°5′29″ N 109°38′57″ E, in soil, 29 October 2020, Xin-Cun Wang, Huan-Di Zheng and Chang Liu, culture, Zhi-Kang Zhang, CS15-05; *ibid.*, Wushan County, Shuanglong Town, Huazhu Village, 31°9′48″ N 109°47′7″ E, in soil, 29 October 2020, Xin-Cun Wang, Huan-Di Zheng and Chang Liu, culture, Zhi-Kang Zhang, CS18-06.

**Notes:** This species has close relationship with *P. qii* and *P. vulgatum* (Figure 3). It differs from *P. qii* in 8 bp for BenA, 12 bp for CaM and 30 bp for RPB2; and differs from *P. vulgatum* in 7 bp for BenA, 8 bp for CaM and 34 bp for RPB2. Morphologically, *P. rarum* has terverticillate or monoverticillate conidiophores which are seldom found in *P. qii* and *P. vulgatum*. Additionally, the new species grows slower than *P. vulgatum* on MEA and YES at 25 °C, especially on PDA at 25 °C (18–21 vs. 23–25 mm).

**Figure 31:**
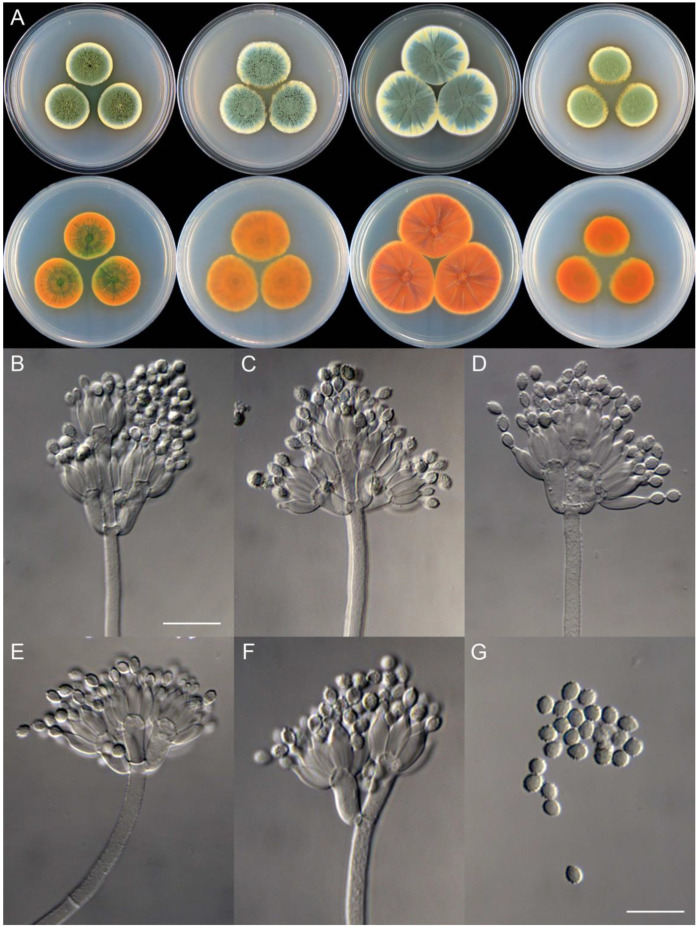
*Penicillium scruposum* (CS13-09). (**A**) Colonies: top row left to right, obverse CYA, MEA, YES, and PDA; bottom row left to right, reverse CYA, MEA, YES, and PDA; (**B**–**F**) Conidiophores; (**G**) Conidia. Bars: (**B**) = 12.5 µm, also for (**C**–**E**); (**G**) = 10 µm, also for (**F**).

***Penicillium scruposum*** X.C. Wang & W.Y. Zhuang, sp. nov. Figure 31.

**Fungal Names:** FN571555.

**Etymology:** The specific epithet refers to the rough-walled conidia.

In *Penicillium* subgenus *Aspergilloides* section *Sclerotiorum* series *Herqueorum*.

**Typification:** China. Chongqing City, Wanzhou City, Wangerbao Nature Reserve, Longju Town, Wutong Village, 30°36′26″ N 108°38′24″ E, in soil, 28 October 2020, Xin-Cun Wang, Huan-Di Zheng and Chang Liu, culture, Zhi-Kang Zhang, CS13-09 (holotype HMAS 247906, ex-type strain CGMCC 3.25167).

**DNA barcodes:** ITS OQ870841, BenA OR051190, CaM OR051365, RPB2 OR062055.

**Colony diam.**, 7 days, 25 °C (unless stated otherwise): CYA 26–27 mm; CYA 37 °C no growth; CYA 5 °C no growth; MEA 29–31 mm; YES 39–40 mm; PDA 25–26 mm.

**Colony characteristics:** On CYA 25 °C, 7 days: Colonies nearly circular, slightly sulcate; margins narrow, entire; mycelia yellow; texture velutinous; sporulation dense; conidia *en masse* viridian green; soluble pigments yellow; exudates hyaline, clear; reverse orange, green to olive at centers.

On MEA 25 °C, 7 days: Colonies nearly circular; margins narrow to moderately wide, entire; mycelia yellow; texture velutinous; sporulation dense; conidia *en masse* yellowish green; soluble pigments absent; exudates hyaline, clear; reverse orange with a brown tint.

On YES 25 °C, 7 days: Colonies nearly circular, radially sulcate, concave at centers; margins moderately wide, undulated; mycelia yellow; texture velutinous; sporulation dense; conidia *en masse* dull green; soluble pigments absent; exudates absent; reverse orange brown with radiate branches.

On PDA 25 °C, 7 days: Colonies nearly circular, slightly protuberant at centers; margins narrow, entire; mycelia yellow; texture velutinous; sporulation dense; conidia *en masse* dull green; soluble pigments orange; exudates absent; reverse brownish orange.

**Micromorphology:** Conidiophores biverticillate; stipes finely rough-walled, 185–435 × 3.0–3.5 μm; metulae 3–5, 9–23 × 2.5–6.5 μm; phialides ampulliform, tapering into very thin neck, 6–9 per metula, 8.5–11.5 × 2.5–4.0 μm; conidia ellipsoidal to broad fusiform, rough-walled to echinulate, 3.5–4.0 × 3.0–3.5 μm.

**Additional strains examined:** China. Chongqing City, Wanzhou City, Wangerbao Nature Reserve, Longju Town, Wutong Village, 30°36′26″ N 108°38′24″ E, in soil, 28 October 2020, Xin-Cun Wang, Huan-Di Zheng and Chang Liu, culture, Zhi-Kang Zhang, CS13-19; *ibid.*, CS13-20.

**Notes:** This species is a sister of *P. subasperum* with strong support (BP = 96, PP = 1.00, Figure 7). It differs from the latter in 14 bp for BenA, 20 bp for CaM and 15 bp for RPB2. Morphologically, it differs in slower growth rate on CYA at 25 °C (26–27 vs. 30–32 mm), finely rough-walled stipes, larger metulae (9–23 × 2.5–6.5 vs. 8.5–13.5 × 3.0–4.5 μm) and echinulate conidia.

**Figure 32:**
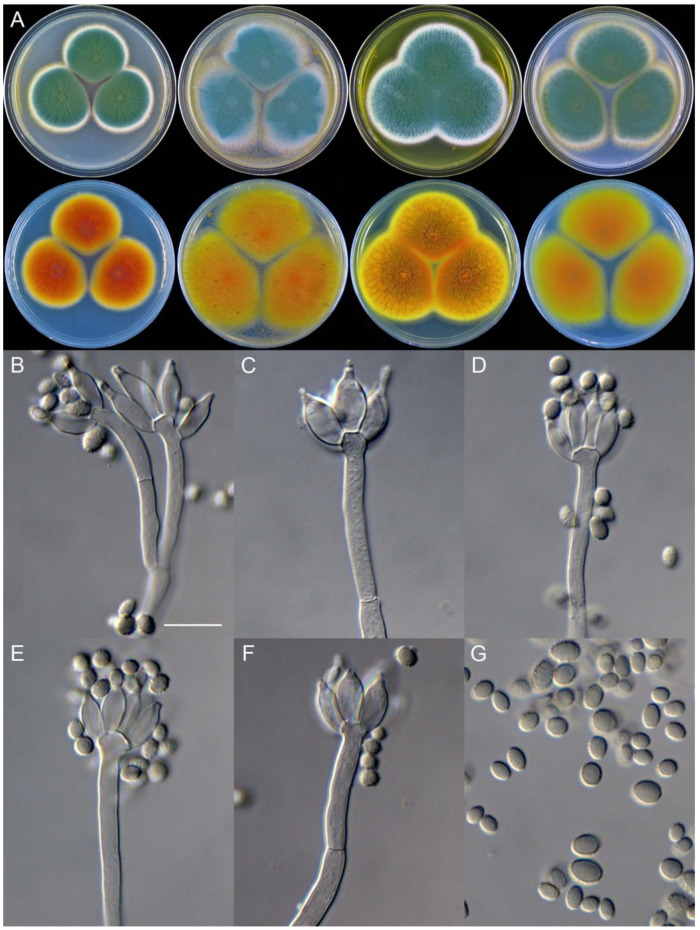
*Penicillium shihii* (CS22-03). (**A**) Colonies: top row left to right, obverse CYA, MEA, YES, and PDA; bottom row left to right, reverse CYA, MEA, YES, and PDA; (**B**–**F**) Conidiophores; (**G**) Conidia. Bar: (**B**) = 10 µm, also for (**C**–**G**).

***Penicillium shihii*** X.C. Wang & W.Y. Zhuang, sp. nov. Figure 32.

**Fungal Names:** FN571556.

**Etymology:** The specific epithet is named after the late Chinese microbiologist You-Kuang Shih (1905.10–1991.01). He is a pioneer on taxonomy of this group, and published five *Aspergillus* taxa and three *Penicillium* species.

In *Penicillium* subgenus *Aspergilloides* section *Aspergilloides* series *Livida*.

**Typification:** China. Chongqing City, Wushan County, Wulipo National Nature Reserve, 31°22′59″ N 109°56′11″ E, in soil, 29 October 2020, Xin-Cun Wang, Huan-Di Zheng and Chang Liu, culture, Zhi-Kang Zhang, CS22-03 (holotype HMAS 247907, ex-type strain CGMCC 3.25168).

**DNA barcodes:** ITS OQ870799, BenA OR051060, CaM OR051239, RPB2 OR051412.

**Colony diam.**, 7 days, 25 °C (unless stated otherwise): CYA 40–42 mm; CYA 37 °C no growth; CYA 5 °C no growth; MEA 58–60 mm; YES 54–56 mm; PDA 57–60 mm.

**Colony characteristics:** On CYA 25 °C, 7 days: Colonies nearly circular, radially sulcate, slightly concave at centers; margins moderately wide, entire; mycelia white; texture velutinous; sporulation dense; conidia *en masse* bluish green; soluble pigments absent; exudates absent; reverse orange, yellow at margins.

On MEA 25 °C, 7 days: Colonies nearly circular, plain; margins wide, entire; mycelia colorless; texture velutinous; sporulation dense, sporulation area irregular, star-shaped; conidia *en masse* dull green; soluble pigments absent; exudates absent; reverse orange yellow and paler at margins.

On YES 25 °C, 7 days: Colonies nearly circular, strongly sulcate; margins moderately wide, fimbriate; mycelia white; texture velutinous; sporulation dense; conidia *en masse* greenish blue; soluble pigments absent; exudates absent; reverse orange brown and yellow at margins.

On PDA 25 °C, 7 days: Colonies nearly circular, plain, slightly protuberant at centers; margins wide, entire; mycelia colorless; texture velutinous; sporulation dense; conidia *en masse* bluish green; soluble pigments absent; exudates absent; reverse light orange brown, greenish yellow at margins.

**Micromorphology:** Conidiophores monoverticillate or biverticillate; stipes smooth–rough-walled, 400–575 × 2.0–4.5 μm; metulae 2, 28–32 × 2.5–3.5 μm; phialides obovate to ampulliform, tapering into very thin neck, 5–7 per metula, 9–12.5 × 3.0–5.5 μm; conidia ellipsoidal, rough-walled, 4.0–6.0 × 3.0–4.5 μm.

**Additional strains examined:** China. Chongqing City, Wushan County, Wulipo National Nature Reserve, 31°22′59″ N 109°56′11″ E, in soil, 29 October 2020, Xin-Cun Wang, Huan-Di Zheng and Chang Liu, culture, Zhi-Kang Zhang, CS20-40; *ibid.*, CS20-44; Sichuan Province, Dazhou City, Xuanhan County, Bashan Grand Canyon, 31°39′44″ N 108°51′17″ E, in soil, 1 November 2020, Xin-Cun Wang, Huan-Di Zheng and Chang Liu, culture, Zhi-Kang Zhang, CS30-12; *ibid.*, in soil of ant hole, CS31-05; in soil of ant hole, 1 November 2020, Xin-Cun Wang, Huan-Di Zheng and Chang Liu, culture, Zhi-Kang Zhang, CS32-01.

**Notes:** This species is a member of Ser. *Livida* and sister to the other known species: *P. kananaskense*, *P. lividum* and *P. odoratum* (=*P. lividum* according to Pitt [62]), but of significant differences in sequence (Figure 2). Morphologically, it differs from *P. kananaskense* in faster growth rates on CYA (40–42 vs. 27–35 mm) and MEA (58–60 vs. 43–52 mm) at 25 °C, longer stipe (400–575 vs. 200–400 μm) and broader phialides (3.0–5.5 vs. 2.5–4.0 μm) [71]; and it differs from *P. lividum* and *P. odoratum* in faster growth rate on MEA (58–60 vs. 40–45 mm) at 25 °C, broader phialides (3.0–5.5 vs. 2.5–3.0 μm) and larger conidia (4.0–6.0 × 3.0–4.5 vs. 3.5–4.0 × 2.5–3.0 μm) [62].

**Figure 33:**
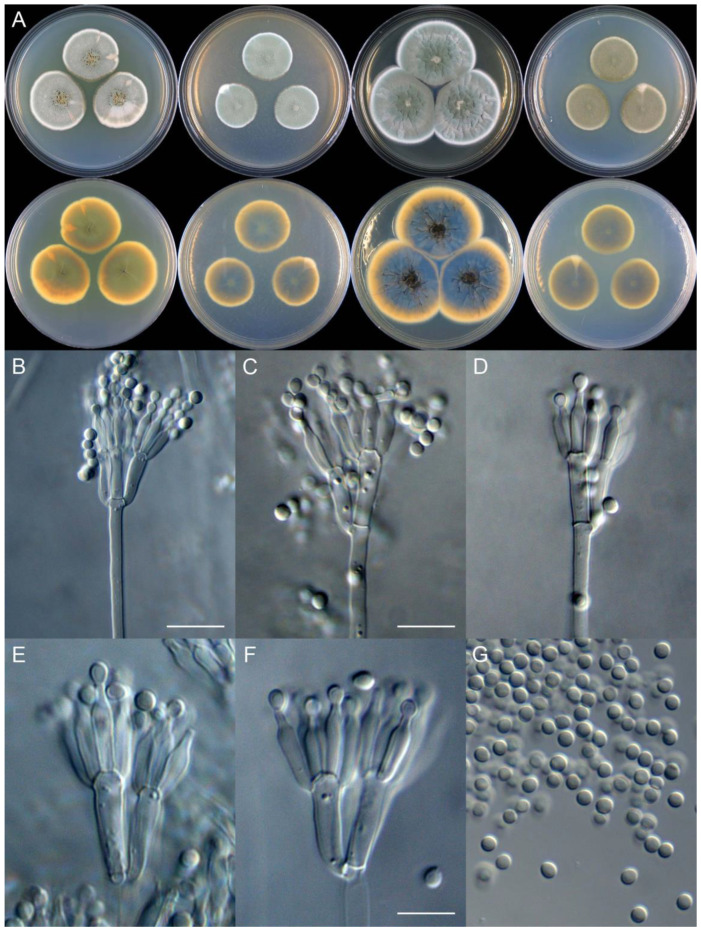
*Penicillium sichuanense* (CS32-04). (**A**) Colonies: top row left to right, obverse CYA, MEA, YES, and PDA; bottom row left to right, reverse CYA, MEA, YES, and PDA; (**B**–**F**) Conidiophores; (**G**) Conidia. Bars: (**B**) = 12.5 µm; (**C**) = 10 µm, also for (**D**,**G**); (**F**) = 7.5 µm, also for (**E**).

***Penicillium sichuanense*** X.C. Wang & W.Y. Zhuang, sp. nov. Figure 33.

**Fungal Names:** FN571557.

**Etymology:** The specific epithet refers to the type locality.

In *Penicillium* subgenus *Aspergilloides* section *Gracilenta* series *Estinogena*.

**Typification:** China. Sichuan Province, Dazhou City, Xuanhan County, Bashan Grand Canyon, 31°39′44″ N 108°51′17″ E, in soil of ant hole, 1 November 2020, Xin-Cun Wang, Huan-Di Zheng and Chang Liu, culture, Zhi-Kang Zhang, CS32-04 (holotype HMAS 247908, ex-type strain CGMCC 3.25169).

**DNA barcodes:** ITS OQ870825, BenA OR051101, CaM OR051278, RPB2 OR051447.

**Colony diam.**, 7 days, 25 °C (unless stated otherwise): CYA 31–36 mm; CYA 37 °C no growth; CYA 5 °C no growth; MEA 26–29 mm; YES 47–49 mm; PDA 27–30 mm.

**Colony characteristics:** On CYA 25 °C, 7 days: Colonies nearly circular, plain or sulcate, slightly protuberant or concave at centers; margins narrow, entire; mycelia white; texture velutinous; sporulation dense; conidia *en masse* greyish green; soluble pigments yellow or absent; exudates yellow, clear; reverse olive and pale orange at margins.

On MEA 25 °C, 7 days: Colonies nearly circular, plain, slightly protuberant at centers; margins narrow, entire; mycelia white; texture velutinous, but floccose at centers or not; sporulation dense; conidia *en masse* dull green; soluble pigments absent; exudates absent; reverse dark mouse grey, pale orange at margins.

On YES 25 °C, 7 days: Colonies nearly circular, strongly sulcate; margins narrow to moderately wide, fimbriate; mycelia white; texture velutinous; sporulation dense; conidia *en masse* greyish green; soluble pigments absent; exudates absent; reverse dark purplish, pale orange at margins.

On PDA 25 °C, 7 days: Colonies nearly circular, plain, slightly protuberant at centers; margins narrow, entire; mycelia white; texture velutinous; sporulation dense; conidia *en masse* dull green; soluble pigments yellow or absent; exudates absent; reverse dark mouse grey, yellowish at margins.

**Micromorphology:** Conidiophores biverticillate; stipes smooth-walled, 25–135 × 2.5–3.0 μm; metulae 3–5, 12.5–15.5 × 3.0–4.0 μm; phialides acerose, tapering into very thin neck, 5–7 per metula, 10–12 × 2.5–3.5 μm; conidia subglobose, smooth-walled, 2.5–3.5 μm.

**Notes:** This species is a sister to *P. estinogenum* with strong support (BP = 100, PP = 1.00, Figure 5), It differs from the latter in 40 bp for BenA and 58 bp for CaM. Morphologically, it differs in smooth-walled stipes [21].

**Figure 34:**
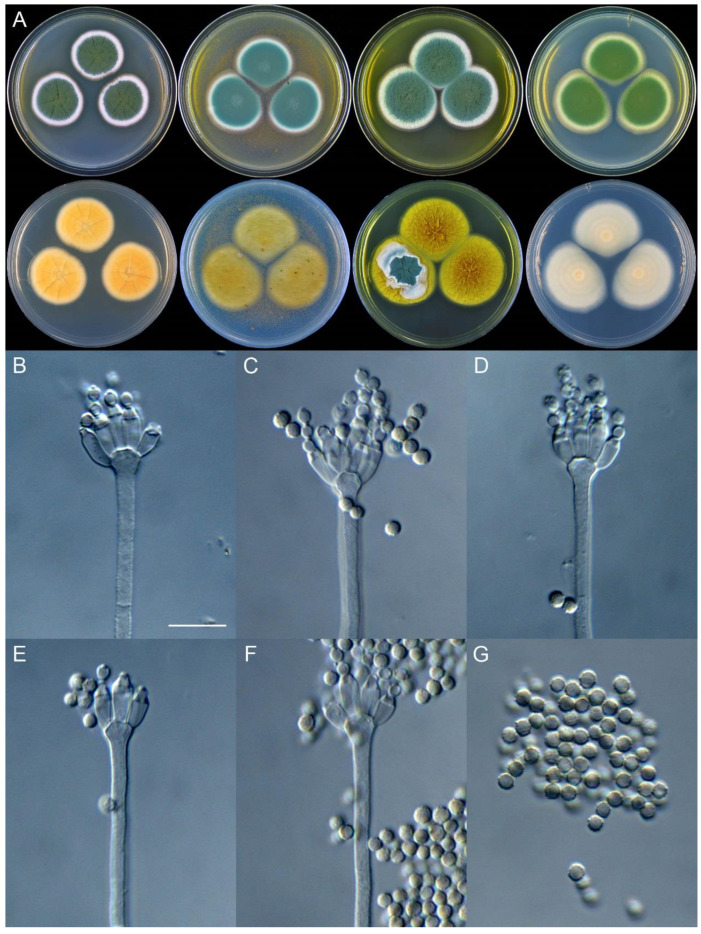
*Penicillium simianshanicum* (CS04-04). (**A**) Colonies: top row left to right, obverse CYA, MEA, YES, and PDA; bottom row left to right, reverse CYA, MEA, YES, and PDA; (**B**–**F**) Conidiophores; (**G**) Conidia. Bar: (**B**) = 10 µm, also for (**C**–**G**).

***Penicillium simianshanicum*** X.C. Wang & W.Y. Zhuang, sp. nov. Figure 34.

**Fungal Names:** FN571558.

**Etymology:** The specific epithet refers to the type locality.

In *Penicillium* subgenus *Aspergilloides* section *Aspergilloides* series *Simianshanica*.

**Typification:** China. Chongqing City, Jiangjin District, Simian Mountain Nature Reserve, 28°35′57″ N 106°26′51″ E, in soil of ant hole, 24 October 2020, Chang Liu, Zhao-Qing Zeng, Xin-Cun Wang and Huan-Di Zheng, culture, Zhi-Kang Zhang, CS04-04 (holotype HMAS 247909, ex-type strain CGMCC 3.25170).

**DNA barcodes:** ITS OQ870805, BenA OR051066, CaM OR051245, RPB2 OR051418.

**Colony diam.**, 7 days, 25 °C (unless stated otherwise): CYA 32–34 mm; CYA 37 °C no growth; CYA 5 °C no growth; MEA 37–39 mm; YES 40–41 mm; PDA 39–40 mm.

**Colony characteristics:** On CYA 25 °C, 7 days: Colonies nearly circular, radially sulcate, slightly concave at centers; margins wide, entire; mycelia white; texture velutinous; sporulation dense; conidia *en masse* bluish green; soluble pigments absent; exudates yellow, clear; reverse light orange yellow.

On MEA 25 °C, 7 days: Colonies nearly circular, plain; margins wide, entire; mycelia white; texture velutinous, but floccose at centers; sporulation dense; conidia *en masse* bluish green; soluble pigments absent; exudates absent; reverse buff.

On YES 25 °C, 7 days: Colonies nearly circular, strongly sulcate, with funiculose hyphae at centers, protuberant or not; margins moderately wide, entire; mycelia white; texture velutinous; sporulation dense; conidia *en masse* bluish green; soluble pigments absent; exudates absent; reverse brownish yellow with radiate branches.

On PDA 25 °C, 7 days: Colonies nearly circular, plain, slightly protuberant at centers; margins wide, entire; mycelia white; texture velutinous; sporulation dense; conidia *en masse* bluish green; soluble pigments absent; exudates absent; reverse whitish to pale buff.

**Micromorphology:** Conidiophores monoverticillate; stipes septate, rough-walled, 80–160 × 2.5–3.5 μm; phialides ampulliform, tapering into very thin neck, 8–12, 8–14 × 3.0–4.0 μm; conidia subglobose, rough-walled, 3.0–3.5 μm.

**Additional strains examined:** China. Chongqing City, Jiangjin District, Simian Mountain Nature Reserve, 28°35′57″ N 106°26′51″ E, in soil of ant hole, 24 October 2020, Chang Liu, Zhao-Qing Zeng, Xin-Cun Wang and Huan-Di Zheng, culture, Zhi-Kang Zhang, CS04-05; *ibid.*, CS04-08.

**Notes:** This species is phylogenetically close to taxa of ser. *Verhageniorum* but having significant branch distance (Figure 2). The species of ser. *Verhageniorum* have biverticillate or divaricate conidiophores [59], but *P. simianshanicum* differs from them in monoverticillate conidiophores. A separate series, ser. *Simianshanica*, has been proposed to accommodate this morphologically distinct species.

**Figure 35:**
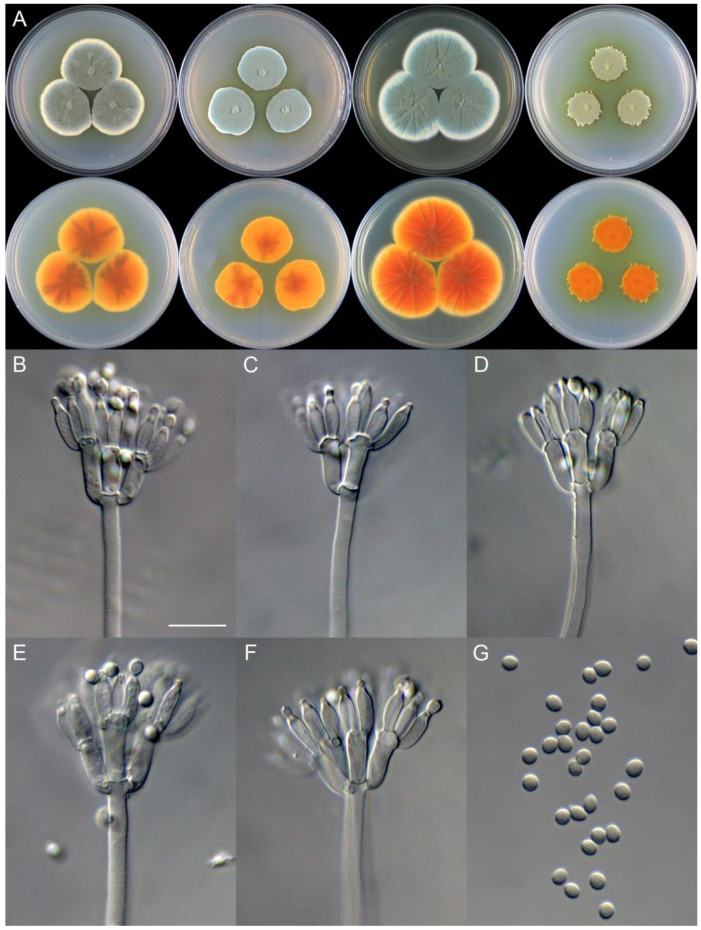
*Penicillium sphaerioides* (CS02-11). (**A**) Colonies: top row left to right, obverse CYA, MEA, YES, and PDA; bottom row left to right, reverse CYA, MEA, YES, and PDA; (**B**–**F**) Conidiophores; (**G**) Conidia. Bar: (**B**) = 10 µm, also for (**C**–**G**).

***Penicillium sphaerioides*** X.C. Wang & W.Y. Zhuang, sp. nov. Figure 35.

**Fungal Names:** FN571563.

**Etymology:** The specific epithet refers to the shape of the conidia.

In *Penicillium* subgenus *Aspergilloides* section *Sclerotiorum* series *Herqueorum*.

**Typification:** China. Chongqing City, Beibei District, Jinyun Mountain National Nature Reserve, 29°50′18″ N 106°23′45″ E, in soil, 23 October 2020, Chang Liu, Zhao-Qing Zeng, Xin-Cun Wang and Huan-Di Zheng, culture, Zhi-Kang Zhang, CS02-11 (holotype HMAS 247914, ex-type strain CGMCC 3.25175).

**DNA barcodes:** ITS OQ870850, BenA OR051199, CaM OR051374, RPB2 OR062064.

**Colony diam.**, 7 days, 25 °C (unless stated otherwise): CYA 34–36 mm; CYA 37 °C no growth; CYA 5 °C no growth; MEA 25–28 mm; YES 44–46 mm; PDA 19–22 mm.

**Colony characteristics:** On CYA 25 °C, 7 days: Colonies nearly circular, protuberant at centers; margins narrow, entire; mycelia yellow; texture velutinous; sporulation dense; conidia *en masse* viridian green; soluble pigments yellow; exudates yellow, clear; reverse yellow to orange, with brown radiate branches.

On MEA 25 °C, 7 days: Colonies nearly circular, plain, protuberant at centers; margins narrow, entire; mycelia yellow; texture velutinous; sporulation dense; conidia *en masse* dull green; soluble pigments yellow; exudates absent; reverse light orange, with brownish radiate branches or patches.

On YES 25 °C, 7 days: Colonies nearly circular, radially sulcate, concave at centers; margins narrow to moderately wide, undulated; mycelia yellow; texture velutinous to funiculose; sporulation dense; conidia *en masse* dull green; soluble pigments absent; exudates absent; reverse reddish orange, yellow at margins.

On PDA 25 °C, 7 days: Colonies irregular, protuberant at centers; margins narrow, irregular; mycelia white; texture velutinous; sporulation dense; conidia *en masse* dark green; soluble pigments orange; exudates absent; reverse reddish orange.

**Micromorphology:** Conidiophores biverticillate; stipes smooth to rough-walled, 135–285 × 2.5–3.5 μm; metulae 3–5, 8.5–12 × 2.5–5.0 μm; phialides ampulliform to acerose, tapering into very thin neck, 5–6 per metula, 7.5–11 × 2.5–3.0 μm; conidia subglobose, ellipsoidal to broad fusiform, finely rough-walled, 3.0–3.5 × 2.5–3.0 μm.

**Additional strains examined:** China. Chongqing City, Beibei District, Jinyun Mountain National Nature Reserve, 29°50′18″ N 106°23′45″ E, in soil, 23 October 2020, Chang Liu, Zhao-Qing Zeng, Xin-Cun Wang and Huan-Di Zheng, culture, Zhi-Kang Zhang, CS02-12; *ibid.*, Jiangjin District, Simian Mountain Nature Reserve, 28°35′57″ N 106°26′51″ E, in soil of ant hole, 24 October 2020, Chang Liu, Zhao-Qing Zeng, Xin-Cun Wang and Huan-Di Zheng, culture, Zhi-Kang Zhang, CS04-03.

**Notes:** This species is a sister of *P. scruposum* and *P. subasperum* with strong support (BP = 100, PP = 1.00, Figure 7). It differs from *P. scruposum* in 23 bp for BenA, 26 bp for CaM and 21 bp for RPB2; and differs from *P. subasperum* in 21 bp for BenA, 19 bp for CaM and 23 bp for RPB2. Morphologically, it differs from *P. scruposum* in faster growth rates on CYA (34–36 vs. 26–27 mm) and YES (44–46 vs. 39–40 mm), slower growth rate on PDA (19–22 vs. 25–26 mm), irregular margins on PDA, shorter stipes (135–285 vs. 185–435 μm) and metulae (8.5–12 vs. 9–23 μm), fewer phialides per metula (5–6 vs. 6–9) and subglobose conidia; and differs from *P. subasperum* in faster growth rate on YES (44–46 vs. 37–39 mm) and slower growth rate on PDA (19–22 vs. 24–25 mm).

**Figure 36:**
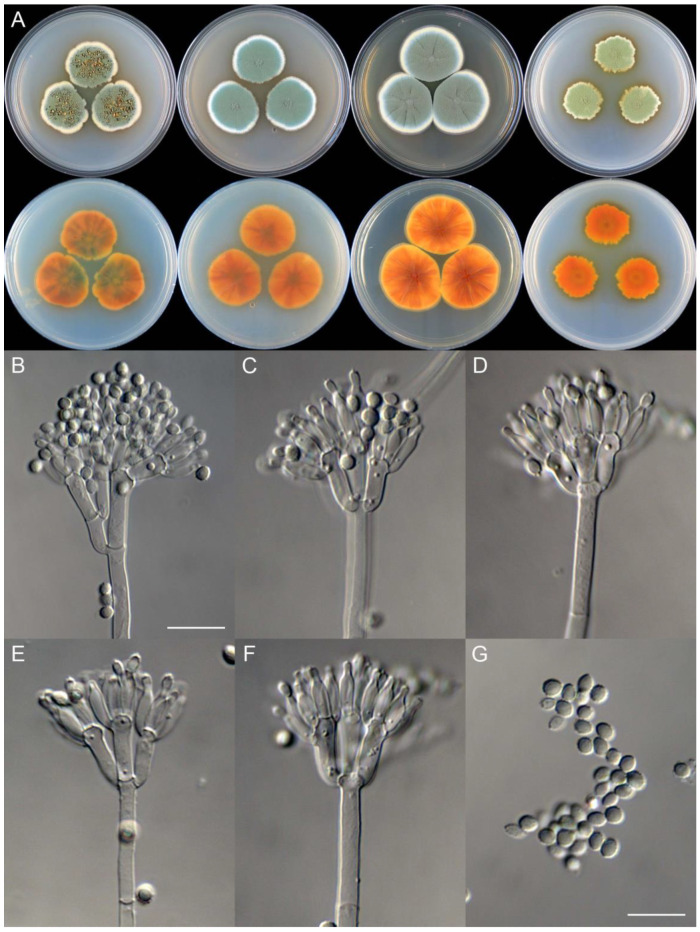
*Penicillium subasperum* (CS04-02). (**A**) Colonies: top row left to right, obverse CYA, MEA, YES, and PDA; bottom row left to right, reverse CYA, MEA, YES, and PDA; (**B**–**F**) Conidiophores; (**G**) Conidia. Bars: (**B**) = 12.5 µm; (**G**) = 10 µm, also for (**C**–**F**).

***Penicillium subasperum*** X.C. Wang & W.Y. Zhuang, sp. nov. Figure 36.

**Fungal Names:** FN571561.

**Etymology:** The specific epithet refers to the rough-walled conidia.

In *Penicillium* subgenus *Aspergilloides* section *Sclerotiorum* series *Herqueorum*.

**Typification:** China. Chongqing City, Jiangjin District, Simian Mountain Nature Reserve, 28°35′57″ N 106°26′51″ E, in soil of ant hole, 24 October 2020, Chang Liu, Zhao-Qing Zeng, Xin-Cun Wang and Huan-Di Zheng, culture, Zhi-Kang Zhang, CS04-02 (holotype HMAS 247912, ex-type strain CGMCC 3.25173).

**DNA barcodes:** ITS OQ870849, BenA OR051198, CaM OR051373, RPB2 OR062063.

**Colony diam.**, 7 days, 25 °C (unless stated otherwise): CYA 30–32 mm; CYA 37 °C no growth; CYA 5 °C no growth; MEA 29–31 mm; YES 37–39 mm; PDA 24–25 mm.

**Colony characteristics:** On CYA 25 °C, 7 days: Colonies irregular, slightly sulcate; margins narrow, entire or irregular; mycelia yellow; texture velutinous; sporulation dense; conidia *en masse* viridian green; soluble pigments yellow; exudates bright yellow, clear, massive; reverse yellow to orange, with greenish patches.

On MEA 25 °C, 7 days: Colonies nearly circular, protuberant at centers; margins narrow, entire or irregular; mycelia yellow; texture velutinous; sporulation dense; conidia *en masse* dark green; soluble pigments light brown; exudates absent; reverse brownish orange, with reddish brown radiate branches.

On YES 25 °C, 7 days: Colonies nearly circular, radially sulcate, concave at centers; margins narrow, entire; mycelia yellow; texture velutinous; sporulation dense; conidia *en masse* dark green; soluble pigments absent; exudates absent; reverse yellow to orange, with brownish red radiate branches.

On PDA 25 °C, 7 days: Colonies irregular, protuberant at centers; margins narrow, irregular; mycelia yellow; texture velutinous; sporulation dense; conidia *en masse* dark green; soluble pigments orange brown; exudates absent; reverse reddish orange.

**Micromorphology:** Conidiophores biverticillate, occasionally terverticillate; stipes smooth-walled, 125–315 × 2.5–4.0 μm; rami 2, 8.0–18 × 3.0–4.0 μm; metulae 2–4, 8.5–13.5 × 3.0–4.5 μm; phialides ampulliform to acerose, tapering into very thin neck, 5–8 per metula, 7.5–9.0 × 2.5–3.5 μm; conidia ellipsoidal to broad fusiform, rough-walled, 3.0–4.0 × 2.5–3.5 μm.

**Notes:** This species is a sister of *P. scruposum* with strong support (BP = 96, PP = 1.00, Figure 7). It differs from the latter in 14 bp for BenA, 20 bp for CaM and 15 bp for RPB2. Morphologically, it differs in faster growth rate on CYA at 25 °C (30–32 mm vs. 26–27), smooth-walled stipes, smaller metulae (8.5–13.5 × 3.0–4.5 vs. 9–23 × 2.5–6.5 μm) and rough-walled instead of echinulate conidia.

**Figure 37:**
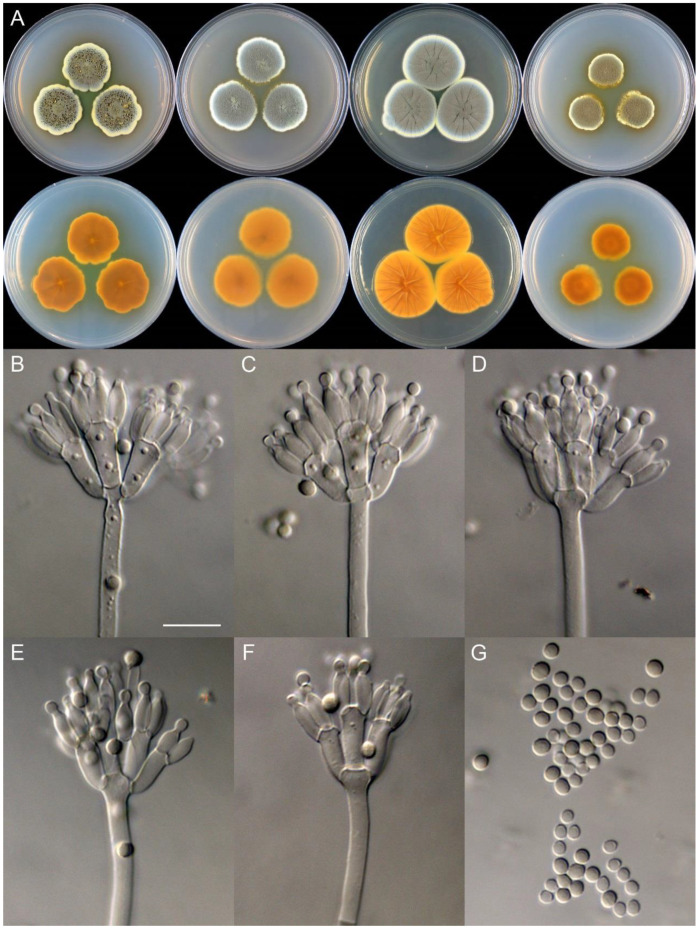
*Penicillium subglobosum* (CS16-01). (**A**) Colonies: top row left to right, obverse CYA, MEA, YES, and PDA; bottom row left to right, reverse CYA, MEA, YES, and PDA; (**B**–**F**) Conidiophores; (**G**) Conidia. Bar: (**B**) = 10 µm, also for (**C**–**G**).

***Penicillium subglobosum*** X.C. Wang & W.Y. Zhuang, sp. nov. Figure 37.

**Fungal Names:** FN571559.

**Etymology:** The specific epithet refers to the subglobose to ellipsoidal conidia.

In *Penicillium* subgenus *Aspergilloides* section *Sclerotiorum* series *Herqueorum*.

**Typification:** China. Chongqing City, Fengjie County, Caotang Town, 31°5′29″ N 109°38′57″ E, in soil, 29 October 2020, Xin-Cun Wang, Huan-Di Zheng and Chang Liu, culture, Zhi-Kang Zhang, CS16-01 (holotype HMAS 247910, ex-type strain CGMCC 3.25171).

**DNA barcodes:** ITS OQ870844, BenA OR051193, CaM OR051368, RPB2 OR062058.

**Colony diam.**, 7 days, 25 °C (unless stated otherwise): CYA 27–29 mm; CYA 37 °C no growth; CYA 5 °C no growth; MEA 28–29 mm; YES 31–36 mm; PDA 21–23 mm.

**Colony characteristics:** On CYA 25 °C, 7 days: Colonies nearly circular or irregular; margins narrow, entire; mycelia yellow; texture velutinous; sporulation dense; conidia *en masse* yellowish green; soluble pigments light green or light brown; exudates yellow, clear; reverse light brownish orange.

On MEA 25 °C, 7 days: Colonies nearly circular, protuberant at centers; margins narrow, entire; mycelia yellow; texture velutinous; sporulation dense; conidia *en masse* viridian green; soluble pigments absent; exudates hyaline, tiny; reverse yellow brown.

On YES 25 °C, 7 days: Colonies nearly circular, radially and concentrically sulcate, concave at centers; margins narrow to moderately wide, undulated; mycelia yellow; texture velutinous; sporulation dense; conidia *en masse* dull green; soluble pigments absent; exudates absent; reverse light orange brown with radiate branches.

On PDA 25 °C, 7 days: Colonies nearly circular or irregular; margins narrow, entire or irregular; mycelia yellow; texture velutinous; sporulation moderately dense; conidia *en masse* bluish green; soluble pigments light brown; exudates hyaline, tiny; reverse orange brown.

**Micromorphology:** Conidiophores biverticillate; stipes smooth to rough-walled, 110–245 × 3.0–3.5 μm; metulae 5–7, 9–13.5 × 3.5–6.0 μm; phialides ampulliform, tapering into very thin neck, 5–8 per metula, 7–10.5 × 2.5–4.0 μm; conidia subglobose to ellipsoidal, finely rough-walled, 3.0–4.0 × 2.5–3.5 μm.

**Additional strains examined:** China. Chongqing City, Fengjie County, Caotang Town, 31°5′29″ N 109°38′57″ E, in soil, 29 October 2020, Xin-Cun Wang, Huan-Di Zheng and Chang Liu, culture, Zhi-Kang Zhang, CS16-02; *ibid.*, CS16-04; *ibid.*, Wushan County, Shuanglong Town, Huazhu Village, 31°9′48″ N 109°47′7″ E, in soil, 29 October 2020, Xin-Cun Wang, Huan-Di Zheng and Chang Liu, culture, Zhi-Kang Zhang, CS18-26.

**Notes:** This species is a sister of *P. sanshaense* with strong support (BP = 100, PP = 1.00, Figure 7). It differs from the latter in 20 bp for BenA and 26 bp for CaM. Morphologically, it differs in faster growth rate on CYA at 25 °C (27–29 vs. 21–23 mm), yellow brown instead of reddish brown on YES reverse, shorter stipes (110–245 vs. 200–500 μm) and subglobose and rough-walled conidia [42].

**Figure 38:**
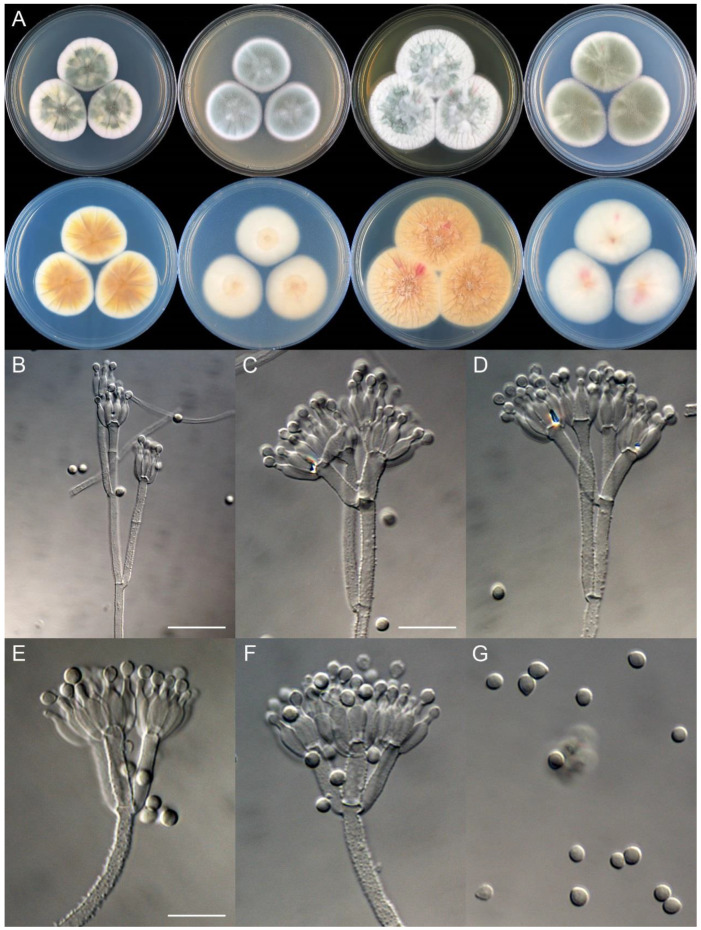
*Penicillium subrutilans* (CS20-14). (**A**) Colonies: top row left to right, obverse CYA, MEA, YES, and PDA; bottom row left to right, reverse CYA, MEA, YES, and PDA; (**B**–**F**) Conidiophores; (**G**) Conidia. Bars: (**B**) = 20 µm; (**C**) = 12.5 µm, also for (**D**); (**E**) = 10 µm, also for (**F**,**G**).

***Penicillium subrutilans*** X.C. Wang & W.Y. Zhuang, sp. nov. Figure 38.

**Fungal Names:** FN571562.

**Etymology:** The specific epithet refers to the pink patches on reverse of PDA.

In *Penicillium* subgenus *Aspergilloides* section *Lanata-Divaricata* series *Rolfsiorum*.

**Typification:** China. Chongqing City, Wushan County, Wulipo National Nature Reserve, 31°22′59″N 109°56′11″E, in soil, October 29 2020, Xin-Cun Wang, Huan-Di Zheng and Chang Liu, culture, Zhi-Kang Zhang, CS20-14 (holotype HMAS 247913, ex-type strain CGMCC 3.25174).

**DNA barcodes:** ITS OQ870816, BenA OR051137, CaM OR051314, RPB2 OR051479.

**Colony diam.**, 7 days, 25 °C (unless stated otherwise): CYA 34–37 mm; CYA 37 °C no growth; CYA 5 °C no growth; MEA 35–36 mm; YES 45–47 mm; PDA 40–41 mm.

**Colony characteristics:** On CYA 25 °C, 7 days: Colonies nearly circular, radially sulcate; margins wide, entire; mycelia white; texture velutinous; sporulation moderately dense; conidia *en masse* greyish green; soluble pigments absent; exudates hyaline, clear; reverse buff, with light brown radiate branches, whitish at margins.

On MEA 25 °C, 7 days: Colonies nearly circular, slightly protuberant at centers; margins narrow to moderately wide, entire; mycelia white; texture velutinous; sporulation dense; conidia *en masse* greyish green; soluble pigments absent; exudates absent; reverse whitish.

On YES 25 °C, 7 days: Colonies nearly circular, strongly sulcate, protuberant at centers; margins wide, fimbriate; mycelia white; texture velutinous; sporulation dense; conidia *en masse* greenish grey; soluble pigments absent; exudates absent; reverse buff, with interwoven light brown cracks.

On PDA 25 °C, 7 days: Colonies nearly circular, with light-colored sectors; margins moderately wide, entire; mycelia white; texture velutinous; sporulation dense; conidia *en masse* greyish green; soluble pigments absent; exudates absent; reverse white, with pinkish patches.

**Micromorphology:** Conidiophores terverticillate or biverticillate; stipes strongly rough-walled, 160–375 × 2.5–3.5 μm; rami 2, 22–31 × 3.0–3.5 μm; metulae 2–4, 11–23 × 2.5–4.5 μm; phialides ampulliform to acerose, tapering into very thin neck, 5–8 per metula, 8.5–11.5 × 2.5–3.5 μm; conidia subglobose, smooth-walled, 2.5–4.0 μm.

**Additional strains examined:** China. Chongqing City, Wushan County, Wulipo National Nature Reserve, 31°22′59″ N 109°56′11″ E, in soil, 29 October 2020, Xin-Cun Wang, Huan-Di Zheng and Chang Liu, culture, Zhi-Kang Zhang, CS20-27; *ibid.*, CS20-58.

**Notes:** This species is a sister to *P. camponotum* with strong support (BP = 100, PP = 1.00, Figure 6). It differs from the latter in three bp for BenA, nine bp for CaM and nine bp for RPB2. Morphologically, it differs in slower growth rate on MEA (35–36 vs. 48–55 mm) and YES (45–47 vs. 53–60 mm) at 25 °C, shorter stipes (160–375 vs. 220–620 μm) and smooth-walled conidia [67].

**Figure 39:**
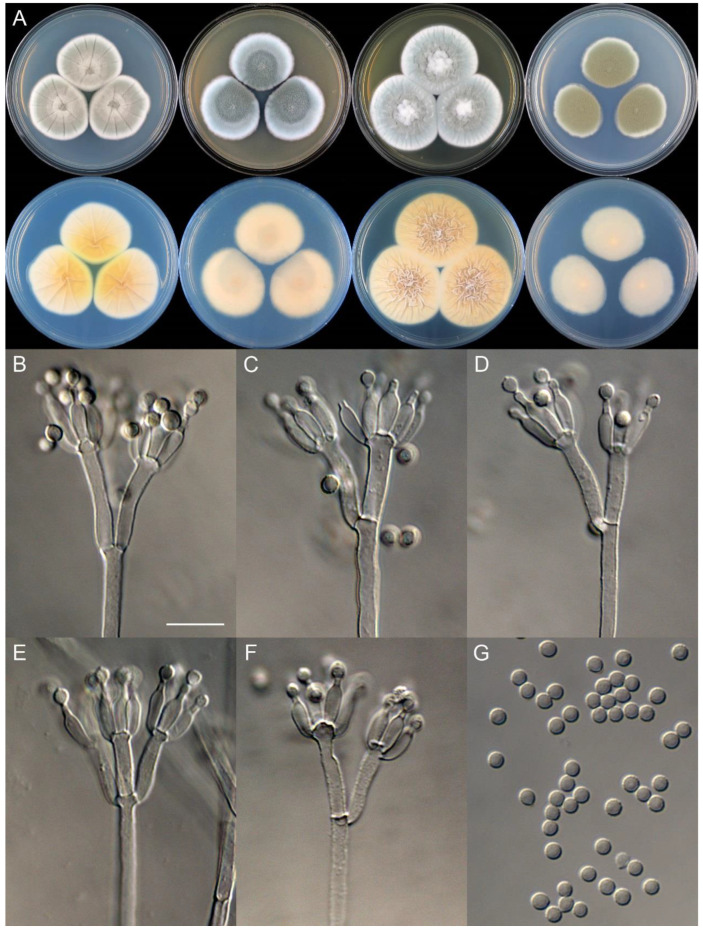
*Penicillium taii* (CS16-09). (**A**) Colonies: top row left to right, obverse CYA, MEA, YES, and PDA; bottom row left to right, reverse CYA, MEA, YES, and PDA; (**B**–**F**) Conidiophores; (**G**) Conidia. Bar: (**B**) = 10 µm, also for (**C**–**G**).

***Penicillium taii*** X.C. Wang & W.Y. Zhuang, sp. nov. Figure 39.

**Fungal Names:** FN571564.

**Etymology:** The specific epithet is named after the late distinguished mycologist Professor Fang-Lan Tai (1893.05–1973.01), one of the founders of Mycology and Plant Pathology of China.

In *Penicillium* subgenus *Aspergilloides* section *Lanata-Divaricata* series *Simplicissima*.

**Typification:** China. Chongqing City, Fengjie County, Caotang Town, 31°5′29″ N 109°38′57″ E, in soil, 29 October 2020, Xin-Cun Wang, Huan-Di Zheng and Chang Liu, culture, Zhi-Kang Zhang, CS16-09 (holotype HMAS 247915, ex-type strain CGMCC 3.25176).

**DNA barcodes:** ITS OQ870778, BenA OR051170, CaM OR051347, RPB2 OR051496.

**Colony diam.**, 7 days, 25 °C (unless stated otherwise): CYA 37–39 mm; CYA 37 °C no growth; CYA 5 °C no growth; MEA 37–39 mm; YES 44–46 mm; PDA 32–34 mm.

**Colony characteristics:** On CYA 25 °C, 7 days: Colonies nearly circular, concave at centers, radially sulcate; margins narrow, entire; mycelia white; texture velutinous; sporulation dense; conidia *en masse* light grey; soluble pigments absent; exudates hyaline, clear, tiny; reverse buff.

On MEA 25 °C, 7 days: Colonies nearly circular, slightly concave at central areas; margins narrow to moderately wide, entire; mycelia white; texture velutinous; sporulation dense; conidia *en masse* greyish green; soluble pigments absent; exudates absent; reverse whitish.

On YES 25 °C, 7 days: Colonies nearly circular, radially sulcate and strongly sulcate at centers, protuberant at centers; margins moderately wide, fimbriate; mycelia white; texture velutinous; sporulation dense; conidia *en masse* greenish grey; soluble pigments absent; exudates absent; reverse buff, with radiate branches.

On PDA 25 °C, 7 days: Colonies nearly circular or irregular, slightly protuberant at centers; margins narrow, entire or irregular; mycelia white; texture velutinous; sporulation dense; conidia *en masse* greyish green; soluble pigments absent; exudates absent; reverse whitish.

**Micromorphology:** Conidiophores biverticillate; stipes rough-walled, 175–500 × 2.5–3.0 μm; metulae 2–3, 11–20 × 2.5–5.5 μm; phialides ampulliform to acerose, tapering into very thin neck, 5–7 per metula, 7.5–10 × 2.5–3.0 μm; conidia subglobose, finely rough-walled, 3.0–3.5 μm.

**Additional strain examined:** China. Sichuan Province, Dazhou City, Xuanhan County, Bashan Grand Canyon, 31°39′44″ N 108°51′17″ E, in soil, 1 November 2020, Xin-Cun Wang, Huan-Di Zheng and Chang Liu, culture, Zhi-Kang Zhang, CS30-11.

**Notes:** This species is a sister to *P. globosum* and *P. yuyongnianii* (BP = 74, PP = 100, Figure 6). It differs from *P. globosum* in six bp for BenA, eight bp for CaM and seven bp for RPB2; and it differs from *P. yuyongnianii* in three bp for BenA, eight bp for CaM and five bp for RPB2. Morphologically, it grows faster than *P. globosum* on CYA (37–39 vs. 21–22 mm), MEA (37–39 vs. 21–24 mm) and YES (44–46 vs. 17–19 mm) at 25 °C, and no growth on CYA at 37 °C [43]; and it differs from *P. yuyongnianii* in fast growth rate on MEA (37–39 vs. 22–24 mm) and PDA (32–34 vs. 21–24 mm) at 25 °C, and subglobose and finely rough-walled conidia.

**Figure 40:**
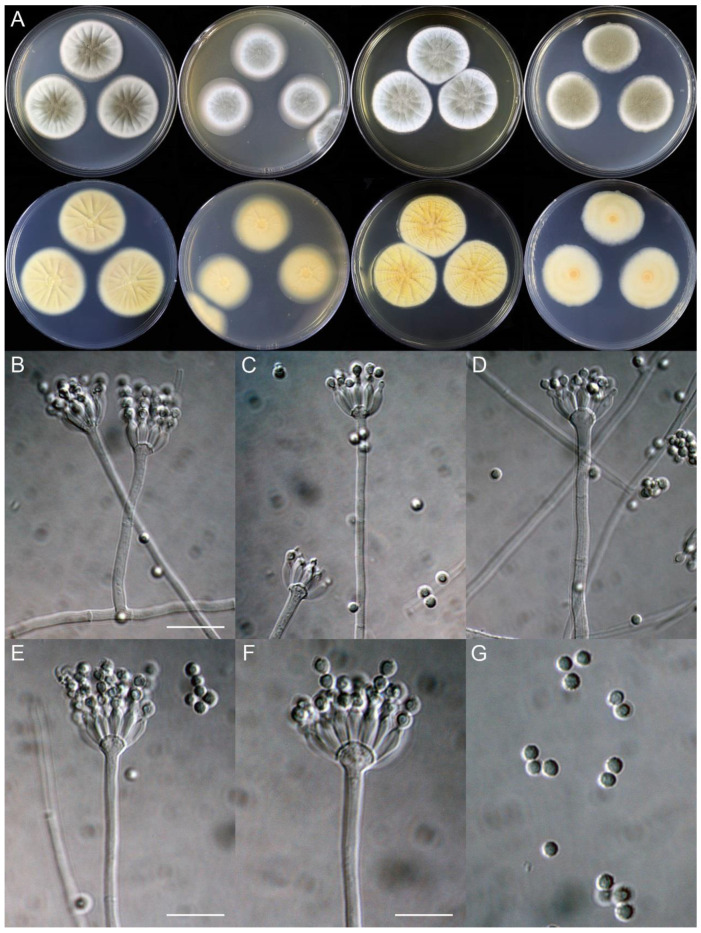
*Penicillium tangii* (CS04-07). (**A**) Colonies: top row left to right, obverse CYA, MEA, YES, and PDA; bottom row left to right, reverse CYA, MEA, YES, and PDA; (**B**–**F**) Conidiophores; (**G**) Conidia. Bars: (**B**) = 15 µm, also for (**C**,**D**); (**E**) = 12.5 µm; (**F**) = 10 µm, also for (**G**).

***Penicillium tangii*** X.C. Wang & W.Y. Zhuang, sp. nov. Figure 40.

**Fungal Names:** FN571565.

**Etymology:** The specific epithet is named in memory of Chinese medical microbiologist Fei-Fan Tang (1897.07–1958.09). He is famous for performing the first isolation of *Chlamydia trachomatis*, and the first production of penicillin in China during World War II was also conducted by his team.

In *Penicillium* subgenus *Aspergilloides* section *Aspergilloides* series *Spinulosa*.

**Typification:** China. Chongqing City, Jiangjin District, Simian Mountain Nature Reserve, 28°35′57″ N 106°26′51″ E, in soil of ant hole, 24 October 2020, Chang Liu, Zhao-Qing Zeng, Xin-Cun Wang and Huan-Di Zheng, culture, Zhi-Kang Zhang, CS04-07 (holotype HMAS 247916, ex-type strain CGMCC 3.25177).

**DNA barcodes:** ITS OQ870808, BenA OR051069, CaM OR051248, RPB2 OR051421.

**Colony diam.**, 7 days, 25 °C (unless stated otherwise): CYA 36–37 mm; CYA 37 °C no growth; CYA 5 °C 2–4 mm; MEA 31–37 mm; YES 36–37 mm; PDA 37–38 mm.

**Colony characteristics:** On CYA 25 °C, 7 days: Colonies nearly circular, sulcate and slightly protuberant at centers; margins narrow to moderately wide, entire; mycelia white; texture velutinous; sporulation dense; conidia *en masse* bluish grey to dull green; soluble pigments absent; exudates tiny, clear; reverse cream.

On MEA 25 °C, 7 days: Colonies nearly circular, plain; margins moderately wide to wide, entire; mycelia white; texture velutinous; sporulation dense; conidia *en masse* bluish grey to dull green; soluble pigments absent; exudates absent; reverse cream to buff.

On YES 25 °C, 7 days: Colonies nearly circular, strongly sulcate, protuberant at centers; margins moderately narrow, fimbriate; mycelia white; texture velutinous, but floccose at centers; sporulation dense; conidia *en masse* bluish grey to dull green; soluble pigments absent; exudates absent; reverse buff, with radiate branches.

On PDA 25 °C, 7 days: Colonies nearly circular, plain; margins moderately wide, irregular; mycelia white; texture velutinous; sporulation dense; conidia *en masse* brownish green; soluble pigments absent; exudates absent; reverse cream to buff.

**Micromorphology:** Conidiophores monoverticillate; stipes smooth to slightly rough-walled, 50–337.5 × 2.0–3.5 μm; phialides ampulliform, tapering into very thin neck, 6–14, 7.5–9.0 × 2.5–3.5 μm; conidia subglobose, rough-walled, 2.5–3.5 μm.

**Notes:** This species is a sister to *P. subspinulosum* with strong support (BP = 91, PP = 0.97, Figure 2). It differs the latter in three bp for BenA, eight bp for CaM and three bp for RPB2. Morphologically, it differs in shorter stipe (50–337.5 vs. 200–400 μm) [59].

**Figure 41:**
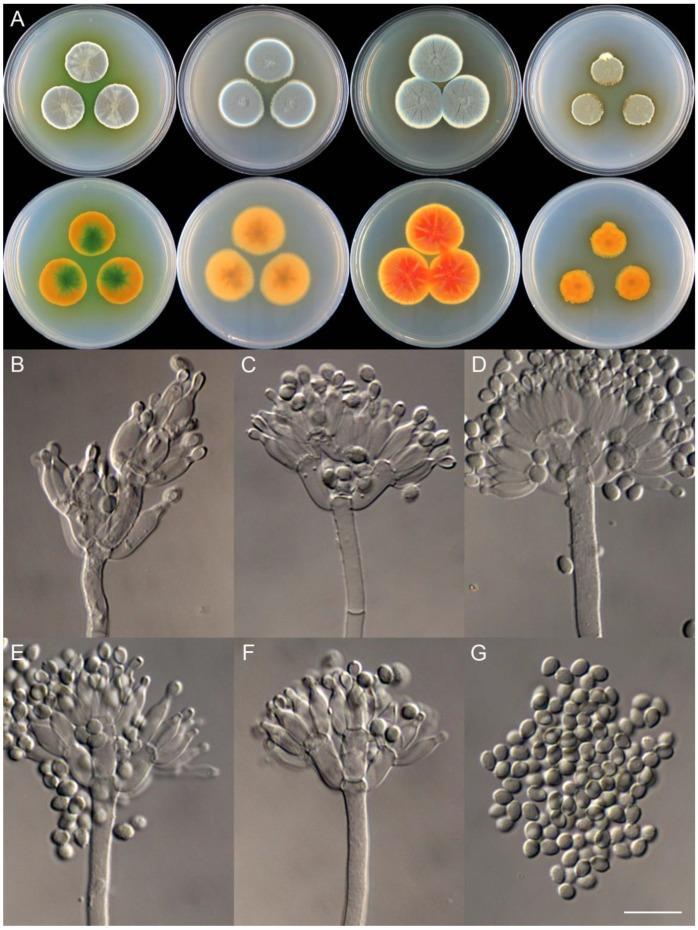
*Penicillium tardicrescens* (CS14-24). (**A**) Colonies: top row left to right, obverse CYA, MEA, YES, and PDA; bottom row left to right, reverse CYA, MEA, YES, and PDA; (**B**–**F**) Conidiophores; (**G**) Conidia. Bar: (**G**) = 10 µm, also for (**B**–**F**).

***Penicillium tardicrescens*** X.C. Wang & W.Y. Zhuang, sp. nov. Figure 41.

**Fungal Names:** FN571566.

**Etymology:** The specific epithet refers to the slow growth rate on PDA.

In *Penicillium* subgenus *Aspergilloides* section *Sclerotiorum* series *Herqueorum*.

**Typification:** China. Chongqing City, Fengjie County, Caotang Town, 31°5′29″ N 109°38′57″ E, in soil, 29 October 2020, Xin-Cun Wang, Huan-Di Zheng and Chang Liu, culture, Zhi-Kang Zhang, CS14-24 (holotype HMAS 247917, ex-type strain CGMCC 3.25178).

**DNA barcodes:** ITS OQ870853, BenA OR051202, CaM OR051377, RPB2 OR062067.

**Colony diam.**, 7 days, 25 °C (unless stated otherwise): CYA 24–25 mm; CYA 37 °C no growth; CYA 5 °C no growth; MEA 27–28 mm; YES 30–31 mm; PDA 18–19 mm.

**Colony characteristics:** On CYA 25 °C, 7 days: Colonies nearly circular, protuberant at centers, radially sulcate, with sectors; margins narrow, entire; mycelia yellow; texture velutinous; sporulation dense; conidia *en masse* dull green; soluble pigments greenish yellow; exudates absent; reverse brownish orange, with dark green patches.

On MEA 25 °C, 7 days: Colonies nearly circular, protuberant at centers; margins narrow, entire; mycelia yellow; texture velutinous; sporulation dense; conidia *en masse* dull green; soluble pigments absent; exudates absent; reverse light brownish orange, with brownish patches at centers.

On YES 25 °C, 7 days: Colonies nearly circular, radially sulcate, concave at centers; margins narrow, undulated; mycelia yellow; texture velutinous; sporulation dense; conidia *en masse* dull green; soluble pigments yellow; exudates absent; reverse orange, yellow at margins, with red patches at centers.

On PDA 25 °C, 7 days: Colonies nearly circular or irregular, slightly protuberant at centers; margins narrow, entire or irregular; mycelia yellow; texture velutinous; sporulation dense; conidia *en masse* dull green; soluble pigments yellow brown; exudates absent; reverse light brownish orange, slightly brownish at centers.

**Micromorphology:** Conidiophores biverticillate, occasionally terverticillate; stipes smooth- to finely rough-walled, 200–350 × 2.5–4.5 μm; metulae 4–6, 10–12.5 × 3.5–5.5 μm; phialides ampulliform, tapering into very thin neck, 5–6 per metula, 9–11 × 3.0–4.0 μm; conidia ellipsoidal to broad fusiform, finely rough-walled, 3.0–4.0 × 2.5–3.5 μm.

**Notes:** This species is a distinct lineage in ser. *Herqueorum* and seems to have close relationship with *P. malachiteum* (Figure 7). It differs from the latter in 35 bp for BenA, 60 bp for CaM and 42 bp for RPB2. Morphologically, it differs in lacking of sclerotia on the media [61].

**Figure 42:**
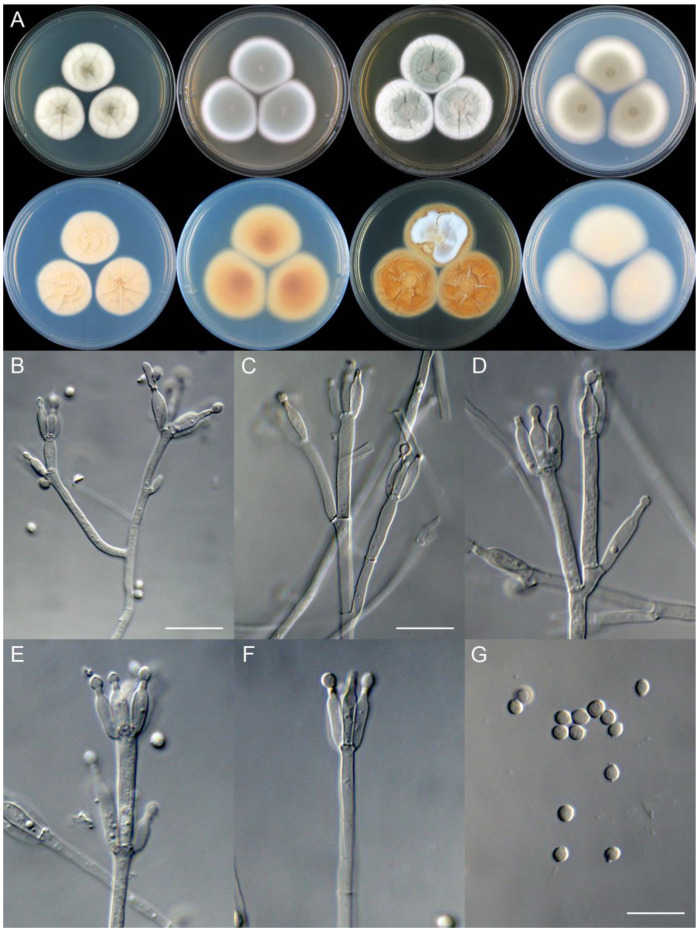
*Penicillium tengii* (CS27-03). (**A**) Colonies: top row left to right, obverse CYA, MEA, YES, and PDA; bottom row left to right, reverse CYA, MEA, YES, and PDA; (**B**–**F**) Conidiophores; (**G**) Conidia. Bars: (**B**) = 15 µm; (**C**) = 12.5 µm; (**G**) = 10 µm, also for (**D**–**F**).

***Penicillium tengii*** X.C. Wang & W.Y. Zhuang, sp. nov. Figure 42.

**Fungal Names:** FN571567.

**Etymology:** The specific epithet is named after the late distinguished mycologist and plant pathologist Professor Shu-Chun Teng (1902.12–1970.05), one of the founders of Mycology and Forest Pathology of China.

In *Penicillium* subgenus *Aspergilloides* section *Lanata-Divaricata* series *Janthinella*.

**Typification:** China. Chongqing City, Chengkou County, Gaoguan Town, Donghong Village, 31°47′11″ N 108°59′29″ E, in soil, 30 October 2020, Xin-Cun Wang, Huan-Di Zheng and Chang Liu, culture, Zhi-Kang Zhang, CS27-03 (holotype HMAS 247918, ex-type strain CGMCC 3.25179).

**DNA barcodes:** ITS OQ870735, BenA OR051120, CaM OR051297, RPB2 OR051465.

**Colony diam.**, 7 days, 25 °C (unless stated otherwise): CYA 31–32 mm; CYA 37 °C 10–12 mm; CYA 5 °C no growth; MEA 38–40 mm; YES 38–39 mm; PDA 41–42 mm.

**Colony characteristics:** On CYA 25 °C, 7 days: Colonies nearly circular, concave at centers, radially and concentrically sulcate; margins wide, entire; mycelia white; texture velutinous; sporulation sparse; conidia *en masse* greyish brown; soluble pigments absent; exudates hyaline, clear; reverse cream to buff.

On CYA 37 °C, 7 days: Colonies nearly circular, papillate at centers; margins wide, entire; mycelia white; texture velutinous; sporulation absent; soluble pigments absent; exudates absent; reverse buff.

On MEA 25 °C, 7 days: Colonies nearly circular, plain, slightly protuberant at centers; margins moderately wide, entire; mycelia white; texture velutinous; sporulation dense; conidia *en masse* greyish green; soluble pigments absent; exudates absent; reverse buff, light brown at centers.

On YES 25 °C, 7 days: Colonies nearly circular, strongly sulcate, concave at centers; margins wide, entire; mycelia white; texture velutinous; sporulation moderately dense; conidia *en masse* light grey; soluble pigments absent; exudates absent; reverse buff to light brown.

On PDA 25 °C, 7 days: Colonies nearly circular, plain, slightly protuberant at centers; margins wide, entire; mycelia white; texture velutinous; sporulation dense; conidia *en masse* greyish green; soluble pigments absent; exudates absent; reverse white.

**Micromorphology:** Conidiophores terverticillate, biverticillate or monoverticillate; stipes smooth-walled, 100–425 × 1.5–3.0 μm; rami 2, 17.5–30 × 2.0–3.0 μm; metulae 2–4, 10–29 × 2.0–3.5 μm; phialides ampulliform to acerose, tapering into very thin neck, 4–6 per metula, 8.5–14.5 × 2.0–3.5 μm; conidia ellipsoidal, smooth-walled, 3.0–4.0 × 2.5–3.5 μm.

**Notes:** This species is a sister of *P. koreense* with strong support (BP = 100, PP = 1.00, Figure 6). It differs from the latter in 11 bp for BenA, 11 bp for CaM and five bp for RPB2. Morphologically, it differs in slower growth rate (10–12 vs. 15–19 mm) on CYA at 37 °C, shorter stipes (100–425 vs. 200–800 μm) and fewer phialides per metula (4–6 vs. 6–10) [72].

**Figure 43:**
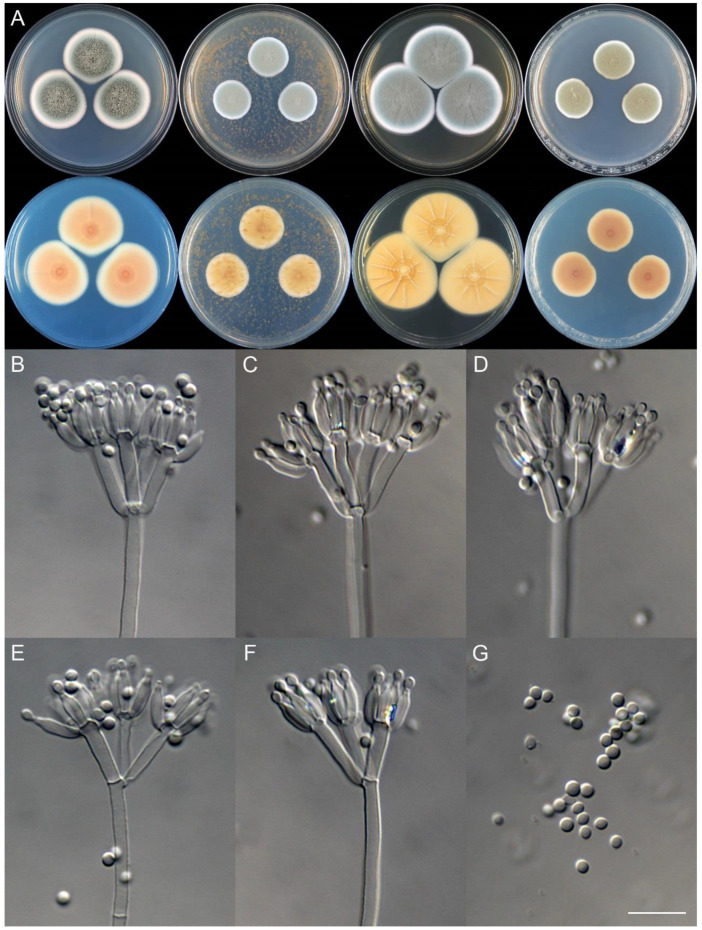
*Penicillium vulgatum* (CS15-03). (**A**) Colonies: top row left to right, obverse CYA, MEA, YES, and PDA; bottom row left to right, reverse CYA, MEA, YES, and PDA; (**B**–**F**) Conidiophores; (**G**) Conidia. Bar: (**G**) = 10 µm, also for (**B**–**F**).

***Penicillium vulgatum*** X.C. Wang & W.Y. Zhuang, sp. nov. Figure 43.

**Fungal Names:** FN571568.

**Etymology:** The specific epithet refers to its typical morphology in this series: conidia *en masse* bluish green to dull green, conidiophores biverticillate, conidia subglobose to ellipsoidal, smooth-walled to finely roughened.

In *Penicillium* subgenus *Aspergilloides* section *Citrina* series *Sumatraensia*.

**Typification:** China. Chongqing City, Fengjie County, Caotang Town, 31°5′29″ N 109°38′57″ E, in soil, 29 October 2020, Xin-Cun Wang, Huan-Di Zheng and Chang Liu, culture, Zhi-Kang Zhang, CS15-03 (holotype HMAS 247919, ex-type strain CGMCC 3.25180).

**DNA barcodes:** ITS OQ870884, BenA OR051086, CaM OR051263, RPB2 OR051434.

**Colony diam.**, 7 days, 25 °C (unless stated otherwise): CYA 34–35 mm; CYA 37 °C no growth; CYA 5 °C no growth; MEA 23–24 mm; YES 42–43 mm; PDA 23–25 mm.

**Colony characteristics:** On CYA 25 °C, 7 days: Colonies nearly circular, slightly sulcate, protuberant at centers; margins moderately wide, entire; mycelia white; texture velutinous; sporulation dense; conidia *en masse* viridian green; soluble pigments absent; exudates hyaline, clear, massive; reverse buff to pale brown.

On MEA 25 °C, 7 days: Colonies nearly circular, plain, protuberant at centers; margins narrow, entire; mycelia white; texture velutinous; sporulation dense; conidia *en masse* brownish green; soluble pigments absent; exudates absent; reverse buff.

On YES 25 °C, 7 days: Colonies nearly circular, radially sulcate, deep; margins moderately wide, entire; mycelia white; texture velutinous; sporulation dense; conidia *en masse* dull green; soluble pigments absent; exudates absent; reverse buff to yellow brown.

On PDA 25 °C, 7 days: Colonies irregular, protuberant at centers; margins narrow, entire; mycelia white; texture velutinous; sporulation dense; conidia *en masse* brownish green; soluble pigments absent; exudates absent; reverse light cinnamon brown.

**Micromorphology:** Conidiophores biverticillate; stipes smooth-walled, 175–425 × 2.5–3.0 μm; metulae 3–4, 12–16 × 2.5–3.0 μm; phialides ampulliform to acerose, tapering into very thin neck, 5–7 per metula, 7–9 × 2.0–2.5 μm; conidia subglobose to ellipsoidal, smooth-walled, 2.5–3.0 × 2.0–2.8 μm.

**Notes:** This species is closely related to *P. jenningsiae* and *P. rarum* in the phylogenetic tree (Figure 3). It differs from *P. jenningsiae* in 8 bp for BenA, 13 bp for CaM and 18 bp for RPB2, and differs from *P. rarum* in 7 bp for BenA, 8 bp for CaM and 34 bp for RPB2. Morphologically, this species differs from *P. rarum* in lacking terverticillate or monoverticillate conidiophores; and differs from *P. jenningsiae* in longer stipes (175–425 vs. 100–250 μm) [30].

**Figure 44:**
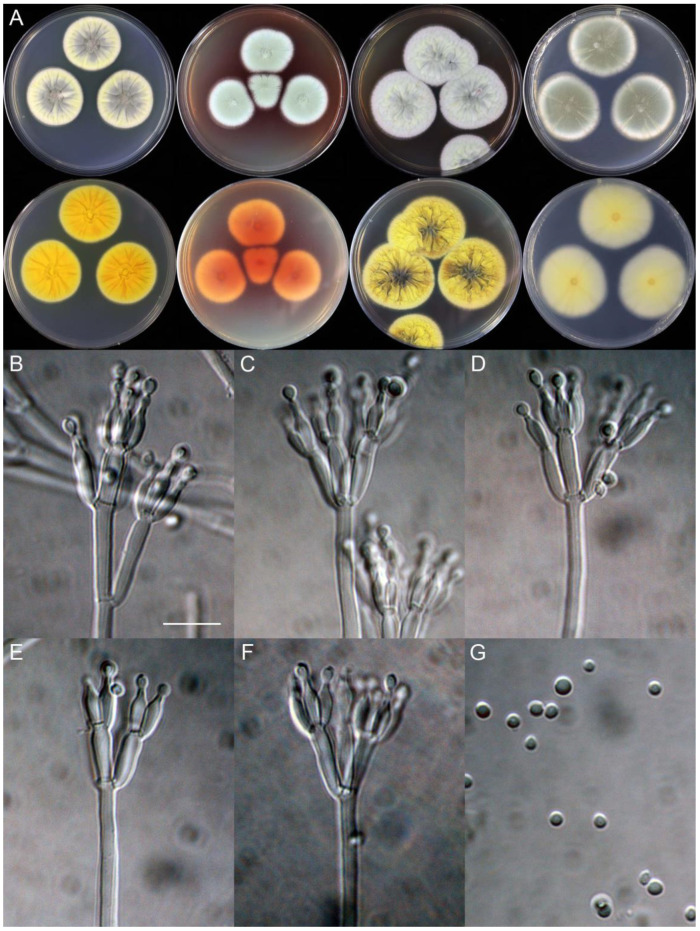
*Penicillium wangwentsaii* (CS20-42). (**A**) Colonies: top row left to right, obverse CYA, MEA, YES, and PDA; bottom row left to right, reverse CYA, MEA, YES, and PDA; (**B**–**F**) Conidiophores; (**G**) Conidia. Bar: (**B**) = 10 µm, also for (**C**–**G**).

***Penicillium wangwentsaii*** X.C. Wang & W.Y. Zhuang, sp. nov. Figure 44.

**Fungal Names:** FN571569.

**Etymology:** The specific epithet is in memory of the late Chinese distinguished plant taxonomist Wen-Tsai Wang (1926.06–2022.11). He is a leading taxonomic authority on several difficult plant families in China, including Boraginaceae, Gesneriaceae, Ranunculaceae, Rubiaceae, Urticaceae and Vitaceae, and described 28 new genera, 303 new taxa at the tribal, sectional, and series ranks, ca. 1370 new species, and 242 new combinations.

In *Penicillium* subgenus *Aspergilloides* section *Citrina* series *Westlingiorum*.

**Typification:** China. Chongqing City, Wushan County, Wulipo National Nature Reserve, 31°22′59″ N 109°56′11″ E, in soil, 29 October 2020, Xin-Cun Wang, Huan-Di Zheng and Chang Liu, culture, Zhi-Kang Zhang, CS20-42 (holotype HMAS 247920, ex-type strain CGMCC 3.25181).

**DNA barcodes:** ITS OQ870887, BenA OR051089, CaM OR051266, RPB2 OR051437.

**Colony diam.**, 7 days, 25 °C (unless stated otherwise): CYA 33–37 mm; CYA 37 °C no growth; CYA 5 °C 3–4 mm; MEA 28–37 mm; YES 39–47 mm; PDA 39–40 mm.

**Colony characteristics:** On CYA 25 °C, 7 days: Colonies nearly circular, protuberant at centers, radially sulcate; margins narrow, fimbriate; mycelia white and yellow; texture velutinous, but floccose at centers; sporulation dense; conidia *en masse* bluish grey to dull green; soluble pigments yellow; exudates absent; reverse bright yellow, with light brown radiate branches.

On MEA 25 °C, 7 days: Colonies nearly circular, plain, protuberant at centers; margins narrow to moderately wide, fimbriate; mycelia white; texture velutinous, but floccose at centers; sporulation dense; conidia *en masse* dull green; soluble pigments pink; exudates absent; reverse reddish.

On YES 25 °C, 7 days: Colonies nearly circular, strongly sulcate; margins narrow, fimbriate; mycelia white and yellow; texture velutinous; sporulation dense; conidia *en masse* greyish green; soluble pigments pink; exudates absent; reverse yellow, with blackish radiate branches at centers.

On PDA 25 °C, 7 days: Colonies nearly circular, plain, slightly protuberant at centers; margins moderately wide, entire; mycelia white; texture velutinous; sporulation dense; conidia *en masse* dull green; soluble pigments absent; exudates absent; reverse cream to light yellow.

**Micromorphology:** Conidiophores biverticillate, occasionally terverticillate; stipes smooth-walled, 90–350 × 2.5–3.0 μm; rami 2, 17–18 × 2.5–3.0 μm; metulae 3–5, 10–16 × 2.0–4.0 μm; phialides ampulliform, tapering into very thin neck, 4–6, 8.0–13 × 2.5–3.0 μm; conidia subglobose to obovate, smooth-walled, 2.5–3.5 μm.

**Notes:** This species is a sister to *P. cairnsense* with strong support (BP = 82, PP = 1.00, Figure 3). It differs from the latter in one bp for BenA, one bp for CaM and 19 bp for RPB2. Morphologically, it differs in occasionally terverticillate conidiophores instead of a large portion terverticillate ones, shorter stipes (90–350 vs. 200–400 μm) and longer phialides (8–13 vs. 7–9 μm) [73].

**Figure 45:**
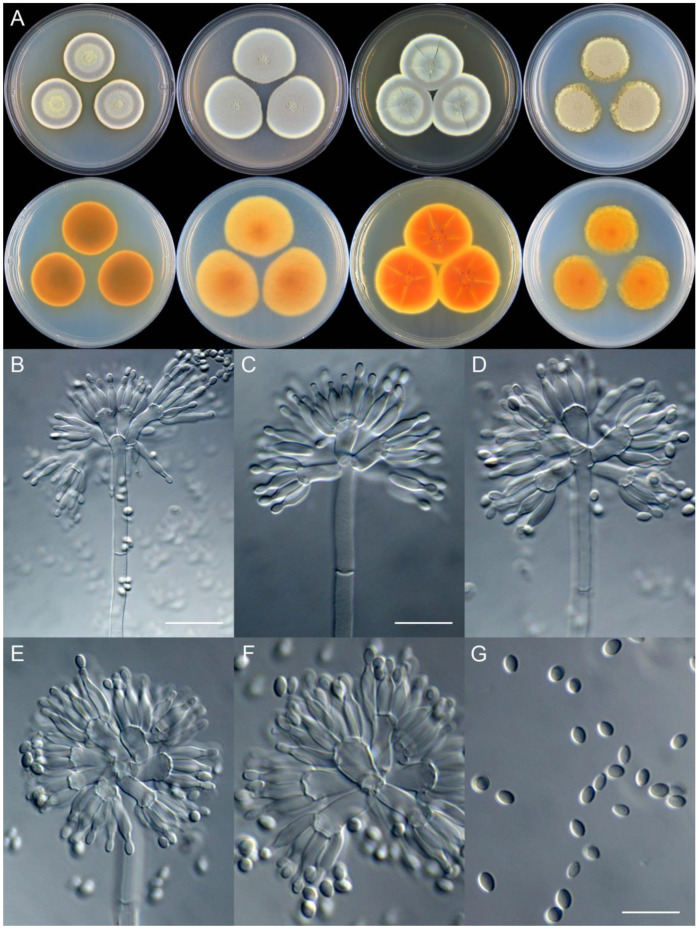
*Penicillium wanyuanense* (CS33-06). (**A**) Colonies: top row left to right, obverse CYA, MEA, YES, and PDA; bottom row left to right, reverse CYA, MEA, YES, and PDA; (**B**–**F**) Conidiophores; (**G**) Conidia. Bars: (**B**) = 20 µm; (**C**) = 12.5 µm, also for (**D**,**E**); (**G**) = 10 µm, also for (**F**).

***Penicillium wanyuanense*** X.C. Wang & W.Y. Zhuang, sp. nov. Figure 45.

**Fungal Names:** FN571570.

**Etymology:** The specific epithet refers to the type locality.

In *Penicillium* subgenus *Aspergilloides* section *Sclerotiorum* series *Herqueorum*.

**Typification:** China. Sichuan Province, Dazhou City, Wanyuan City, Longtanhe, 31°50′19″ N 108°19′15″ E, in soil, 1 November 2020, Xin-Cun Wang, Huan-Di Zheng and Chang Liu, culture, Zhi-Kang Zhang, CS33-06 (holotype HMAS 247921, ex-type strain CGMCC 3.25182).

**DNA barcodes:** ITS OQ870854, BenA OR051203, CaM OR051378, RPB2 OR062068.

**Colony diam.**, 7 days, 25 °C (unless stated otherwise): CYA 28–30 mm; CYA 37 °C no growth; CYA 5 °C no growth; MEA 36–38 mm; YES 36–38 mm; PDA 27–30 mm.

**Colony characteristics:** On CYA 25 °C, 7 days: Colonies nearly circular, protuberant at centers; margins narrow, entire; mycelia yellow; texture velutinous; sporulation dense; conidia *en masse* yellowish green at central areas, greyish purple at periphery; soluble pigments yellow brown; exudates hyaline, clear; reverse dirty orange, with a brown tint at centers.

On MEA 25 °C, 7 days: Colonies nearly circular, funiculose at centers; margins moderately wide, entire; mycelia yellow; texture velutinous; sporulation dense; conidia *en masse* dull green; soluble pigments absent; exudates absent; reverse buff, slightly orange brown at centers.

On YES 25 °C, 7 days: Colonies nearly circular, radially sulcate; margins narrow to moderately wide, entire or undulated; mycelia white and yellow; texture velutinous; sporulation dense; conidia *en masse* yellowish green at central areas, dull green at periphery; soluble pigments light brown; exudates absent; reverse orange, yellow at margins.

On PDA 25 °C, 7 days: Colonies irregular, plain; margins narrow, irregular; mycelia yellow; texture velutinous; sporulation dense; conidia *en masse* dull green; soluble pigments light brown; exudates absent; reverse light dirty orange, orange at centers.

**Micromorphology:** Conidiophores biverticillate, occasionally terverticillate; stipes smooth to finely rough-walled, 235–275 × 3.5–4.5 μm; metulae 7–8, 10–12.5 × 3.5–5.5 μm; phialides ampulliform to acerose, tapering into very thin neck, 6–8 per metula, 7.5–10 × 2.5–3.5 μm; conidia ellipsoidal to broad fusiform, smooth-walled, 3.0–4.5 × 2.5–3.5 μm.

**Additional strain examined:** China. Sichuan Province, Dazhou City, Wanyuan City, Longtanhe, 31°50′19″ N 108°19′15″ E, in soil, 1 November 2020, Xin-Cun Wang, Huan-Di Zheng and Chang Liu, culture, Zhi-Kang Zhang, CS33-09.

**Notes:** This species is closely related to *P. herquei* (Figure 7). It differs from the ex-type strain of the latter species in 13 bp for BenA, 26 bp for CaM and 21 bp for RPB2. Because of the wide species concept held in the previous literatures [61,62], the morphological difference between them was obscure. The greyish purple conidia *en masse* on CYA makes this species different from the traditional concept of *P. herquei*.

**Figure 46:**
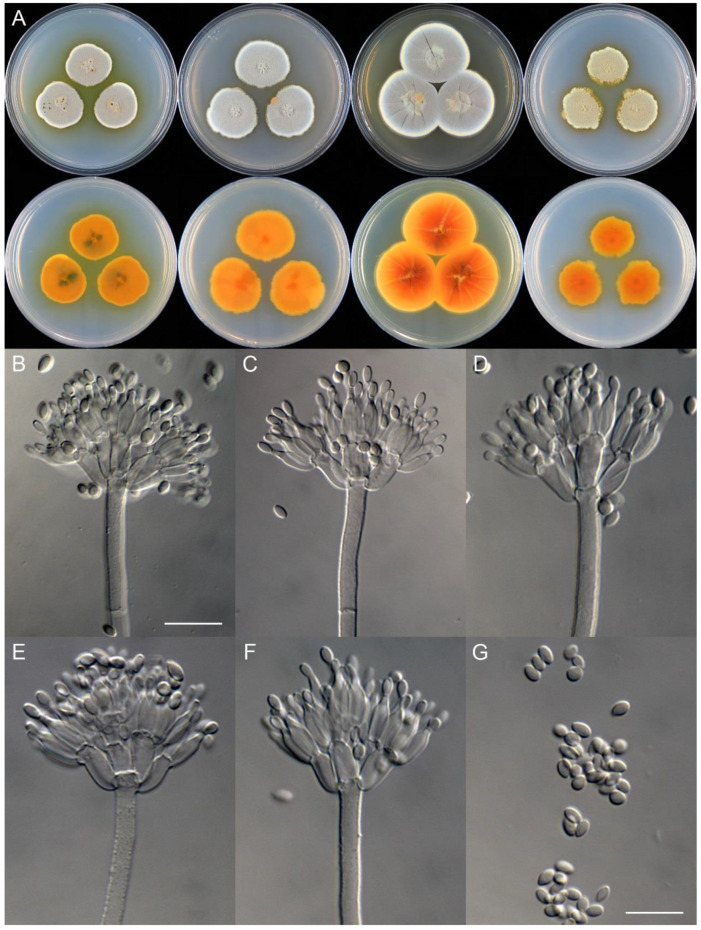
*Penicillium wulientehii* (CS32-02). (**A**) Colonies: top row left to right, obverse CYA, MEA, YES, and PDA; bottom row left to right, reverse CYA, MEA, YES, and PDA; (**B**–**F**) Conidiophores; (**G**) Conidia. Bars: (**B**) = 12.5 µm, also for (**C**); (**G**) = 10 µm, also for (**D**–**F**).

***Penicillium wulientehii*** X.C. Wang & W.Y. Zhuang, sp. nov. Figure 46.

**Fungal Names:** FN571571.

**Etymology:** The specific epithet is in memory of the late epidemiologist Lien-teh Wu (1879.03–1960.01), who pioneered the use of face mask and defeated a plague epidemic in northeastern China during 1910s.

In *Penicillium* subgenus *Aspergilloides* section *Sclerotiorum* series *Herqueorum*.

**Typification:** China. Sichuan Province, Dazhou City, Xuanhan County, Bashan Grand Canyon, 31°39′44″ N 108°51′17″ E, in soil of ant hole, 1 November 2020, Xin-Cun Wang, Huan-Di Zheng and Chang Liu, culture, Zhi-Kang Zhang, CS32-02 (holotype HMAS 247922, ex-type strain CGMCC 3.25183).

**DNA barcodes:** ITS OQ870856, BenA OR051205, CaM OR051380, RPB2 OR062070.

**Colony diam.**, 7 days, 25 °C (unless stated otherwise): CYA 26–27 mm; CYA 37 °C no growth; CYA 5 °C no growth; MEA 29–31 mm; YES 37–40 mm; PDA 24–26 mm.

**Colony characteristics:** On CYA 25 °C, 7 days: Colonies nearly circular or irregular, protuberant at centers; margins narrow, entire; mycelia yellow; texture velutinous; sporulation dense; conidia *en masse* dull green; soluble pigments greenish yellow; exudates brown and hyaline, clear; reverse dirty orange, with a few dark patches at centers.

On MEA 25 °C, 7 days: Colonies nearly circular or irregular, funiculose at centers; margins narrow, entire or irregular; mycelia yellow; texture velutinous; sporulation dense; conidia *en masse* dull green; soluble pigments absent; exudates absent; reverse orange, reddish at centers.

On YES 25 °C, 7 days: Colonies nearly circular, radially sulcate, concave at centers; margins narrow, undulated; mycelia white and yellow; texture velutinous; sporulation dense; conidia *en masse* yellowish green; soluble pigments yellow; exudates absent; reverse orange, yellow at margins, brownish at centers.

On PDA 25 °C, 7 days: Colonies nearly circular or irregular, funiculose at centers; margins narrow, entire or irregular; mycelia yellow; texture velutinous; sporulation dense; conidia *en masse* yellowish green; soluble pigments light brown; exudates absent; reverse orange with margins lighter.

**Micromorphology:** Conidiophores biverticillate; stipes finely rough-walled, 200–425 × 3.0–4.5 μm; metulae 6–7, 8–12.5 × 3.5–5.5 μm; phialides ampulliform, tapering into very thin neck, 6–8 per metula, 8–11 × 2.5–4.0 μm; conidia ellipsoidal to broad fusiform, smooth-walled, 3.0–4.5 × 2.0–3.0 μm.

**Notes:** This species is closely related to *P. creberum* and *P. flosculum* (BP = 100, PP = 1.00, Figure 7). It differs from *P. creberum* in 17 bp for BenA, 22 bp for CaM and 14 bp for RPB2; and differs from *P. flosculum* in 12 bp for BenA, 19 bp for CaM and 19 bp for RPB2. Morphologically, it differs from *P. creberum* in slower growth rate on MEA at 25 °C (29–31 mm vs. 34–36 mm), longer phialides (8–11 vs. 7.5–9 μm) and conidia (3.0–4.5 vs. 3.0–3.5 μm); it differs from *P. flosculum* in slower growth rates on CYA (26–27 mm vs. 29–31 mm), MEA (29–31 mm vs. 32–34 mm) and PDA (24–26 mm vs. 29–31 mm) and lacking of dark green radiate branches on CYA reverse. But it shows no morphological differences with the traditional concept of *P. herquei*.

**Figure 47:**
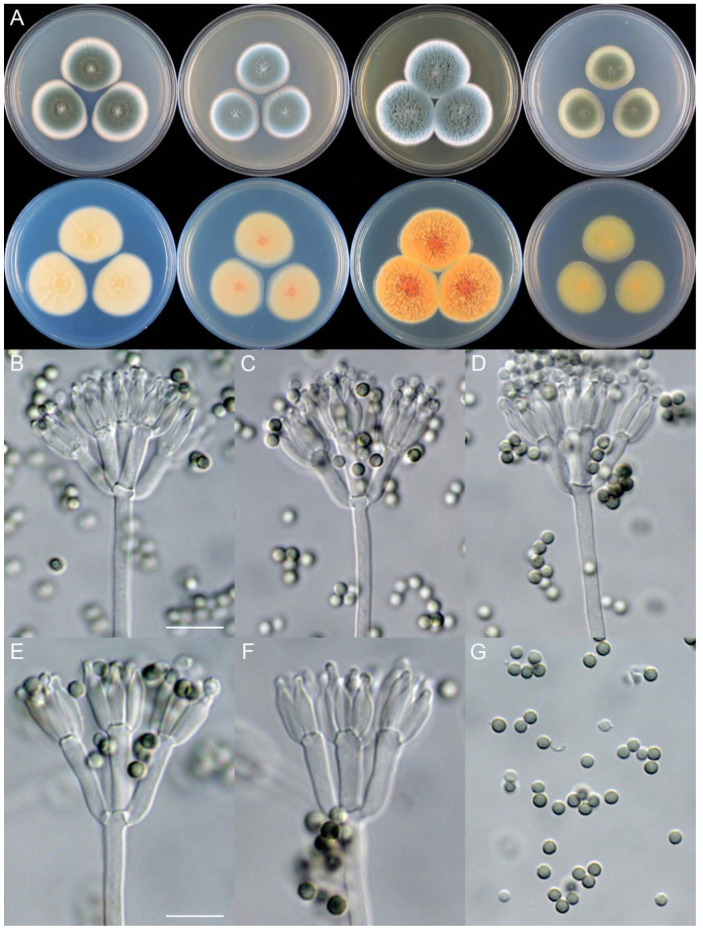
*Penicillium wushanicum* (CS21-01). (**A**) Colonies: top row left to right, obverse CYA, MEA, YES, and PDA; bottom row left to right, reverse CYA, MEA, YES, and PDA; (**B**–**F**) Conidiophores; (**G**) Conidia. Bars: (**B**) = 10 µm, also for (**C**,**D**,**G**); (**E**) = 7.5 µm, also for (**F**).

***Penicillium wushanicum*** X.C. Wang & W.Y. Zhuang, sp. nov. Figure 47.

**Fungal Names:** FN571572.

**Etymology:** The specific epithet refers to the type locality.

In *Penicillium* subgenus *Aspergilloides* section *Citrina* series *Westlingiorum*.

**Typification:** China. Chongqing City, Wushan County, Wulipo National Nature Reserve, 31°22′59″ N 109°56′11″ E, in soil, 29 October 2020, Xin-Cun Wang, Huan-Di Zheng and Chang Liu, culture, Zhi-Kang Zhang, CS21-01 (holotype HMAS 247923, ex-type strain CGMCC 3.25184).

**DNA barcodes:** ITS OQ870889, BenA OR051091, CaM OR051268, RPB2 OR051439.

**Colony diam.**, 7 days, 25 °C (unless stated otherwise): CYA 34–35 mm; CYA 37 °C no growth; CYA 5 °C no growth; MEA 32–34 mm; YES 39–40 mm; PDA 28–30 mm.

**Colony characteristics:** On CYA 25 °C, 7 days: Colonies nearly circular, radially and concentrically sulcate; margins moderately wide, entire; mycelia white; texture velutinous; sporulation dense; conidia *en masse* bluish green; soluble pigments absent; exudates absent; reverse buff.

On MEA 25 °C, 7 days: Colonies nearly circular, plain, funiculose at centers; margins moderately wide, entire; mycelia white; texture velutinous; sporulation dense; conidia *en masse* bluish green; soluble pigments absent; exudates absent; reverse cream to buff.

On YES 25 °C, 7 days: Colonies nearly circular, strongly sulcate; margins narrow to moderately wide, entire; mycelia white; texture velutinous; sporulation dense; conidia *en masse* bluish green; soluble pigments absent; exudates absent; reverse yellowish orange, with lignt brown cracks, orange at centers.

On PDA 25 °C, 7 days: Colonies nearly circular, plain, slightly protuberant at centers; margins wide, entire; mycelia white; texture velutinous; sporulation dense; conidia *en masse* dull green; soluble pigments absent; exudates absent; reverse dirty yellow.

**Micromorphology:** Conidiophores biverticillate; stipes smooth-walled, 200–525 × 2.0–3.0 μm; metulae 5, 11–13 × 2.5–3.5 μm; phialides acerose to ampulliform, tapering into very thin neck, 4–7 per metula, 8–10 × 2.5–3.0 μm; conidia subglobose, smooth- to slightly rough-walled, 2.5–3.0 μm.

**Notes:** This species is a sister of *P. raphiae* in the phylogenetic tree with strong support (BP = 100, PP = 100, Figure 3). It differs from the latter in seven bp for BenA, 19 bp for CaM and 18 bp for RPB2. Additionally, it has faster growth rate on MEA (32–34 vs. 21–25 mm) and YES (39–40 vs. 31–35 mm) at 25 °C, orange colonial centers on YES and slightly larger conidia (2.5–3.0 vs. 1.8–2.5 μm) [73].

**Figure 48:**
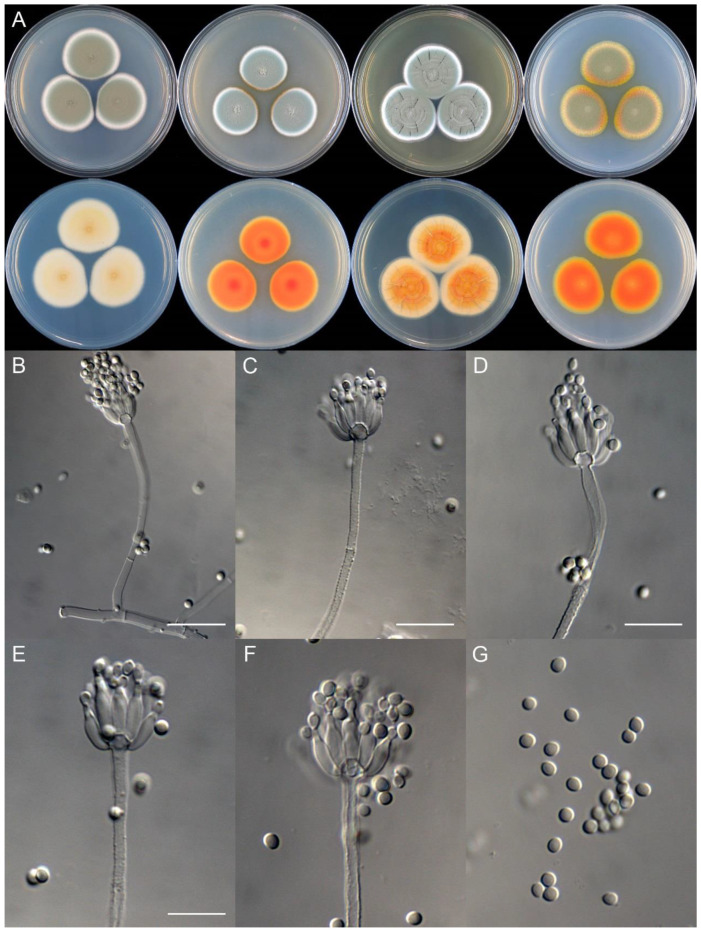
*Penicillium wuxiense* (CS25-12). (**A**) Colonies: top row left to right, obverse CYA, MEA, YES, and PDA; bottom row left to right, reverse CYA, MEA, YES, and PDA; (**B**–**F**) Conidiophores; (**G**) Conidia. Bars: (**B**) = 17.5 µm; (**C**) = 15 µm; (**D**) = 12.5 µm; (**E**) = 10 µm, also for (**F**,**G**).

***Penicillium wuxiense*** X.C. Wang & W.Y. Zhuang, sp. nov. Figure 48.

**Fungal Names:** FN571573.

**Etymology:** The specific epithet refers to the type locality.

In *Penicillium* subgenus *Aspergilloides* section *Sclerotiorum* series *Sclerotiorum*.

**Typification:** China. Chongqing City, Wuxi County, Hongchiba National Forest Park, 31°33′3″ N 109°1′36″ E, in soil, 30 October 2020, Xin-Cun Wang, Huan-Di Zheng and Chang Liu, culture, Zhi-Kang Zhang, CS25-12 (holotype HMAS 247924, ex-type strain CGMCC 3.25185).

**DNA barcodes:** ITS OQ870872, BenA OR051221, CaM OR051395, RPB2 OR062085.

**Colony diam.**, 7 days, 25 °C (unless stated otherwise): CYA 32–33 mm; CYA 37 °C no growth; CYA 5 °C no growth; MEA 27–28 mm; YES 34–35 mm; PDA 31–32 mm.

**Colony characteristics:** On CYA 25 °C, 7 days: Colonies nearly circular, protuberant at centers; margins moderately wide, entire; mycelia white; texture velutinous; sporulation dense; conidia *en masse* dull green; soluble pigments absent; exudates hyaline, clear; reverse cream to buff.

On MEA 25 °C, 7 days: Colonies nearly circular, funiculose at centers; margins narrow, entire; mycelia white; texture velutinous; sporulation dense; conidia *en masse* dull green; soluble pigments absent; exudates absent; reverse orange, reddish at centers.

On YES 25 °C, 7 days: Colonies nearly circular, radially and concentrically sulcate, concave at centers; margins narrow, undulated; mycelia white; texture velutinous; sporulation dense; conidia *en masse* dull green; soluble pigments absent; exudates absent; reverse buff to orange, cream at margins, with radiate branches.

On PDA 25 °C, 7 days: Colonies nearly circular, slightly protuberant at centers; margins moderately wide, entire; mycelia yellow; texture velutinous; sporulation dense; conidia *en masse* greyish green; soluble pigments absent; exudates absent; reverse raddish orange, lighter at centers and margins.

**Micromorphology:** Conidiophores monoverticillate; stipes smooth to rough-walled, 110–150 × 2.0–3.0 μm; phialides ampulliform to acerose, tapering into very thin neck, 8–12 per stipe, 10–11.5 × 2.5–3.5 μm; conidia subglobose to ellipsoidal, smooth-walled, 2.5–3.5 × 2.5–3.0 μm.

**Notes:** This species is a sister of *P. cainii* with strong support (BP = 100, PP = 1.00, Figure 7). It differs from the latter in 10 bp for BenA, 12 bp for CaM and 18 bp for RPB2. Morphologically, it differs in faster growth rate on CYA at 25 °C (32–33 vs. 23–29 mm), longer stipes (110–150 vs. 70–80 μm) and phialides (10–11.5 vs. 7.5–10 μm) and larger (2.5–3.5 × 2.5–3.0 vs. 2.0–2.5 μm in diam) and ellipsoidal conidia [64].

**Figure 49:**
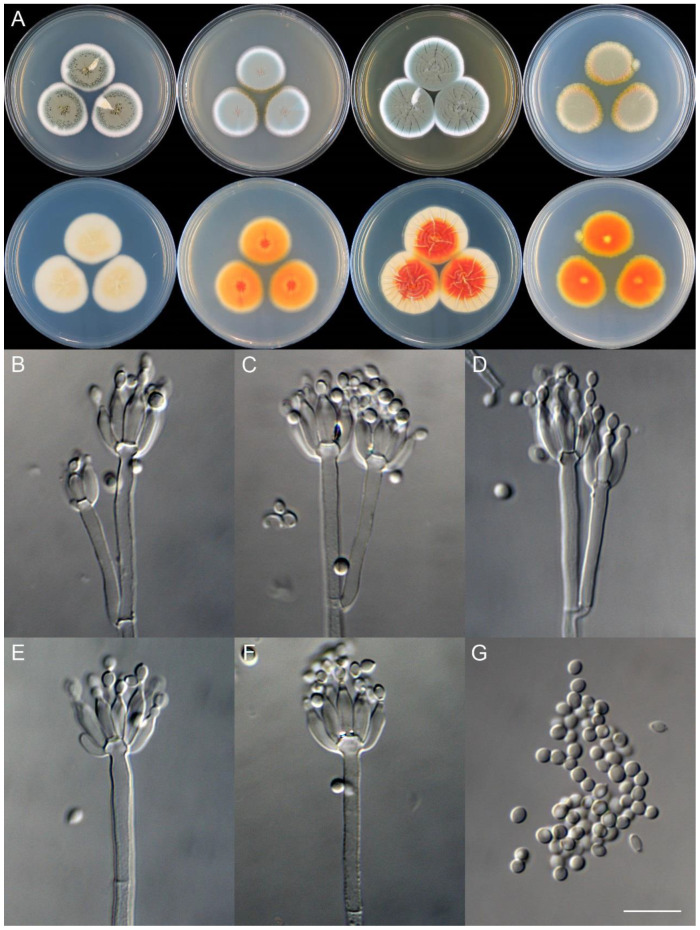
*Penicillium xuanhanense* (CS31-04). (**A**) Colonies: top row left to right, obverse CYA, MEA, YES, and PDA; bottom row left to right, reverse CYA, MEA, YES, and PDA; (**B**–**F**) Conidiophores; (**G**) Conidia. Bar: (**G**) = 10 µm, also for (**B**–**F**).

***Penicillium xuanhanense*** X.C. Wang & W.Y. Zhuang, sp. nov. Figure 49.

**Fungal Names:** FN571574.

**Etymology:** The specific epithet refers to the type locality.

In *Penicillium* subgenus *Aspergilloides* section *Sclerotiorum* series *Sclerotiorum*.

**Typification:** China. Sichuan Province, Dazhou City, Xuanhan County, Bashan Grand Canyon, 31°39′44″ N 108°51′17″ E, in soil of ant hole, 1 November 2020, Xin-Cun Wang, Huan-Di Zheng and Chang Liu, culture, Zhi-Kang Zhang, CS31-04 (holotype HMAS 247925, ex-type strain CGMCC 3.25186).

**DNA barcodes:** ITS OQ870873, BenA OR051222, CaM OR051396, RPB2 OR062086.

**Colony diam.**, 7 days, 25 °C (unless stated otherwise): CYA 30–31 mm; CYA 37 °C no growth; CYA 5 °C no growth; MEA 27–29 mm; YES 35–36 mm; PDA 27–28 mm.

**Colony characteristics:** On CYA 25 °C, 7 days: Colonies nearly circular, slightly sulcate, with white sectors; margins moderately wide, entire; mycelia white; texture velutinous; sporulation dense; conidia *en masse* bluish green to dull green; soluble pigments absent; exudates yellow and hyaline, clear; reverse cream to buff.

On MEA 25 °C, 7 days: Colonies nearly circular, protuberant at centers with pink hyphae; margins moderately wide, entire; mycelia white; texture velutinous; sporulation dense; conidia *en masse* dull green; soluble pigments absent; exudates absent; reverse light orange, red brown at centers.

On YES 25 °C, 7 days: Colonies nearly circular, radially and concentrically sulcate, concave at centers; margins moderately wide, undulated; mycelia white; texture velutinous; sporulation dense; conidia *en masse* dull green; soluble pigments absent; exudates absent; reverse white, red at centers.

On PDA 25 °C, 7 days: Colonies nearly circular, plain; margins moderately wide, entire; mycelia yellow; texture velutinous; sporulation dense; conidia *en masse* greyish green; soluble pigments absent; exudates absent; reverse orange, yellowish buff at centers and margins.

**Micromorphology:** Conidiophores monoverticillate or divaricate; stipes smooth to rough-walled, 45–150 × 2.5–3.5 μm; phialides ampulliform to acerose, tapering into very thin neck, 5–12 per stipe, 9–11 × 3.0–4.0 μm; conidia subglobose to ellipsoidal, smooth-walled, 3.0–3.5 × 2.5–3.0 μm.

**Notes:** This species is a sister of *P. asterineum* and *P. ferraniaense* (BP = 83, PP = 1.00, Figure 7). It differs from *P. asterineum* in 13 bp for BenA, three bp for CaM and 11 bp for RPB2; and differs from *P. ferraniaense* in nine bp for BenA, five bp for CaM and eight bp for RPB2. Morphologically, it differs from *P. asterineum* in slower growth rate on CYA (30–31 vs. 35–40 mm), MEA (27–29 vs. 34–37 mm) and YES (35–36 vs. 40–42 mm) at 25 °C, lacking of red radiate branches on the reverse of CYA and MEA, divaricate conidiophores, shorter stipes (45–150 vs. 100–300 μm) and larger conidia (3.0–3.5 vs. 2.5–3.0 μm); and it differs from *P. ferraniaense* in faster growth rate on CYA (30–31 vs. 25–28 mm) and YES (35–36 vs. 21–23 mm) at 25 °C, red brown reverse on MEA and YES, and longer stipes (45–150 vs. 50–80 μm) [60].

**Figure 50:**
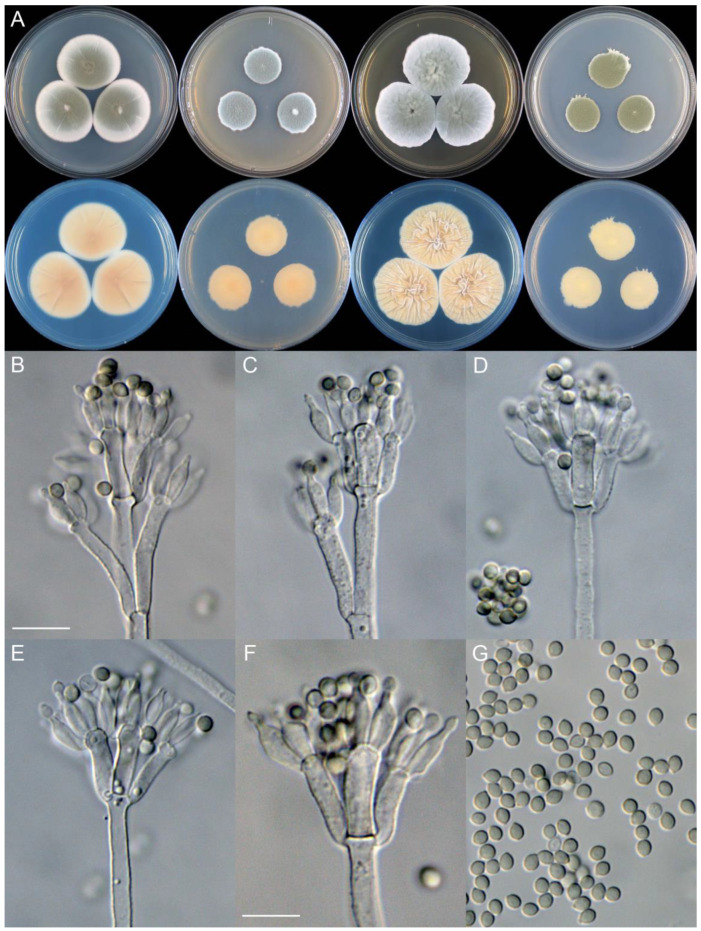
*Penicillium yuyongnianii* (CS13-01). (**A**) Colonies: top row left to right, obverse CYA, MEA, YES, and PDA; bottom row left to right, reverse CYA, MEA, YES, and PDA; (**B**–**F**) Conidiophores; (**G**) Conidia. Bars: (**B**) = 10 µm, also for (**C**–**E**,**G**); (**F**) = 7.5 µm.

***Penicillium yuyongnianii*** X.C. Wang & W.Y. Zhuang, sp. nov. Figure 50.

**Fungal Names:** FN571575.

**Etymology:** The specific epithet is named in memory of the late distinguished mycologist Professor Yong-Nian Yu (1923.04–2014.08).

In *Penicillium* subgenus *Aspergilloides* section *Lanata-Divaricata* series *Simplicissima*.

**Typification:** China. Chongqing City, Wanzhou City, Wangerbao Nature Reserve, Longju Town, Wutong Village, 30°36′26″ N 108°38′24″ E, in soil, 28 October 2020, Xin-Cun Wang, Huan-Di Zheng and Chang Liu, culture, Zhi-Kang Zhang, CS13-01 (holotype HMAS 247926, ex-type strain CGMCC 3.25187).

**DNA barcodes:** ITS OQ870820, BenA OR051175, CaM OR051352, RPB2 OR051499.

**Colony diam.**, 7 days, 25 °C (unless stated otherwise): CYA 35–37 mm; CYA 37 °C no growth; CYA 5 °C no growth; MEA 22–24 mm; YES 37–39 mm; PDA 21–24 mm.

**Colony characteristics:** On CYA 25 °C, 7 days: Colonies nearly circular, radially sulcate, furrows with white hyphae; margins narrow, entire; mycelia white; texture velutinous; sporulation dense; conidia *en masse* dull green; soluble pigments absent; exudates absent; reverse cream to buff.

On MEA 25 °C, 7 days: Colonies nearly circular, plain, slightly protuberant or funiculose at centers; margins narrow, entire; mycelia white; texture velutinous; sporulation dense; conidia *en masse* dull green; soluble pigments absent; exudates absent; reverse buff.

On YES 25 °C, 7 days: Colonies nearly circular or irregular, radially sulcate; margins narrow, entire; mycelia white; texture velutinous; sporulation dense; conidia *en masse* bluish green; soluble pigments absent; exudates absent; reverse buff, with brownish branches.

On PDA 25 °C, 7 days: Colonies nearly circular or irregular, plain; margins narrow, entire or irregular; mycelia white; texture velutinous; sporulation dense; conidia *en masse* dull green; soluble pigments absent; exudates absent; reverse cream.

**Micromorphology:** Conidiophores biverticillate or terverticillate; stipes smooth to rough-walled, 300–900 × 2.5–4.5 μm; rami 2–3, 19–23 × 3.0–4.0 μm; metulae 4–5, 10–15 × 3.0–4.5 μm; phialides ampulliform to acerose, tapering into very thin neck, 4–6 per metula, 7.5–9.5 × 2.5–3.5 μm; conidia subglobose to broad ellipsoidal, smooth-walled, 3.0–3.5 × 2.5–3.0 μm.

**Additional strain examined:** China. Chongqing City, Fengjie County, Caotang Town, 31°5′29″ N 109°38′57″ E, in soil, 29 October 2020, Xin-Cun Wang, Huan-Di Zheng and Chang Liu, culture, Zhi-Kang Zhang, CS14-23.

**Notes:** This species is a sister to *P. globosum* and *P. taii* (BP = 74, PP = 1.00, Figure 6). The molecular and morphological differences between this taxa and *P. taii* have been given in the Notes of the latter. This fungus differs from *P. globosum* in three bp for BenA, 13 bp for CaM and six bp for RPB2. Morphologically, it differs in fast growth rate on CYA (35–37 vs. 21–22 mm) and YES (37–39 vs. 17–19 mm) at 25 °C, no growth on CYA at 37 °C, terverticillate conidiophores and ellipsoidal conidia [43].

### 4.3. New Records for China

***Penicillium aurantioviolaceum*** Biourge, La Cellule 33(1): 282, 1923.

In *Penicillium* subgenus *Aspergilloides* section *Aspergilloides* series *Thomiorum*.

**Strains examined:** China. Chongqing City, Wushan County, Wulipo National Nature Reserve, 31°22′59″ N 109°56′11″ E, in soil, 29 October 2020, Xin-Cun Wang, Huan-Di Zheng and Chang Liu, culture, Zhi-Kang Zhang, CS20-37; *ibid.*, CS20-38; *ibid.*, CS21-02; *ibid.*, CS22-04.

**Notes:** This species was originally described from Puerto Rico [62], and had been isolated from Japan, Madagascar and Zambia [59], and was recently reported from South Korea [74]. Compared with the ex-type culture, the studied Chinese strains have identical sequences for BenA, one bp difference for CaM, and three bp differences for RPB2, which are treated as intra-specific variations.

***Penicillium cainii*** K.G. Rivera, Malloch and Seifert, Stud. Mycol. 70: 147, 2011.

In *Penicillium* subgenus *Aspergilloides* section *Sclerotiorum* series *Sclerotiorum*.

**Strains examined:** China. Chongqing City, Wushan County, Wulipo National Nature Reserve, 31°22′59″ N 109°56′11″ E, in soil, 29 October 2020, Xin-Cun Wang, Huan-Di Zheng and Chang Liu, culture, Zhi-Kang Zhang, CS21-03. Sichuan Province, Dazhou City, Wanyuan City, Longtanhe, 31°50′19″ N 108°19′15″ E, in soil, 1 November 2020, Xin-Cun Wang, Huan-Di Zheng and Chang Liu, culture, Zhi-Kang Zhang, CS33-11.

**Notes:** This species was initially discovered in Canada [64], and then reported from South Korea [75]. The strain CS33-11 has sequence similarity with the ex-type culture, but CS21-03 shows a few divergences.

***Penicillium circulare*** Hyang B. Lee, P.M. Kirk & T.T.T. Nguyen, Fungal Diversity 96: 97, 2019.

In *Penicillium* subgenus *Aspergilloides* section *Sclerotiorum* series *Sclerotiorum*.

**Strains examined:** China. Chongqing City, Fengjie County, Caotang Town, 31°5′29″ N 109°38′57″ E, in soil, 29 October 2020, Xin-Cun Wang, Huan-Di Zheng and Chang Liu, culture, Zhi-Kang Zhang, CS16-06; *ibid.*, CS16-07; *ibid.*, Wushan County, Shuanglong Town, Huazhu Village, 31°9′48″ N 109°47′7″ E, in soil, 29 October 2020, Xin-Cun Wang, Huan-Di Zheng and Chang Liu, culture, Zhi-Kang Zhang, CS18-12; *ibid.*, CS18-14. Sichuan Province, Dazhou City, Wanyuan City, Longtanhe, 31°50′19″ N 108°19′15″ E, in soil, 1 November 2020, Xin-Cun Wang, Huan-Di Zheng and Chang Liu, culture, Zhi-Kang Zhang, CS33-08.

**Notes:** This species was first reported from South Korea [69], and now discovered from different areas of Southwestern China. The strains examined have identical BenA and RPB2 sequences with the ex-type culture, but has four bp differences for CaM.

***Penicillium cosmopolitanum*** Houbraken, Frisvad & Samson, Stud. Mycol. 70: 91, 2011.

In *Penicillium* subgenus *Aspergilloides* section *Citrina* series *Westlingiorum*.

**Strain examined:** China. Chongqing City, Nanchuan District, Jinfo Mountain National Nature Reserve, Lingguan Cave, 29°1′55″ N 107°11′57″ E, in soil of dry stream, 26 October 2020, Chang Liu, Zhao-Qing Zeng, Xin-Cun Wang and Huan-Di Zheng, culture, Zhi-Kang Zhang, CS11-04.

**Notes:** This species was discovered from Europe (Denmark, The Netherlands and Poland), Oceania (New Zealand), and Asia (South Korea) [73,74]. The Chongqing strain has identical BenA, CaM and RPB2 sequences with the ex-type.

***Penicillium hetheringtonii*** Houbraken, Frisvad & Samson, Fungal Diversity 44: 125, 2010.

In *Penicillium* subgenus *Aspergilloides* section *Citrina* series *Citrina*.

**Strain examined:** China. Chongqing City, Beibei District, Jinyun Mountain National Nature Reserve, 29°50′18″ N 106°23′45″ E, in soil of bamboo grove23 October 2020, Chang Liu, Zhao-Qing Zeng, Xin-Cun Wang and Huan-Di Zheng, culture, Zhi-Kang Zhang, CS01-09.

**Notes:** This species was originally isolated from beach soil in Florida [76], and then discovered in marine environment in Jeju Island, South Korea [77]. This Chinese collection extends its distribution to mountainous region.

**Figure 51:**
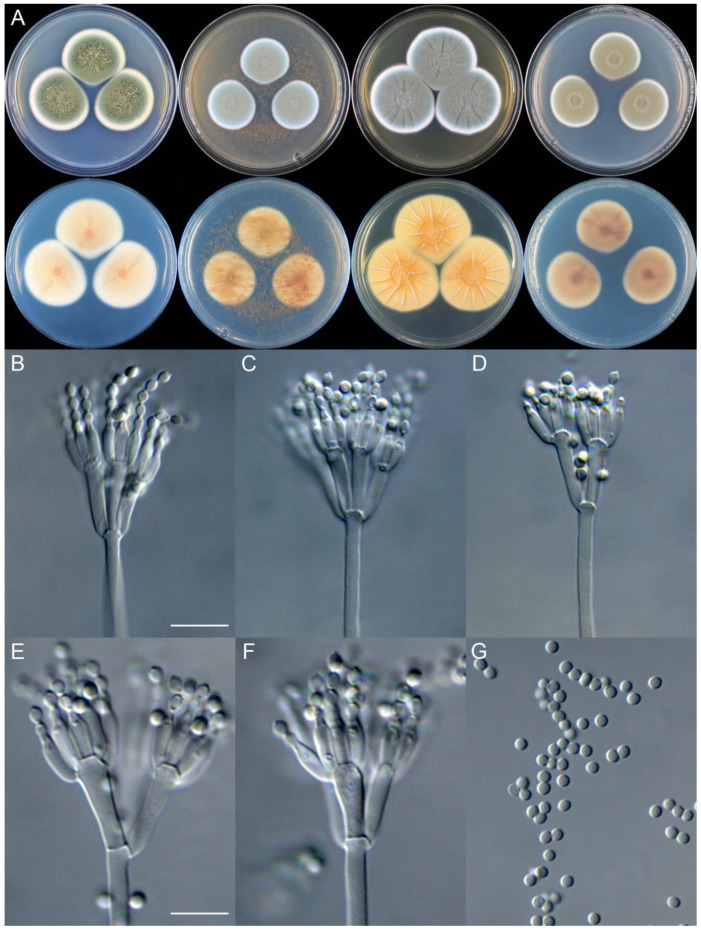
*Penicillium jenningsiae* (CS02-04). (**A**) Colonies: top row left to right, obverse CYA, MEA, YES, and PDA; bottom row left to right, reverse CYA, MEA, YES, and PDA; (**B**–**F**) Conidiophores; (**G**) Conidia. Bars: (**B**) = 10 µm, also for (**C**,**D**,**G**); (**E**) = 7.5 µm, also for (**F**).

***Penicillium jenningsiae*** Y.P. Tan, Bishop-Hurley, E. Lacey & R.G. Shivas, Index of Australian Fungi 3: 8, 2022. Figure 51.

In *Penicillium* subgenus *Aspergilloides* section *Citrina* series *Sumatraensia*.

**Strains examined:** China. Chongqing City, Beibei District, Jinyun Mountain National Nature Reserve, 29°50′18″ N 106°23′45″ E, in soil, 23 October 2020, Chang Liu, Zhao-Qing Zeng, Xin-Cun Wang and Huan-Di Zheng, culture, Zhi-Kang Zhang, CS02-04. Sichuan Province, Dazhou City, Wanyuan City, Longtanhe, 31°50′19″ N 108°19′15″ E, in soil, 1 November 2020, Xin-Cun Wang, Huan-Di Zheng and Chang Liu, culture, Zhi-Kang Zhang, CS33-13.

**Colony diam.**, 7 days, 25 °C (unless stated otherwise): CYA 37–38 mm; CYA 37 °C no growth; CYA 5 °C no growth; MEA 29–30 mm; YES 42–43 mm; PDA 30–32 mm.

**Colony characteristics:** On CYA 25 °C, 7 days: Colonies nearly circular, plain, protuberant at centers; margins moderately wide, entire; mycelia white; texture velutinous; sporulation dense; conidia *en masse* viridian green; soluble pigments absent; exudates hyaline, clear, massive; reverse buff.

On MEA 25 °C, 7 days: Colonies nearly circular, plain, slightly protuberant at centers; margins narrow, entire; mycelia white; texture velutinous; sporulation dense; conidia *en masse* brownish green; soluble pigments absent; exudates absent; reverse white.

On YES 25 °C, 7 days: Colonies nearly circular, radially sulcate, deep; margins moderately wide, entire; mycelia white; texture velutinous; sporulation dense; conidia *en masse* dull green; soluble pigments absent; exudates absent; reverse buff to yellow brown.

On PDA 25 °C, 7 days: Colonies nearly circular; margins narrow, entire; mycelia white; texture velutinous; sporulation dense; conidia *en masse* brownish green; soluble pigments pink to light purple; exudates absent; reverse purplish brown.

**Micromorphology:** Conidiophores biverticillate; stipes smooth-walled, 200–315 × 2.5 μm; metulae 3–5, 10.5–14 × 2.5–4.5 μm; phialides ampulliform, tapering into very thin neck, 5–7 per metula, 7–9.5 × 2.0–2.5 μm; conidia subglobose to broad ellipsoidal, smooth-walled, 2.5 × 2.0–2.5 μm.

**Notes:** This species was recently described from Australia based on BenA and RPB2 sequence divergences [30]. The two strains examined in this study have identical BenA sequences with the ex-type culture, but CS02-04 has five bp differences for RPB2 from the ex-type culture. Due to illustration and description on different media of this species was not given in the protologue, this species was described and illustrated in detail.

***Penicillium koreense*** S.B. Hong, D.H. Kim & Y.H. You, J. Microbiol. Biotechnol. 24(12): 1607, 2014.

In *Penicillium* subgenus *Aspergilloides* section *Lanata-Divaricata* series *Janthinella*.

**Strain examined:** China. Chongqing City, Wushan County, Shuanglong Town, Wulong Village, 31°12′17″ N 109°47′31″ E, in soil, 29 October 2020, Xin-Cun Wang, Huan-Di Zheng and Chang Liu, culture, Zhi-Kang Zhang, CS19-06.

**Notes:** This species was reported from South Korea [72], and appeared in Southwestern China. The strain examined has two bp differences for BenA and five bp for CaM from the Korea material.

***Penicillium smithii*** Quintan., Avances en Alimentación y Mejora Animal 23: 340, 1982.

In *Penicillium* subgenus *Aspergilloides* section *Exilicaulis* series *Lapidosa*.

**Strain examined:** China. Chongqing City, Beibei District, Jinyun Mountain National Nature Reserve, 29°50′18″ N 106°23′45″ E, in soil, 23 October 2020, Chang Liu, Zhao-Qing Zeng, Xin-Cun Wang and Huan-Di Zheng, culture, Zhi-Kang Zhang, CS02-06.

**Notes:** This species was originally isolated from *Secale cereal* in Spain, and was thought to be distribute in Australia and Indonesia [78]. The Chinese strain was determined as *P. smithii* with strong support (BP = 100, PP = 1.00, Figure 4), but it still has some sequence divergences, i.e., four bp for BenA, eight bp for CaM and two bp for RPB2. This is attributed to intra-specific variation.

**Figure 52:**
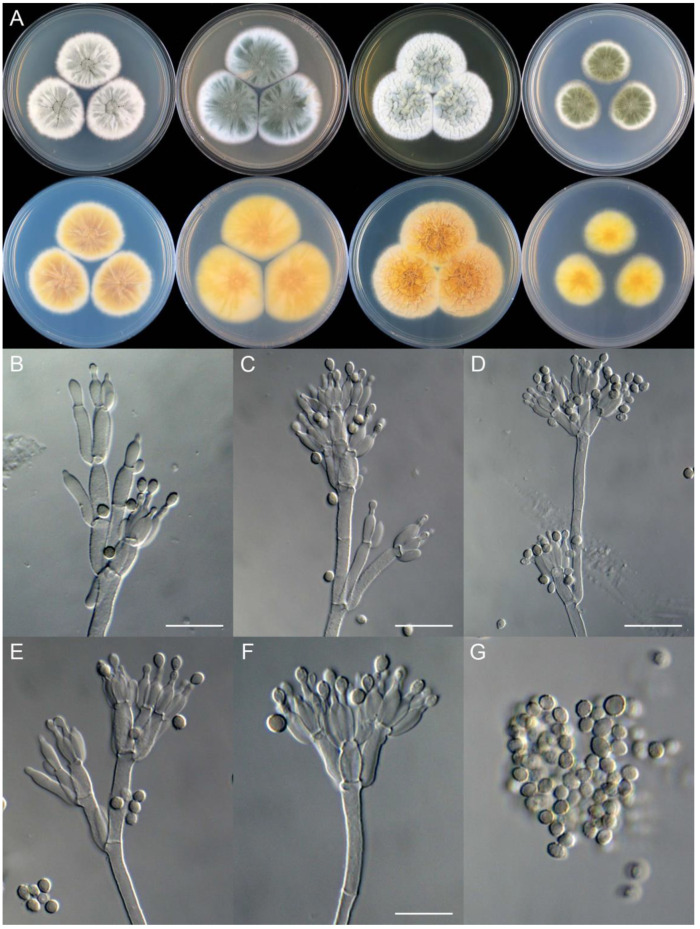
*Penicillium uttarakhandense* (CS24-01). (**A**) Colonies: top row left to right, obverse CYA, MEA, YES, and PDA; bottom row left to right, reverse CYA, MEA, YES, and PDA; (**B**–**F**) Conidiophores; (**G**) Conidia. Bars: (**B**) = 12.5 µm, also for (**E**); (**C**) = 15 µm; (**D**) = 17.5 µm; (**F**) = 10 µm, also for (**G**).

***Penicillium uttarakhandense*** Rajeshk., N. Ashtekar, Visagie, G. Anand & Yilmaz, Persoonia 46: 493, 2021. Figure 52.

In *Penicillium* subgenus *Aspergilloides* section *Lanata-Divaricata* series *Simplicissima*

**Strains examined:** China. Chongqing City, Wushan County, Wulipo National Nature Reserve, 31°22′59″ N 109°56′11″ E, in soil, 29 October 2020, Xin-Cun Wang, Huan-Di Zheng and Chang Liu, culture, Zhi-Kang Zhang, CS20-36; *ibid.*, Wuxi County, Hongchiba National Forest Park, 31°33′3″ N 109°1′36″ E, in soil under *Larix* sp., 30 October 2020, Xin-Cun Wang, Huan-Di Zheng and Chang Liu, culture, Zhi-Kang Zhang, CS24-01; *ibid.*, CS24-06.

**Colony diam.**, 7 days, 25 °C (unless stated otherwise): CYA 36–38 mm; CYA 37 °C 7–9 mm; CYA 5 °C no growth; MEA 42–44 mm; YES 41–42 mm; PDA 28–29 mm.

**Colony characteristics:** On CYA 25 °C, 7 days: Colonies nearly circular, concave at centers, radially sulcate; margins wide, entire; mycelia white; texture velutinous; sporulation dense; conidia *en masse* greenish grey; soluble pigments absent; exudates hyaline, clear; reverse yellow brown.

On CYA 37 °C, 7 days: Colonies irregular, concave at centers; margins moderately wide, entire; mycelia white; texture velutinous; sporulation dense; conidia *en masse* greyish green; soluble pigments absent; exudates absent; reverse orange brown.

On MEA 25 °C, 7 days: Colonies nearly circular, slightly protuberant at centers; margins moderately wide, entire; mycelia white; texture velutinous; sporulation dense; conidia *en masse* greyish green; soluble pigments absent; exudates absent; reverse yellow brown.

On YES 25 °C, 7 days: Colonies nearly circular, strongly sulcate, protuberant at centers; margins wide, fimbriate; mycelia white; texture velutinous; sporulation moderately dense; conidia *en masse* yellow and grey; soluble pigments absent; exudates absent; reverse yellow brown.

On PDA 25 °C, 7 days: Colonies nearly circular, protuberant at centers; margins moderately wide, entire; mycelia white; texture velutinous; sporulation dense; conidia *en masse* greyish green and dull green; soluble pigments absent; exudates absent; reverse greenish yellow.

**Micromorphology:** Conidiophores quaterverticillate, terverticillate or biverticillate; stipes usually rough-walled, sometimes smooth-walled, 125–300 × 2.5–4.0 μm; branches 2–3, 12.5–31.5 × 3.0–4.0 μm; rami 2–3, 9.0–31.5 (–54) × 2.5–3.5 μm; metulae 2–6, 8.5–17.5 × 2.5–4.5 μm; phialides ampulliform, tapering into very thin neck, 5–8 per metula, 7.5–12.5 × 2.5–3.5 μm; conidia ellipsoidal, rough-walled, 3.5–4.0 (–6.0) × 2.5–3.5 (–5.0) μm.

**Notes:** This species was recently described from Northern India [60]. The Chinese material examined share identical BenA sequence with the ex-type culture, but have two bp divergences for CaM and one bp for RPB2, which are treated as intraspecific variations. The morphological difference between the Chinese strains and the type is obscure. This record extends the distribution of the fungus to East Asia.

***Penicillium vasconiae*** C. Ramírez & A.T. Martínez, Mycopathologia 72(3): 189, 1980.

In *Penicillium* subgenus *Aspergilloides* section *Lanata-Divaricata* series *Rolfsiorum*.

**Strain examined:** China. Chongqing City, Wushan County, Wulipo National Nature Reserve, 31°22′59″ N 109°56′11″ E, in soil, 29 October 2020, Xin-Cun Wang, Huan-Di Zheng and Chang Liu, culture, Zhi-Kang Zhang, CS20-23.

**Notes:** This species was originally described from Spain [65], and was subsequently reported rarely in other regions of the world. The Chinese strain has two bp differences for BenA, six bp for CaM and four bp for RPB2, which are treated as intra-specific variations. It might be the first record in Asia.

***Penicillium westlingii*** K.W. Zaleski, Bull. Acad. Polon. Sci., Math. Nat., Sér. B: 473, 1927.

In *Penicillium* subgenus *Aspergilloides* section *Citrina* series *Westlingiorum*.

**Strain examined:** China. Chongqing City, Nanchuan District, Jinfo Mountain National Nature Reserve, North mountain slope, 29°5′35″ N 107°14′47″ E, in soil, 25 October 2020, Chang Liu, Zhao-Qing Zeng, Xin-Cun Wang and Huan-Di Zheng, culture, Zhi-Kang Zhang, CS10-16.

**Notes:** This species was originally described from Poland, and has a worldwide distribution [73]. In Asia, it was reported from marine environments in South Korea [77]. This Chinese strain has three bp differences for BenA, one bp for CaM and five bp for RPB2 from the ex-type.

## 5. Discussion

A total of 179 cultures of *Penicillium* were isolated from 33 soil samples collected in Southwest China, and subsequently identified and classified into 2 subgenera (*Aspergilloides* and *Penicillium*), 11 sections (*Aspergilloides*, *Canescentia*, *Citrina*, *Exilicaulis*, *Fasciculata*, *Gracilenta*, *Lanata-Divaricata*, *Penicillium*, *Ramosum*, *Robsamsonia*, and *Sclerotiorum*), 25 series, and 74 species with different isolation frequencies. Based on 3-locus phylogenetic analyses and morphological comparisons, 43 species were discovered as new to science, and a new series *Simianshanica* was established in sect. *Aspergilloides*. Additionally, 11 species were recorded for the first time from China.

Different species have different isolation frequencies. *Penicillium brasilianum* and *P. ochrochloron* of sect. *Lanata-Divaricata* were from seven soil samples, respectively, and *P. daleae* of this section and *P. shihii* of sect. *Aspergilloides* were from five samples, respectively. In contrast, 46 species were discovered from only a single sample, such as *P. chengkouense* from sample CS28, *P. dabashanicum* from CS26, *P. jinfoshanicum* from CS12, and *P. simianshanicum* from CS04 (Table 1, Table 2, Table 3, Table 4, Table 5, Table 6 and Table 7). On the other hand, different soil samples harbored various levels of *Penicillium* diversity. There are nine species in sample CS20: *P. aurantioviolaceum*, *P. brasilianum*, *P. ellipsoideum*, *P. paraherquei*, *P. shihii*, *P. subrutilans*, *P. uttarakhandense*, *P. vasconiae*, and *P. wangwentsaii*; eight species in sample CS02: *P. beibeiense*, *P. creberum*, *P. janczewskii*, *P. jenningsiae*, *P. jinyunshanicum*, *P. smithii*, *P. soliforme*, and *P. sphaerioides*; eight species in CS33: *P. brevistipitatum*, *P. cainii*, *P. circulare*, *P. dazhouense*, *P. flosculum*, *P. jenningsiae*, *P. johnpittii*, and *P. wanyuanense*; and seven species in CS04: *P. adametzii*, *P. jiangjinense*, *P. pauciramulum*, *P. simianshanicum*, *P. sphaerioides*, *P. subasperum*, and *P. tangii*. Five samples contained only one species: *P. ochrochloron* in CS07, *P. brasilianum* in CS17, *P. koreense* in CS19, *P. tengii* in CS27, and *P. ellipsoideum* in CS29. Aspergillaceae was not found in sample CS06. This gives the hint that different soil samples, even though from the nearby localities, might have different compositions of fungal communities.

Among the 74 species, seven belong to subgen. *Penicillium* and the remaining 67 in subgen. *Aspergilloides*: two in sect. *Exilicaulis*, three in *Gracilenta*, six in sect. *Aspergilloides*, 11 in *Citrina*, 21 in *Lanata-Divaricata*, and 24 in *Sclerotiorum*. Among the 43 new species, two belong to subgen. *Penicillium* and the rest 41 are in subgen. *Aspergilloides*: one in sect. *Exilicaulis*, three in *Gracilenta*, four in sect. *Aspergilloides*, five in *Citrina*, eight in *Lanata-Divaricata*, and 20 in *Sclerotiorum*. Section *Sclerotiorum* is the most speciose, and embrace most of the new species of this study. For the 20 new species of the section, 14 of them are in ser. *Herqueorum*, six in ser. *Sclerotiorum*, but none in ser. *Adametziorum*. As the previous study clearly pointed out, “it should be noted, however, that preliminary data show that this clade (ser. *Herqueorum*) represents a species complex, with several species undescribed” [61]. As a result, three species of the series had been described from South China by our team: *P. choerospondiatis*, *P. sanshaense* and *P. verrucisporum* [42]. This could partly explain that why so many new species appeared in this series. In addition, so many undescribed species discovered in series *Herqueorum* and *Sclerotiorum* might indicate that East Asia is the diversification center of them. East Asia had been reported as diversification center of many plants, e.g., *Galium* of Rubiaceae [79], *Panax* of Araliaceae [80] and *Pinus* of Pinaceae [81]. Many described species of ser. *Sclerotiorum* from East Asia and Southeast Asia could be the additional evidence: *P. acidum*, *P. circulare*, *P. daejeonium* and *P. ulleungdoense* from South Korea, *P. austrosinicum* and *P. exsudans* from China, *P. viticola* from Japan, *P. hirayamae* from Thailand, *P. johnkrugii* from Malaysia, and *P. sclerotiorum* from Indonesia (Table 7).

For the 48 *Penicillium* species previously described from China, it can be summarized that they are from 21 provinces or province-level administrative divisions: 14 from Hainan, 3 in Guangdong and Hubei, respectively, 2 species from 10 provinces, respectively (Beijing, Guangxi, Guizhou, Hunan, Jilin, Qinghai, Shaanxi, Taiwan, Tibet, and Yunnan), and 1 from eight provinces, respectively (Chongqing, Fujian, Gansu, Heilongjiang, Jiangxi, Liaoning, Shandong, and Xinjiang). In contrast to only 1 new species, *P. macrosclerotiorum,* reported from Chongqing [34] and none from Sichuan, 43 new species were discovered from some parts of the same area in this study. This might be attributed to two reasons. First, Southwestern China possesses three of the 35 global biodiversity hotspots [82], thus the level of biodiversity there is very high. For example, two new species of *Talaromyces* discovered during this exploration had been reported previously [49]. Second, dense sampling leads to more discovery of undescribed species. Diao et al. [43] collected samples from seven different sites (Diaoluo Mountain, Jianfengling, Lingshui, Qixianling, Wanning, Wuzhishan and Xinglong) of Hainan Province, and discovered 11 new species from acidic soil. Visagie and Yilmaz [27] collected 6 soil samples from a National Park and 18 *Penicillium* species were identified, including 6 new ones. More explorations or surveys are badly needed in the under-investigated areas of this country.

## Figures and Tables

**Table 1:** Species and sequences of *Penicillium* subgen. *Penicillium* used in phylogenetic analyses.

Subgenus	Section	Series	Species	Strain	Locality	Substrate	ITS	BenA	CaM	RPB2
*Penicillium*	*Canescentia*	*Canescentia*	*P. allsoppiae* Visagie et al. 2021	CBS 138943 T	South Africa	soil	JX140830	JX140992	JX157384	KP016895
*Penicillium*	*Canescentia*	*Canescentia*	*P. arizonense* Frisvad et al. 2016	CBS 141311 T	USA	soil	MH492021	MH492019	MH492020	MH492022
*Penicillium*	*Canescentia*	*Canescentia*	*P. canescens* Sopp 1912	CBS 300.48 T	UK	soil	AF033493	JX140946	MN969241	JN121485
*Penicillium*	*Canescentia*	*Canescentia*	***P. chengkouense*** X.C. Wang & W.Y. Zhuang, sp. nov.	CS28-01 = CGMCC 3.25149 T	China: Chongqing	soil	**OQ870783**	**OR051044**	**OR051223**	**OR051397**
*Penicillium*	*Canescentia*	*Canescentia*	*P. corvianum* Visagie & Seifert 2016	CBS 141000 T	Italy	soil	KT887875	KT887836	KT887797	MN969170
*Penicillium*	*Canescentia*	*Canescentia*	*P. dunedinense* Visagie et al. 2014	CBS 138218 T	New Zealand	house dust	KJ775678	KJ775171	KJ775405	MN969116
*Penicillium*	*Canescentia*	*Canescentia*	*P. eickeri* Visagie et al. 2021	CBS 138939 T	South Africa	mite from Protea repens infructescence	JX140824	JX140979	JX157365	KP016876
*Penicillium*	*Canescentia*	*Canescentia*	*P. elizabethiae* Visagie & Frisvad 2021	NRRL 917 T	UK	soil	KP016840	KJ866964	KJ867021	KP016918
*Penicillium*	*Canescentia*	*Canescentia*	*P. griseoazureum* Moreau & Moreau ex C. Ramírez 1982	CBS 162.42 T	France	sand dunes	KC411679	KP016919	KP016823	KP016852
*Penicillium*	*Canescentia*	*Canescentia*	*P. irregulare* Torres-García et al. 2022	FMR 17859 T	Spain	fluvial sediment	LR814181	LR814144	LR814151	LR814182
*Penicillium*	*Canescentia*	*Canescentia*	*P. janczewskii* K.W. Zaleski 1927	CBS 221.28 T	Poland	soil under *Pinus* sp.	AY157487	MN969386	MN969267	JN406612
				CS02-07	China: Chongqing	soil	**OQ870784**	**OR051045**	**OR051224**	**OR051398**
				CS10-08	China: Chongqing	soil	**OQ870785**	**OR051046**	**OR051225**	**OR051399**
*Penicillium*	*Canescentia*	*Canescentia*	*P. jensenii* K.W. Zaleski 1927	CBS 327.59 T	Japan	forest soil	AY443470	JX140954	AY443490	JN406614
*Penicillium*	*Canescentia*	*Canescentia*	*P. linzhiense* H.K. Wang & R. Jeewon 2021	CCTCC M2019870 T	China: Tibet	soil	MT461156	MT461157	MT461162	n.a.
*Penicillium*	*Canescentia*	*Canescentia*	*P. murcianum* C. Ramírez & A.T. Martínez 1981	CBS 161.81 T	Spain	sandy soil	MN431400	MN969419	MN969341	MN969202
*Penicillium*	*Canescentia*	*Canescentia*	*P. nigricans* Bainier ex Thom 1930	CBS 354.48 T	France	unknown	KC411755	KJ866965	KJ867012	KP016857
*Penicillium*	*Canescentia*	*Canescentia*	*P. radiatolobatum* Lörinczi 1972	CBS 340.79 T	Romania	soil	KC411745	MN969413	MT066183	MN969168
*Penicillium*	*Canescentia*	*Canescentia*	*P. scottii* Visagie et al. 2021	CBS 138951 T	South Africa	soil	JX140812	JX140991	JX157383	KP016894
*Penicillium*	*Canescentia*	*Canescentia*	*P. yarmokense* Baghd. 1968	CBS 410.69 T	Syria	soil	KC411757	MN969407	MN969314	JN406553
*Penicillium*	*Fasciculata*	*Camembertiorum*	*P. biforme* Thom 1910	CBS 297.48 T	USA	French cheese	KC411731	MN969373	KU896823	KU904346
*Penicillium*	*Fasciculata*	*Camembertiorum*	*P. camemberti* Thom 1906	CBS 299.48 T	USA	Camembert cheese	AB479314	FJ930956	KU896825	MN969109
*Penicillium*	*Fasciculata*	*Camembertiorum*	*P. caseifulvum* F. Lund et al. 1998	CBS 101134 T	Denmark	Danablu cheese	KJ834504	AY674372	KU896826	KU904347
*Penicillium*	*Fasciculata*	*Camembertiorum*	*P. cavernicola* Frisvad & Samson 2004	CBS 100540 T	USA	unknown	KJ834505	KJ834439	KU896827	KU904348
*Penicillium*	*Fasciculata*	*Camembertiorum*	*P. commune* Thom 1910	CBS 311.48 T	USA	cheese	AY213672	MN969377	KU896829	KU904350
*Penicillium*	*Fasciculata*	*Camembertiorum*	*P. crustosum* Thom 1930	CBS 115503 T	UK	lemon	AF033472	MN969379	DQ911132	MN969114
*Penicillium*	*Fasciculata*	*Camembertiorum*	***P. dabashanicum*** X.C. Wang & W.Y. Zhuang, sp. nov.	CS26-07 = CGMCC 3.25154 T	China: Chongqing	soil under a palm tree	**OQ870786**	**OR051047**	**OR051226**	**OR051400**
*Penicillium*	*Fasciculata*	*Camembertiorum*	*P. discolor* Frisvad & Samson 1997	CBS 474.84 T	Israel	*Raphanus sativus*	AJ004816	AY674348	KU896834	KU904351
*Penicillium*	*Fasciculata*	*Camembertiorum*	*P. echinulatum* Raper & Thom ex Fassat. 1977	CBS 317.48 T	Canada	culture contaminant	AF033473	AY674341	DQ911133	KU904352
*Penicillium*	*Fasciculata*	*Camembertiorum*	*P. palitans* Westling 1911	CBS 107.11 T	Germany	unknown	KJ834514	KJ834480	KU896847	KU904360
*Penicillium*	*Fasciculata*	*Camembertiorum*	*P. solitum* Westling 1911	CBS 424.89 T	Germany	unknown	AY373932	MN969398	KU896851	KU904363
*Penicillium*	*Fasciculata*	*Camembertiorum*	*P. speluncae* Visagie & N. Yilmaz 2020	DAOMC 251701 T	Canada	swab of deer mouse fur	MG490869	MG490889	MG490959	MN170741
*Penicillium*	*Penicillium*	*Penicillium*	*P. expansum* Link 1809	CBS 325.48 T	USA	fruit of *Malus sylvestris*	AY373912	AY674400	DQ911134	JF417427
				CS11-01	China: Chongqing	soil in a cave	**OQ870787**	**OR051048**	**OR051227**	**OR051401**
				CS11-02	China: Chongqing	soil in a cave	**OQ870788**	**OR051049**	**OR051228**	**OR051402**
				CS11-03	China: Chongqing	soil in a cave	**OQ870789**	**OR051050**	**OR051229**	**OR051403**
				CS28-03	China: Chongqing	soil	**OQ870790**	**OR051051**	**OR051230**	**OR051404**
				CS30-02	China: Sichuan	soil	**OQ870791**	**OR051052**	**OR051231**	**OR051405**
*Penicillium*	*Penicillium*	*Penicillium*	*P. marinum* Frisvad & Samson 2004	CBS 109550 T	Japan	sandy soil	KJ834512	AY674392	KU896842	KU904357
*Penicillium*	*Ramosum*	*Scabrosa*	*P. scabrosum* Frisvad et al. 1990	CBS 683.89 T	Denmark	soil associated with *Zea mays*	DQ267906	DQ285610	FJ530987	JN406541
				CS28-02	China: Chongqing	soil	**OQ870792**	**OR051053**	**OR051232**	**OR051406**
				CS28-21	China: Chongqing	soil	**OQ870793**	**OR051054**	**OR051233**	**OR051407**
*Penicillium*	*Ramosum*	*Virgata*	*P. virgatum* Nirenberg & Kwaśna 2005	CBS 114838 T	New Caledonia	soil of field of *Glycine max*	AJ748692	KJ834500	KJ866992	JN406641
				CS26-77	China: Chongqing	soil under a palm tree	**OQ870794**	**OR051055**	**OR051234**	**OR051408**
				CS26-78	China: Chongqing	soil under a palm tree	**OQ870795**	**OR051056**	**OR051235**	n.a.
*Penicillium*	*Robsamsonia*	*Robsamsonia*	*P. brevistipitatum* L. Wang & W.Y. Zhuang 2005	CGMCC 3.6887 T	China: Jilin	soil	DQ221696	DQ221695	KU896824	JN406528
				CS33-12	China: Sichuan	soil	**OQ870796**	**OR051057**	**OR051236**	**OR051409**
*Penicillium*	*Robsamsonia*	*Robsamsonia*	*P. compactum* L. Wang & Houbraken 2016	CGMCC 3.15411 T	China: Heilongjiang	soil	KM973207	KM973203	KM973200	KT698909
*Penicillium*	*Robsamsonia*	*Robsamsonia*	*P. concentricum* Samson et al. 1976	CBS 477.75 T	Germany	colon of Cervidae	KC411763	AY674413	DQ911131	KT900575
*Penicillium*	*Robsamsonia*	*Robsamsonia*	*P. coprobium* Frisvad 1990	CBS 561.90 T	Norway	pig feed	DQ339559	AY674425	KU896830	KT900576
*Penicillium*	*Robsamsonia*	*Robsamsonia*	*P. coprophilum* (Berk. & M.A. Curtis) Seifert & Samson 1986	CBS 110760 T	Cuba	dung of Aves	AF033469	AY674421	KU896831	JN406645
*Penicillium*	*Robsamsonia*	*Robsamsonia*	*P. fimorum* Frisvad & Houbraken 2016	CBS 140575 T	Denmark	dung of mouse	KU904343	KT698889	KT698898	KT698908
*Penicillium*	*Robsamsonia*	*Robsamsonia*	*P. robsamsonii* Frisvad & Houbraken 2016	CBS 140573 T	Denmark	dung of mouse	KU904339	KT698885	KT698894	KT698904
*Penicillium*	*Eladia*	*Eladia*	*P. sacculum* E. Dale 1926	CBS 231.61 T	UK	soil	KC411707	KJ834488	KU896849	JN121462

GenBank accession numbers in bold indicate the newly generated sequences. The phrase ‘n.a.’ is the abbreviation of ‘not available’.

**Table 2:** Species and sequences of *Penicillium* subgen. *Aspergilloides* sect. *Aspergilloides* used in phylogenetic analyses.

Subgenus	Section	Series	Species	Strain	Locality	Substrate	ITS	BenA	CaM	RPB2
*Aspergilloides*	*Aspergilloides*	*Glabra*	*P. armarii* Houbraken et al. 2014	CBS 138171 T	Australia	house dust	KM189758	KM089007	KM089394	KM089781
*Aspergilloides*	*Aspergilloides*	*Glabra*	*P. bussumense* Houbraken 2014	CBS 138160 T	The Netherlands	soil	KM189458	KM088685	KM089070	KM089457
*Aspergilloides*	*Aspergilloides*	*Glabra*	*P. frequentans* Westling 1911	CBS 105.11 T	unknown	unknown	KM189525	KM088762	KM089147	KM089534
*Aspergilloides*	*Aspergilloides*	*Glabra*	*P. glabrum* (Wehmer) Westling 1911	CBS 125543 T	unknown	unknown	GU981567	GU981619	KM089152	JF417447
				CS14-37	China: Chongqing	soil	**OQ870797**	**OR051058**	**OR051237**	**OR051410**
				CS25-45	China: Chongqing	soil	**OQ870798**	**OR051059**	**OR051238**	**OR051411**
*Aspergilloides*	*Aspergilloides*	*Glabra*	*P. pulvis* Houbraken et al. 2014	CBS 138432 T	South Africa	house dust	KM189632	KM088876	KM089263	KM089650
*Aspergilloides*	*Aspergilloides*	*Glabra*	*P. purpurescens* (Sopp) Biourge 1923	CBS 366.48 T	Canada	soil	KM189561	KM088801	KM089186	KM089573
*Aspergilloides*	*Aspergilloides*	*Glabra*	*P. rudallense* Houbraken et al. 2014	CBS 138162 T	Australia	soil	KM189504	KM088741	KM089126	KM089513
*Aspergilloides*	*Aspergilloides*	*Livida*	*P. lividum* Westling 1911	CBS 347.48 T	UK	unknown	KM189582	KM088825	KM089211	KM089598
*Aspergilloides*	*Aspergilloides*	*Livida*	*P. kananaskense* Seifert et al. 1994	CBS 530.93 T	Canada	soil under *Pinus contorta* var. *latifolia*	KM189780	KM089030	KM089417	KM089804
*Aspergilloides*	*Aspergilloides*	*Livida*	*P. odoratum* M. Chr. & Backus 1962	CBS 294.62 T	USA	peaty soil	KC411730	KJ834478	KM089363	JN406583
*Aspergilloides*	*Aspergilloides*	*Livida*	***P. shihii*** X.C. Wang & W.Y. Zhuang, sp. nov.	CS22-03 = CGMCC 3.25168 T	China: Chongqing	soil	**OQ870799**	**OR051060**	**OR051239**	**OR051412**
				CS20-40	China: Chongqing	soil	**OQ870800**	**OR051061**	**OR051240**	**OR051413**
				CS20-44	China: Chongqing	soil	**OQ870801**	**OR051062**	**OR051241**	**OR051414**
				CS30-12	China: Sichuan	soil	**OQ870802**	**OR051063**	**OR051242**	**OR051415**
				CS31-05	China: Sichuan	soil	**OQ870803**	**OR051064**	**OR051243**	**OR051416**
				CS32-01	China: Sichuan	soil of ant hole	**OQ870804**	**OR051065**	**OR051244**	**OR051417**
*Aspergilloides*	*Aspergilloides*	*Simianshanica*	***P. simianshanicum*** X.C. Wang & W.Y. Zhuang, sp. nov.	CS04-04 = CGMCC 3.25170 T	China: Chongqing	soil of ant hole	**OQ870805**	**OR051066**	**OR051245**	**OR051418**
				CS04-05	China: Chongqing	soil of ant hole	**OQ870806**	**OR051067**	**OR051246**	**OR051419**
				CS04-08	China: Chongqing	soil of ant hole	**OQ870807**	**OR051068**	**OR051247**	**OR051420**
*Aspergilloides*	*Aspergilloides*	*Spinulosa*	*P. grancanariae* C. Ramírez et al. 1978	CBS 687.77 T	Canary Islands	air	KM189529	KM088766	KM089151	KM089538
*Aspergilloides*	*Aspergilloides*	*Spinulosa*	*P. palmense* C. Ramírez et al. 1978	CBS 336.79 T	Canary Islands	air	KJ834515	GQ367508	GQ367534	JN406566
*Aspergilloides*	*Aspergilloides*	*Spinulosa*	*P. roseomaculatum* Biourge 1923	CBS 137962 T	unknown	unknown	KM189755	KM089004	KM089391	KM089778
*Aspergilloides*	*Aspergilloides*	*Spinulosa*	P. *spinulosum* Thom 1910	CBS 374.48 T	Germany	culture contaminant	AF033410	KJ834493	GQ367524	JN406558
*Aspergilloides*	*Aspergilloides*	*Spinulosa*	*P. sterculiniicola* Houbraken 2014	CBS 122426 T	USA	compost	KM189464	KM088693	KM089078	KM089465
*Aspergilloides*	*Aspergilloides*	*Spinulosa*	*P. subspinulosum* Houbraken 2014	CBS 137946 T	New Zealand	house dust	KM189483	KM088719	KM089104	KM089491
*Aspergilloides*	*Aspergilloides*	*Spinulosa*	***P. tangii*** X.C. Wang & W.Y. Zhuang, sp. nov.	CS04-07 = CGMCC 3.25177 T	China: Chongqing	soil of ant hole	**OQ870808**	**OR051069**	**OR051248**	**OR051421**
*Aspergilloides*	*Aspergilloides*	*Spinulosa*	*P. trzebinskii* K.W. Zaleski 1927	CBS 382.48 T	Poland	forest soil	KM189784	KM089034	KM089421	KM089808
*Aspergilloides*	*Aspergilloides*	*Thomiorum*	*P. aurantioviolaceum* Biourge 1923	CBS 137777 T	Puerto Rico	unknown	KM189756	KM089005	KM089392	KM089779
				CS20-37	China: Chongqing	soil	**OQ870809**	**OR051070**	**OR051249**	**OR051422**
				CS20-38	China: Chongqing	soil	**OQ870810**	**OR051071**	**OR051250**	n.a.
				CS21-02	China: Chongqing	soil	**OQ870811**	**OR051072**	**OR051251**	**OR051423**
				CS22-04	China: Chongqing	soil	**OQ870812**	**OR051073**	**OR051252**	**OR051424**
*Aspergilloides*	*Aspergilloides*	*Thomiorum*	*P. austroafricanum* Houbraken & Visagie 2014	CBS 137773 T	South Africa	leaves of *Phaenocoma prolifera*	KM189610	KM088854	KM089241	KM089628
*Aspergilloides*	*Aspergilloides*	*Thomiorum*	*P. cartierense* Houbraken 2014	CBS 137956 T	The Netherlands	soil	KM189564	KM088804	KM089189	KM089576
*Aspergilloides*	*Aspergilloides*	*Thomiorum*	*P. contaminatum* Houbraken 2014	CBS 345.52 T	UK	culture contaminant	KM189554	KM088793	KM089178	KM089565
*Aspergilloides*	*Aspergilloides*	*Thomiorum*	*P. crocicola* T. Yamam. 1956	CBS 745.70 T	Japan	corms of *Crocus sativus*	KM189581	KJ834445	KM089210	JN406535
*Aspergilloides*	*Aspergilloides*	*Thomiorum*	*P. fusisporum* L. Wang 2014	CGMCC 3.15338 T	China: Shaanxi	leaves of *Rhododendron*	KF769424	KF769400	KF769413	MN969117
*Aspergilloides*	*Aspergilloides*	*Thomiorum*	*P. grevilleicola* Houbraken & Quaedvlieg 2014	CBS 137775 T	Australia	leaf of *Grevillea ilicifolia*	KM189630	KM088874	KM089261	KM089648
*Aspergilloides*	*Aspergilloides*	*Thomiorum*	*P. jejuense* M.S. Park & Y.W. Lim 2015	CBS 138646 T	South Korea	*Pollicipes mitella*	KF818464	KF818461	KF818470	KF818467
*Aspergilloides*	*Aspergilloides*	*Thomiorum*	***P. jinfoshanicum*** X.C. Wang & W.Y. Zhuang, sp. nov.	CS12-10 = CGMCC 3.25161 T	China: Chongqing	soil	**OQ870813**	**OR051074**	**OR051253**	**OR051425**
				CS12-11	China: Chongqing	soil	**OQ870814**	**OR051075**	**OR051254**	**OR051426**
*Aspergilloides*	*Aspergilloides*	*Thomiorum*	*P. roseoviride* Stapp & Bortels 1935	CBS 267.35 T	Germany	soil in a beech forest	KM189549	KM088787	KM089172	KM089559
*Aspergilloides*	*Aspergilloides*	*Thomiorum*	*P. thomii* Maire 1917	CBS 225.81 T	USA	pinecone	KM189560	KM088799	KM089184	KM089571
*Aspergilloides*	*Aspergilloides*	*Thomiorum*	*P. valentinum* C. Ramírez & A.T. Martínez 1980	CBS 172.81 T	Spain	Air	KM189550	KM088788	KM089173	KM089560
*Aspergilloides*	*Aspergilloides*	*Thomiorum*	*P. yezoense* Hanzawa ex Houbraken 2014	CBS 350.59 T	Japan	butter	KM189553	KM088792	KM089177	KM089564
*Aspergilloides*	*Aspergilloides*	*Verhageniorum*	*P. ranomafanaense* Houbraken & F. Hagen 2014	CBS 137953 T	Madagascar	Soil	KM189541	KM088779	KM089164	KM089551
*Aspergilloides*	*Aspergilloides*	*Verhageniorum*	*P. verhagenii* Houbraken 2014	CBS 137959 T	Belgium	mosses under *Myrica gale*	KM189708	KM088955	KM089342	KM089729
*Aspergilloides*	*Aspergilloides*	*Thiersiorum*	*P. thiersii* S.W. Peterson et al. 2004	CBS 117503 T	USA	old stroma of *Hypoxylon*	AF125936	KJ834497	AY741726	JN121434

GenBank accession numbers in bold indicate the newly generated sequences. The phrase ‘n.a.’ is the abbreviation of ‘not available’.

**Table 3:** Species and sequences of *Penicillium* subgen. *Aspergilloides* sect. *Citrina* used in phylogenetic analyses.

Subgenus	Section	Series	Species	Strain	Locality	Substrate	ITS	BenA	CaM	RPB2
*Aspergilloides*	*Citrina*	*Citrina*	*P. citrinum* Thom 1910	CBS 139.45 T	unknown	unknown	AF033422	GU944545	MN969245	JF417416
				CS18-10	China: Chongqing	Soil	**OQ870874**	**OR051076**	n.a.	**OR051427**
*Aspergilloides*	*Citrina*	*Citrina*	*P. gorlenkoanum* Baghd. 1968	CBS 408.69 T	Syria	Soil	GU944581	GU944520	MN969259	JN606601
*Aspergilloides*	*Citrina*	*Citrina*	*P. hetheringtonii* Houbraken et al. 2010	CBS 122392 T	USA	beach soil	GU944558	GU944538	MN969263	JN606606
				CS01-09	China: Chongqing	soil in bamboo grove	**OQ870875**	**OR051077**	n.a.	**OR051428**
*Aspergilloides*	*Citrina*	*Citrina*	*P. sizovae* Baghd. 1968	CBS 413.69 T	Syria	Soil	GU944588	GU944535	MN969298	JN606603
*Aspergilloides*	*Citrina*	*Citrina*	*P. steckii* K.W. Zaleski 1927	CBS 260.55 T	Panama	cotton fabric treated with copper naphthenate	GU944597	GU944522	MN969300	JN606602
*Aspergilloides*	*Citrina*	*Citrina*	*P. tropicoides* Houbraken et al. 2010	CBS 122410 T	Thailand	soil of rainforest	GU944584	GU944531	MN969303	JN606608
*Aspergilloides*	*Citrina*	*Citrina*	*P. tropicum* Houbraken et al. 2010	CBS 112584 T	India	soil between *Coffea arabica*	GU944582	GU944532	MN969304	JN606607
*Aspergilloides*	*Citrina*	*Sumatraensia*	*P. cerradense* Cruvinel et al. 2021	DCFS6a T	Brazil	Soil	MT006126	MT416533	MT416534	MT416532
*Aspergilloides*	*Citrina*	*Sumatraensia*	*P. jenningsiae* Y.P. Tan et al. 2022	BRIP 45936a T	Australia	compost	n.a.	OL741657	n.a.	OL741660
*Aspergilloides*	*Citrina*	*Sumatraensia*		CS02-04	China: Chongqing	Soil	**OQ870876**	**OR051078**	**OR051255**	**OR051429**
*Aspergilloides*	*Citrina*	*Sumatraensia*		CS33-13	China: Sichuan	Soil	**OQ870877**	**OR051079**	**OR051256**	n.a.
*Aspergilloides*	*Citrina*	*Sumatraensia*	***P. qii*** X.C. Wang & W.Y. Zhuang, sp. nov.	CS18-09 = CGMCC 3.25165 T	China: Chongqing	Soil	**OQ870878**	**OR051080**	**OR051257**	**OR051430**
*Aspergilloides*	*Citrina*	*Sumatraensia*		CS18-05	China: Chongqing	Soil	**OQ870879**	**OR051081**	**OR051258**	n.a.
*Aspergilloides*	*Citrina*	*Sumatraensia*		CS18-27	China: Chongqing	soil	**OQ870880**	**OR051082**	**OR051259**	**OR051431**
*Aspergilloides*	*Citrina*	*Sumatraensia*	***P. rarum*** X.C. Wang & W.Y. Zhuang, sp. nov.	CS15-04 = CGMCC 3.25166 T	China: Chongqing	soil	**OQ870881**	**OR051083**	**OR051260**	**OR051432**
*Aspergilloides*	*Citrina*	*Sumatraensia*		CS15-05	China: Chongqing	soil	**OQ870882**	**OR051084**	**OR051261**	n.a.
*Aspergilloides*	*Citrina*	*Sumatraensia*		CS18-06	China: Chongqing	soil	**OQ870883**	**OR051085**	**OR051262**	**OR051433**
*Aspergilloides*	*Citrina*	*Sumatraensia*	*P. sumatraense* Svilv. 1936	CBS 281.36 T	Indonesia	heath soil	GU944578	JN606639	MN969301	EF198541
*Aspergilloides*	*Citrina*	*Sumatraensia*	***P. vulgatum*** X.C. Wang & W.Y. Zhuang, sp. nov.	CS15-03 = CGMCC 3.25180 T	China: Chongqing	soil	**OQ870884**	**OR051086**	**OR051263**	**OR051434**
*Aspergilloides*	*Citrina*	*Westlingiorum*	*P. aquadulcis* Hyang B. Lee & T.T.T. Nguyen 2021	CNUFC JT1301 T	South Korea	freshwater	OK356194	OK105100	OK105102	n.a.
*Aspergilloides*	*Citrina*	*Westlingiorum*	*P. atrofulvum* Houbraken et al. 2011	CBS 109.66 T	Democratic Republic of the Congo	soil	JN617663	JN606677	JN606387	JN606620
*Aspergilloides*	*Citrina*	*Westlingiorum*	*P. aurantiacobrunneum* Houbraken et al. 2011	CBS 126228 T	Denmark	air of cake factory	JN617670	JN606702	MN969238	MN969106
*Aspergilloides*	*Citrina*	*Westlingiorum*	*P. cairnsense* Houbraken et al. 2011	CBS 124325 T	Canada	soil	JN617669	JN606693	MN969240	MN969108
				CS25-11	China: Chongqing	soil	**OQ870885**	**OR051087**	**OR051264**	**OR051435**
*Aspergilloides*	*Citrina*	*Westlingiorum*	*P. christenseniae* Houbraken et al. 2011	CBS 126236 T	Costa Rica	forest soil	JN617674	JN606680	MN969243	JN606624
*Aspergilloides*	*Citrina*	*Westlingiorum*	*P. chrzaszczii* K.W. Zaleski 1927	CBS 217.28 T	Poland	woodland soil	GU944603	JN606758	MN969244	JN606628
*Aspergilloides*	*Citrina*	*Westlingiorum*	*P. cosmopolitanum* Houbraken et al. 2011	CBS 126995 T	The Netherlands	heathland soil	JN617691	JN606733	MN969249	MN969113
				CS11-04	China: Chongqing	soil in a cave	**OQ870886**	**OR051088**	**OR051265**	**OR051436**
*Aspergilloides*	*Citrina*	*Westlingiorum*	*P. decaturense* S.W. Peterson et al. 2004	CBS 117509 T	USA	old resupinate fungus	GU944604	JN606685	MN969252	JN606621
*Aspergilloides*	*Citrina*	*Westlingiorum*	*P. godlewskii* K.W. Zaleski 1927	CBS 215.28 T	Poland	soil under pine	JN617692	JN606768	MN969258	JN606626
*Aspergilloides*	*Citrina*	*Westlingiorum*	*P. manginii* Duché and R. Heim 1931	CBS 253.31 T	unknown	soil	GU944599	JN606651	MN969274	JN606618
*Aspergilloides*	*Citrina*	*Westlingiorum*	*P. miczynskii* K.W. Zaleski 1927	CBS 220.28 T	Poland	soil under conifer	GU944600	JN606706	MN969277	JN606623
*Aspergilloides*	*Citrina*	*Westlingiorum*	*P. neomiczynskii* A.L.J. Cole et al. 2011	CBS 126231 T	New Zealand	soil	JN617671	JN606705	MN969278	MN969128
*Aspergilloides*	*Citrina*	*Westlingiorum*	*P. nothofagi* Houbraken et al. 2011	CBS 130383 T	Chile	soil under *Nothofagus*	JN617712	JN606732	JN606507	MN969129
*Aspergilloides*	*Citrina*	*Westlingiorum*	*P. outeniquaense* Visagie & Yilmaz 2023	CBS 147414 T	South Africa	soil	MT949903	MT957405	MT957450	MT957476
*Aspergilloides*	*Citrina*	*Westlingiorum*	*P. pancosmium* Houbraken et al. 2011	CBS 276.75 T	Canada	old basidioma of *Armillaria mellea*	JN617660	JN606790	MN969284	MN969130
*Aspergilloides*	*Citrina*	*Westlingiorum*	*P. pasqualense* Houbraken et al. 2011	CBS 126330 T	Easter Is., Chile	soil	JN617676	JN606673	MN969286	JN606617
*Aspergilloides*	*Citrina*	*Westlingiorum*	*P. quebecense* Houbraken et al. 2011	CBS 101623 T	Canada	air in sawmill	JN617661	JN606700	JN606509	JN606622
*Aspergilloides*	*Citrina*	*Westlingiorum*	*P. raphiae* Houbraken et al. 2011	CBS 126234 T	Costa Rica	soil under *Raphia*	JN617673	JN606657	MN969292	JN606619
*Aspergilloides*	*Citrina*	*Westlingiorum*	*P. sucrivorum* Visagie & K. Jacobs 2014	CBS 135116 T	South Africa	mite inside infructescence of *Protea repens*	JX140872	JX141015	JX141506	MN969140
*Aspergilloides*	*Citrina*	*Westlingiorum*	*P. ubiquetum* Houbraken et al. 2011	CBS 126437 T	Costa Rica	soil	JN617680	JN606800	MN969306	MN969142
*Aspergilloides*	*Citrina*	*Westlingiorum*	*P. vancouverense* Houbraken 2011	CBS 126323 T	Canada	soil under maple	JN617675	JN606663	MN969307	MN969143
*Aspergilloides*	*Citrina*	*Westlingiorum*	*P. waksmanii* K.W. Zaleski 1927	CBS 230.28 T	Poland	woodland soil	GU944602	JN606779	MN969310	JN606627
*Aspergilloides*	*Citrina*	*Westlingiorum*	***P. wangwentsaii*** X.C. Wang & W.Y. Zhuang, sp. nov.	CS20-42 = CGMCC 3.25181 T	China: Chongqing	soil	**OQ870887**	**OR051089**	**OR051266**	**OR051437**
*Aspergilloides*	*Citrina*	*Westlingiorum*	*P. wellingtonense* A.L.J. Cole et al. 2011	CBS 130375 T	New Zealand	soil	JN617713	JN606670	MN969311	JN606616
*Aspergilloides*	*Citrina*	*Westlingiorum*	*P. westlingii* K.W. Zaleski 1927	CBS 231.28 T	Poland	soil under conifer	GU944601	JN606718	MN969312	JN606625
				CS10-16	China: Chongqing	soil	**OQ870888**	**OR051090**	**OR051267**	**OR051438**
*Aspergilloides*	*Citrina*	*Westlingiorum*	***P. wushanicum*** X.C. Wang & W.Y. Zhuang, sp. nov.	CS21-01 = CGMCC 3.25184 T	China: Chongqing	soil	**OQ870889**	**OR051091**	**OR051268**	**OR051439**
*Aspergilloides*	*Citrina*	*Gallaica*	*P. gallaicum* C. Ramírez et al. 1980	CBS 167.81 T	Spain	air	JN617690	JN606837	JN606548	JN606609

GenBank accession numbers in bold indicate the newly generated sequences. The phrase ‘n.a.’ is the abbreviation of ‘not available’.

**Table 4:** Species and sequences of *Penicillium* subgen. *Aspergilloides* sect. *Exilicaulis* used in phylogenetic analyses.

Subgenus	Section	Series	Species	Strain	Locality	Substrate	ITS	BenA	CaM	RPB2
*Aspergilloides*	*Exilicaulis*	*Lapidosa*	*P. aotearoae* Visagie & Seifert 2016	CBS 140999 T	New Zealand	clay	KT887874	KT887835	KT887796	MN969174
*Aspergilloides*	*Exilicaulis*	*Lapidosa*	*P. atrosanguineum* B.X. Dong 1973	CBS 380.75 T	Czech	seeds of *Triticum*	JN617706	KJ834435	KP016771	JN406557
*Aspergilloides*	*Exilicaulis*	*Lapidosa*	*P. burgense* Quintan. ex Visagie 2016	CBS 325.89 T	Spain	soil	KC411736	KJ834437	KP016772	JN406572
*Aspergilloides*	*Exilicaulis*	*Lapidosa*	*P. diabolicalicense* Visagie & Seifert 2016	CBS 140967 T	New Zealand	beneath moss and *Nothofagus*	KT887840	KT887801	KT887762	MN969175
*Aspergilloides*	*Exilicaulis*	*Lapidosa*	*P. hemitrachum* Visagie & K. Jacobs 2016	CBS 139134 T	South Africa	air	FJ231003	JX141048	JX157526	KP064642
*Aspergilloides*	*Exilicaulis*	*Lapidosa*	*P. lapidosum* Raper & Fennell 1948	CBS 343.48 T	USA	canned blueberries	MN431392	KJ834465	FJ530984	JN121500
*Aspergilloides*	*Exilicaulis*	*Lapidosa*	*P. maclennaniae* H.Y. Yip 1981	CBS 198.81 T	Australia	rhizoplane of *Gahnia radula*	KC411689	KJ834468	KP016791	KP064648
*Aspergilloides*	*Exilicaulis*	*Lapidosa*	*P. melinii* Thom 1930	CBS 218.30 T	USA	forest soil	AF033449	KJ834471	KP016792	JN406613
*Aspergilloides*	*Exilicaulis*	*Lapidosa*	*P. namyslowskii* K.W. Zaleski 1927	CBS 353.48 T	Poland	soil under *Pinus*	AF033463	JX141067	KP016795	JF417430
*Aspergilloides*	*Exilicaulis*	*Lapidosa*	*P. raciborskii* K.W. Zaleski 1927	CBS 224.28 T	Poland	soil under conifer	AF033447	JX141069	KP016800	JN406607
*Aspergilloides*	*Exilicaulis*	*Lapidosa*	*P. smithii* Quintan. 1982	CBS 276.83 T	Spain	*Secale cereale*	KC411723	KJ834492	KP016806	JN406589
				CS02-06	China: Chongqing	soil	**OQ867292**	**OR051092**	**OR051269**	**OR051440**
*Aspergilloides*	*Exilicaulis*	*Lapidosa*	*P. terrenum* D.B. Scott 1968	CBS 313.67 T	South Africa	soil	AM992111	KJ834496	KP016808	JN406577
*Aspergilloides*	*Exilicaulis*	*Lapidosa*	*P. velutinum* J.F.H. Beyma 1935	CBS 250.32 T	The Netherlands	sputum from man	AF033448	JX141170	MT478037	KP064682
*Aspergilloides*	*Exilicaulis*	*Lapidosa*	*P. xanthomelinii* Visagie & K. Jacobs 2016	CBS 139163 T	South Africa	soil	JX140921	JX141120	JX157495	KP064683
*Aspergilloides*	*Exilicaulis*	*Restricta*	*P. allaniae* Y.P. Tan et al. 2022	BRIP 74886a T	Australia	soil	OP903475	OP921956	OP921954	OP921955
*Aspergilloides*	*Exilicaulis*	*Restricta*	*P. arabicum* Baghd. 1968	CBS 414.69 T	Syria	soil	KC411758	KP016750	KP016770	KP064574
*Aspergilloides*	*Exilicaulis*	*Restricta*	*P. archerae* Y.P. Tan et al. 2022	BRIP 72549c T	Australia	soil	OP903477	OP921961	n.a.	OP921960
*Aspergilloides*	*Exilicaulis*	*Restricta*	*P. chalabudae* Visagie 2016	CBS 219.66 T	Ukraine	soil	KP016811	KP016748	KP016767	KP064572
*Aspergilloides*	*Exilicaulis*	*Restricta*	*P. cinereoatrum* Chalab. 1950	CBS 222.66 T	Ukraine	forest soil	KC411700	KJ834442	KP125335	JN406608
*Aspergilloides*	*Exilicaulis*	*Restricta*	***P. flemingii*** X.C. Wang & W.Y. Zhuang, sp. nov.	CS26-22 = CGMCC 3.25158 T	China: Chongqing	soil under a palm tree	**OQ867293**	**OR051093**	**OR051270**	**OR051441**
*Aspergilloides*	*Exilicaulis*	*Restricta*		CS26-45	China: Chongqing	soil under a palm tree	**OQ867294**	**OR051094**	**OR051271**	**OR051442**
*Aspergilloides*	*Exilicaulis*	*Restricta*		CS26-59	China: Chongqing	soil under a palm tree	**OQ867295**	**OR051095**	**OR051272**	n.a.
*Aspergilloides*	*Exilicaulis*	*Restricta*		CS26-80	China: Chongqing	soil under a palm tree	**OQ867296**	**OR051096**	**OR051273**	n.a.
*Aspergilloides*	*Exilicaulis*	*Restricta*		CS26-88	China: Chongqing	soil under a palm tree	**OQ867297**	**OR051097**	**OR051274**	**OR051443**
*Aspergilloides*	*Exilicaulis*	*Restricta*	*P. heteromorphum* H.Z. Kong & Z.T. Qi 1988	CBS 226.89 T	China: Hubei	soil	KC411702	KJ834455	KP016786	JN406605
*Aspergilloides*	*Exilicaulis*	*Restricta*	*P. katangense* Stolk 1968	CBS 247.67 T	Congo	soil	AF033458	KP016757	KP016788	KP064646
*Aspergilloides*	*Exilicaulis*	*Restricta*	*P. krskae* Labuda et al. 2021	CBS 147776 T	Austria	culture contaminant	MW794123	MW774594	MW774595	MW774593
*Aspergilloides*	*Exilicaulis*	*Restricta*	*P. kurssanovii* Chalab. 1950	CBS 625.67 T	Ukraine	maize-field soil	EF422849	KP016758	KP016789	KP064647
*Aspergilloides*	*Exilicaulis*	*Restricta*	*P. meridianum* D.B. Scott 1968	CBS 314.67 T	South Africa	soil	AF033451	KJ834472	KP016794	JN406576
*Aspergilloides*	*Exilicaulis*	*Restricta*	*P. philippinense* Udagawa & Y. Horie 1972	CBS 623.72 T	Philippines	soil	KC411770	KJ834482	KP016799	JN406543
*Aspergilloides*	*Exilicaulis*	*Restricta*	*P. restrictum* J.C. Gilman & E.V. Abbott 1927	CBS 367.48 T	Honduras	soil	AF033457	KJ834486	KP016803	JN121506
*Aspergilloides*	*Exilicaulis*	*Restricta*	*P. silybi* Labuda et al. 2021	CBS 147777 T	USA	*Silybum marianum*	KF367458	MW774592	MW774591	AB860248
*Aspergilloides*	*Exilicaulis*	*Alutacea*	*P. alutaceum* D.B. Scott 1968	CBS 317.67 T	South Africa	soil	AF033454	KJ834430	KP016768	JN121489

GenBank accession numbers in bold indicate the newly generated sequences. The phrase ‘n.a.’ is the abbreviation of ‘not available’.

**Table 5:** Species and sequences of *Penicillium* subgen. *Aspergilloides* sect. *Gracilenta* used in phylogenetic analyses.

Subgenus	Section	Series	Species	Strain	Locality	Substrate	ITS	BenA	CaM	RPB2
*Aspergilloides*	*Gracilenta*	*Angustiporcata*	*P. angustiporcatum* Takada & Udagawa 1983	CBS 202.84 T	Nepal	soil	KC411690	KJ834431	MN969235	JN406617
*Aspergilloides*	*Gracilenta*	*Estinogena*	***P. chongqingense*** X.C. Wang & W.Y. Zhuang, sp. nov.	CS03-01 = CGMCC 3.25150 T	China: Chongqing	soil	**OQ870822**	**OR051098**	**OR051275**	**OR051444**
				CS03-02	China: Chongqing	soil	**OQ870823**	**OR051099**	**OR051276**	**OR051445**
				CS03-08	China: Chongqing	soil	**OQ870824**	**OR051100**	**OR051277**	**OR051446**
*Aspergilloides*	*Gracilenta*	*Estinogena*	*P. estinogenum* A. Komatsu & S. Abe ex G. Sm. 1963	CBS 329.59 T	Japan	soil	MN431388	MN969381	MN969255	n.a.
*Aspergilloides*	*Gracilenta*	*Estinogena*	*P. guarroi* Torres-Garcia et al. 2022	FMR 17747 T	Spain	fluvial sediment	LR814139	LR814134	LR814140	LR814145
*Aspergilloides*	*Gracilenta*	*Estinogena*	***P. sichuanense*** X.C. Wang & W.Y. Zhuang, sp. nov.	CS32-04 = CGMCC 3.25169 T	China: Sichuan	soil of ant hole	**OQ870825**	**OR051101**	**OR051278**	**OR051447**
*Aspergilloides*	*Gracilenta*	*Gracilenta*	*P. gracilentum* Udagawa & Y. Horie 1973	CBS 599.73 T	Papua New Guinea	soil	KC411768	KJ834453	MN969260	JN121537
*Aspergilloides*	*Gracilenta*	*Macrosclerotiorum*	*P. apimei* R.N. Barbosa et al. 2018	CBS 142502 T	Brazil	honey	MF278310	LT854641	LT882717	LT854650
*Aspergilloides*	*Gracilenta*	*Macrosclerotiorum*	*P. aquaticum* Hyang B. Lee et al. 2018	CNUFC YSW8-1 T	South Korea	plant debris in freshwater	KY587453	KY587450	KY587447	KY587449
*Aspergilloides*	*Gracilenta*	*Macrosclerotiorum*	***P. johnpittii*** X.C. Wang & W.Y. Zhuang, sp. nov.	CS23-04 = CGMCC 3.25163 T	China: Chongqing	soil	**OQ870826**	**OR051102**	**OR051279**	**OR051448**
				CS33-02	China: Sichuan	soil	**OQ870827**	**OR051103**	**OR051280**	**OR051449**
*Aspergilloides*	*Gracilenta*	*Macrosclerotiorum*	*P. macrosclerotiorum* L. Wang et al. 2007	CGMCC 3.6581 T	China: Chongqing	soil	KJ834511	KJ834469	AY678538	JN121432
*Aspergilloides*	*Stolkia*	*Stolkia*	*P. stolkiae* D.B. Scott 1968	CBS 315.67 T	South Africa	soil	AF033444	JN617717	AF481135	JN121488

GenBank accession numbers in bold indicate the newly generated sequences. The phrase ‘n.a.’ is the abbreviation of ‘not available’.

**Table 6:** Species and sequences of *Penicillium* subgen. *Aspergilloides* sect. *Lanata-Divaricata* used in phylogenetic analyses.

Subgenus	Section	Series	Species	Strain	Locality	Substrate	ITS	BenA	CaM	RPB2
*Aspergilloides*	*Lanata-Divaricata*	*Dalearum*	*P. abidjanum* Stolk 1968	CBS 246.67 T	Côte d’Ivoire	soil	GU981582	GU981650	MN969234	JN121469
*Aspergilloides*	*Lanata-Divaricata*	*Dalearum*	*P. amphipolaria* Visagie et al. 2016	CBS 140997 T	Antarctica	soil	KT887872	KT887833	KT887794	MN969177
*Aspergilloides*	*Lanata-Divaricata*	*Dalearum*	*P. ausonanum* Torres-Garcia et al. 2022	FMR 16948 T	Spain	fluvial sediment	LR655808	LR655809	LR655810	LR655811
*Aspergilloides*	*Lanata-Divaricata*	*Dalearum*	*P. austrosinense* L. Cai et al. 2018	CGMCC 3.18797 T	China: Hainan	acidic soil	KY495007	KY495116	MN969328	KY495061
*Aspergilloides*	*Lanata-Divaricata*	*Dalearum*	*P. daleae* K.W. Zaleski 1927	CBS 211.28 T	Poland	soil under conifer	GU981583	GU981649	MN969251	KF296427
				CS01-10	China: Chongqing	soil in bamboo grove	**OQ870719**	**OR051104**	**OR051281**	**OR051450**
				CS01-11	China: Chongqing	soil in bamboo grove	**OQ870720**	**OR051105**	**OR051282**	**OR051451**
				CS03-03	China: Chongqing	soil	**OQ870721**	**OR051106**	**OR051283**	**OR051452**
				CS05-01	China: Chongqing	soil	**OQ870722**	**OR051107**	**OR051284**	**OR051453**
				CS09-02	China: Chongqing	soil	**OQ870723**	**OR051108**	**OR051285**	**OR051454**
				CS12-09	China: Chongqing	soil	**OQ870724**	**OR051109**	**OR051286**	**OR051455**
*Aspergilloides*	*Lanata-Divaricata*	*Dalearum*	*P. griseopurpureum* G. Sm. 1965	CBS 406.65 T	UK	soil under Coniferae	KF296408	KF296467	MN969261	KF296431
				CS24-03	China: Chongqing	soil under *Larix* sp.	**OQ870725**	**OR051110**	**OR051287**	**OR051456**
*Aspergilloides*	*Lanata-Divaricata*	*Dalearum*	*P. guaibinense* J.P. Andrade et al. 2018	CCDCA 11512 T	Brazil	soil	MH674389	MH674391	MH674393	n.a.
*Aspergilloides*	*Lanata-Divaricata*	*Dalearum*	*P. jianfenglingense* L. Cai & X.Z. Jiang 2018	CGMCC 3.18802 T	China: Hainan	acidic soil	KY495016	KY495125	MN969334	KY495069
*Aspergilloides*	*Lanata-Divaricata*	*Dalearum*	***P. pauciramulum*** X.C. Wang & W.Y. Zhuang, sp. nov.	CS04-09 = CGMCC 3.25164 T	China: Chongqing	ant hole soil	**OQ870726**	**OR051111**	**OR051288**	**OR051457**
				CS04-10	China: Chongqing	ant hole soil	**OQ870727**	**OR051112**	**OR051289**	**OR051458**
				CS04-11	China: Chongqing	ant hole soil	**OQ870728**	**OR051113**	**OR051290**	**OR051459**
*Aspergilloides*	*Lanata-Divaricata*	*Dalearum*	*P. penarojense* Houbraken et al. 2011	CBS 113178 T	Colombia	leaf litter	GU981570	GU981646	MN969287	KF296450
*Aspergilloides*	*Lanata-Divaricata*	*Dalearum*	*P. rubriannulatum* L. Cai et al. 2018	CGMCC 3.18804 T	China: Hainan	acidic soil	KY495029	KY495138	MN969336	KY495080
*Aspergilloides*	*Lanata-Divaricata*	*Dalearum*	*P. singorense* Visagie et al. 2014	CBS 138214 T	Thailand	house dust	KJ775674	KJ775167	KJ775403	MN969138
*Aspergilloides*	*Lanata-Divaricata*	*Dalearum*	*P. stangiae* A.L. Alves & P.V. Tiago 2022	URM 8347 T	Brazil	forest soil	MW648590	MW646388	MW646390	MW646392
*Aspergilloides*	*Lanata-Divaricata*	*Dalearum*	*P. vanderhammenii* Houbraken et al. 2011	CBS 126216 T	Colombia	leaf litter	GU981574	GU981647	MN969308	KF296458
*Aspergilloides*	*Lanata-Divaricata*	*Dalearum*	*P. viridissimum* L. Cai & X.Z. Jiang 2018	CGMCC 3.18796 T	China: Hainan	acidic soil	KY495004	KY495113	MN969339	KY495059
				CS03-05	China: Chongqing	soil	**OQ870729**	**OR051114**	**OR051291**	**OR051460**
				CS08-03	China: Chongqing	soil	**OQ870730**	**OR051115**	**OR051292**	**OR051461**
*Aspergilloides*	*Lanata-Divaricata*	*Dalearum*	*P. zonatum* Hodges & T.J. Perry 1973	CBS 992.72 T	USA	soil	GU981581	GU981651	MN969315	KF296461
*Aspergilloides*	*Lanata-Divaricata*	*Janthinella*	*P. brefeldianum* B.O. Dodge 1933	CBS 235.81 T	North America	human alimentary tract	AF033435	GU981623	EU021683	KF296421
*Aspergilloides*	*Lanata-Divaricata*	*Janthinella*	*P. caperatum* Udagawa & Y. Horie 1973	CBS 443.75 T	Papua New Guinea	soil	KC411761	GU981660	MN969242	KF296422
*Aspergilloides*	*Lanata-Divaricata*	*Janthinella*	*P. cluniae* Quintan. 1990	CBS 326.89 T	Spain	soil	MN431386	MN969376	MN969246	KF296424
*Aspergilloides*	*Lanata-Divaricata*	*Janthinella*	*P. coeruleum* Sopp 1923	CBS 141.45 T	unknown	unknown	GU981606	GU981655	MN969247	KF296425
*Aspergilloides*	*Lanata-Divaricata*	*Janthinella*	*P. cremeogriseum* Chalab. 1950	CBS 223.66 T	Ukraine	forest soil	GU981586	GU981624	MN969250	KF296426
				CS09-07	China: Chongqing	soil	**OQ870731**	**OR051116**	**OR051293**	**OR051462**
*Aspergilloides*	*Lanata-Divaricata*	*Janthinella*	*P. curticaule* Visagie & K. Jacobs 2015	CBS 135127 T	South Africa	sandveld fynbos soil	FJ231021	JX091526	JX141536	KF296417
*Aspergilloides*	*Lanata-Divaricata*	*Janthinella*	*P. donggangicum* L.Wang 2022	CGMCC 3.15900 T	China: Liaoning	soil of tidal flats	MW946996	MZ004914	MZ004918	MW979253
*Aspergilloides*	*Lanata-Divaricata*	*Janthinella*	*P. ehrlichii* Kleb. 1930	CBS 324.48 T	Poland	unknown	GU981578	GU981652	MN969253	KF296428
*Aspergilloides*	*Lanata-Divaricata*	*Janthinella*	*P. elleniae* Houbraken et al. 2011	CBS 118135 T	Colombia	leaf litter	GU981612	GU981663	MN969254	KF296429
*Aspergilloides*	*Lanata-Divaricata*	*Janthinella*	*P. glaucoroseum* Demelius 1923	CBS 138908 T	USA	soil	MN431390	MN969383	MN969257	MN969119
*Aspergilloides*	*Lanata-Divaricata*	*Janthinella*	*P. janthinellum* Biourge 1923	CBS 340.48 T	Nicaragua	soil	GU981585	GU981625	MN969268	JN121497
*Aspergilloides*	*Lanata-Divaricata*	*Janthinella*	*P. javanicum* J.F.H. Beyma 1929	CBS 341.48 T	Indonesia	root of *Camellia sinensis*	GU981613	GU981657	MN969269	JN121498
				CS23-07	China: Chongqing	soil	**OQ870732**	**OR051117**	**OR051294**	**OR051463**
				CS23-40	China: Chongqing	soil	**OQ870733**	**OR051118**	**OR051295**	**OR051464**
*Aspergilloides*	*Lanata-Divaricata*	*Janthinella*	*P. koreense* S.B. Hong et al. 2014	CBS 141338 T	South Korea	bamboo field soil	KJ801939	KM000846	MN969317	MN969159
				CS19-06	China: Chongqing	soil	**OQ870734**	**OR051119**	**OR051296**	n.a.
*Aspergilloides*	*Lanata-Divaricata*	*Janthinella*	*P. levitum* Raper & Fennell 1948	CBS 345.48 T	USA	modelling clay	GU981607	GU981654	MN969270	KF296432
*Aspergilloides*	*Lanata-Divaricata*	*Janthinella*	*P. limosum* S. Ueda 1995	CBS 339.97 T	Japan	marine sediment	GU981568	GU981621	MN969271	KF296433
*Aspergilloides*	*Lanata-Divaricata*	*Janthinella*	*P. lineolatum* Udagawa & Y. Horie 1977	CBS 188.77 T	Japan	soil	GU981579	GU981620	MN969272	KF296434
*Aspergilloides*	*Lanata-Divaricata*	*Janthinella*	*P. ludwigii* Udagawa 1969	CBS 417.68 T	Japan	polished seed of *Oryza sativa*	KF296409	KF296468	MN969273	KF296435
*Aspergilloides*	*Lanata-Divaricata*	*Janthinella*	*P. malacosphaerulum* Visagie & K. Jacobs 2015	CBS 135120 T	South Africa	sandveld fynbos soil	FJ231026	JX091524	JX141542	KF296438
*Aspergilloides*	*Lanata-Divaricata*	*Janthinella*	*P. melanosporum* Rodr.-Andr. et al. 2021	FMR 17424 T	Spain	soil	LR655192	LR655196	LR655200	LR655204
*Aspergilloides*	*Lanata-Divaricata*	*Janthinella*	*P. meloforme* Udagawa & Y. Horie 1973	CBS 445.74 T	Papua New Guinea	soil	KC411762	GU981656	MN969276	KF296440
*Aspergilloides*	*Lanata-Divaricata*	*Janthinella*	*P. michoacanense* Rodr.-Andr. et al. 2021	FMR 17612 T	Mexico	soil	LR655194	LR655198	LR655202	LR655206
*Aspergilloides*	*Lanata-Divaricata*	*Janthinella*	*P. nordestinense* J.E.F. Santos & R.N. Barbosa 2022	URM 8423 T	Brazil	pollen	OV265270	OV265324	OV265272	OM927721
*Aspergilloides*	*Lanata-Divaricata*	*Janthinella*	*P. ortum* Visagie & K. Jacobs 2015	CBS 135669 T	South Africa	soil	JX091427	JX091520	JX141551	KF296443
*Aspergilloides*	*Lanata-Divaricata*	*Janthinella*	*P. raperi* G. Sm. 1957	CBS 281.58 T	UK	soil in field of *Brassica oleracea*	AF033433	GU981622	MN969291	KF296453
*Aspergilloides*	*Lanata-Divaricata*	*Janthinella*	*P. reticulisporum* Udagawa 1968	CBS 122.68 T	Japan	soil	AF033437	MN969394	MN969293	KF296454
*Aspergilloides*	*Lanata-Divaricata*	*Janthinella*	*P. setosum* T.K. George et al. 2022	CBS 144865 T	India	*Withania somnifera*	KT852579	MF184995	MH105905	n.a.
*Aspergilloides*	*Lanata-Divaricata*	*Janthinella*	*P. siccitolerans* Rodr.-Andr. et al. 2021	FMR 17381 T	Spain	soil	LR655193	LR655197	LR655201	LR655205
*Aspergilloides*	*Lanata-Divaricata*	*Janthinella*	*P. soli* Doilom et al. 2020	KUMCC 18-0202 T	China: Yunnan	rhizosphere soil of *Quercus rubra*	MT152337	MT161681	MT178249	MT384372
*Aspergilloides*	*Lanata-Divaricata*	*Janthinella*	***P. tengii*** X.C. Wang & W.Y. Zhuang, sp. nov.	CS27-03 = CGMCC 3.25179 T	China: Chongqing	soil	**OQ870735**	**OR051120**	**OR051297**	**OR051465**
*Aspergilloides*	*Lanata-Divaricata*	*Janthinella*	*P. uruguayense* Guevara-Suarez et al. 2017	CBS 143247 T	Uruguay	soil	LT904729	LT904699	LT904698	MN969200
*Aspergilloides*	*Lanata-Divaricata*	*Janthinella*	*P. yunnanense* L. Cai & X.Z. Jiang 2018	CGMCC 3.18794 T	China: Yunnan	acidic soil	KY494990	KY495099	MN969340	KY495048
*Aspergilloides*	*Lanata-Divaricata*	*Rolfsiorum*	*P. annulatum* Visagie & K. Jacobs 2015	CBS 135126 T	South Africa	air	JX091426	JX091514	JX141545	KF296410
*Aspergilloides*	*Lanata-Divaricata*	*Rolfsiorum*	*P. bissettii* Visagie & Seifert 2016	CBS 140972 T	Canada	soil of *Picea* forest	KT887845	KT887806	KT887767	MN969178
*Aspergilloides*	*Lanata-Divaricata*	*Rolfsiorum*	*P. camponotum* Visagie et al. 2016	CBS 140982 T	Canada	*Camponotus pennsylvanicus*	KT887855	KT887816	KT887777	MN969179
*Aspergilloides*	*Lanata-Divaricata*	*Rolfsiorum*	***P. coffeatum*** X.C. Wang & W.Y. Zhuang, sp. nov.	CS10-15 = CGMCC 3.25152 T	China: Chongqing	soil	**OQ870815**	**OR051121**	**OR051298**	**OR051466**
*Aspergilloides*	*Lanata-Divaricata*	*Rolfsiorum*	*P. excelsum* Taniwaki et al. 2015	IBT 31516 T	Brazil	shell of *Bertholletia excelsa*	KR815341	KP691061	KR815342	MN969166
*Aspergilloides*	*Lanata-Divaricata*	*Rolfsiorum*	*P. flaviroseum* L. Cai & X.Z. Jiang 2018	CGMCC 3.18805 T	China: Hainan	acidic soil	KY495032	KY495141	MN969329	KY495083
*Aspergilloides*	*Lanata-Divaricata*	*Rolfsiorum*	*P. fructuariae-cellae* M. Lorenzini et al. 2019	CBS 145110 T	Italy	grape berry	MK039434	KU554679	MK045337	n.a.
*Aspergilloides*	*Lanata-Divaricata*	*Rolfsiorum*	*P. hainanense* L. Cai & X.Z. Jiang 2018	CGMCC 3.18798 T	China: Hainan	acidic soil	KY495009	KY495118	MN969333	KY495062
*Aspergilloides*	*Lanata-Divaricata*	*Rolfsiorum*	*P. ochrochloron* Biourge 1923	CBS 357.48 T	USA	copper sulphate solution	GU981604	GU981672	MN969280	KF296445
				CS05-02	China: Chongqing	soil	**OQ870736**	**OR051122**	**OR051299**	**OR051467**
				CS05-05	China: Chongqing	soil	**OQ870737**	**OR051123**	**OR051300**	**OR051468**
				CS07-01	China: Chongqing	soil	**OQ870738**	**OR051124**	**OR051301**	**OR051469**
				CS07-02	China: Chongqing	soil	**OQ870739**	**OR051125**	**OR051302**	**OR051470**
				CS08-02	China: Chongqing	soil	**OQ870740**	**OR051126**	**OR051303**	n.a.
				CS08-04	China: Chongqing	soil	**OQ870741**	**OR051127**	**OR051304**	**OR051471**
				CS09-01	China: Chongqing	soil	**OQ870742**	**OR051128**	**OR051305**	**OR051472**
				CS09-05	China: Chongqing	soil	n.a.	**OR051129**	**OR051306**	n.a.
				CS10-07	China: Chongqing	soil	**OQ870743**	**OR051130**	**OR051307**	**OR051473**
				CS10-14	China: Chongqing	soil	**OQ870744**	**OR051131**	**OR051308**	**OR051474**
				CS13-10	China: Chongqing	soil	**OQ870745**	**OR051132**	**OR051309**	**OR051475**
				CS22-01	China: Chongqing	soil	**OQ870746**	**OR051133**	**OR051310**	**OR051476**
*Aspergilloides*	*Lanata-Divaricata*	*Rolfsiorum*	*P. piscarium* Westling 1911	CBS 362.48 T	Germany	cod-liver oil emulsion	GU981600	GU981668	MN969288	KF296451
*Aspergilloides*	*Lanata-Divaricata*	*Rolfsiorum*	*P. pulvillorum* Turfitt 1939	CBS 280.39 T	UK	soil	AF178517	GU981670	MN969289	KF296452
*Aspergilloides*	*Lanata-Divaricata*	*Rolfsiorum*	*P. rolfsii* Thom 1930	CBS 368.48 T	North America	fruit of *Ananas sativus*	JN617705	GU981667	MN969294	KF296455
*Aspergilloides*	*Lanata-Divaricata*	*Rolfsiorum*	*P. rotoruae* O’Callahan & Vaidya 2020	CBS 145838 T	New Zealand	*Pinus radiata* timber stake	MN315103	MN315104	MN315102	MT240842
*Aspergilloides*	*Lanata-Divaricata*	*Rolfsiorum*	*P. soliforme* L. Cai et al. 2018	CGMCC 3.18806 T	China: Hainan	acidic soil	KY495038	KY495147	MN969337	KY495047
				CS02-03	China: Chongqing	soil	**OQ870747**	**OR051134**	**OR051311**	**OR051477**
				CS05-07	China: Chongqing	soil	**OQ870748**	**OR051135**	**OR051312**	**OR051478**
				CS09-03	China: Chongqing	soil	**OQ870749**	**OR051136**	**OR051313**	n.a.
*Aspergilloides*	*Lanata-Divaricata*	*Rolfsiorum*	*P. subrubescens* Houbraken et al. 2013	CBS 132785 T	Finland	soil of *Helianthus tuberosus* field	KC346350	KC346327	KC346330	KC346306
*Aspergilloides*	*Lanata-Divaricata*	*Rolfsiorum*	***P. subrutilans*** X.C. Wang & W.Y. Zhuang, sp. nov.	CS20-14 = CGMCC 3.25174 T	China: Chongqing	soil	**OQ870816**	**OR051137**	**OR051314**	**OR051479**
				CS20-27	China: Chongqing	soil	**OQ870817**	**OR051138**	**OR051315**	n.a.
				CS20-58	China: Chongqing	soil	**OQ870818**	**OR051139**	**OR051316**	**OR051480**
*Aspergilloides*	*Lanata-Divaricata*	*Rolfsiorum*	*P. svalbardense* Frisvad et al. 2007	CBS 122416 T	Norway: Svalbard	glacial ice	GU981603	DQ486644	KC346338	KF296457
*Aspergilloides*	*Lanata-Divaricata*	*Rolfsiorum*	*P. terrarumae* Houbraken et al. 2016	CBS 131811 T	China: Guizhou	soil contaminated by heavy metals	MN431397	KX650295	MN969323	MN969185
				CS14-17	China: Chongqing	soil	**OQ870750**	**OR051140**	**OR051317**	n.a.
				CS23-08	China: Chongqing	soil	**OQ870751**	**OR051141**	**OR051318**	**OR051481**
				CS23-26	China: Chongqing	soil	**OQ870752**	**OR051142**	**OR051319**	n.a.
				CS23-48	China: Chongqing	soil	**OQ870753**	**OR051143**	**OR051320**	n.a.
*Aspergilloides*	*Lanata-Divaricata*	*Rolfsiorum*	*P. vasconiae* C. Ramírez & A.T. Martínez 1980	CBS 339.79 T	Spain	soil	GU981599	GU981653	MN969309	MN969144
				CS20-23	China: Chongqing	soil	**OQ870819**	**OR051144**	**OR051321**	**OR051482**
*Aspergilloides*	*Lanata-Divaricata*	*Simplicissima*	*P. alagoense* L.O. Ferro et al. 2019	URM 8086 T	Brazil	leaf endophyte of *Miconia*	MK804503	MK802333	MK802336	MK802338
*Aspergilloides*	*Lanata-Divaricata*	*Simplicissima*	*P. araracuaraense* Houbraken et al. 2011	CBS 113149 T	Colombia	leaf litter	GU981597	GU981642	MN969237	KF296414
*Aspergilloides*	*Lanata-Divaricata*	*Simplicissima*	*P. brasilianum* Bat. 1957	CBS 253.55 T	Brazil	herbarium exsiccata	GU981577	GU981629	MN969239	KF296420
				CS13-08	China: Chongqing	soil	**OQ870754**	**OR051145**	**OR051322**	**OR051483**
				CS13-12	China: Chongqing	soil	**OQ870755**	**OR051146**	**OR051323**	**OR051484**
				CS17-03	China: Chongqing	soil	**OQ870756**	**OR051147**	**OR051324**	n.a.
				CS20-08	China: Chongqing	soil	**OQ870757**	**OR051148**	**OR051325**	n.a.
				CS24-02	China: Chongqing	soil under *Larix* sp.	**OQ870758**	**OR051149**	**OR051326**	**OR051485**
				CS26-24	China: Chongqing	soil under a palm tree	**OQ870759**	**OR051150**	**OR051327**	n.a.
				CS26-53	China: Chongqing	soil under a palm tree	**OQ870760**	**OR051151**	**OR051328**	n.a.
				CS26-56	China: Chongqing	soil under a palm tree	**OQ870761**	**OR051152**	**OR051329**	n.a.
				CS28-06	China: Chongqing	soil	**OQ870762**	**OR051153**	**OR051330**	**OR051486**
				CS28-07	China: Chongqing	soil	**OQ870763**	**OR051154**	**OR051331**	**OR051487**
				CS32-03	China: Sichuan	soil of ant hole	**OQ870764**	**OR051155**	**OR051332**	**OR051488**
*Aspergilloides*	*Lanata-Divaricata*	*Simplicissima*	*P. cataractum* Visagie et al. 2016	CBS 140974 T	Canada	nuts of *Carya cordiformis*	KT887847	KT887808	KT887769	MN969180
*Aspergilloides*	*Lanata-Divaricata*	*Simplicissima*	*P. echinulonalgiovense* S. Abe ex Houbraken & R.N. Barbosa 2018	CBS 328.59 T	Japan	soil	GU981587	GU981631	KX961269	KX961301
*Aspergilloides*	*Lanata-Divaricata*	*Simplicissima*	***P. fengjieense*** X.C. Wang & W.Y. Zhuang, sp. nov.	CS15-01 = CGMCC 3.25157 T	China: Chongqing	soil	**OQ870765**	**OR051156**	**OR051333**	**OR051489**
*Aspergilloides*	*Lanata-Divaricata*	*Simplicissima*	*P. globosum* L. Cai et al. 2018	CGMCC 3.18800 T	China: Hainan	acidic soil	KY495014	KY495123	MN969330	KY495067
*Aspergilloides*	*Lanata-Divaricata*	*Simplicissima*	*P. griseoflavum* L. Cai & X.Z. Jiang 2018	CGMCC 3.18799 T	China: Hainan	acidic soil	KY495011	KY495120	MN969331	KY495064
*Aspergilloides*	*Lanata-Divaricata*	*Simplicissima*	*P. guangxiense* L. Cai & X.Z. Jiang 2018	CGMCC 3.18793 T	China: Guangxi	soil	KY494986	KY495095	MN969332	KY495045
*Aspergilloides*	*Lanata-Divaricata*	*Simplicissima*	*P. infrabuccalum* Visagie et al. 2016	CBS 140983 T	Canada	*Camponotus pennsylvanicus*	KT887856	KT887817	KT887778	MN969181
*Aspergilloides*	*Lanata-Divaricata*	*Simplicissima*	***P. jinyunshanicum*** X.C. Wang & W.Y. Zhuang, sp. nov.	CS02-01 = CGMCC 3.25162 T	China: Chongqing	soil	**OQ870766**	**OR051157**	**OR051334**	**OR051490**
				CS02-02	China: Chongqing	soil	**OQ870767**	**OR051158**	**OR051335**	**OR051491**
				CS02-10	China: Chongqing	soil	**OQ870768**	**OR051159**	**OR051336**	**OR051492**
				CS03-06	China: Chongqing	soil	**OQ870769**	**OR051160**	**OR051337**	n.a.
				CS03-07	China: Chongqing	soil	**OQ870770**	**OR051161**	**OR051338**	**OR051493**
*Aspergilloides*	*Lanata-Divaricata*	*Simplicissima*	*P. laevigatum* L. Cai et al. 2018	CGMCC 3.18801 T	China: Hainan	acidic soil	KY495015	KY495124	MN969335	KY495068
*Aspergilloides*	*Lanata-Divaricata*	*Simplicissima*	*P. mariae-crucis* Quintan. 1982	CBS 271.83 T	Spain	*Secale cereale*	GU981593	GU981630	MN969275	KF296439
*Aspergilloides*	*Lanata-Divaricata*	*Simplicissima*	*P. newtonturnerae* Y.P. Tan et al. 2022	BRIP 74909a T	Australia	soil	OP903478	OP921964	OP921962	OP921963
*Aspergilloides*	*Lanata-Divaricata*	*Simplicissima*	*P. onobense* C. Ramírez & A.T. Martínez 1981	CBS 174.81 T	Spain	andosol	GU981575	GU981627	MN969281	KF296447
*Aspergilloides*	*Lanata-Divaricata*	*Simplicissima*	*P. panissanguineum* Visagie et al. 2016	CBS 140989 T	Tanzania	soil near termite mound	KT887862	KT887823	KT887784	MN969182
*Aspergilloides*	*Lanata-Divaricata*	*Simplicissima*	*P. paraherquei* S. Abe ex G. Sm. 1963	CBS 338.59 T	Japan	soil	AF178511	KF296465	MN969285	KF296449
				CS20-07	China: Chongqing	soil	**OQ870771**	**OR051162**	**OR051339**	**OR051494**
				CS20-15	China: Chongqing	soil	**OQ870772**	**OR051163**	**OR051340**	n.a.
				CS20-20	China: Chongqing	soil	**OQ870773**	**OR051164**	**OR051341**	n.a.
				CS20-21	China: Chongqing	soil	**OQ870774**	**OR051165**	**OR051342**	n.a.
				CS20-25	China: Chongqing	soil	**OQ870775**	**OR051166**	**OR051343**	n.a.
				CS20-31	China: Chongqing	soil	**OQ870776**	**OR051167**	**OR051344**	n.a.
				CS20-80	China: Chongqing	soil	**OQ870777**	**OR051168**	**OR051345**	n.a.
				CS21-05	China: Chongqing	soil	n.a.	**OR051169**	**OR051346**	**OR051495**
*Aspergilloides*	*Lanata-Divaricata*	*Simplicissima*	*P. pedernalense* Laich & J. Andrade 2018	CBS 140770 T	Ecuador	*Litopenaeus vannamei*	KU255398	KU255396	MN969322	MN969184
*Aspergilloides*	*Lanata-Divaricata*	*Simplicissima*	*P. simplicissimum* (Oudem.) Thom 1930	CBS 372.48 T	South Africa	flannel bag	GU981588	GU981632	MN969297	JN121507
*Aspergilloides*	*Lanata-Divaricata*	*Simplicissima*	*P. skrjabinii* Schmotina & Golovleva 1974	CBS 439.75 T	Russia	soil	GU981576	GU981626	MN969299	EU427252
*Aspergilloides*	*Lanata-Divaricata*	*Simplicissima*	*P. spinuliferum* L. Cai & X.Z. Jiang 2018	CGMCC 3.18807 T	China: Hainan	acidic soil with *Litchi chinensis*	KY495040	KY495149	MN969338	KY495090
*Aspergilloides*	*Lanata-Divaricata*	*Simplicissima*	*P. subfuscum* Visagie & Yilmaz 2023	CBS 147455 T	South Africa	soil	MT949907	MT957412	MT957454	MT957480
*Aspergilloides*	*Lanata-Divaricata*	*Simplicissima*	***P. taii*** X.C. Wang & W.Y. Zhuang, sp. nov.	CS16-09 = CGMCC 3.25176 T	China: Chongqing	soil	**OQ870778**	**OR051170**	**OR051347**	**OR051496**
				CS30-11	China: Sichuan	soil	**OQ870779**	**OR051171**	**OR051348**	**OR051497**
*Aspergilloides*	*Lanata-Divaricata*	*Simplicissima*	*P. tanzanicum* Visagie et al. 2016	CBS 140968 T	Tanzania	soil near termite mound	KT887841	KT887802	KT887763	MN969183
*Aspergilloides*	*Lanata-Divaricata*	*Simplicissima*	*P. uttarakhandense* Rajeshk. et al. 2021	NFCCI 4808 T	India	garden soil	MN967315	MN972443	MN972445	MN972447
				CS24-01	China: Chongqing	soil under *Larix* sp.	**OQ870780**	**OR051172**	**OR051349**	**OR051498**
				CS20-36	China: Chongqing	soil	**OQ870781**	**OR051173**	**OR051350**	n.a.
				CS24-06	China: Chongqing	soil under *Larix* sp.	**OQ870782**	**OR051174**	**OR051351**	n.a.
*Aspergilloides*	*Lanata-Divaricata*	*Simplicissima*	*P. vickeryae* Y.P. Tan & R.G. Shivas 2022	BRIP 72552a T	Australia	soil	OP903479	OP921966	n.a.	OP921965
*Aspergilloides*	*Lanata-Divaricata*	*Simplicissima*	*P. wandoense* Hyang B. Lee et al. 2019	CNUFC-WT31-1 T	South Korea	freshwater	n.a.	MK080564	MK080566	MK080562
*Aspergilloides*	*Lanata-Divaricata*	*Simplicissima*	*P. wotroi* Houbraken et al. 2011	CBS 118171 T	Colombia	leaf litter	GU981591	GU981637	MN969313	KF296460
*Aspergilloides*	*Lanata-Divaricata*	*Simplicissima*	***P. yuyongnianii*** X.C. Wang & W.Y. Zhuang, sp. nov.	CS13-01 = CGMCC 3.25187 T	China: Chongqing	soil	**OQ870820**	**OR051175**	**OR051352**	**OR051499**
				CS14-23	China: Chongqing	soil	**OQ870821**	**OR051176**	**OR051353**	**OR051500**
*Aspergilloides*	*Lanata-Divaricata*	*Oxalica*	*P. oxalicum* Currie & Thom 1915	CBS 219.30 T	USA	soil	AF033438	KF296462	MN969283	JN121456

GenBank accession numbers in bold indicate the newly generated sequences. The phrase ‘n.a.’ is the abbreviation of ‘not available’.

**Table 7:** Species and sequences of *Penicillium* subgen. *Aspergilloides* sect. *Sclerotiorum* used in phylogenetic analyses.

Subgenus	Section	Series	Species	Strain	Locality	Substrate	ITS	BenA	CaM	RPB2
*Aspergilloides*	*Sclerotiorum*	*Adametziorum*	*P. adametzii* K.W. Zaleski 1927	CBS 209.28 T	Poland	soil under conifers	JN714929	JN625957	KC773796	JN121455
				CS04-01	China: Chongqing	soil of ant hole	**OQ870828**	**OR051177**	n.a.	**OR062043**
				CS08-01	China: Chongqing	soil	**OQ870829**	**OR051178**	**OR051354**	**OR062044**
				CS08-05	China: Chongqing	soil	**OQ870830**	**OR051179**	n.a.	**OR062045**
*Aspergilloides*	*Sclerotiorum*	*Adametziorum*	*P. adametzioides* S. Abe ex G. Sm. 1963	CBS 313.59 T	Japan		JN686433	JN799642	JN686387	JN406578
*Aspergilloides*	*Sclerotiorum*	*Adametziorum*	*P. alexiae* Visagie et al. 2013	CBS 134558 T	Tunisia	soil of *Quercus suber* forest	KC790400	KC773778	KC773803	KX961291
*Aspergilloides*	*Sclerotiorum*	*Adametziorum*	*P. amaliae* Visagie et al. 2013	CBS 134209 T	South Africa	infructescence of *Protea repens*	JX091443	JX091563	JX141557	KX961292
*Aspergilloides*	*Sclerotiorum*	*Adametziorum*	*P. angulare* S.W. Peterson et al. 2004	CBS 130293 T	USA	old polypore on dead stump of conifer	AF125937	KC773779	KC773804	JN406554
*Aspergilloides*	*Sclerotiorum*	*Adametziorum*	*P. arianeae* Visagie et al. 2013	CBS 134559 T	The Netherlands	soil	KC773833	KC773784	KC773811	KX961294
*Aspergilloides*	*Sclerotiorum*	*Adametziorum*	*P. barbosae* S. Ramos et al. 2021	URM 7705 T	Brazil	sugarcane soil	MW191494	MG452818	MW183245	LR898886
*Aspergilloides*	*Sclerotiorum*	*Adametziorum*	*P. bilaiae* Chalab. 1950	CBS 221.66 T	former Soviet Union	soil	JN714937	JN625966	JN626009	JN406610
*Aspergilloides*	*Sclerotiorum*	*Adametziorum*	*P. brocae* S.W. Peterson et al. 2003	CBS 116113 T	Mexico	feces of *Hypothenemus hampei*	AF484398	KC773787	KC773814	JN406639
*Aspergilloides*	*Sclerotiorum*	*Adametziorum*	*P. jugoslavicum* C. Ramírez & Munt.-Cvetk. 1984	CBS 192.87 T	former Yugoslavia	seed of *Helianthus annuus*	KC773836	KC773789	KC773815	JN406618
*Aspergilloides*	*Sclerotiorum*	*Adametziorum*	*P. lilacinoechinulatum* S. Abe ex G. Sm. 1963	CBS 454.93 T	Japan		AY157489	KC773790	KC773816	KX961293
*Aspergilloides*	*Sclerotiorum*	*Adametziorum*	*P. limae* S. Ramos et al. 2021	URM 7706 T	Brazil	sugarcane soil	MW191493	MG452820	MW183244	LR898888
*Aspergilloides*	*Sclerotiorum*	*Adametziorum*	*P. mellis* R.N. Barbosa et al. 2018	CBS 142499 T	Brazil	honey produced by *Melipona scutellaris*	MN431398	MN969417	MN969327	LT854652
*Aspergilloides*	*Sclerotiorum*	*Adametziorum*	*P. reconvexovelosoi* J.P. Andrade et al. 2019	CCDCA 11500 T	Brazil	leaf litter	n.a.	MN497417	MN497418	n.a.
*Aspergilloides*	*Sclerotiorum*	*Adametziorum*	*P. restingae* J.P. Andrade et al. 2014	CBS 140379 T	Brazil	soil	KF803355	KF803349	KF803352	MN969134
*Aspergilloides*	*Sclerotiorum*	*Herqueorum*	***P. additum*** X.C. Wang & W.Y. Zhuang, sp. nov.	CS16-03 = CGMCC 3.25145 T	China: Chongqing	soil	**OQ870831**	**OR051180**	**OR051355**	**OR062046**
*Aspergilloides*	*Sclerotiorum*	*Herqueorum*	***P. brachycaulis*** X.C. Wang & W.Y. Zhuang, sp. nov.	CS24-11 = CGMCC 3.25148 T	China: Chongqing	soil under *Larix* sp.	**OQ870832**	**OR051181**	**OR051356**	**OR062047**
*Aspergilloides*	*Sclerotiorum*	*Herqueorum*	***P. celere*** X.C. Wang & W.Y. Zhuang, sp. nov.	CS28-05 = CGMCC 3.25172 T	China: Chongqing	soil	**OQ870848**	**OR051197**	**OR051372**	**OR062062**
*Aspergilloides*	*Sclerotiorum*	*Herqueorum*	*P. choerospondiatis* X.C. Wang & W.Y. Zhuang 2017	CGMCC 3.18411 T	China: Hunan	fruits of *Choerospondias axillaris*	KX885063	KX885043	KX885053	KX885034
*Aspergilloides*	*Sclerotiorum*	*Herqueorum*	***P. creberum*** X.C. Wang & W.Y. Zhuang, sp. nov.	CS02-09 = CGMCC 3.25153 T	China: Chongqing	soil	**OQ870833**	**OR051182**	**OR051357**	**OR062048**
				CS16-08	China: Chongqing	soil	**OQ870834**	**OR051183**	**OR051358**	**OR062049**
*Aspergilloides*	*Sclerotiorum*	*Herqueorum*	***P. ellipsoideum*** X.C. Wang & W.Y. Zhuang, sp. nov.	CS20-01 = CGMCC 3.25156 T	China: Chongqing	soil	**OQ870835**	**OR051184**	**OR051359**	**OR062050**
				CS20-11	China: Chongqing	soil	**OQ870836**	**OR051185**	**OR051360**	n.a.
				CS28-04	China: Chongqing	soil	**OQ870837**	**OR051186**	**OR051361**	**OR062051**
				CS29-01	China: Shaanxi	soil	**OQ870838**	**OR051187**	**OR051362**	**OR062052**
*Aspergilloides*	*Sclerotiorum*	*Herqueorum*	***P. flosculum*** X.C. Wang & W.Y. Zhuang, sp. nov.	CS33-03 = CGMCC 3.25159 T	China: Sichuan	soil	**OQ870839**	**OR051188**	**OR051363**	**OR062053**
*Aspergilloides*	*Sclerotiorum*	*Herqueorum*	*P. herquei* Bainier & Sartory 1912	CBS 336.48 T	France	leaf of *Agauria pirifolia*	JN626101	JN625970	JN626013	JN121494
*Aspergilloides*	*Sclerotiorum*	*Herqueorum*	***P. jiangjinense*** X.C. Wang & W.Y. Zhuang, sp. nov.	CS04-14 = CGMCC 3.25160 T	China: Chongqing	soil	**OQ870840**	**OR051189**	**OR051364**	**OR062054**
*Aspergilloides*	*Sclerotiorum*	*Herqueorum*	*P. malachiteum* (Yaguchi & Udagawa) Houbraken & Samson 2011	CBS 647.95 T	Japan	soil	KC773838	KC773794	KC773820	MN969125
*Aspergilloides*	*Sclerotiorum*	*Herqueorum*	*P. neoherquei* Labuda et al. 2022	CBS 148692 T	USA	mushroom sporocarp	MW341222	OL840853	OL840855	MW349119
*Aspergilloides*	*Sclerotiorum*	*Herqueorum*	*P. sanshaense* X.C. Wang & W.Y. Zhuang 2017	CGMCC 3.18413 T	China: Hainan	soil	KX885070	KX885050	KX885060	n.a.
*Aspergilloides*	*Sclerotiorum*	*Herqueorum*	***P. scruposum*** X.C. Wang & W.Y. Zhuang, sp. nov.	CS13-09 = CGMCC 3.25167 T	China: Chongqing	soil	**OQ870841**	**OR051190**	**OR051365**	**OR062055**
				CS13-19	China: Chongqing	soil	**OQ870842**	**OR051191**	**OR051366**	**OR062056**
				CS13-20	China: Chongqing	soil	**OQ870843**	**OR051192**	**OR051367**	**OR062057**
*Aspergilloides*	*Sclerotiorum*	*Herqueorum*	***P. sphaerioides*** X.C. Wang & W.Y. Zhuang, sp. nov.	CS02-11 = CGMCC 3.25175 T	China: Chongqing	soil	**OQ870850**	**OR051199**	**OR051374**	**OR062064**
				CS02-12	China: Chongqing	soil	**OQ870851**	**OR051200**	**OR051375**	**OR062065**
				CS04-03	China: Chongqing	soil	**OQ870852**	**OR051201**	**OR051376**	**OR062066**
*Aspergilloides*	*Sclerotiorum*	*Herqueorum*	***P. subasperum*** X.C. Wang & W.Y. Zhuang, sp. nov.	CS04-02 = CGMCC 3.25173 T	China: Chongqing	soil	**OQ870849**	**OR051198**	**OR051373**	**OR062063**
*Aspergilloides*	*Sclerotiorum*	*Herqueorum*	***P. subglobosum*** X.C. Wang & W.Y. Zhuang, sp. nov.	CS16-01 = CGMCC 3.25171 T	China: Chongqing	soil	**OQ870844**	**OR051193**	**OR051368**	**OR062058**
				CS16-02	China: Chongqing	soil	**OQ870845**	**OR051194**	**OR051369**	**OR062059**
				CS16-04	China: Chongqing	soil	**OQ870846**	**OR051195**	**OR051370**	**OR062060**
				CS18-26	China: Chongqing	soil	**OQ870847**	**OR051196**	**OR051371**	**OR062061**
*Aspergilloides*	*Sclerotiorum*	*Herqueorum*	***P. tardicrescens*** X.C. Wang & W.Y. Zhuang, sp. nov.	CS14-24 = CGMCC 3.25178 T	China: Chongqing	soil	**OQ870853**	**OR051202**	**OR051377**	**OR062067**
*Aspergilloides*	*Sclerotiorum*	*Herqueorum*	*P. umkhoba* Visagie & Yilmaz 2023	CBS 147457 T	South Africa	soil	MT949912	MT957417	MT957459	MT957485
*Aspergilloides*	*Sclerotiorum*	*Herqueorum*	*P. verrucisporum* X.C. Wang & W.Y. Zhuang 2017	CGMCC 3.18415 T	China: Hunan	soil	KX885069	KX885049	KX885059	KX885040
*Aspergilloides*	*Sclerotiorum*	*Herqueorum*	***P. wanyuanense*** X.C. Wang & W.Y. Zhuang, sp. nov.	CS33-06 = CGMCC 3.25182 T	China: Sichuan	soil	**OQ870854**	**OR051203**	**OR051378**	**OR062068**
				CS33-09	China: Sichuan	soil	**OQ870855**	**OR051204**	**OR051379**	**OR062069**
*Aspergilloides*	*Sclerotiorum*	*Herqueorum*	***P. wulientehii*** X.C. Wang & W.Y. Zhuang, sp. nov.	CS32-02 = CGMCC 3.25183 T	China: Sichuan	soil of ant hole	**OQ870856**	**OR051205**	**OR051380**	**OR062070**
*Aspergilloides*	*Sclerotiorum*	*Sclerotiorum*	*P. acidum* Hyang B. Lee et al. 2018	CNUFC DLW4-1 T	South Korea	plant debris in water	KY587441	KY587439	KY587442	KY587446
*Aspergilloides*	*Sclerotiorum*	*Sclerotiorum*	***P. asterineum*** X.C. Wang & W.Y. Zhuang, sp. nov.	CS05-03 = CGMCC 3.25146 T	China: Chongqing	soil	**OQ870857**	**OR051206**	**OR051381**	**OR062071**
*Aspergilloides*	*Sclerotiorum*	*Sclerotiorum*	*P. austrosinicum* X.C. Wang & W.Y. Zhuang 2017	CGMCC 3.18410 T	China: Guangdong	rotten fruit	KX885061	KX885041	KX885051	KX885032
				CS03-04	China: Chongqing	soil	**OQ870858**	**OR051207**	**OR051382**	**OR062072**
*Aspergilloides*	*Sclerotiorum*	*Sclerotiorum*	***P. beibeiense*** X.C. Wang & W.Y. Zhuang, sp. nov.	CS02-05 = CGMCC 3.25147 T	China: Chongqing	soil	**OQ870859**	**OR051208**	**OR051383**	**OR062073**
				CS02-08	China: Chongqing	soil	**OQ870860**	**OR051209**	**OR051384**	**OR062074**
*Aspergilloides*	*Sclerotiorum*	*Sclerotiorum*	*P. cainii* K.G. Rivera et al. 2011	CCFC 239914 T	Canada	nuts of *Juglans nigra*	JN686435	JN686366	JN686389	MT156346
				CS21-03	China: Chongqing	soil	**OQ870861**	**OR051210**	**OR051385**	**OR062075**
				CS33-11	China: Sichuan	soil	**OQ870862**	**OR051211**	**OR051386**	**OR062076**
*Aspergilloides*	*Sclerotiorum*	*Sclerotiorum*	*P. circulare* Hyang B. Lee et al. 2019	CNUFC GEU220-1 T	South Korea	forest soil	n.a.	MK481057	MK481061	MK481053
				CS16-06	China: Chongqing	soil	**OQ870863**	**OR051212**	**OR051387**	**OR062077**
				CS16-07	China: Chongqing	soil	**OQ870864**	**OR051213**	**OR051388**	**OR062078**
				CS18-12	China: Chongqing	soil	**OQ870865**	**OR051214**	**OR051389**	**OR062079**
				CS18-14	China: Chongqing	soil	**OQ870866**	**OR051215**	**OR051390**	**OR062080**
				CS33-08	China: Sichuan	soil	**OQ870867**	**OR051216**	**OR051391**	**OR062081**
*Aspergilloides*	*Sclerotiorum*	*Sclerotiorum*	***P. coccineum*** X.C. Wang & W.Y. Zhuang, sp. nov.	CS15-02 = CGMCC 3.25151 T	China: Chongqing	soil	**OQ870868**	**OR051217**	**OR051392**	**OR062082**
				CS18-01	China: Chongqing	soil	**OQ870869**	**OR051218**	n.a.	**OR062083**
				CS18-15	China: Chongqing	soil	**OQ870870**	**OR051219**	**OR051393**	**OR062084**
*Aspergilloides*	*Sclerotiorum*	*Sclerotiorum*	*P. daejeonium* S.H. Yu & H.K. Sang 2013	KACC 46609 T	South Korea	fruits of *Vitis* cv. *Cheongsoo*	JX436489	JX436493	JX436491	n.a.
*Aspergilloides*	*Sclerotiorum*	*Sclerotiorum*	***P. dazhouense*** X.C. Wang & W.Y. Zhuang, sp. nov.	CS33-19 = CGMCC 3.25155 T	China: Sichuan	soil	**OQ870871**	**OR051220**	**OR051394**	n.a.
*Aspergilloides*	*Sclerotiorum*	*Sclerotiorum*	*P. exsudans* X.C. Wang & W.Y. Zhuang 2017	CGMCC 3.18412 T	China: Guangdong	rotten fruit	KX885062	KX885042	KX885052	KX885033
*Aspergilloides*	*Sclerotiorum*	*Sclerotiorum*	*P. fernandesiae* R.N. Barbosa et al. 2018	CBS 142500 T	Brazil	nests of *Melipona scutellaris*	MF278314	MN969416	LT854649	LT854654
*Aspergilloides*	*Sclerotiorum*	*Sclerotiorum*	*P. ferraniaense* Houbraken & Di Piazza 2021	CBS 147595 T	Italy	compost	MW694951	MW689336	MW689338	MW689340
*Aspergilloides*	*Sclerotiorum*	*Sclerotiorum*	*P. guanacastense* K.G. Rivera et al. 2012	CCFC 239912 T	Costa Rica	caterpillar of *Eutelia*	JN626098	JN625967	JN626010	KX961295
*Aspergilloides*	*Sclerotiorum*	*Sclerotiorum*	*P. hirayamae* Udagawa 1959	CBS 229.60 T	Thailand	milled *Oryza sativa*	JN626095	JN625955	JN626003	JN121459
*Aspergilloides*	*Sclerotiorum*	*Sclerotiorum*	*P. jacksonii* K.G. Rivera et al. 2011	CCFC 239937 T	Canada	soil	JN686437	JN686368	JN686391	n.a.
*Aspergilloides*	*Sclerotiorum*	*Sclerotiorum*	*P. johnkrugii* K.G. Rivera et al. 2011	CCFC 239943 T	Malaysia	forest soil	JN686447	JN686378	JN686401	n.a.
*Aspergilloides*	*Sclerotiorum*	*Sclerotiorum*	*P. kalander* Visagie & Yilmaz 2023	CMW 56202 T	South Africa	soil	MT949914	MT957421	MT957461	MT957487
*Aspergilloides*	*Sclerotiorum*	*Sclerotiorum*	*P. mallochii* K.G. Rivera et al. 2012	CCFC 239917 T	Costa Rica	leaf of *Spondias mombin*	JN626104	JN625973	JN626016	KX961296
*Aspergilloides*	*Sclerotiorum*	*Sclerotiorum*	*P. maximae* Visagie et al. 2013	CBS 134565 T	USA	weathering treated cellophane	EU427298	KC773795	KC773821	MN969126
*Aspergilloides*	*Sclerotiorum*	*Sclerotiorum*	*P. meliponae* R.N. Barbosa et al. 2018	CBS 142495 T	Brazil	honey by *Melipona scutellaris*	MF278315	MN969418	LT854648	LT854653
*Aspergilloides*	*Sclerotiorum*	*Sclerotiorum*	*P. sclerotiorum* J.F.H. Beyma 1937	CBS 287.36 T	Indonesia	air	JN626132	JN626001	JN626044	JN406585
*Aspergilloides*	*Sclerotiorum*	*Sclerotiorum*	*P. tolerans* Y.P. Tan et al. 2022	BRIP 64090a T	Australia	soil	OK639006	OL741658	n.a.	n.a.
*Aspergilloides*	*Sclerotiorum*	*Sclerotiorum*	*P. ulleungdoense* D.H. Choi & J.G. Kim 2021	KACC 48990 T	South Korea	root of *Phedimus takesimensis*	MN640087	MN737487	MN745074	MN756007
*Aspergilloides*	*Sclerotiorum*	*Sclerotiorum*	*P. vanoranjei* Visagie et al. 2013	CBS 134406 T	Tunisia	soil of *Quercus suber* forest	KC695696	KC695686	KC695691	n.a.
*Aspergilloides*	*Sclerotiorum*	*Sclerotiorum*	*P. viticola* Nonaka & Masuma 2011	JCM 17636 T	Japan	fruit of *Vitis*	AB606414	AB540174	n.a.	n.a.
*Aspergilloides*	*Sclerotiorum*	*Sclerotiorum*	***P. wuxiense*** X.C. Wang & W.Y. Zhuang, sp. nov.	CS25-12 = CGMCC 3.25185 T	China: Chongqing	soil	**OQ870872**	**OR051221**	**OR051395**	**OR062085**
*Aspergilloides*	*Sclerotiorum*	*Sclerotiorum*	***P. xuanhanense*** X.C. Wang & W.Y. Zhuang, sp. nov.	CS31-04 = CGMCC 3.25186 T	China: Sichuan	soil	**OQ870873**	**OR051222**	**OR051396**	**OR062086**
*Aspergilloides*	*Griseola*	*Griseola*	*P. griseolum* G. Sm. 1957	CBS 277.58 T	UK	acid dunes	EF422848	EF506213	EF506232	JN121480

GenBank accession numbers in bold indicate the newly generated sequences. The phrase ‘n.a.’ is the abbreviation of ‘not available’.

**Table 8:** Detailed characteristics of the datasets.

Dataset	No. of Seq.	Length of Alignment (bp)	Model for BI
subgen. *Penicillium*	53	1867	TIMef + I + G
sect. *Aspergilloides*	50	1839	SYM + I + G
sect. *Citrina*	51	1983	GTR + I + G
sect. *Exilicaulis*	34	1919	TrNef + I + G
sect. *Gracilenta*	14	2073	TrN + I + G
sect. *Lanata-Divaricata*	161	1960	TVM + I + G
sect. *Sclerotiorum*	90	2123	GTR + I + G

## Data Availability

Data are contained within the article.

## Supplementary Materials

The following supporting information can be downloaded at: https://www.mdpi.com/article/10.3390/jof9121150/s1, Figure S1: Maximum likelihood phylogeny of *Penicillium* subgen. *Penicillium* inferred from BenA dataset. Bootstrap values ≥ 70% are indicated at nodes. Asterisk denotes 100% bootstrap. Figure S2: Maximum likelihood phylogeny of *Penicillium* subgen. *Penicillium* inferred from CaM dataset. Bootstrap values ≥ 70% are indicated at nodes. Asterisk denotes 100% bootstrap. Figure S3: Maximum likelihood phylogeny of *Penicillium* subgen. *Penicillium* inferred from RPB2 dataset. Bootstrap values ≥ 70% are indicated at nodes. Asterisk denotes 100% bootstrap. Figure S4: Maximum likelihood phylogeny of *Penicillium* subgen. *Aspergilloides* sect. *Aspergilloides* inferred from BenA dataset. Bootstrap values ≥ 70% are indicated at nodes. Asterisk denotes 100% bootstrap. Figure S5: Maximum likelihood phylogeny of *Penicillium* subgen. *Aspergilloides* sect. *Aspergilloides* inferred from CaM dataset. Bootstrap values ≥ 70% are indicated at nodes. Asterisk denotes 100% bootstrap. Figure S6: Maximum likelihood phylogeny of *Penicillium* subgen. *Aspergilloides* sect. *Aspergilloides* inferred from RPB2 dataset. Bootstrap values ≥ 70% are indicated at nodes. Asterisk denotes 100% bootstrap. Figure S7: Maximum likelihood phylogeny of *Penicillium* subgen. *Aspergilloides* sect. *Citrina* inferred from BenA dataset. Bootstrap values ≥ 70% are indicated at nodes. Asterisk denotes 100% bootstrap. Figure S8: Maximum likelihood phylogeny of *Penicillium* subgen. *Aspergilloides* sect. *Citrina* inferred from CaM dataset. Bootstrap values ≥ 70% are indicated at nodes. Asterisk denotes 100% bootstrap. Figure S9: Maximum likelihood phylogeny of *Penicillium* subgen. *Aspergilloides* sect. *Citrina* inferred from RPB2 dataset. Bootstrap values ≥ 70% are indicated at nodes. Asterisk denotes 100% bootstrap. Figure S10: Maximum likelihood phylogeny of *Penicillium* subgen. *Aspergilloides* sect. *Exilicaulis* inferred from BenA dataset. Bootstrap values ≥ 70% are indicated at nodes. Asterisk denotes 100% bootstrap. Figure S11: Maximum likelihood phylogeny of *Penicillium* subgen. *Aspergilloides* sect. *Exilicaulis* inferred from CaM dataset. Bootstrap values ≥ 70% are indicated at nodes. Asterisk denotes 100% bootstrap. Figure S12: Maximum likelihood phylogeny of *Penicillium* subgen. *Aspergilloides* sect. *Exilicaulis* inferred from RPB2 dataset. Bootstrap values ≥ 70% are indicated at nodes. Asterisk denotes 100% bootstrap. Figure S13: Maximum likelihood phylogeny of *Penicillium* subgen. *Aspergilloides* sect. *Gracilenta* inferred from BenA dataset. Bootstrap values ≥ 70% are indicated at nodes. Asterisk denotes 100% bootstrap. Figure S14: Maximum likelihood phylogeny of *Penicillium* subgen. *Aspergilloides* sect. *Gracilenta* inferred from CaM dataset. Bootstrap values ≥ 70% are indicated at nodes. Asterisk denotes 100% bootstrap. Figure S15: Maximum likelihood phylogeny of *Penicillium* subgen. *Aspergilloides* sect. *Gracilenta* inferred from RPB2 dataset. Bootstrap values ≥ 70% are indicated at nodes. Asterisk denotes 100% bootstrap. Figure S16: Maximum likelihood phylogeny of *Penicillium* subgen. *Aspergilloides* sect. *Lanata-Divaricata* inferred from BenA dataset. Bootstrap values ≥ 70% are indicated at nodes. Asterisk denotes 100% bootstrap. Figure S17: Maximum likelihood phylogeny of *Penicillium* subgen. *Aspergilloides* sect. *Lanata-Divaricata* inferred from CaM dataset. Bootstrap values ≥ 70% are indicated at nodes. Asterisk denotes 100% bootstrap. Figure S18: Maximum likelihood phylogeny of *Penicillium* subgen. *Aspergilloides* sect. *Lanata-Divaricata* inferred from RPB2 dataset. Bootstrap values ≥ 70% are indicated at nodes. Asterisk denotes 100% bootstrap. Figure S19: Maximum likelihood phylogeny of *Penicillium* subgen. *Aspergilloides* sect. *Sclerotiorum* inferred from BenA dataset. Bootstrap values ≥ 70% are indicated at nodes. Asterisk denotes 100% bootstrap. Figure S20: Maximum likelihood phylogeny of *Penicillium* subgen. *Aspergilloides* sect. *Sclerotiorum* inferred from CaM dataset. Bootstrap values ≥ 70% are indicated at nodes. Asterisk denotes 100% bootstrap. Figure S21: Maximum likelihood phylogeny of *Penicillium* subgen. *Aspergilloides* sect. *Sclerotiorum* inferred from RPB2 dataset. Bootstrap values ≥ 70% are indicated at nodes. Asterisk denotes 100% bootstrap.
