# Supplementary material for: Species Diversity of Penicillium in Southwest China with Discovery of Forty-Three New Species
Source: J Fungi (Basel). 2023 Nov 28;9(12):1150. doi: 10.3390/jof9121150 (PMC10744262; doi:10.3390/jof9121150)
Supplement: Supplementary file 1 [file jof-09-01150-s001.zip › Supplementary figure legends.pdf]

Supplementary figure legends

Figure S1. Maximum likelihood phylogeny of *Penicillium* subgen. *Penicillium* inferred from BenA dataset. Bootstrap values ≥ 70% are indicated at nodes. Asterisk denotes 100% bootstrap.

Figure S2. Maximum likelihood phylogeny of *Penicillium* subgen. *Penicillium* inferred from CaM dataset. Bootstrap values ≥ 70% are indicated at nodes. Asterisk denotes 100% bootstrap.

Figure S3. Maximum likelihood phylogeny of *Penicillium* subgen. *Penicillium* inferred from RPB2 dataset. Bootstrap values ≥ 70% are indicated at nodes. Asterisk denotes 100% bootstrap.

Figure S4. Maximum likelihood phylogeny of *Penicillium* subgen. *Aspergilloides* sect. *Aspergilloides* inferred from BenA dataset. Bootstrap values ≥ 70% are indicated at nodes. Asterisk denotes 100% bootstrap.

Figure S5. Maximum likelihood phylogeny of *Penicillium* subgen. *Aspergilloides* sect. *Aspergilloides* inferred from CaM dataset. Bootstrap values ≥ 70% are indicated at nodes. Asterisk denotes 100% bootstrap.

Figure S6. Maximum likelihood phylogeny of *Penicillium* subgen. *Aspergilloides* sect. *Aspergilloides* inferred from RPB2 dataset. Bootstrap values ≥ 70% are indicated at nodes. Asterisk denotes 100% bootstrap.

Figure S7. Maximum likelihood phylogeny of *Penicillium* subgen. *Aspergilloides* sect. *Citrina* inferred from BenA dataset. Bootstrap values ≥ 70% are indicated at nodes. Asterisk denotes 100% bootstrap.

Figure S8. Maximum likelihood phylogeny of *Penicillium* subgen. *Aspergilloides* sect. *Citrina* inferred from CaM dataset. Bootstrap values ≥ 70% are indicated at nodes. Asterisk denotes 100% bootstrap.

Figure S9. Maximum likelihood phylogeny of *Penicillium* subgen. *Aspergilloides* sect. *Citrina* inferred from RPB2 dataset. Bootstrap values ≥ 70% are indicated at nodes. Asterisk denotes 100% bootstrap.

Figure S10. Maximum likelihood phylogeny of *Penicillium* subgen. *Aspergilloides* sect. *Exilicaulis* inferred from BenA dataset. Bootstrap values ≥ 70% are indicated at nodes. Asterisk denotes 100% bootstrap.

Figure S11. Maximum likelihood phylogeny of *Penicillium* subgen. *Aspergilloides* sect. *Exilicaulis* inferred from CaM dataset. Bootstrap values ≥ 70% are indicated at nodes. Asterisk denotes 100% bootstrap.

Figure S12. Maximum likelihood phylogeny of *Penicillium* subgen. *Aspergilloides* sect. *Exilicaulis* inferred from RPB2 dataset. Bootstrap values ≥ 70% are indicated at nodes. Asterisk denotes 100% bootstrap.

Figure S13. Maximum likelihood phylogeny of *Penicillium* subgen. *Aspergilloides* sect. *Gracilenta* inferred from BenA dataset. Bootstrap values ≥ 70% are indicated at nodes. Asterisk denotes 100% bootstrap.

Figure S14. Maximum likelihood phylogeny of *Penicillium* subgen. *Aspergilloides* sect. *Gracilenta* inferred from CaM dataset. Bootstrap values ≥ 70% are indicated at nodes. Asterisk denotes 100% bootstrap.

Figure S15. Maximum likelihood phylogeny of *Penicillium* subgen. *Aspergilloides* sect. *Gracilenta* inferred from RPB2 dataset. Bootstrap values ≥ 70% are indicated at nodes. Asterisk denotes 100% bootstrap.

Figure S16. Maximum likelihood phylogeny of *Penicillium* subgen. *Aspergilloides* sect. *Lanata-Divaricata* inferred from BenA dataset. Bootstrap values ≥ 70% are indicated at nodes. Asterisk denotes 100% bootstrap.

Figure S17. Maximum likelihood phylogeny of *Penicillium* subgen. *Aspergilloides* sect. *Lanata-Divaricata* inferred from CaM dataset. Bootstrap values ≥ 70% are indicated at nodes. Asterisk denotes 100% bootstrap.

Figure S18. Maximum likelihood phylogeny of *Penicillium* subgen. *Aspergilloides* sect. *Lanata-Divaricata* inferred from RPB2 dataset. Bootstrap values ≥ 70% are indicated at nodes. Asterisk denotes 100% bootstrap.

Figure 19. Maximum likelihood phylogeny of *Penicillium* subgen. *Aspergilloides* sect. *Sclerotiorum* inferred from BenA dataset. Bootstrap values ≥ 70% are indicated at nodes. Asterisk denotes 100% bootstrap.

Figure 20. Maximum likelihood phylogeny of *Penicillium* subgen. *Aspergilloides* sect. *Sclerotiorum* inferred from CaM dataset. Bootstrap values ≥ 70% are indicated at nodes. Asterisk denotes 100% bootstrap.

Figure 21. Maximum likelihood phylogeny of *Penicillium* subgen. *Aspergilloides* sect. *Sclerotiorum* inferred from RPB2 dataset. Bootstrap values ≥ 70% are indicated at nodes. Asterisk denotes 100% bootstrap.
