# Supplementary figures and images for: Species Diversity of Penicillium in Southwest China with Discovery of Forty-Three New Species
Source: J Fungi (Basel). 2023 Nov 28;9(12):1150. doi: 10.3390/jof9121150 (PMC10744262; doi:10.3390/jof9121150)

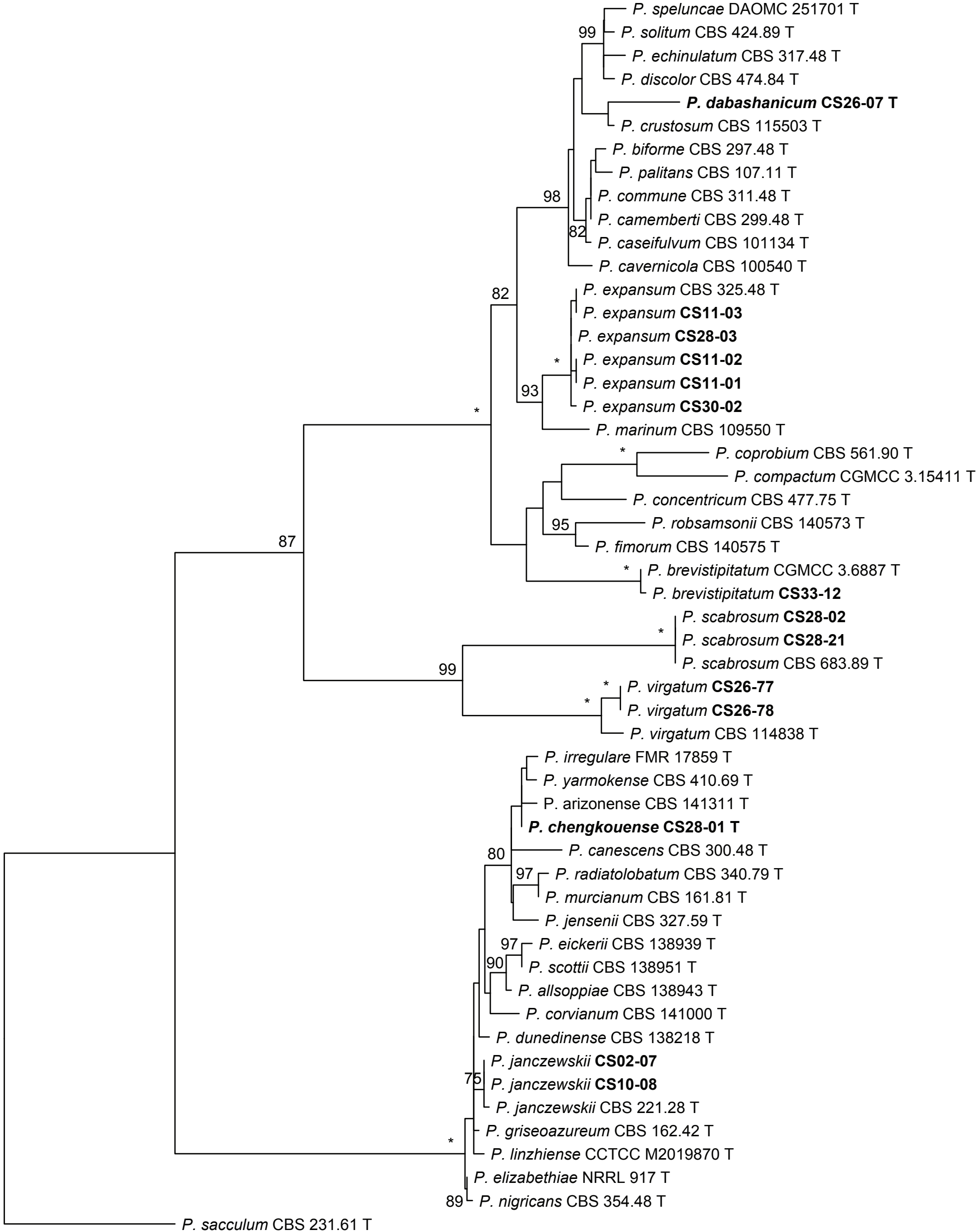

Supplement: Supplementary file 1 [file jof-09-01150-s001.zip › Figure S1 Penicillium BenA.pdf]

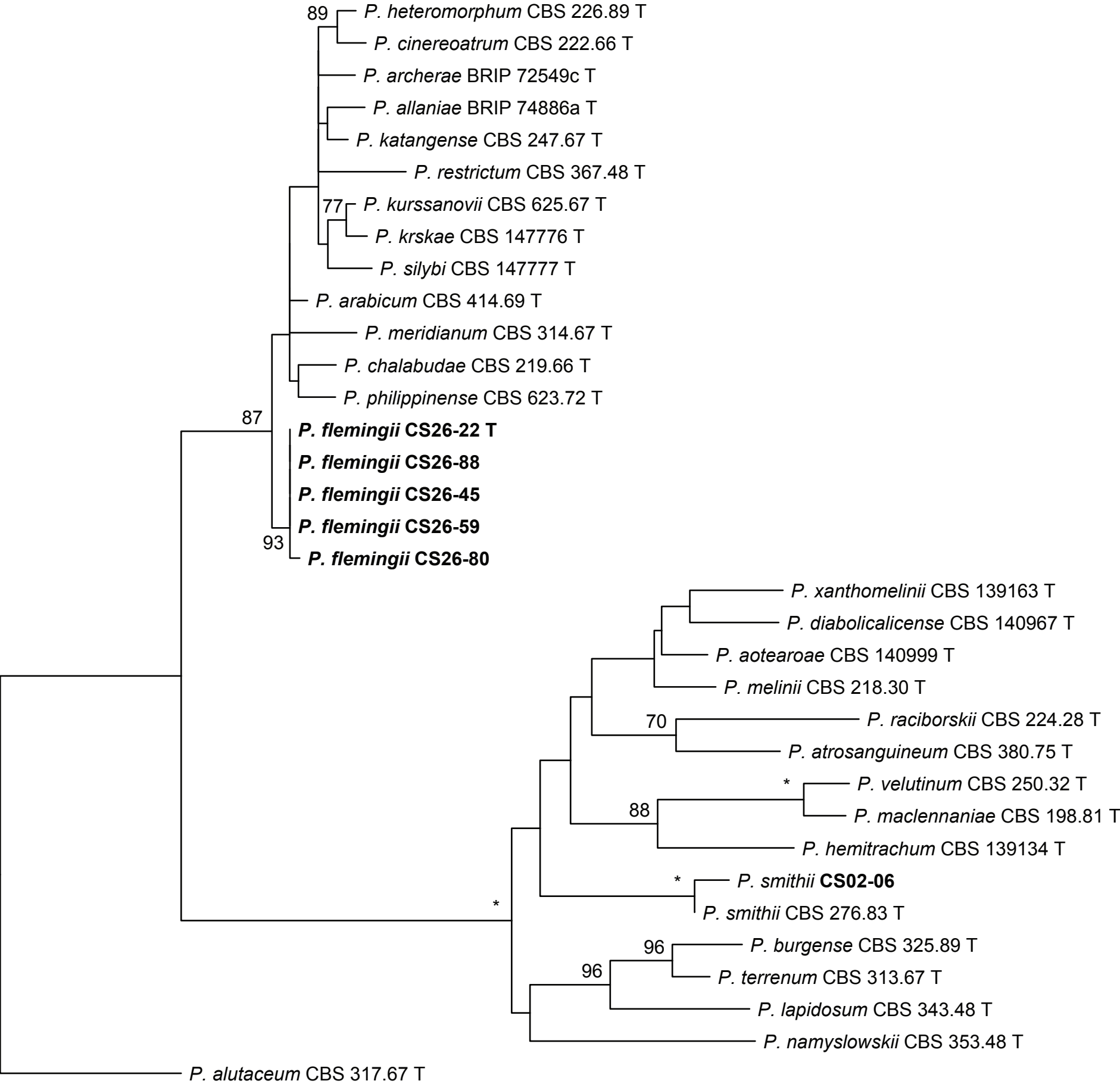

Supplement: Supplementary file 1 [file jof-09-01150-s001.zip › Figure S10 Exilicaulis BenA.pdf]

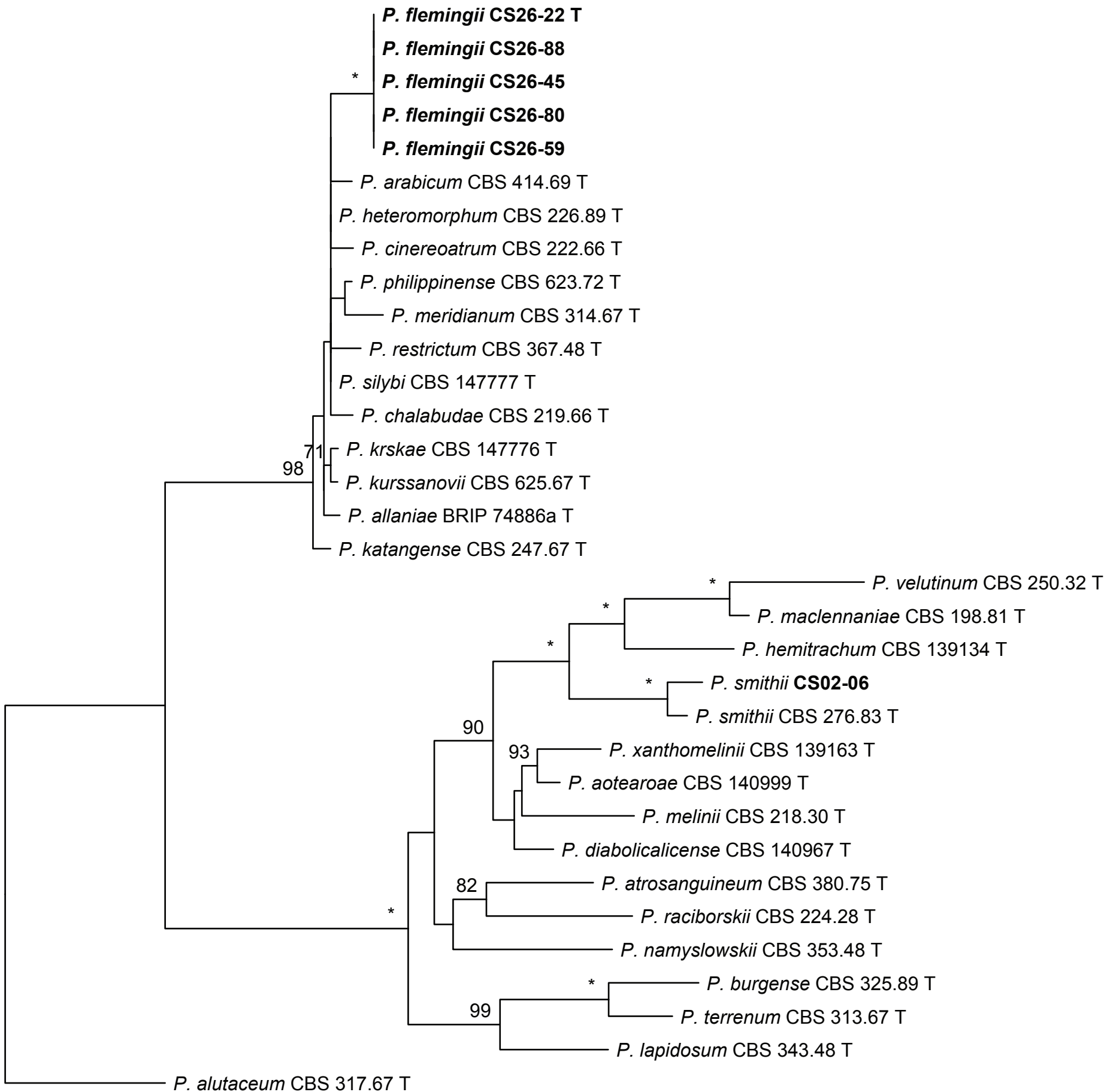

0.04

Supplement: Supplementary file 1 [file jof-09-01150-s001.zip › Figure S11 Exilicaulis CaM.pdf]

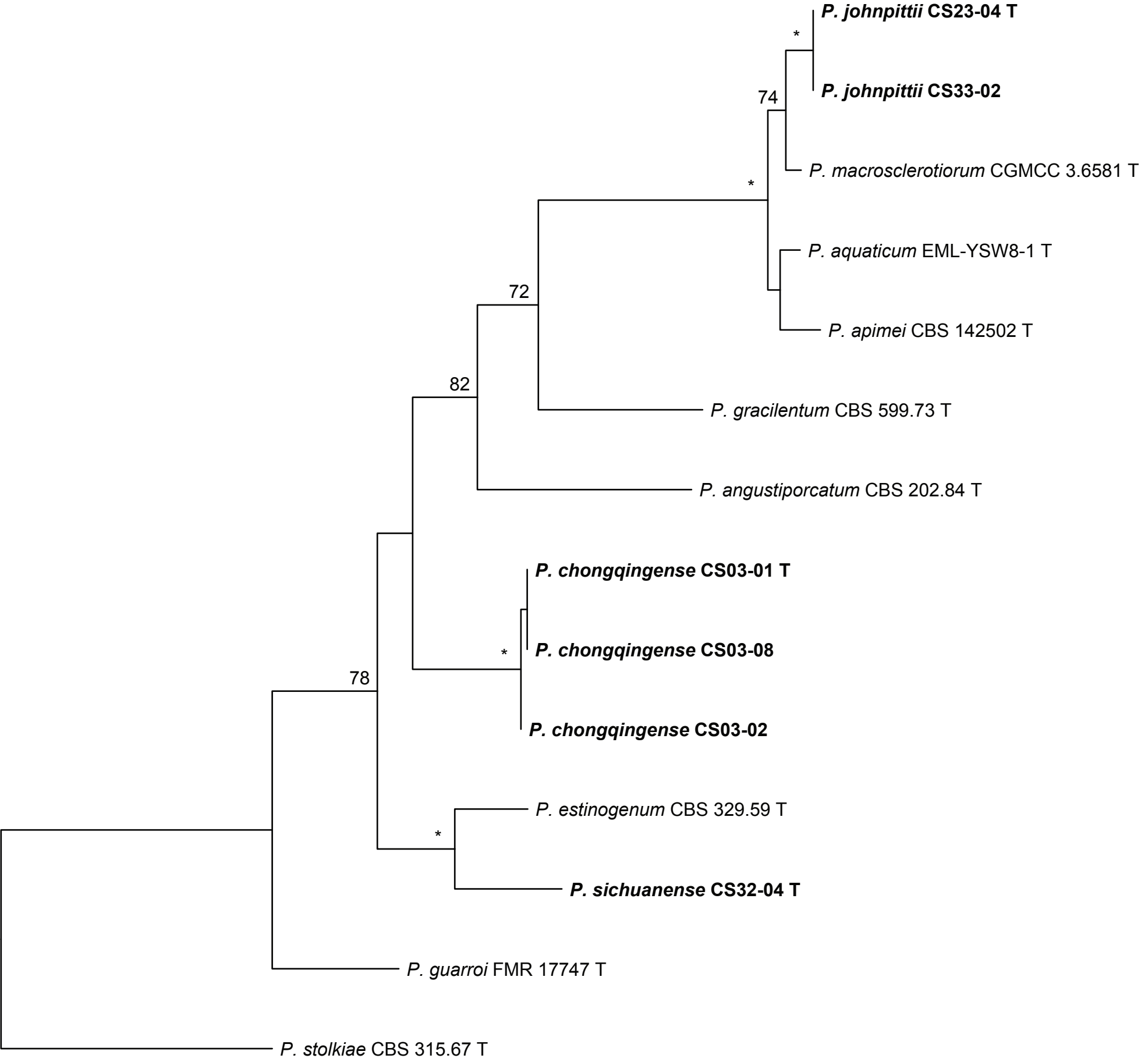

0.09

Supplement: Supplementary file 1 [file jof-09-01150-s001.zip › Figure S13 Gracilenta BenA.pdf]

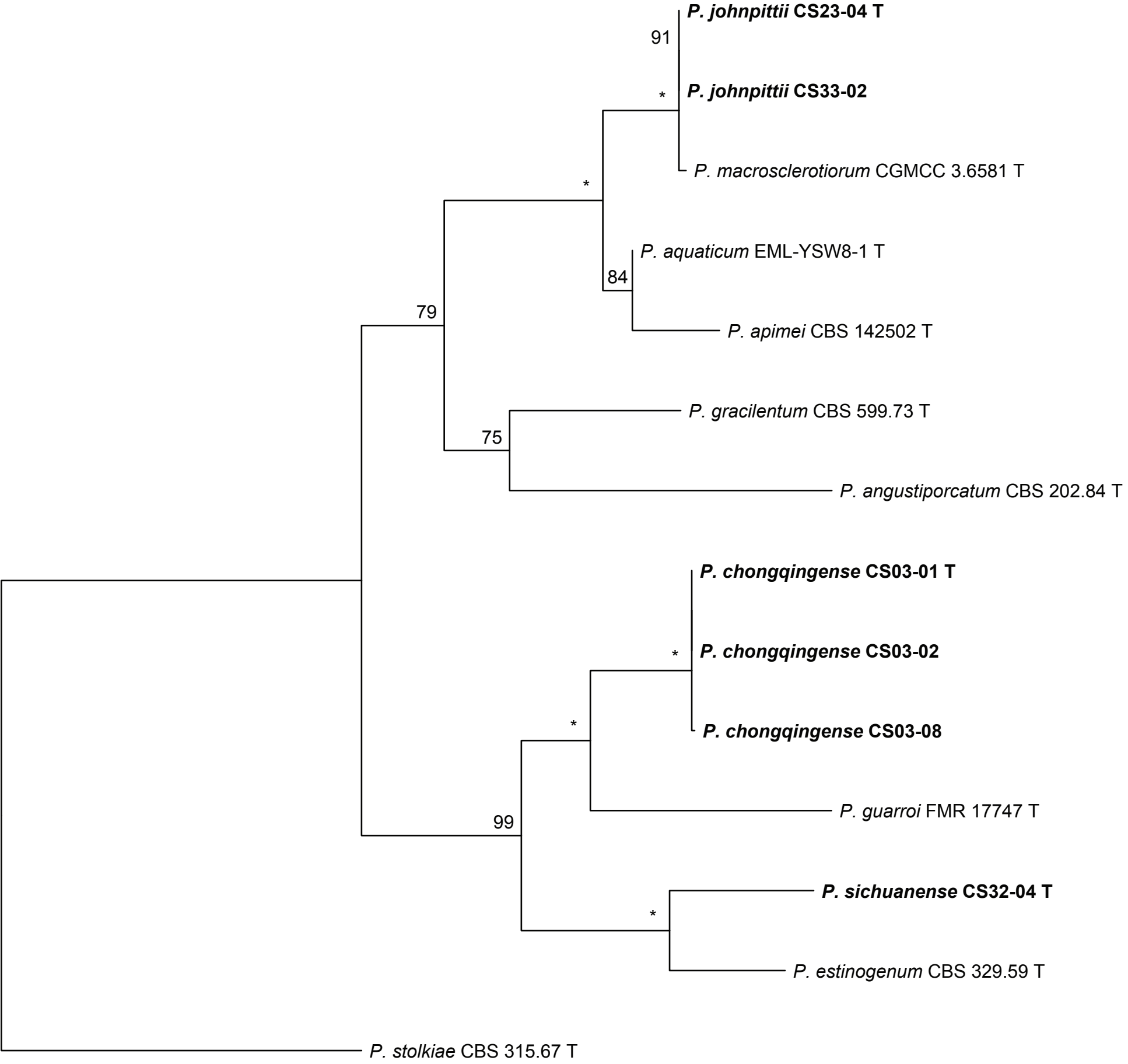

Supplement: Supplementary file 1 [file jof-09-01150-s001.zip › Figure S14 Gracilenta CaM.pdf]

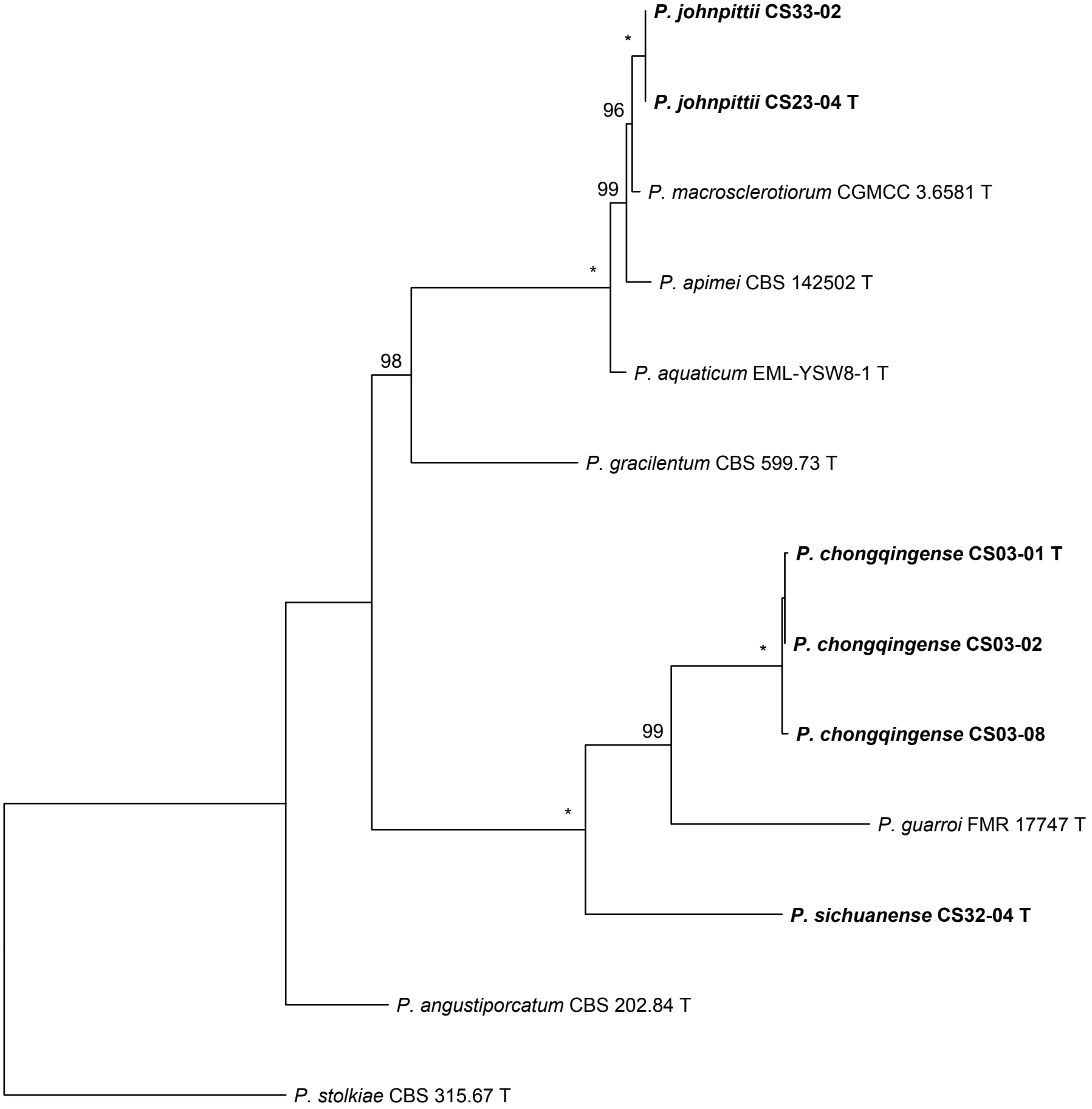

0.06

Supplement: Supplementary file 1 [file jof-09-01150-s001.zip › Figure S15 Gracilenta RPB2.pdf]

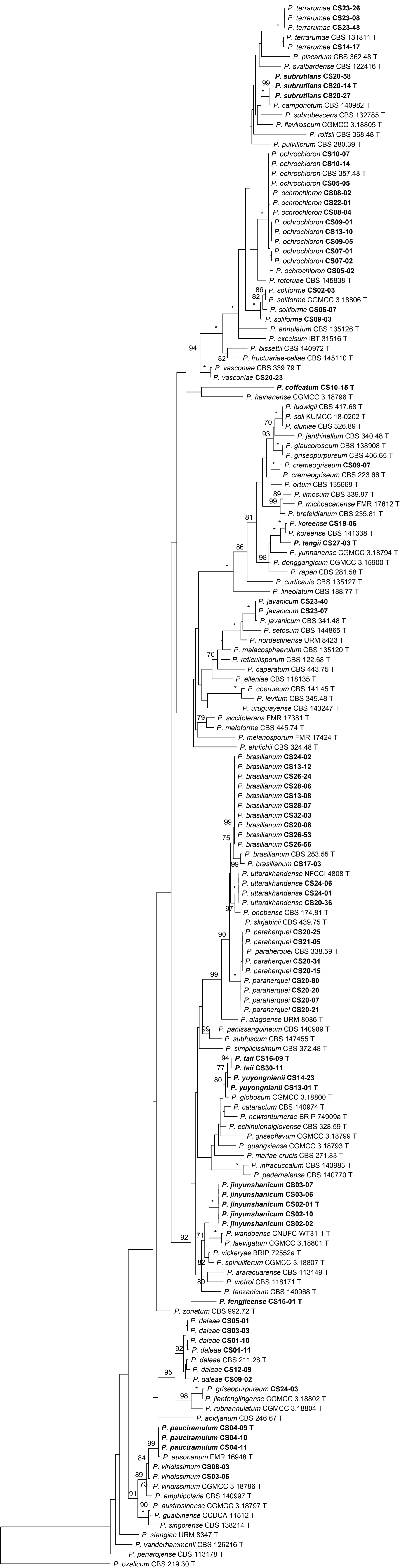

Supplement: Supplementary file 1 [file jof-09-01150-s001.zip › Figure S16 Lanata-Divaricata BenA.pdf]

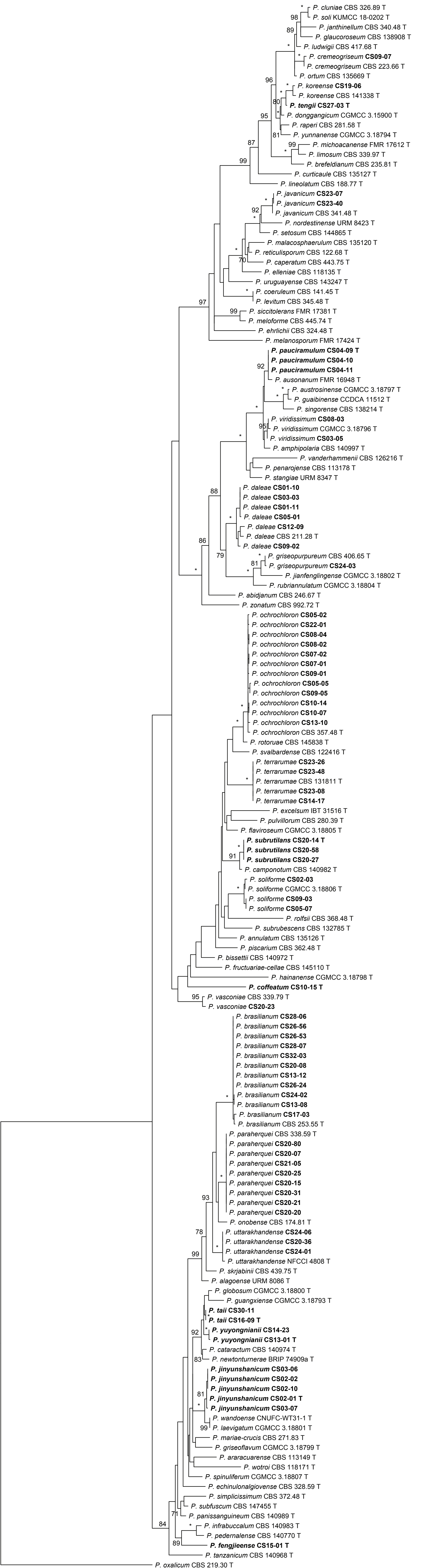

Supplement: Supplementary file 1 [file jof-09-01150-s001.zip › Figure S17 Lanata-Divaricata CaM.pdf]

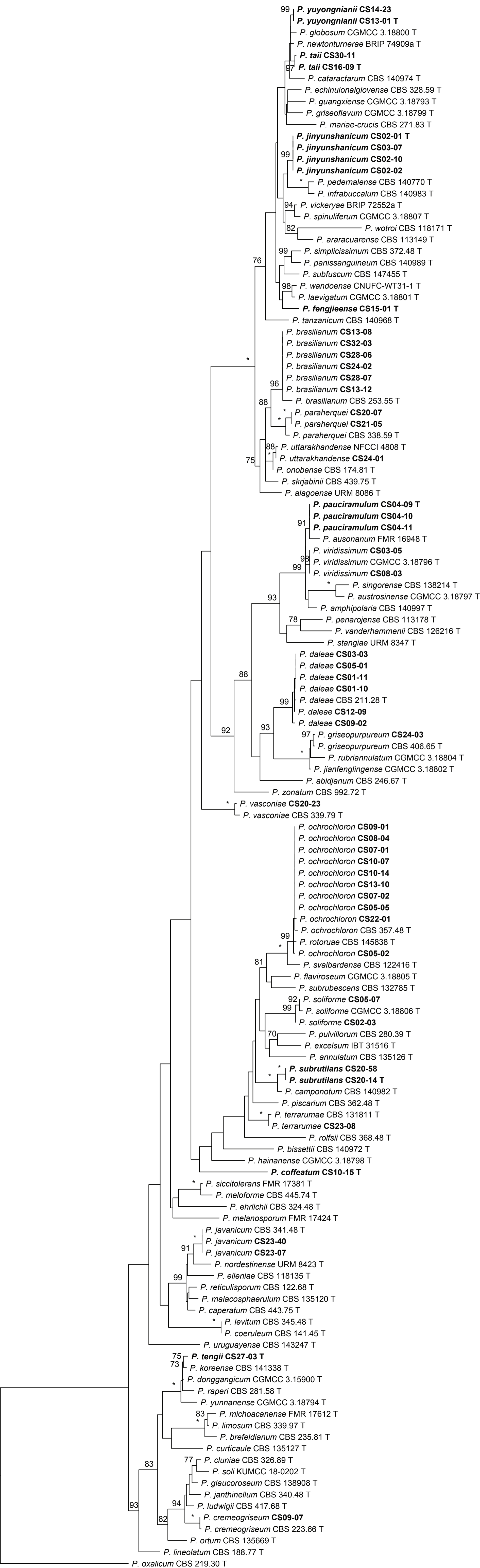

Supplement: Supplementary file 1 [file jof-09-01150-s001.zip › Figure S18 Lanata-Divaricata RPB2.pdf]

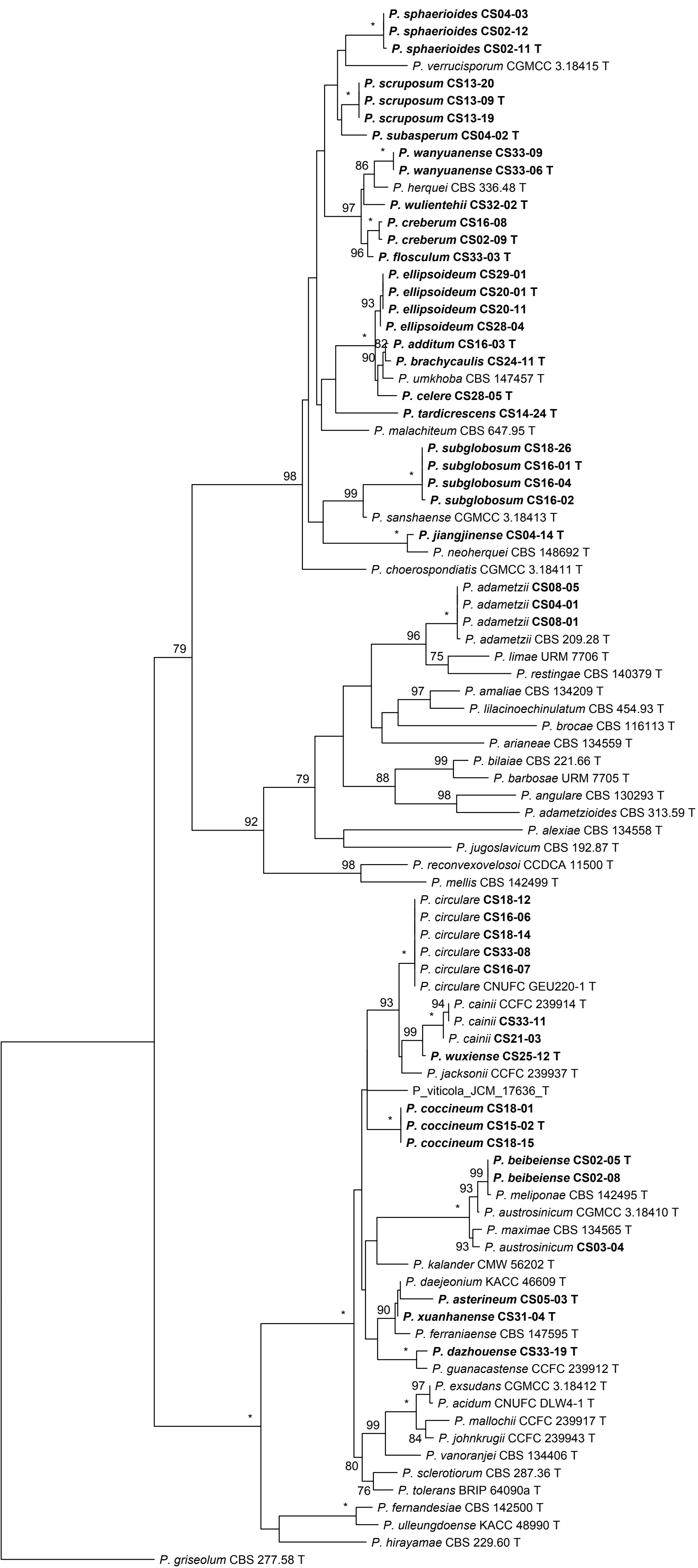

Supplement: Supplementary file 1 [file jof-09-01150-s001.zip › Figure S19 Sclerotiorum BenA.pdf]

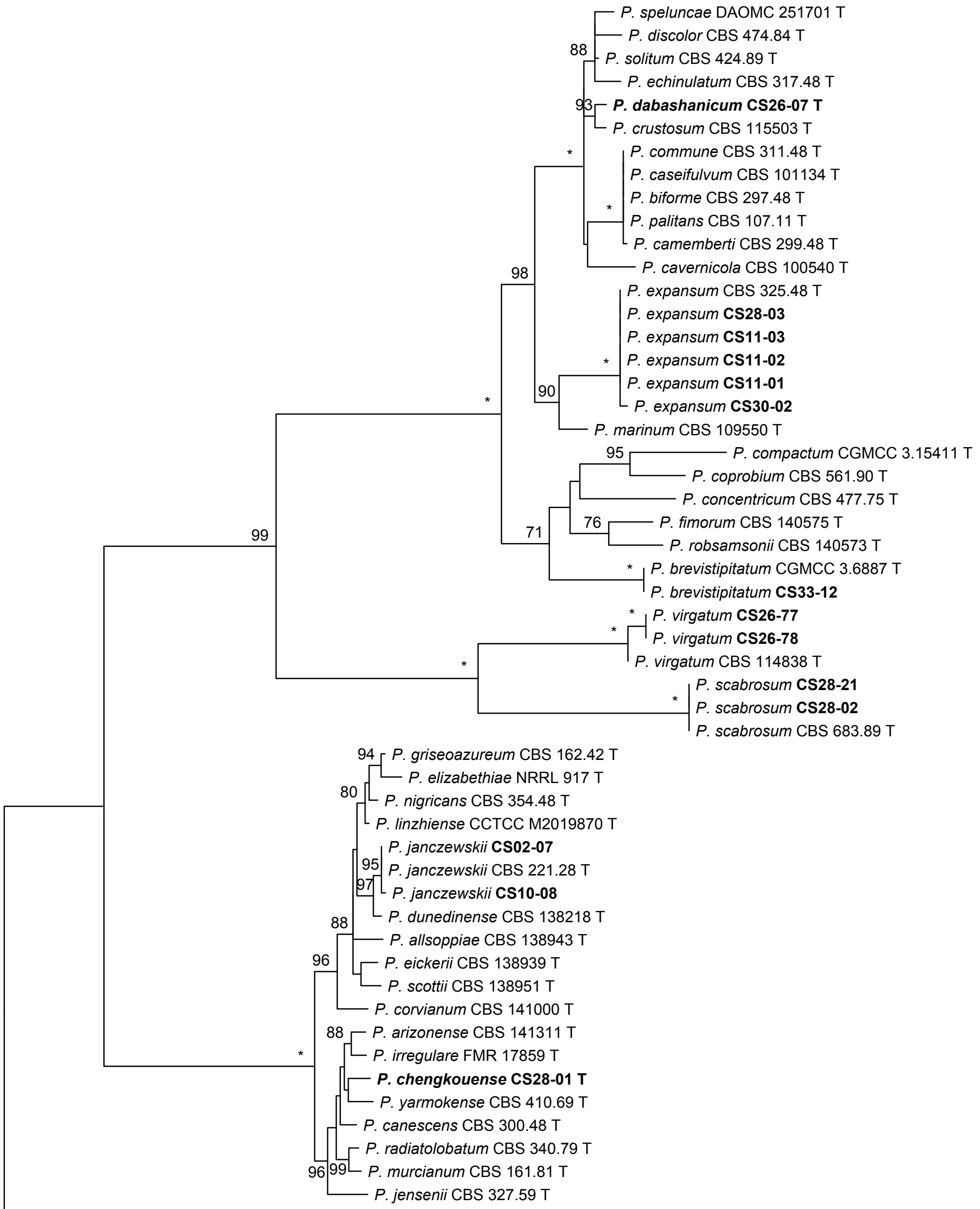

Supplement: Supplementary file 1 [file jof-09-01150-s001.zip › Figure S2 Penicillium CaM.pdf]

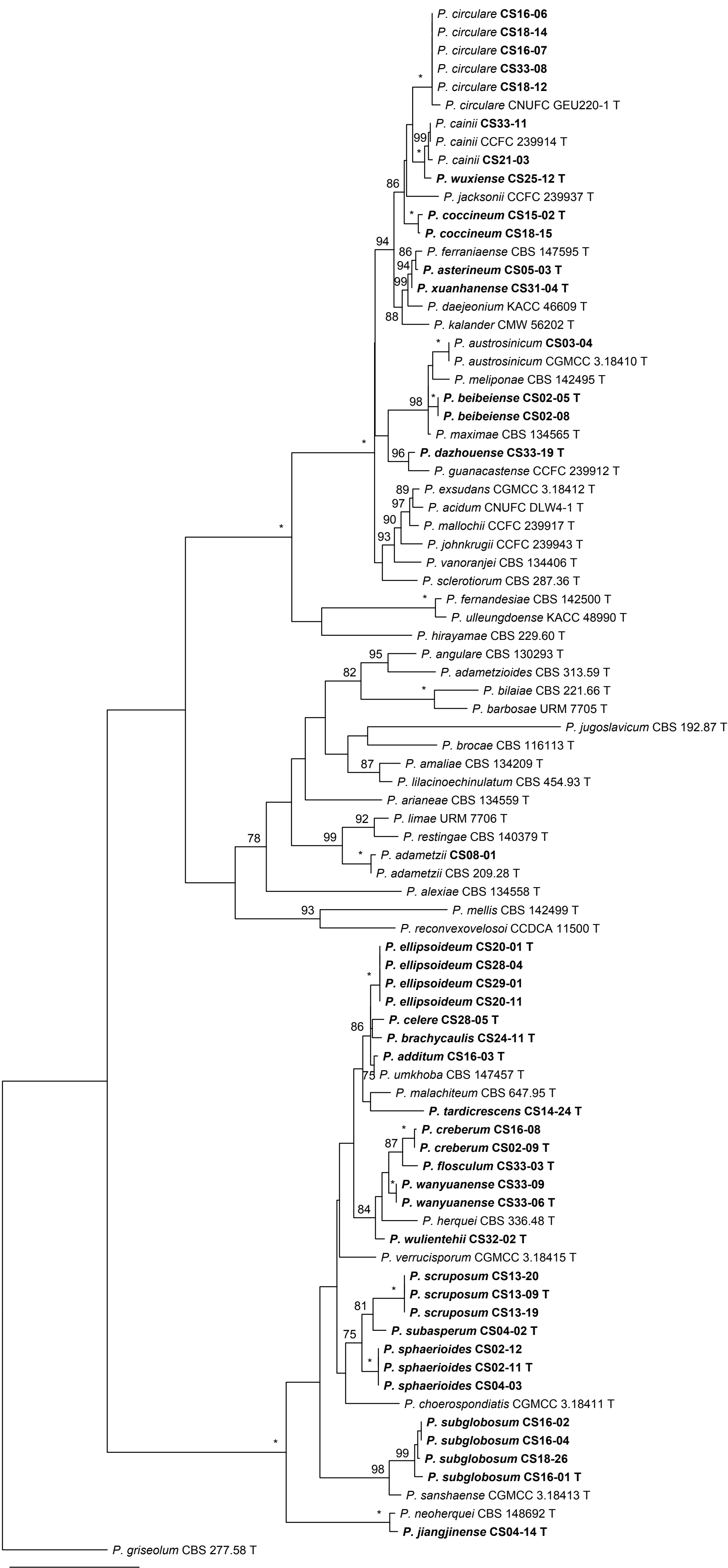

Supplement: Supplementary file 1 [file jof-09-01150-s001.zip › Figure S20 Sclerotiorum CaM.pdf]

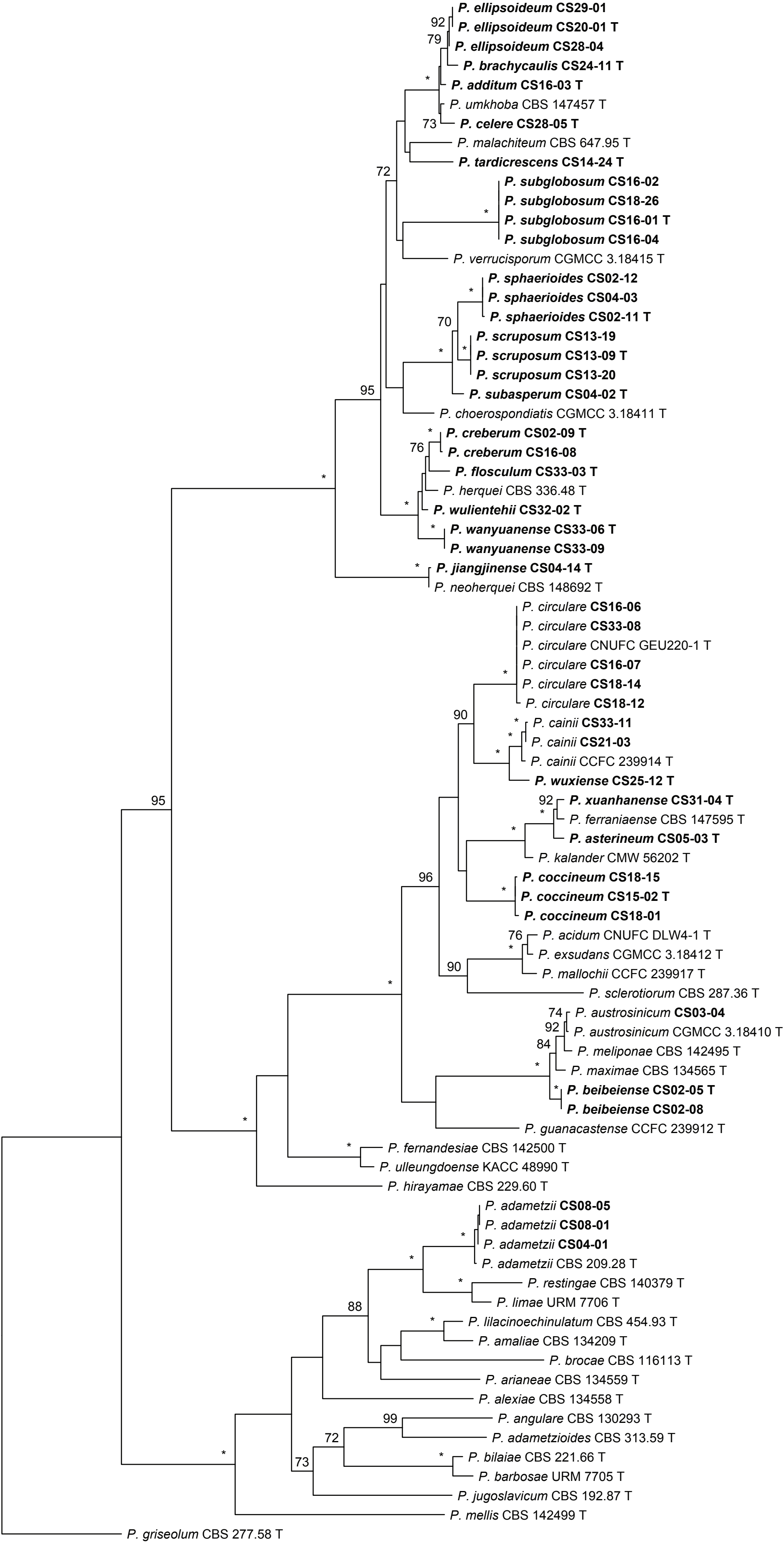

Supplement: Supplementary file 1 [file jof-09-01150-s001.zip › Figure S21 Sclerotiorum RPB2.pdf]

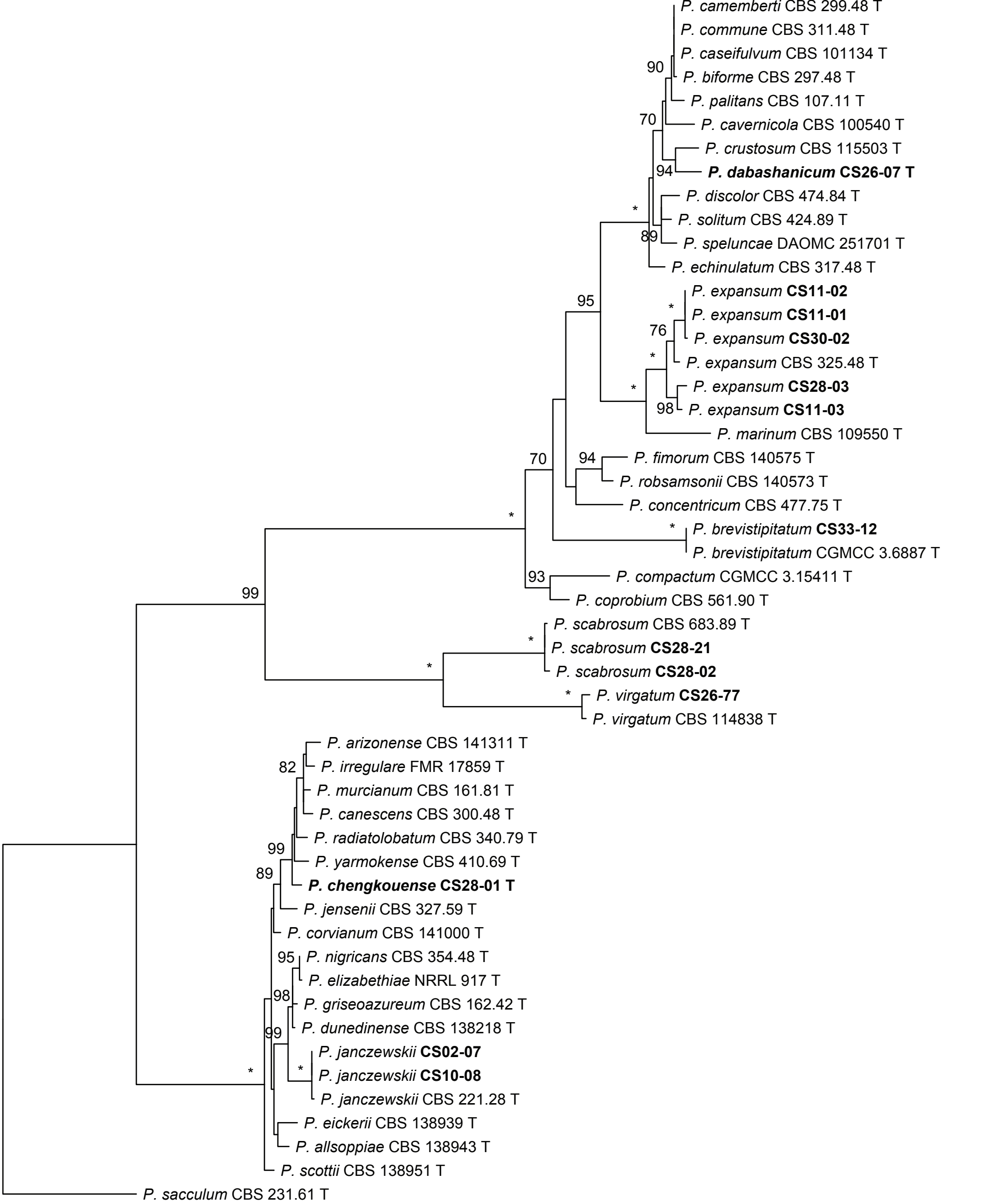

Supplement: Supplementary file 1 [file jof-09-01150-s001.zip › Figure S3 Penicillium RPB2.pdf]

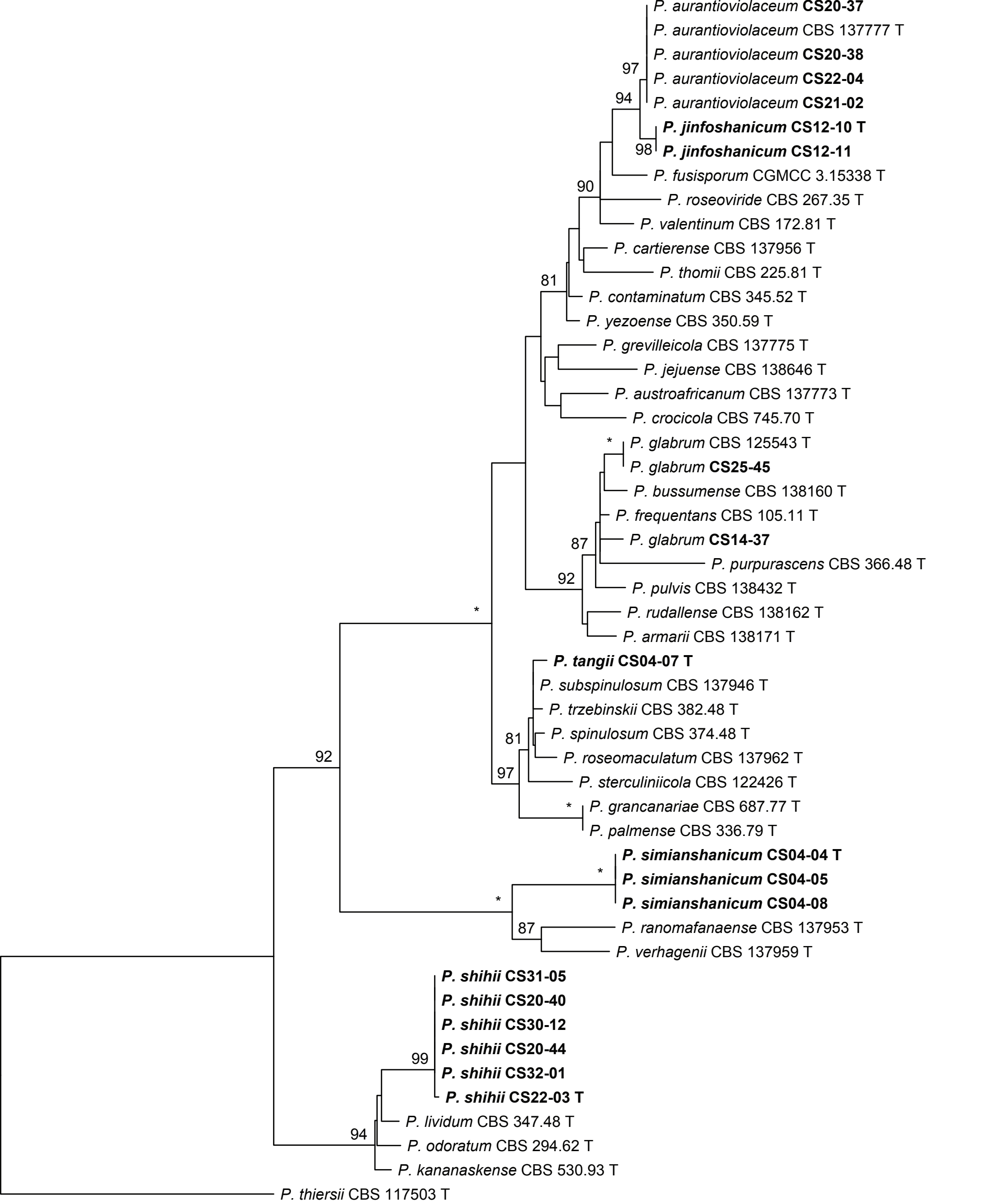

Supplement: Supplementary file 1 [file jof-09-01150-s001.zip › Figure S4 Aspergilloides BenA.pdf]

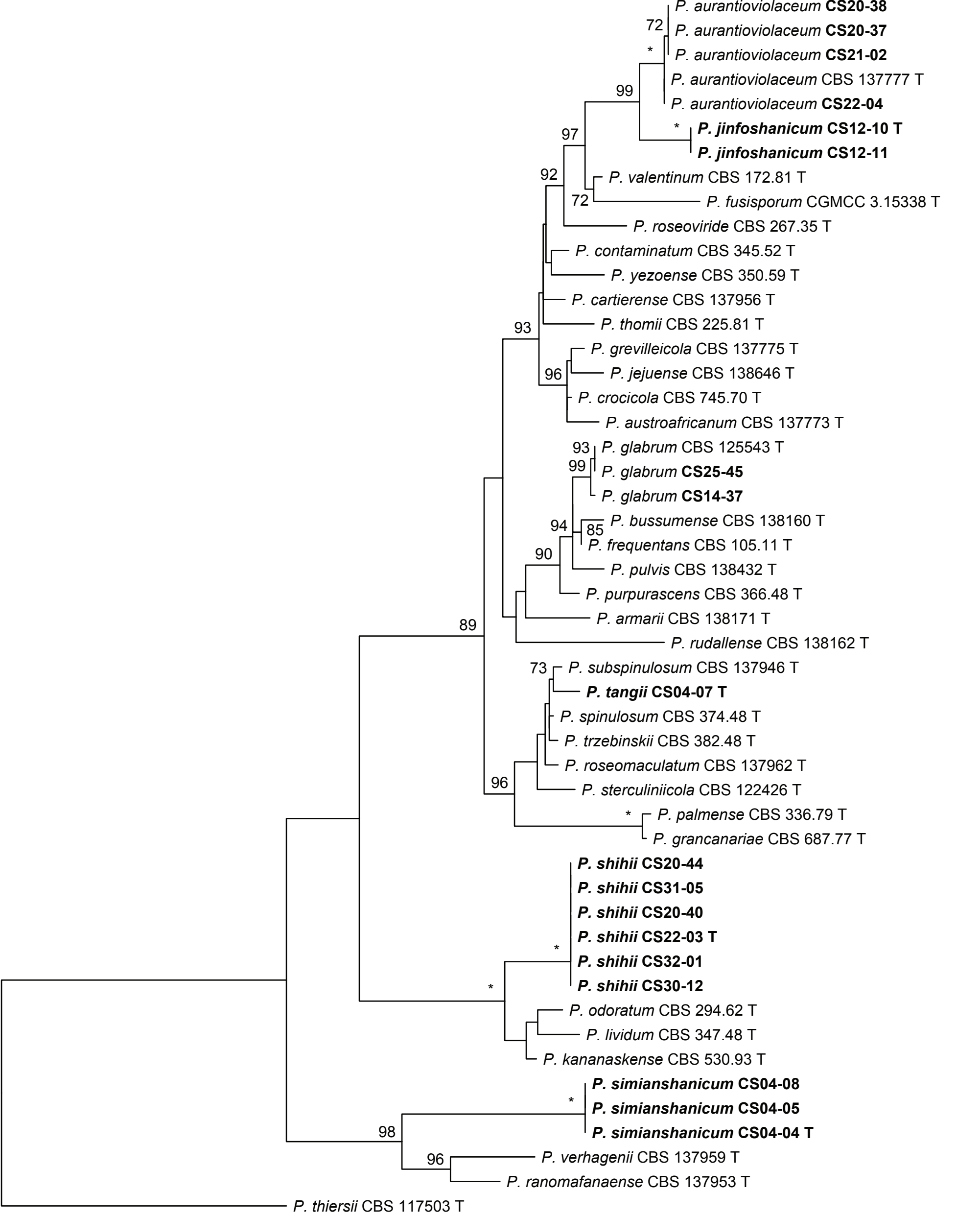

Supplement: Supplementary file 1 [file jof-09-01150-s001.zip › Figure S5 Aspergilloides CaM.pdf]

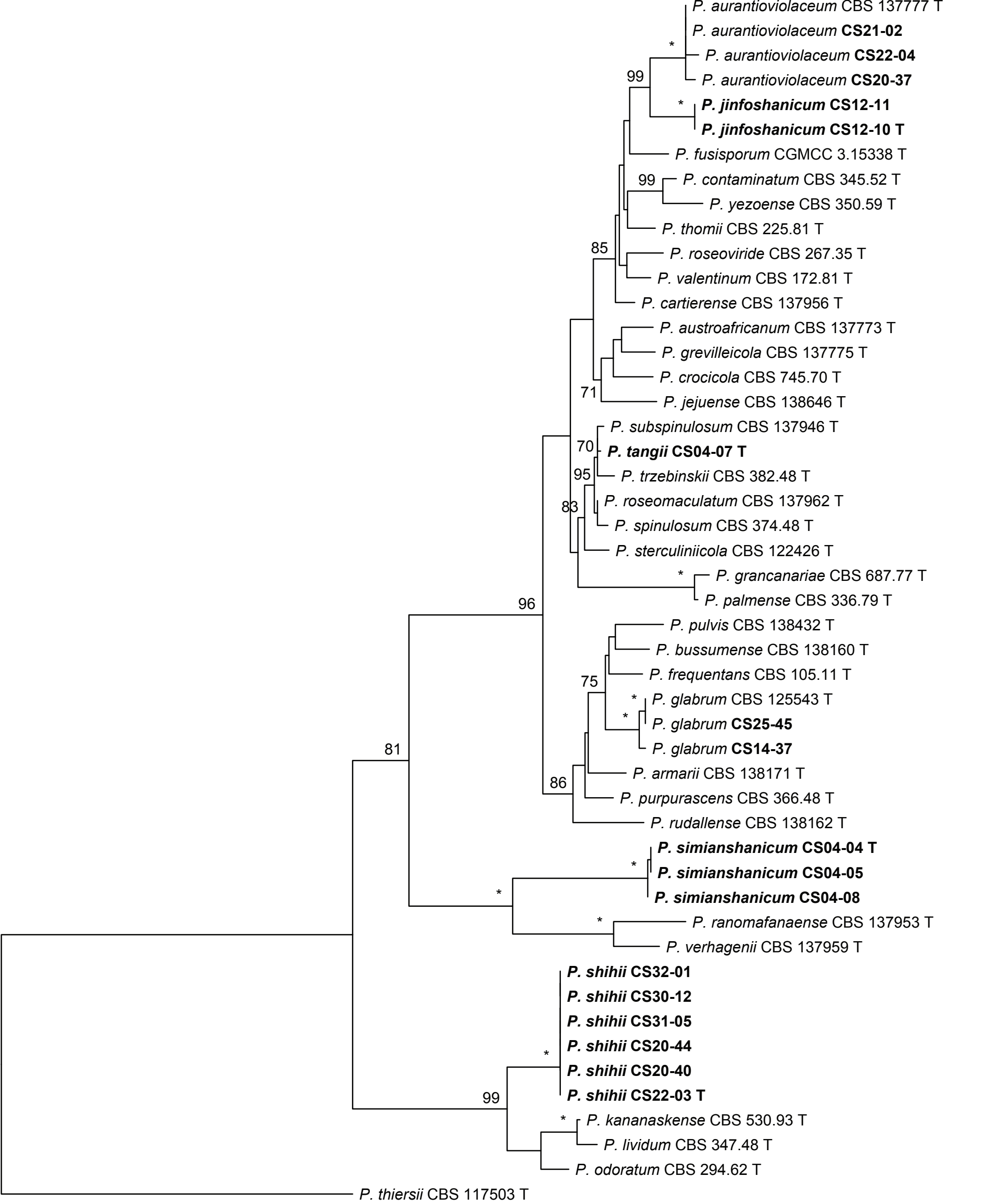

Supplement: Supplementary file 1 [file jof-09-01150-s001.zip › Figure S6 Aspergilloides RPB2.pdf]

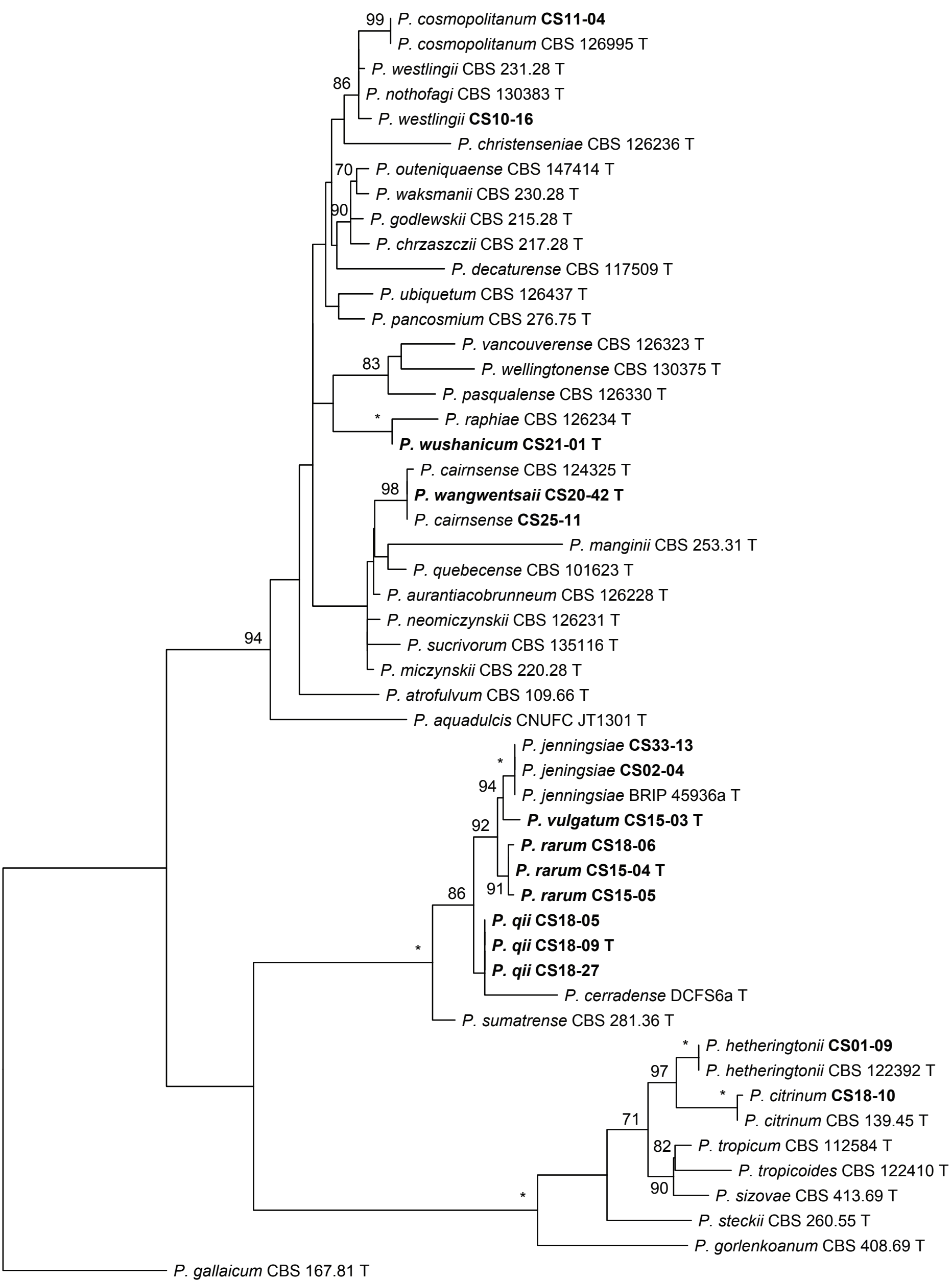

Supplement: Supplementary file 1 [file jof-09-01150-s001.zip › Figure S7 Citrina BenA.pdf]

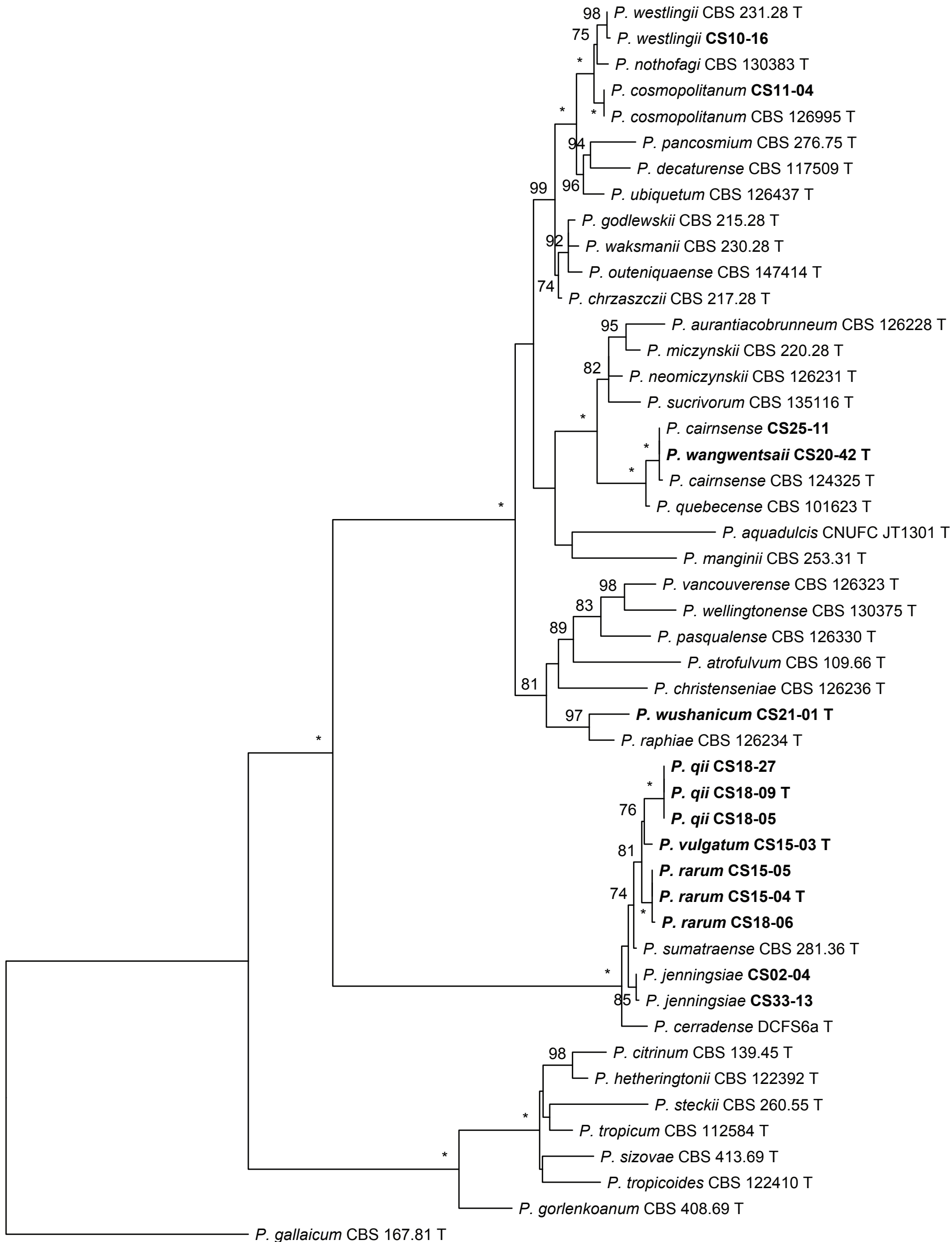

Supplement: Supplementary file 1 [file jof-09-01150-s001.zip › Figure S8 Citrina CaM.pdf]

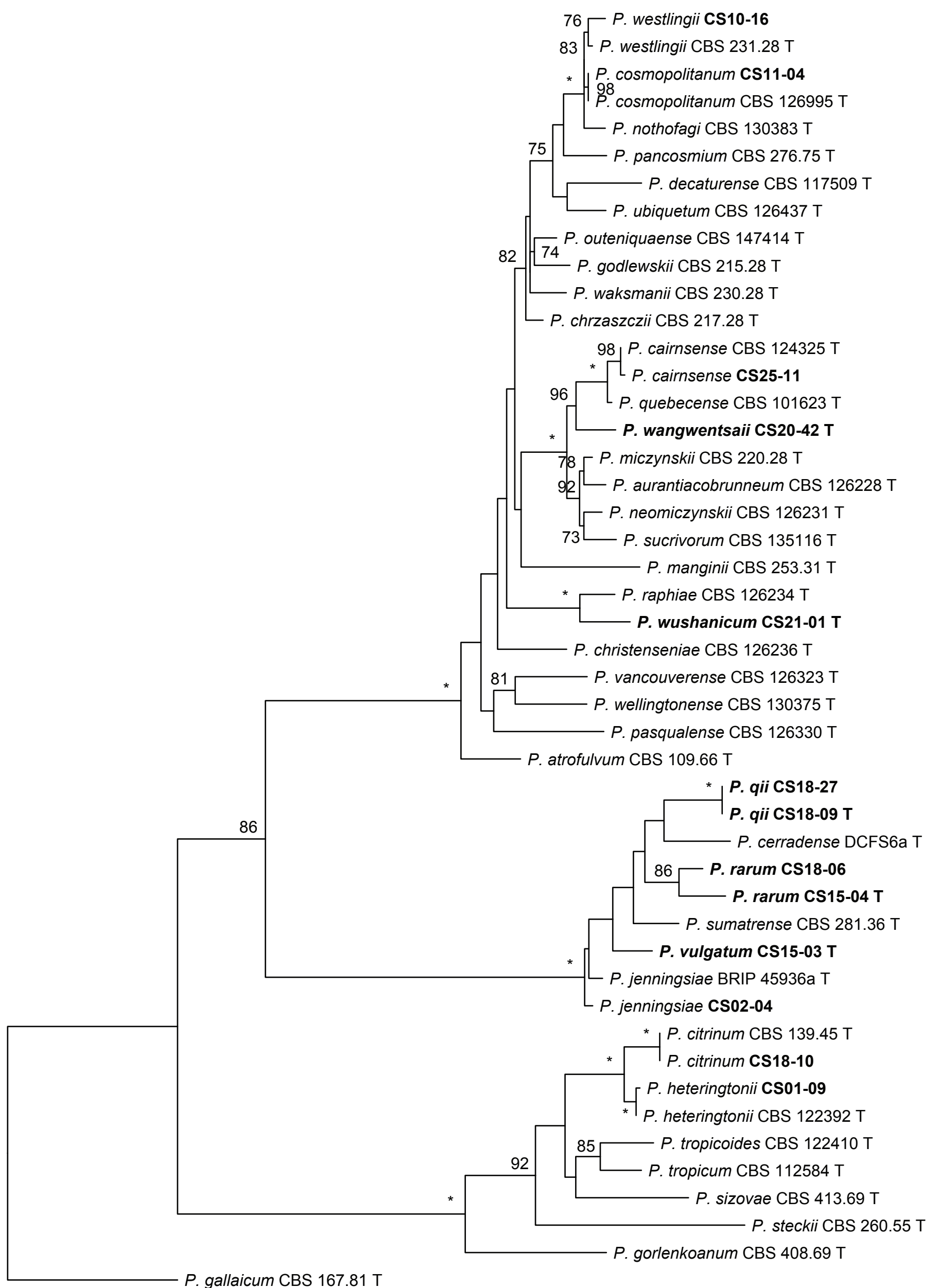

Supplement: Supplementary file 1 [file jof-09-01150-s001.zip › Figure S9 Citrina RPB2.pdf]
