# Supplementary material for: Species Diversity of Penicillium in Southwest China with Discovery of Forty-Three New Species
Source: J Fungi (Basel). 2023 Nov 28;9(12):1150. doi: 10.3390/jof9121150 (PMC10744262; doi:10.3390/jof9121150)
Supplement: Supplementary file 1 [file Figure S12 Exilicaulis RPB2.pdf › Figure S12 Exilicaulis RPB2.pdf]

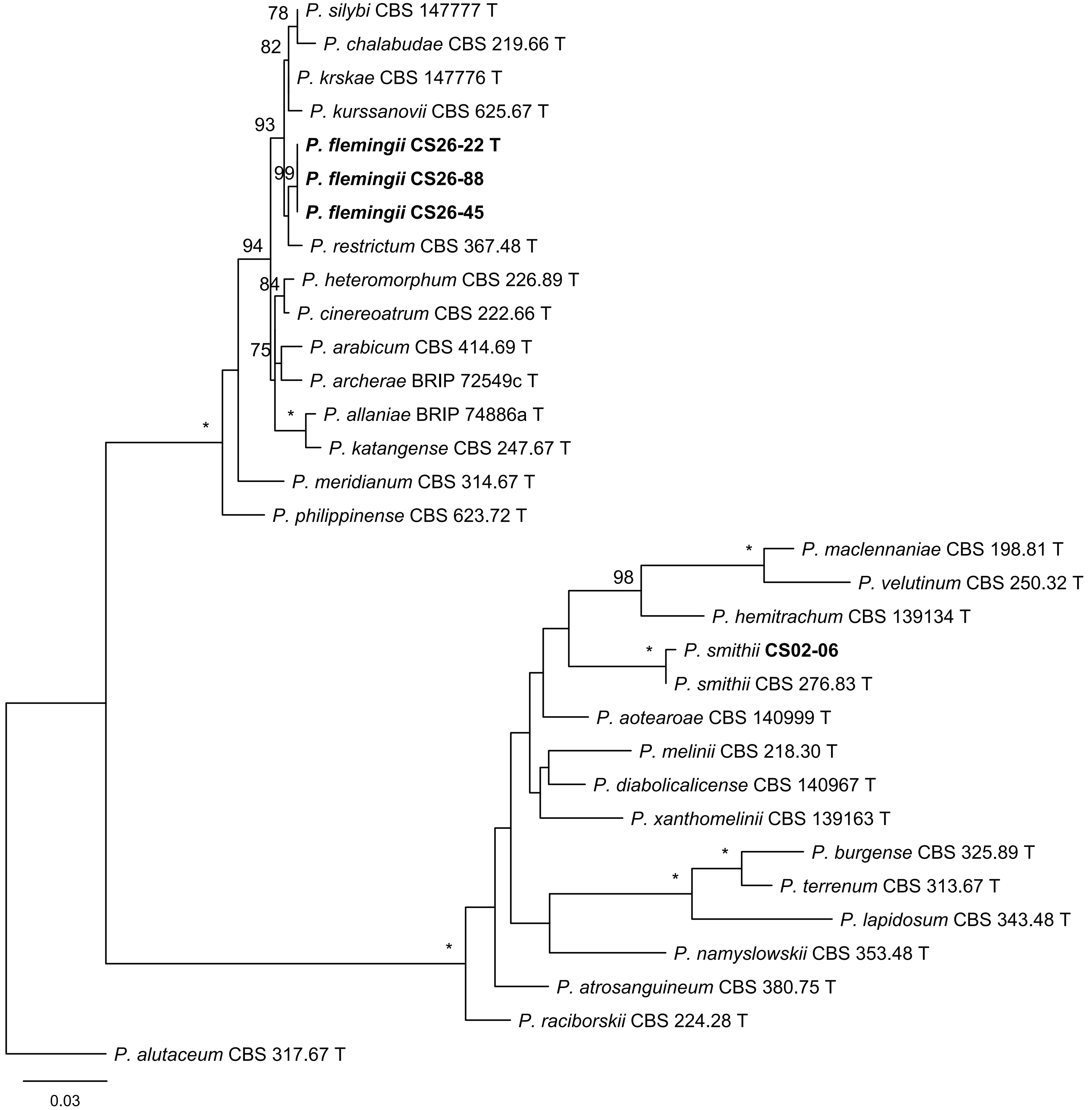

78
P. silybi CBS 147777 T
P. chalabudae CBS 219.66 T
82
P. krskae CBS 147776 T
P. kurssanovii CBS 625.67 T
93
P. flemingii CS26-22 T
99
P. flemingii CS26-88
P. flemingii CS26-45
P. restrictum CBS 367.48 T
94
P. heteromorphum CBS 226.89 T
84
P. cinereoatrum CBS 222.66 T
P. arabicum CBS 414.69 T
75
P. archerae BRIP 72549c T
*
P. allaniae BRIP 74886a T
P. katangense CBS 247.67 T
P. meridianum CBS 314.67 T
P. philippinense CBS 623.72 T
*
P. maclennaniae CBS 198.81 T
98
P. velutinum CBS 250.32 T
P. hemitrachum CBS 139134 T
*
P. smithii CS02-06
P. smithii CBS 276.83 T
P. aotearoae CBS 140999 T
P. melinii CBS 218.30 T
P. diabolicalicense CBS 140967 T
P. xanthomelinii CBS 139163 T
*
P. burgense CBS 325.89 T
*
P. terrenum CBS 313.67 T
P. lapidosum CBS 343.48 T
P. namyslowskii CBS 353.48 T
P. atrosanguineum CBS 380.75 T
P. raciborskii CBS 224.28 T
P. alutaceum CBS 317.67 T
0.03
